# A comparison of twenty-six baits for isolating *Calonectria* species illustrates differences in their efficacy and relevance

**DOI:** 10.3897/imafungus.17.190896

**Published:** 2026-09-09

**Authors:** HuiXia Zhou, HongBin Dong, WenWen Zhou, Michael J. Wingfield, ShuaiFei Chen

**Affiliations:** 1 State Key Laboratory of Agricultural and Forestry Biosecurity, Forest Pathogen Research Center, College of Forestry, Fujian Agriculture and Forestry University, Fuzhou, 350002, Fujian Province, China State Key Laboratory of Agricultural and Forestry Biosecurity, Forest Pathogen Research Center, College of Forestry, Fujian Agriculture and Forestry University Fuzhou China; 2 Department of Biochemistry, Genetics and Microbiology, Forestry and Agricultural Biotechnology Institute (FABI), University of Pretoria, Pretoria 0028, South Africa Department of Biochemistry, Genetics and Microbiology, Forestry and Agricultural Biotechnology Institute (FABI), University of Pretoria Pretoria South Africa

**Keywords:** Baiting assay systems, forest fungi, pathogen induction, plant pathogen, soil baiting, soil-borne fungi

## Abstract

*Calonectria* is a genus of plant-pathogenic fungi that are soil-borne and widely distributed globally. Baiting with plant tissues is commonly used to isolate these *Calonectria* spp. from soils, but the choice of effective baits is typically not based on experimental evidence. In this study, samples of forest soil were collected from five provinces in southeastern, southern, northeastern, central, and southwestern China. A total of 1,040 soil samples were exposed to 26 different baits, resulting in 1,282 *Calonectria* isolates. Based on DNA sequence analyses of the *tef1*, *tub2*, *cmdA*, and *his3* gene regions, the isolates were identified as eight *Calonectria* spp. The number of species, *tef1*-*tub2* genotypes, and isolates obtained from each of the 26 baits was compared. The efficacies and diversities of the baits for detecting *Calonectria* were evaluated for the genus as a whole and for each of the eight *Calonectria* spp. At both the genus and species levels, leaf baits performed better than seed baits, and several baits had consistently higher efficacy. Germinating *Medicago sativa* (alfalfa) seed baits, commonly used in previous studies, were not the most efficient means of isolating *Calonectria* from the soil, and seeds of *Lythrum salicaria* provided comparable results. *Eucalyptus urophylla* leaves were most effective in isolating *Calonectria pseudoreteaudii*, which is an important *Eucalyptus* leaf blight pathogen. This suggests that baiting with leaves of the target host plant could produce optimum results when considering a particular pathogen. Based on the overall results, combined baiting with seeds of *M. sativa* or *L. salicaria* and leaves of a variety of plants, including, where possible, the target host plant, is recommended to obtain *Calonectria* from soil samples in China and other regions of the world.

## Introduction

The genus *Calonectria (Nectriaceae)* includes many aggressive plant pathogens that cause diseases of forest tree species, agronomic crop plants, and ornamentals (Crous 2002; Lombard et al. 2010; Li et al. 2022). These fungi mostly have broad host ranges and have been shown to infect at least 611 plant species in 139 families (https://fungi.ars.usda.gov/). *Calonectria* spp. are particularly well known to cause diseases of forestry crops, such as leaf and shoot blight of *Eucalyptus* spp., especially in China, southeastern Asia, and South America (Old et al. 2003; Freitas et al. 2019; Pham et al. 2019; Li et al. 2023). Examples of diseases of agronomic crops include red crown rot of soybean (*Glycine max*) (Ochi et al. 2011; Gai et al. 2017) and black pod rot of peanut (*Arachis hypogaea*) (Gai et al. 2017). Additionally, some *Calonectria* spp. cause diseases of ornamental plants, such as boxwood (*Buxus* spp.) blight (LeBlanc et al. 2018, 2019) and leaf spots on *Callistemon* spp., *Metrosideros* spp., and *Myrtus* sp. (Lombard et al. 2011; Vitale et al. 2013).

*Calonectria* spp. are soil-borne fungi that are widely distributed, especially in forest soils, including both plantations and natural forests (Crous 2002; Daughtrey 2019; Li et al. 2022; Pham et al. 2022a; Liu and Chen 2023). The survival of their chlamydospores and microsclerotia enables these pathogens to persist in soils for long periods of time (Crous 2002; Li et al. 2022). Approximately 137 *Calonectria* spp. have been identified worldwide based on DNA sequence comparisons (Liu et al. 2020, 2026; Li et al. 2022; https://fungi.ars.usda.gov/). Of these, at least 84 species have been isolated from soils in or near agricultural areas, plantations, and natural forests in Asia, Africa, North America, and South America (Liu et al. 2020, 2026; https://fungi.ars.usda.gov/).

Successful isolation of *Calonectria* from soils represents a fundamental component of studies of these fungi and approaches to controlling the diseases that they cause. These isolations typically involve baiting with plant tissues or germinating seeds. In addition to germinating seeds, studies have used baits including leaf discs, twig segments, and seedling stem segments (Linderman 1972; Hwang and Ko 1976; Hunter et al. 1980; Gonçalves et al. 2001; Crous 2002; Dart et al. 2014; Hong et al. 2022). An unusual example was reported by Griffin (1960), who successfully isolated *Calonectria* sp. from soil using human hair as bait. Among the various baits that have been used in previous studies, germinating *Medicago sativa* seeds have been most commonly utilized (Lombard et al. 2015; Liu et al. 2021; Pham et al. 2022a; Wu and Chen 2023). Following this technique, *M. sativa* seeds are scattered onto moistened soil and incubated at 25 °C for 6–7 days, after which *Calonectria* spp. present in the soils produce conidiophores on the seedling tissues from which cultures can easily be obtained (Crous 2002; Liu and Chen 2017, 2023; Pham et al. 2022b; Liu et al. 2024).

An important question relating to soil baiting is whether the chosen baits provide the greatest richness and highest diversity of the *Calonectria* species present in the soils. Previous studies evaluating baits for isolating *Calonectria* from soil have been limited to one or a few baits (Gonçalves et al. 2001; Dart et al. 2014). The differences in the diversity of *Calonectria* isolated using different baits were not comparatively evaluated (Linderman 1972; Hwang and Ko 1976; Hunter et al. 1980; Gonçalves et al. 2001; Dart et al. 2014; Hong et al. 2022). In most previous research, *Calonectria* isolates were identified solely based on the morphological characteristics of conidia and vesicles (Linderman 1972; Hunter et al. 1980). Additionally, there is a lack of systematic and comprehensive research evaluating the genetic diversity and richness of specific *Calonectria* spp. isolated from soil using different baits.

The aim of this study was to compare a wide range of plant baits to effectively isolate *Calonectria* species from forest soils collected from distinct climatic zones of China. To achieve this objective, 26 baits, including *M. sativa* seeds, were used to isolate *Calonectria* from forest soils collected from five provinces in southeastern, southern, northeastern, central, and southwestern regions of the country. The *Calonectria* isolates were identified using DNA sequence comparisons and phylogenetic analyses based on four gene loci. This made it possible to compare and evaluate the efficacy of the various baits and to refine future studies of *Calonectria* in Chinese forest soils.

## Materials and methods

### Bait selection

Plant seeds, leaves, and stem segments that were successfully used in previous studies were tested as baits to isolate *Calonectria* species from soil samples. These tested baits satisfied at least one of the following three criteria: (1) the bait had been used to successfully isolate *Calonectria* in a previous study; (2) *Calonectria* had been isolated from this plant species in a natural environment; and (3) the family to which the plant species belongs included a relatively high number of *Calonectria* host species (>10 species). Additionally, to isolate *Calonectria* within a short period of time, the baits were required to satisfy the following conditions: (1) successfully isolate *Calonectria* within no more than 15 days; and (2) in the case of seed baits, the seeds should be less than 5 mm in length to be accommodated in an isolation cup and germinate within 2 days. Based on these criteria, 24 baits (bait IDs 1–24) were selected (Table 1). These included seeds of nine plant species, leaves of 14 plant species, and soybean stem segments (Table 1). Furthermore, human hair, which was used to isolate *Calonectria* in a previous study (Griffin 1960), was included. *Medicago sativa* seeds, widely used to bait *Calonectria* spp. (Crous 2002; Lombard et al. 2015; Pham et al. 2022a; Liu et al. 2024), were included as a positive control. In all, a total of 26 baits were used in this study. Seeds were commercially sourced from Taobao Network Co., Ltd. (Yuhang District, Hangzhou City, Zhejiang Province, China). Plant leaves were collected from Fujian Agriculture and Forestry University (Jinshan Campus) (Cangshan District, Fuzhou City, Fujian Province, China). Soybean stem segments were collected from plants grown for 1–2 weeks after germination.

**Table 1.** Twenty-six different baits used in this study.

**Bait type**	**Bait (ID number & species)^a^**	**Order and family**	**Bait previously used**	***Calonectria* previously isolated**	**Days to bait *Calonectria* in soil**	**Average seed length and seed germination data**
**Seed**	**1_*Brassica rapa* subsp. *pekinensis* Seed**	*Brassicales*, *Brassicaceae*		No	8 d	1.85 mm, 1 d
	**2_*Zinnia elegans* Seed**	*Asterales*, *Asteraceae*		No	8 d	6.75 mm, 2 d
	**3_*Lolium perenne* Seed**	*Poales*, *Poaceae*		No	15 d	5.64 mm, 2 d
	**4_*Salvia hispanica* Seed**	*Lamiales*, *Lamiaceae*		No	9 d	1.97 mm, 1 d
	**5_*Impatiens balsamina* Seed**	*Ericales*, *Balsaminaceae*		No	9 d	3.21 mm, 2 d
	**6_*Sesamum indicum* Seed**	*Lamiales*, *Pedaliaceae*		No	7 d	3.18 mm, 1d
	**7_*Dianthus caryophyllus* Seed**	*Caryophyllales*, *Caryophyllaceae*		Yes	8 d	2.05 mm, 2 d
	**8_*Trifolium pratense* Seed**	*Fabales*, *Fabaceae*		Yes	7 d	2.76 mm, 1 d
	**9_*Lythrum salicaria* Seed**	*Myrtales*, *Lythraceae*		No	7 d	0.80 mm, 3 d
**Leaf**	**10*_Buxus sinica* Leaf**	*Buxales*, *Buxaceae*	*Buxus sempervirens* leaf discs (Dart et al. 2014); *Buxus microphylla* cuttings (Hong et al. 2022)	Yes	11 d	N/A ^b^
	**11*_Imperata cylindrica* Leaf**	*Poales*, *Poaceae*		No	7 d	N/A
	**12*_Pinus massoniana* Leaf**	*Pinales*, *Pinaceae*		Yes	6 d	N/A
	**13*_Eucalyptus urophylla* hybrid Leaf**	*Myrtales*, *Myrtaceae*	*Eucalyptus grandis* leaf discs and twig segments (Gonçalves et al. 2001)	Yes	6 d	N/A
	**14*_Syzygium jambos* Leaf**	*Myrtales*, *Myrtaceae*		No	7 d	N/A
	**15*_Pennisetum purpureum* Leaf**	*Poales*, *Poaceae*		No	4 d	N/A
	**16*_Saccharum officinarum* Leaf**	*Poales*, *Poaceae*		Yes	5 d	N/A
	**17_*Rosa chinensis* Leaf**	*Rosales*, *Rosaceae*		No	4 d	N/A
	**18*_Acacia mangium* Leaf**	*Fabales*, *Fabaceae*		Yes	5 d	N/A
	**19*_Dianthus caryophyllus* Leaf**	*Caryophyllales*, *Caryophyllaceae*		Yes	5 d	N/A
	**20*_Cinnamomum camphora* Leaf**	*Laurales*, *Lauraceae*		Yes	7 d	N/A
	**21*_Washingtonia filifera* Leaf**	*Arecales*, *Arecaceae*		No	5 d	N/A
	**22*_Ficus benjamina* Leaf**	*Rosales*, *Moraceae*		Yes	5 d	N/A
	**23*_Rhododendron pulchrum* Leaf**	*Ericales*, *Ericaceae*	*Rhododendron obtusum* leaves (Linderman 1972); *Rhododendron* sp. leaf discs and twig segments (Gonçalves et al. 2001); *Rhododendron* sp. leaf discs (Dart et al. 2014)	No	8 d	N/A
**Stem**	**24_*Glycine max* Stem**	*Fabales*, *Fabaceae*		Yes	5 d	N/A
**Not plant**	**25_Human Hair**	N/A	Human head hair (Griffin 1960)	N/A	Not available	N/A
**Control**	**26_*Medicago sativa* Seed**	*Fabales*, *Fabaceae*	*Medicago sativa* seeds (Crous 2002)	Yes	6 d	2.30 mm, 1 d

^a^ Baits ID numbers refer to those in the Figs 4, 5. ^b^N/A: information not available.

### Soil sampling

**Figure 1.**
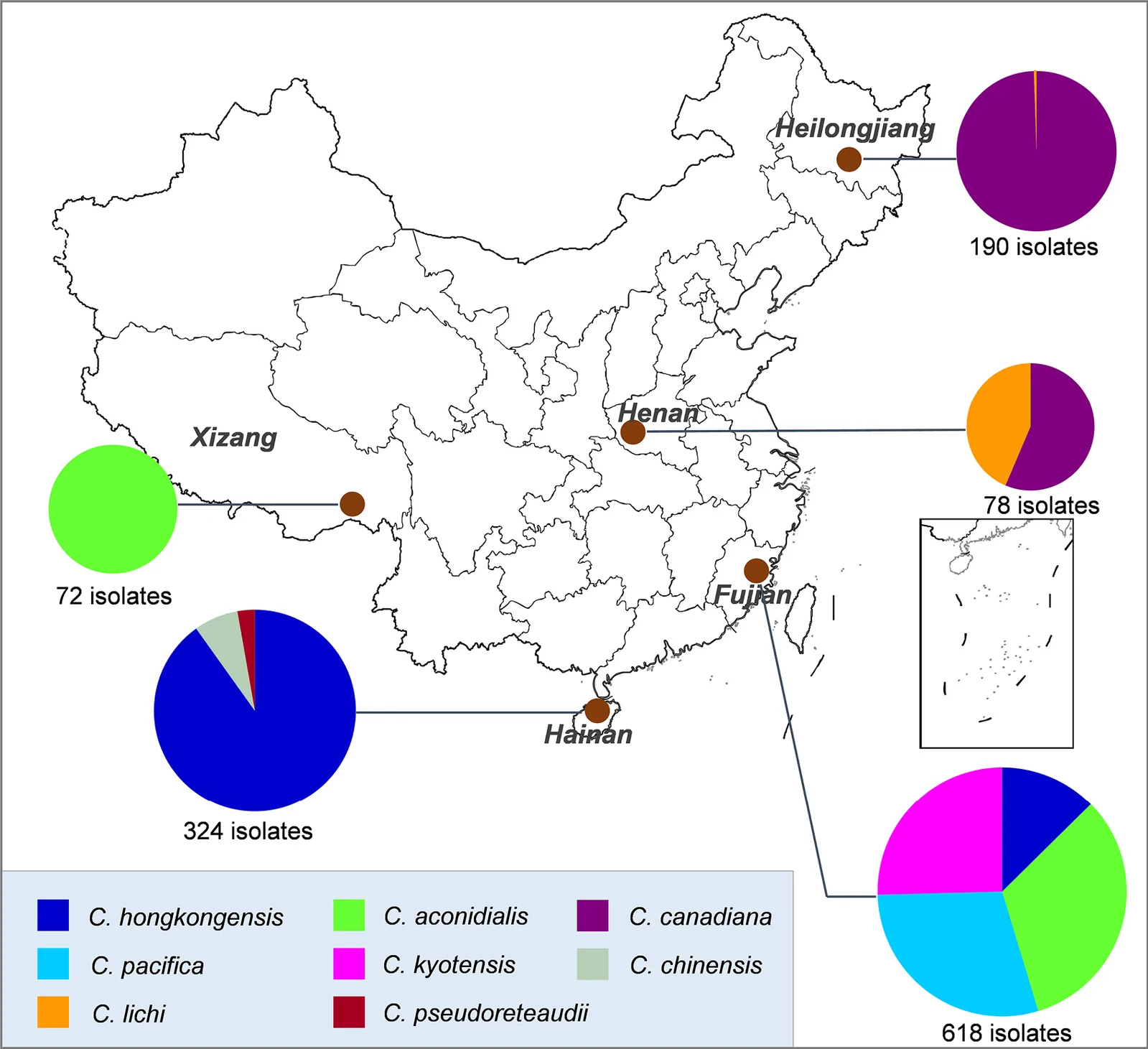
Map of China showing the eight sites in the five provinces (Heilongjiang, Henan, Xizang, Fujian, and Hainan) where soil samples were collected. Pie-charts show the percentage of isolates for *Calonectria* spp. detected in each of the five provinces. Different *Calonectria* spp. are indicated by different colors.

To obtain *Calonectria* with high species and genetic diversity, soil was collected from eight forests in five provinces and autonomous regions of China located in different climatic zones. These sites included forests at eight sites in Fujian (3), Hainan (2), Heilongjiang (1), Henan (1), and Xizang (1) (Table 2, Fig. 1). Details including the geographic region, climatic conditions, GPS coordinates, and altitude of these sites are provided in Table 2. Samples were collected from Xizang in October 2023, from Fujian and Hainan in January 2024, and from Heilongjiang and Henan from August to October 2024.

**Table 2.** Soil samples collected from eight sites in five provinces used in the current study.

**Sampling site**	**Province**	**Geographic region in China & Climatic zone**	**GPS coordinate & altitude**	**Collector**
Site1: Jingxi Town, Minhou County, Fuzhou Region, Fujian Province	Fujian	southeastern China; subtropical	26°10'14.3399"N, 119°14'31.1927"E; 114.7 m	S.F. Chen, Y. Liu, H.X. Zhou, W.W. Zhou, H.B. Dong
Site 2: Jingxi Town, Minhou County, Fuzhou Region, Fujian Province	Fujian	southeastern China; subtropical	26°8'55.0788"N, 119°13'5.5812"E; 148.7 m	S.F. Chen, Y. Liu, H.X. Zhou, W.W. Zhou, H.B. Dong
Site 3: Zhuangbian Town, Hanjiang District, Putian Region, Fujian Province	Fujian	southeastern China; subtropical	25°38'45.5532"N, 118°58'36.6888"E; 162.5 m	S.F. Chen, Y. Liu, H.X. Zhou, W.W. Zhou, H.B. Dong
Site 4: Huangtong Town, Lingao County, Hainan Province	Hainan	southern China; tropical	19°47'41.46"N, 109°49'52.7196"E; 99.1 m	S.F. Chen, Y. Liu, H.X. Zhou
Site 5: Huangtong Town, Lingao County, Hainan Province	Hainan	southern China; tropical	19°47'53.7072"N, 109°49'28.2648"E; 82.7 m	S.F. Chen, Y. Liu, H.X. Zhou
Site 6: Pingshan Town, Acheng District, Harbin Region, Heilongjiang Province	Heilongjiang	northeastern China; cold temperate	45°15'1.4400"N, 127°19'49.2024"E; 279.85 m	S.F. Chen, H.X. Zhou, W.W. Zhou, Y.B. Deng, W.Z. Liu
Site 7: Luanchuan Town, Luanchuan County, Luoyang Region, Henan Province	Henan	central China; warm temperate	33°44'58.9596"N, 111°38'13.578"E; 943.99 m	S.F. Chen, H.X. Zhou, W.W. Zhou, J.W. Liu
Site 8: Motuo Town, Motuo County, Linzhi Region, Xizang Autonomous Region	Xizang	western China; subtropical	29°17'1.158"N, 95°15'44.046"E; 804.59 m	S.F. Chen, Y. Liu, F.Y. Han, H.B. Dong, F. Shi

Soil samples were collected from natural forests in Henan, Heilongjiang, and Xizang and from *E. urophylla × E. grandis* plantations in Fujian and Hainan. The *Eucalyptus* trees were more than 5 years old, and both the natural forests and plantations had thick layers of leaf litter, which were removed before soil sample collection. Samples consisted of a mixture of soil from the surface to a depth of 50 cm. For each sample, approximately 0.02 m^3^ of soil was collected and placed in sturdy plastic bags. Samples were promptly transported to the laboratory and stored at 25 °C for baiting, isolation of *Calonectria*, and identification of isolates using DNA sequencing methods.

### Selection of soil samples for baiting

Prior to comparing different baits, soil samples were tested for the presence of *Calonectria* spp. To achieve this goal, a total of 40 soil samples were collected from the eight sites in five provinces (Table 2, Fig. 1). Each of these soil samples was mixed thoroughly. The mixed soil in each of the 40 bags was transferred to five plastic isolation cups (diameter = 4.4 cm, height = 5.5 cm, and volume = 80 mL; Chengdu Rich Science Industry Co., Ltd., Chengdu, China). The soil in these cups was moistened with sterile water and mixed well using a sterilized bamboo stick. Surface-disinfested *M. sativa* seeds were scattered onto the soil surface, and the samples were incubated at 25 °C. After 7 days of incubation, each isolation cup was examined under a dissection microscope for the presence of conidiophores with typical morphological characteristics of *Calonectria* (Crous 2002).

For each of the eight sampling sites, one soil sample shown to be positive for *Calonectria* was used to compare the isolation efficacy of 26 baits. In total, this provided eight soil samples from eight sites, including Fujian (3), Hainan (2), Heilongjiang (1), Henan (1), and Xizang (1), for bait testing (Table 2, Fig. 1).

### Baiting treatment and *Calonectria* isolation

For each of the eight soil samples, five replicate isolation cups were used for each of the 26 baits. This resulted in a total of 1,040 isolation cups (five isolation cups/bait/soil sample × 26 baits × eight soil samples = 1,040 isolation cups). Each of the 26 baits was dipped in 75% ethanol for 30 s and then washed three times with sterile water. The leaves were cut into 3–4 mm pieces using sterilized scissors. The soils placed in the isolation cups filled two-thirds of the cup volume and were moistened with sterile water and mixed well using a sterilized bamboo stick. Each of the 26 baits was placed on the soil surface in each isolation cup, and these were incubated at 25 °C.

All isolation cups were examined daily for 15 days for the presence of *Calonectria* conidiophores (Crous 2002; Li et al. 2022) using a dissection microscope. Where present, conidial masses were transferred using a sterilized needle onto 2% malt extract agar (MEA) containing 16 g malt extract (Qingdao Hope Bio-Technology Co., Ltd., Qingdao, China) and 16 g agar (Beijing Solarbio Science & Technology Co., Ltd., Beijing, China) per 800 mL of water. After incubation at 25 °C for 3–4 h, germinated conidia were transferred individually onto fresh MEA and incubated at 25 °C for 6–7 days to obtain single-conidium cultures. Two single-conidium cultures were obtained from each sampling cup, with each taken from a different position on the bait. All single-conidium cultures were deposited in the Culture Collection (CSFF) of the Forest Pathogen Center at the College of Forestry of Fujian Agriculture and Forestry University, Fuzhou, Fujian Province, China.

### Isolate identification

Single conidial isolates were grown on 2% MEA at 25 °C for 7 days; mycelium was scraped from the surface of the cultures using a sterilized bamboo skewer and transferred to a 2 mL Eppendorf tube. The total genomic DNA of each isolate was extracted using the CTAB method described by van Burik et al. (1998). Extracted DNA was dissolved by adding 30 μL of TE buffer (1 M Tris-HCl and 0.5 M EDTA, pH 8.0), and 2.5 μL of RNase (10 mg/mL) was added for RNA degradation. The mixture was incubated at 37 °C for 1 h. A NanoDrop Lite Plus spectrophotometer (Thermo Fisher Scientific, Waltham, MA, USA) was used to measure the DNA concentrations. All DNA samples were diluted to approximately 100 ng/μL by adding DEPC-treated water (Sangon Biotech Co., Ltd., Shanghai, China) and stored at –4 °C for further use.

Based on previous research (Liu et al. 2020, 2021; Wu and Chen 2021; Liu and Chen 2023), four gene regions, namely, translation elongation factor 1-alpha (*tef1*), β-tubulin (*tub2*), histone H3 (*his3*), and calmodulin (*cmdA*), were chosen as reliable DNA barcodes to identify the *Calonectria* spp. These four gene regions were amplified using the primer pairs and PCR protocols described by Liu et al. (2020). All PCR products were sequenced in both directions using the same primers used for PCR amplification. The PCR products were sequenced by the Beijing Genomics Institute located in Guangzhou, China. All initial sequences were edited using MEGA v. 7.0 software (Kumar et al. 2016). All sequences obtained in this study were deposited in GenBank (http://www.ncbi.nlm.nih.gov).

The *tef1* and *tub2* gene regions were amplified and sequenced for all isolates. The *tef1* and *tub2* sequences were used in a standard nucleotide BLAST search in the NCBI database (https://blast.ncbi.nlm.nih.gov) for preliminary species identification and to determine the complexes in which the isolates reside. Based on preliminary identification, representative isolates for all the genotypes determined by *tef1* and *tub2* sequences were selected for PCR amplification and sequencing of the *his3* and *cmdA* regions. Data for all isolates with *tef1*, *tub2*, *cmdA*, and *his3* sequences were used for phylogenetic analyses. Sequences of the isolates from the type specimens of all published species in the relevant *Calonectria* species complexes were used in these analyses. The sequence datasets were aligned using the online version of MAFFT v. 7 (Katoh and Standley 2013) (http://mafft.cbrc.jp/alignment/server) with an FFT–NS–i strategy (slow; interactive refinement method). The aligned datasets were curated in MEGA v. 6.0.5 software (Tamura et al. 2013).

Sequence datasets for each of the four gene regions and a concatenated dataset for those regions were used for phylogenetic inference. PhyML v. 3.0 (Guindon and Gascuel 2003) was used to perform maximum likelihood (ML) analyses. Sequence data for two isolates of *Curvicladiella cignea* (CBS 109167 and CBS 109168) were used as outgroups (Liu et al. 2020). For phylogenetic analyses, the best substitution model for each of the five datasets was determined using JModeltest 2.1.7 (Posada 2008). Bootstrap support for ML analyses was estimated using 1,000 replicates to assess the reliability of the inferred phylogenies. Phylogenetic trees were viewed using MEGA v. 7.0. Based on phylogenetic analyses and sequence comparisons, the *Calonectria* isolates obtained in this study were identified.

### Comparison of bait efficacy

The number of soil samples from which *Calonectria* spp. were obtained using the 26 baits was recorded. For each of the 26 baits, the percentage of sampling cups that successfully yielded *Calonectria* isolates was determined. The efficacy of obtaining *Calonectria* for both the genus as a whole and each species was computed and compared across the baits. At the genus level, the number of species in the genus (NSG) and number of isolates of the genus (NIG) isolated using each of the 26 baits were calculated. For each *Calonectria* species, the genotypes of the isolates were determined using the *tef1* and *tub2* sequences, and the number of genotypes of each species (NGS) and the number of isolates of each species (NIS) collected using each bait were calculated and compared. To determine whether the baits differed significantly and were equally influenced across climatic zones, analyses of variance (ANOVA) and multiple comparisons among means were conducted using SPSS Statistics 19 software (IBM Corp., Armonk, NY, USA) to compare the NSG and NIG obtained from each soil sample cup.

## Results

### Baiting treatment and *Calonectria* isolation

A total of 1,040 isolation cups were exposed to the 26 baits and examined for 15 days. No *Calonectria* fruiting bodies were found on the human hair samples. With the exception of *Lolium perenne* seeds and *Buxus sinica* leaves, which yielded conidiophores with morphological characteristics typical of *Calonectria* after 15 and 11 days, respectively, all other 23 baits displayed *Calonectria* sporulation within 10 days. Baits such as leaves of *Dianthus caryophyllus*, *Pennisetum purpureum*, and *Rosa chinensis* showed *Calonectria* sporulation within 4–5 days (Table 1). *Calonectria* spp. were found on baits in 641 of the 1,040 isolation cups tested. Other *Nectriaceae*, including *Cylindrocladiella*, *Gliocladiopsis*, and *Neocosmospora*, were also frequently observed on the baits. Taking two single-conidium isolates from each of these isolation cups resulted in a collection of 1,282 *Calonectria* cultures for further study (Suppl. material 1: table S1).

### Isolate identification

The *tef1* and *tub2* gene regions were sequenced for all 1,282 isolates (Suppl. material 1: table S1), and their genotypes were recorded. This resulted in 20 genotypes for *tef1* sequences, 28 genotypes for *tub2* sequences, and 41 genotypes for the combination of *tef1* and *tub2* sequences. Based on these 41 genotypes, one to four representative isolates of each genotype were further selected for sequencing of the *his3* and *cmdA* gene regions.

The criteria for selecting isolates for *his3* and *cmdA* gene sequencing were as follows: (1) four isolates were selected for each *tef1*-*tub2* genotype in each province; (2) these isolates were obtained from different soil collection bags and isolated from different baits, as far as possible; and (3) for genotypes represented by fewer than four isolates, all isolates of that genotype were selected. Following this approach, a total of 142 isolates were selected for sequencing of the *his3* and *cmdA* gene regions, and 49 genotypes were generated based on the *tef1*, *tub2*, *cmdA*, and *his3* sequences, enabling their identification (Suppl. material 1: table S2). The remaining 1,140 isolates were identified based exclusively on the *tef1* and *tub2* sequences.

Phylogenetic analyses were performed using the 142 isolates representing 49 genotypes based on four gene sequences (Suppl. material 1: table S2). A standard nucleotide BLAST search using the *tef1*, *tub2*, *cmdA*, and *his3* gene sequences indicated that the isolates belonged to three *Calonectria* species complexes. These were the *Calonectria kyotensis* species complex, *Ca. colhounii* species complex, and *Ca. reteaudii* species complex. The sequences of 101 isolates (ex-type and other strains) of 57 *Calonectria* spp. in these three species complexes were retrieved from GenBank (Suppl. material 1: table S3). These sequences were used in phylogenetic analyses of the four individual gene regions and a combination of these four gene regions.

The sequenced isolates yielded approximately 500 bp for the *tef1*, 565 bp for the *tub2*, 440 bp for the *his3*, and 685 bp for the *cmdA* gene regions. The model for each gene region dataset was selected based on JModeltest v. 2.1.5. The TIM2+G, HKY+I+G, TIM1+G, TIM3+I+G, and GTR+I+G models were selected for *tef1*, *tub2*, *cmdA*, *his3*, and the concatenated dataset, respectively. ML trees displaying the bootstrap values from ML analyses are shown in Suppl. material 1: figs S1–S5.

Based on the combined gene phylogenies (Suppl. material 1: fig. S1), 142 isolates clustered in eight groups (Groups 1–8). Of these, 128 isolates belonged to the *Ca. kyotensis* species complex and were identified as six species (Groups 1–6; Suppl. material 1: fig. S1). A total of 29 isolates (including CSFF27699) grouped with *Ca. hongkongensis* (Group 1), 46 (including CSFF27794) with *Ca. kyotensis* (Group 2), nine (including CSFF28233) with *Ca. chinensis* (Group 3), four (including CSFF27818) with *Ca. pacifica* (Group 4), 22 (including CSFF28421) with *Ca. aconidialis* (Group 5), and 18 (including CSFF31784) with *Ca. canadiana* (Group 6). Five isolates (including CSFF28154) were in the *Ca. reteaudii* species complex and clustered with *Ca. pseudoreteaudii* (Group 7). Nine isolates (including CSFF31597) belonged to the *Ca. colhounii* species complex and grouped with *Ca. lichi* (Group 8).

**Figure 2.**
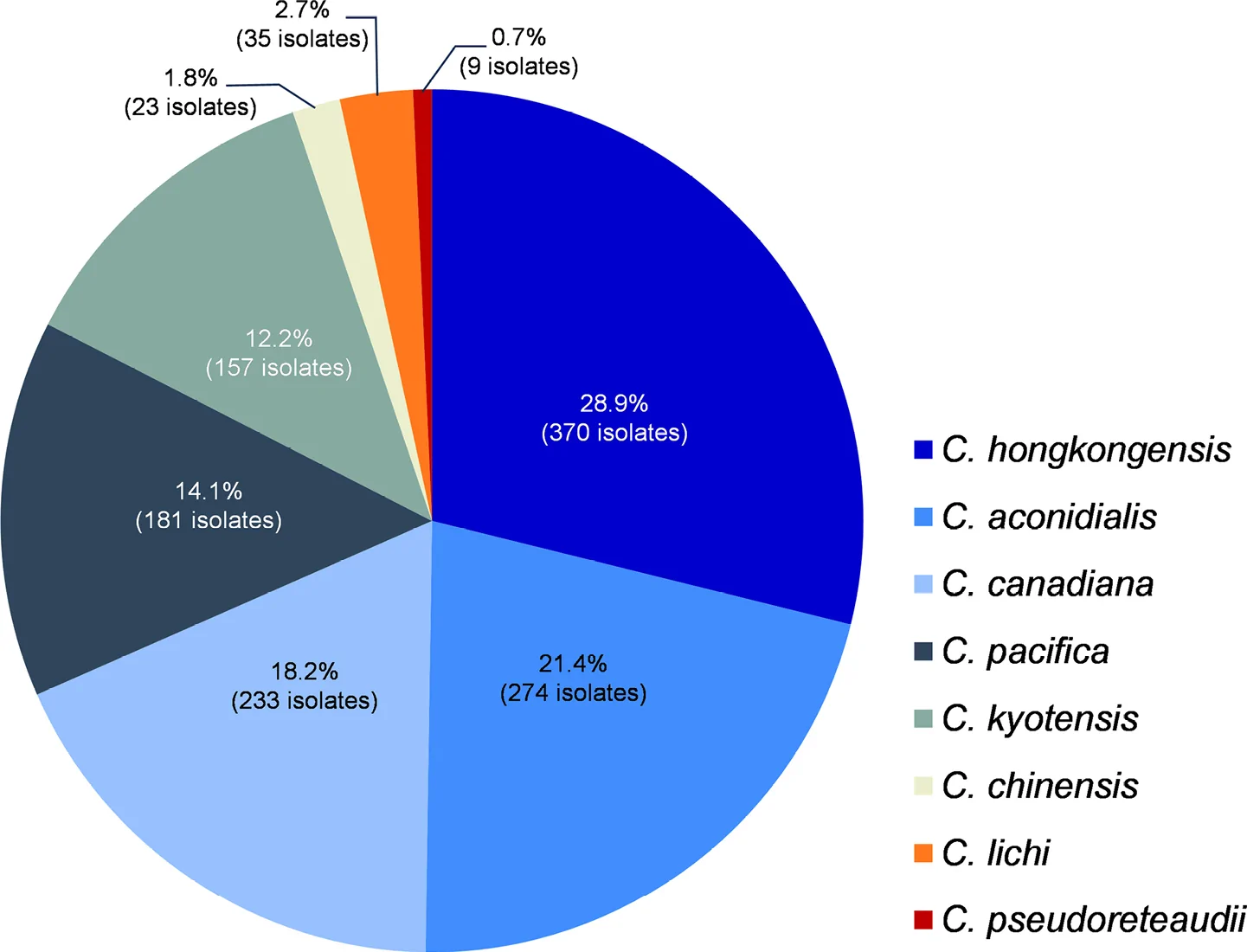
Number of isolates and percentage of each of the eight *Calonectria* spp. recovered from forest soils.

All 1,282 *Calonectria* isolates identified based on two to four gene regions were identified as *Ca. hongkongensis* (370 isolates; 28.9%), *Ca. aconidialis* (274 isolates; 21.4%), *Ca. canadiana* (233 isolates; 18.2%), *Ca. pacifica* (181 isolates; 14.1%), *Ca. kyotensis* (157 isolates; 12.2%), *Ca. chinensis* (23 isolates; 1.8%), *Ca. lichi* (35 isolates; 2.7%), and *Ca. pseudoreteaudii* (nine isolates; 0.7%) (Fig. 2). The first six species belonged to the *Ca. kyotensis* species complex. *Calonectria lichi* resides in the *Ca. colhounii* species complex, and *Ca. pseudoreteaudii* is part of the *Ca. reteaudii* species complex.

Of the 1,282 *Calonectria* isolates identified as eight species, 41 *tef1*-*tub2* genotypes were generated based on the *tef1* and *tub2* gene sequences. These included *Ca. hongkongensis* (7), *Ca. aconidialis* (7), *Ca. canadiana* (4), *Ca. pacifica* (1), *Ca. kyotensis* (14), *Ca. chinensis* (3), *Ca. lichi* (3), and *Ca. pseudoreteaudii* (2).

### Geographic distribution of isolated species

The percentages of isolation cups that yielded *Calonectria* in Fujian, Hainan, Heilongjiang, Henan, and Xizang were 79.2%, 62.3%, 73.1%, 30.0%, and 27.7%, respectively (Table 3). The number of isolates and their identifications are provided in Table 3. *Calonectria hongkongensis* was isolated from Hainan and Fujian soil samples, *Ca. aconidialis* from Fujian and Xizang, and *Ca. canadiana* from Heilongjiang and Henan. All *Ca. pacifica* and *Ca. kyotensis* isolates were obtained from Fujian, and all *Ca. chinensis* and *Ca. pseudoreteaudii* isolates were from Hainan. *Calonectria lichi* was predominantly isolated from Henan, with a small number from Heilongjiang (Suppl. material 1: fig. S6). Other than *Ca. canadiana* and *Ca. lichi*, which were found in both mid-temperate (Heilongjiang) and warm-temperate zones (Henan), all other species were found in tropical (Hainan) and subtropical (Fujian and Xizang) regions (Table 2; Suppl. material 1: fig. S6).

**Table 3.** Collection data for soil samples and *Calonectria* spp. from five provinces in China.

**Province**	**Number of soil samples treated**	**Number of isolation cups for soil samples baited**	**Number of isolation cups positive for *Calonectria***	**Percentage of isolation cups positive for *Calonectria***	**Number of *Calonectria* isolates collected**	**Number of *Calonectria* isolates per soil sample**	**Number of different *Calonectria* species**
**Fujian**	3	390	309	79.2%	618	206	4
**Hainan**	2	260	162	62.3%	324	162	3
**Heilongjiang**	1	130	95	73.1%	190	190	2
**Henan**	1	130	39	30.0%	78	78	2
**Xizang**	1	130	36	27.7%	72	72	1
**In total**	8	1040	641	61.6%	1282	160	8

Results showed that the efficacy of the baits was not significantly influenced across tropical, subtropical, warm temperate, and mid-temperate zones. They also showed that there was a proportional relationship between the number of soil samples and the number of different fungal species isolated (Table 3). Statistical analyses of multiple comparisons indicated that NSG and NIG values from each soil sample cup were not significantly influenced by the climatic regions where soil samples had been collected (Suppl. material 1: figs S7, S8). For example, both the NSGs and NIGs from Hainan (tropical zone) were significantly (*P* < 0.05) greater than those in Henan (warm temperate zone). In contrast, the NSGs and NIGs of Xizang (subtropical zone) were significantly (*P* < 0.05) smaller than those of Heilongjiang (mid-temperate zone) (Suppl. material 1: figs S7, S8).

### Comparison of bait efficacy

#### Percentage of soil sample yielding Calonectria

**Figure 3.**
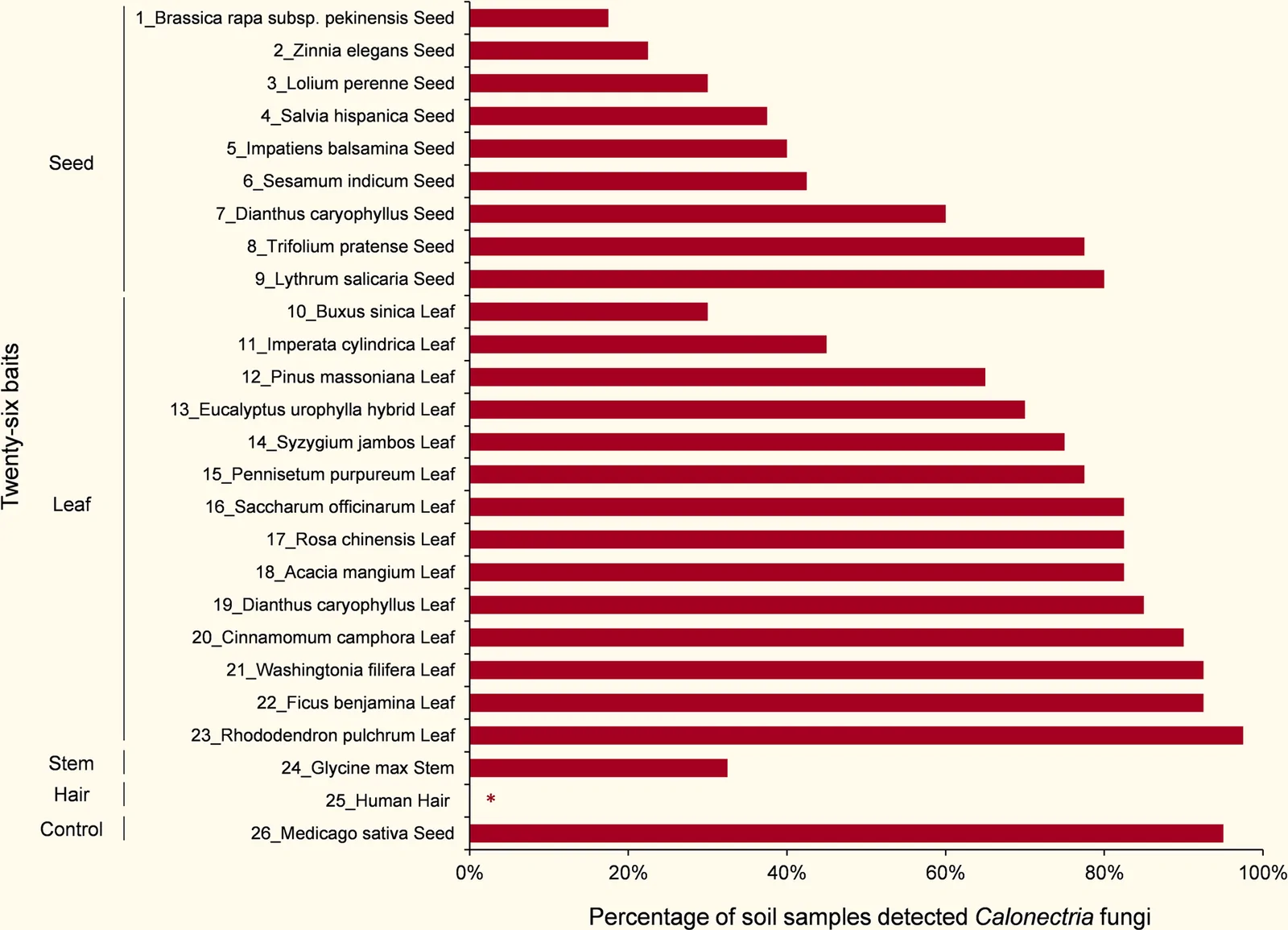
Percentage of forest soil samples in which *Calonectria* was detected using the 26 baits. “*” refers to zero.

With the exception of human hair, all baits yielded *Calonectria* isolates, with success rates ranging from 17.5 to 97.5%. *Rhododendron pulchrum* leaves had the highest success rate (97.5%), which was higher than that of the positive control (*M. sativa* seed; 95.0%). Baits with success rates below 40% were *Brassica rapa* subsp. *pekinensis* seed (17.5%), *Zinnia elegans* seed (22.5%), *Lo. perenne* seed (30.0%), *Salvia hispanica* seed (37.5%), *Bu. sinica* leaves (30.0%), and *G. max* stems (32.5%) (Fig. 3).

#### Number of species and isolates baited

**Figure 4.**
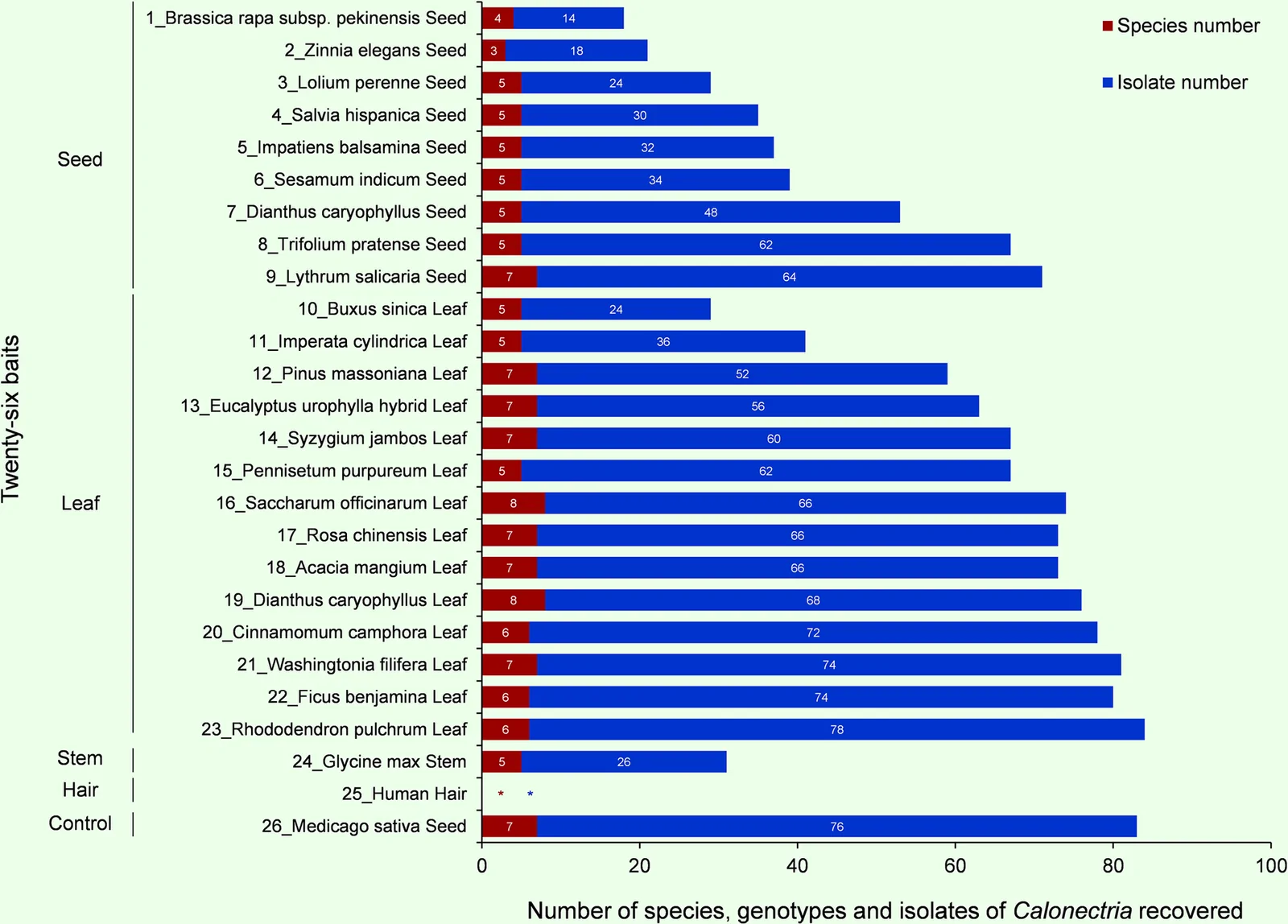
Number of isolates and *Calonectria* spp. recovered from forest soil samples using the 26 different baits. “*” refers to zero.

For the eight *Calonectria* species identified in this study, *Saccharum officinarum* and *D. caryophyllus* leaves each yielded all eight species (Fig. 4). *Lythrum salicaria* seed, *Pinus massoniana* needles, *E. urophylla* hybrid leaves, *Syzygium jambos* leaves, *Ro. chinensis* leaves, *Acacia mangium* leaves, *Washingtonia filifera* leaves, and *M. sativa* seed yielded seven species. *Zinnia elegans* seed (three species) and *Br. rapa* subsp. *pekinensis* seed (four species) yielded fewer species than other baits. The average number of species yielded by the 14 leaf baits (average of 6.5 species) was greater than that of the 10 seed baits (average of 5.1 species) (Fig. 4). Multiple-comparison results indicated that the NSGs of *Sac. officinarum*, *Ro. chinensis*, *Ac. mangium*, *D. caryophyllus*, *Cinnamomum camphora*, *W. filifera*, *Ficus benjamina*, and *Rh. pulchrum* leaves were significantly (*P* < 0.05) greater than those of seeds of *Br. rapa* subsp. *pekinensis*, *Z. elegans*, *Lo. perenne*, *Sal. hispanica*, *Impatiens balsamina*, and *Sesamum indicum* (Suppl. material 1: fig. S9).

*Rhododendron pulchrum* leaves yielded the largest number of isolates, followed by the positive control (*M. sativa* seed) (Fig. 4). Other baits that yielded more than 65 isolates included leaves of *W. filifera*, *F. benjamina*, *Ci. camphora*, *D. caryophyllus*, *Sac. officinarum*, *Ro. chinensis*, and *Ac. mangium*. In contrast, seeds of *Br. rapa* subsp. *pekinensis* and *Z. elegans* yielded fewer than 20 isolates. Overall, the 14 leaf baits yielded a greater number of isolates (average of 61 isolates) than the 10 seed baits (average of 40.2 isolates) (Fig. 4). The multiple-comparison results indicated that the NIGs of leaves of *Sac. officinarum*, *Ro. chinensis*, *Ac. mangium*, *D. caryophyllus*, *Ci. camphora*, *W. filifera*, *F. benjamina*, and *Rh. pulchrum* were significantly (*P* < 0.05) greater than those of seeds of *Br. rapa* subsp. *pekinensis*, *Z. elegans*, *Lo. perenne*, *Sal. hispanica*, *Impa. balsamina*, and *Ses. indicum* (Suppl. material 1: fig. S10).

Among the 25 baits that successfully yielded *Calonectria*, the NSG and NIG obtained using *D. caryophyllus* leaves were 2.7 and 3.8 times those obtained using *Z. elegans* seed, respectively (Fig. 4). The multiple-comparison results showed that the differences were significant (NSG, *P* < 0.01; NIG, *P* < 0.01) (Suppl. material 1: figs S9, S10). Among the 10 seed baits, *Ly. salicaria* seed yielded 1.8 times more species and 4.6 times more isolates than *Br. rapa* subsp. *pekinensis* seed, respectively (Fig. 4), and the differences were significant (NSG, *P* < 0.01; NIG, *P* < 0.01) (Suppl. material 1: figs S9, S10). Among the 14 leaf baits, *Sac. officinarum* leaves yielded 1.6 times more species and 2.8 times more isolates than *Bu. sinica* leaves, respectively (Fig. 4), and the differences were significant (NSG, *P* < 0.01; NIG, *P* < 0.01) (Suppl. material 1: figs S9, S10). *Glycine max* stems yielded fewer species than 11 of the 14 leaf baits and yielded fewer isolates than all leaf baits other than *Bu. sinica* (Fig. 4). The multiple-comparison results showed that the NSG and NIG of the *G. max* stem bait were significantly (*P* < 0.05) lower than those of all leaf baits other than *Bu. sinica*, *Imperata cylindrica*, *Pi. massoniana*, *E. urophylla* hybrid, *Syz. jambos*, and *Pe. purpureum* (Suppl. material 1: figs S9, S10).

Based on a comprehensive evaluation of the NSG and NIG, *D. caryophyllus* leaves (NSG = 8, NIG = 68), *Sac. officinarum* leaves (NSG = 8, NIG = 66), *M. sativa* seed (NSG = 7, NIG = 76), *W. filifera* leaves (NSG = 7, NIG = 74), *Ro. chinensis* leaves (NSG = 7, NIG = 66), *Ac. mangium* leaves (NSG = 7, NIG = 66), and *Ly. salicaria* seed (NSG = 7, NIG = 64) were identified as the most effective baits. These were followed by *Syz. jambos* leaves (NSG = 7, NIG = 60) and *E. urophylla* hybrid leaves (NSG = 7, NIG = 56) (Table 4, Fig. 4).

**Table 4.** Ranking of the 26 baits for their efficacy in isolating *Calonectria* spp. from forest soils.

**Bait typ**e	**Bait number and name**	**Total *Calonectria* species^a^**	***Ca. hongkongensis* ^b^**	***Ca. aconidialis* ^b^**	***Ca. canadiana* ^b^**	***Ca. pacifica* ^b^**	***Ca. kyotensis* ^b^**	***Ca. chinensis* ^b^**	***Ca. lichi* ^b^**	***Ca. pseudoreteaudii* ^b^**
**Seed**	**1_*Brassica rapa* subsp. *pekinensis* Seed**	24	25	23	23	25	22	4	11	5
	**2_*Zinnia elegans* Seed**	25	21	22	25	17	25	13	11	5
	**3_*Lolium perenne* Seed**	22	22	23	23	20	20	13	11	5
	**4_*Salvia hispanica* Seed**	20	24	16	17	20	23	13	11	5
	**5_*Impatiens balsamina* Seed**	19	17	20	17	20	17	13	11	5
	**6_*Sesamum indicum* Seed**	18	20	25	20	15	7	13	11	5
	**7_*Dianthus caryophyllus* Seed**	16	16	18	4	15	17	13	11	5
	**8_*Trifolium pratense* Seed**	14	9	4	13	8	5	13	11	5
	**9_*Lythrum salicaria* Seed**	7	2	10	13	1	14	7	11	4
**Leaf**	**10*_Buxus sinica* Leaf**	22	17	20	20	19	23	13	11	5
	**11*_Imperata cylindrica* Leaf**	17	17	12	20	23	1	13	11	5
	**12*_Pinus massoniana* Leaf**	10	14	15	12	1	15	7	6	5
	**13*_Eucalyptus urophylla* hybrid Leaf**	9	8	17	13	17	3	4	11	1
	**14*_Syzygium jambos* Leaf**	8	15	12	3	1	9	7	6	5
	**15*_Pennisetum purpureum* Leaf**	14	4	4	4	8	19	13	11	5
	**16*_Saccharum officinarum* Leaf**	2	12	4	8	8	13	1	6	2
	**17_*Rosa chinensis* Leaf**	5	3	2	8	8	3	7	6	5
	**18*_Acacia mangium* Leaf**	5	6	12	16	1	2	7	1	5
	**19*_Dianthus caryophyllus* Leaf**	1	12	9	11	8	10	7	4	2
	**20*_Cinnamomum camphora* Leaf**	13	11	4	2	1	10	1	11	5
	**21*_Washingtonia filifera* Leaf**	4	7	10	1	8	6	3	10	5
	**22*_Ficus benjamina* Leaf**	12	1	3	4	8	10	13	3	5
	**23*_Rhododendron pulchrum* Leaf**	11	5	8	7	1	16	13	2	5
**Stem**	***24_Glycine max* Stem**	21	23	18	19	24	7	13	11	5
**Not plant**	**25_Human Hair**	26	25	26	25	25	25	13	11	5
**Control**	**26_*Medicago sativa* Seed**	3	10	1	8	1	21	4	4	5

^a^ Ranking of the 26 baits for their efficacy in detecting *Calonectria* species (genus level), determined by the number of species and isolates of *Calonectria* detected. Ranks 1–5 or the top five are marked in dark green, 6–10 in light green, 11–21 in yellow, and 22–26 or the lowest five in light blue. ^b^ Ranking of the 26 baits for their efficacy in detecting each of the eight *Calonectria* spp., which were determined by the number of genotypes and isolates of each species. Ranks 1–5 or the top five are marked in dark green, 6–10 in light green, 11–21 in yellow, and 22–26 or the lowest five in light blue.

#### Number of genotypes and isolates baited for each species

**Figure 5.**
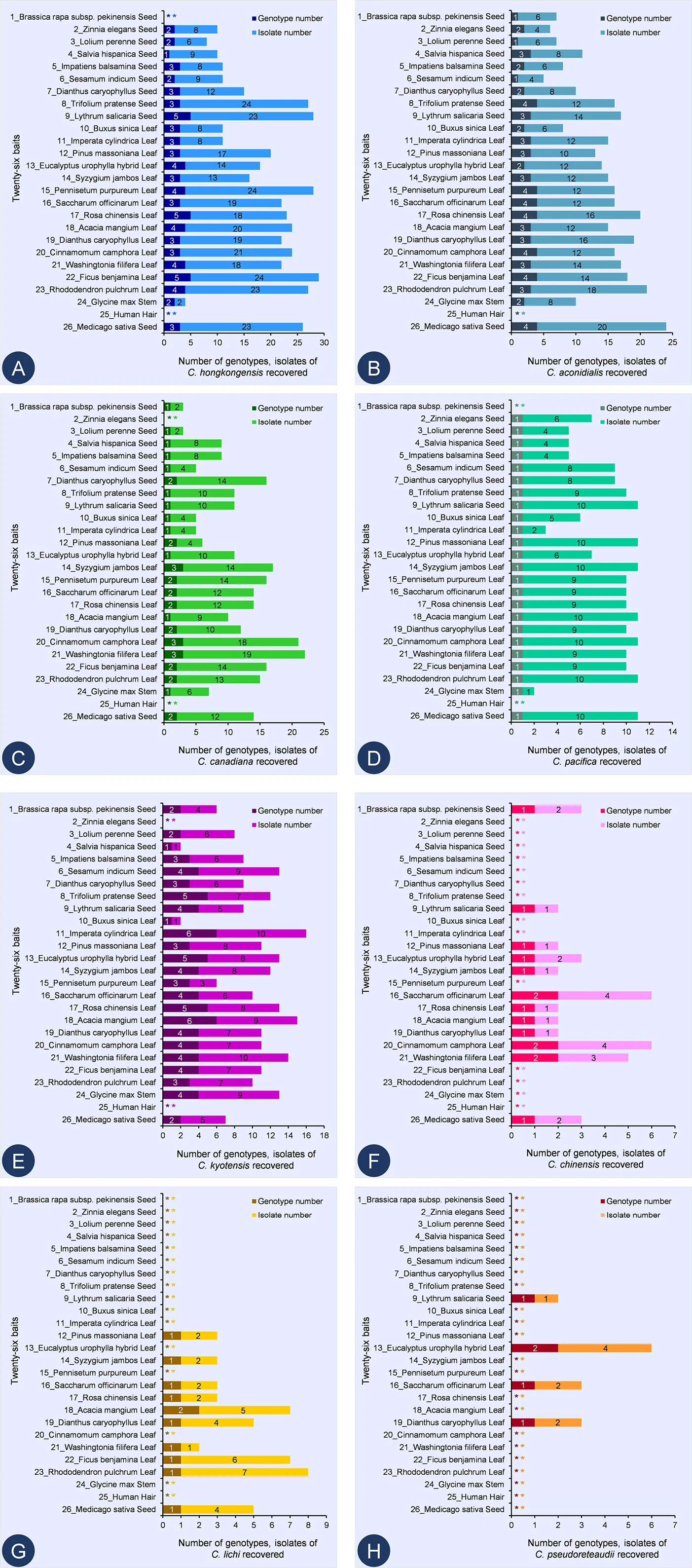
Number of isolates and genotypes of each *Calonectria* spp. recovered from forest soil samples using 26 baits. *Calonectria hongkongensis* (**A**), *Ca. aconidialis* (**B**), *Ca. canadiana* (**C**), *Ca. pacifica* (**D**), *Ca. kyotensis* (**E**), *Ca. chinensis* (**F**), *Ca. lichi* (**G**), *Ca. pseudoreteaudii* (**H**).

All 10 seed baits and 14 leaf baits yielded *Ca. aconidialis*. The percentage of leaf baits that detected each of the other seven species was higher than that of seed baits. In total, 90% (9/10) of seed baits and 100% (14/14) of leaf baits detected *Ca. hongkongensis*, *Ca. canadiana*, *Ca. pacifica*, and *Ca. kyotensis*; 30% (3/10) of seed baits and 64.3% (9/14) of leaf baits detected *Ca. chinensis*; 10% (1/10) of seed baits and 64.3% (9/14) of leaf baits detected *Ca. lichi*; and 10% (1/10) of seed baits and 21.4% (3/14) of leaf baits detected *Ca. pseudoreteaudii* (Fig. 5).

For each of the eight *Calonectria* species, the 14 leaf baits yielded more genotypes and isolates than the 10 seed baits. The average numbers of genotypes/isolates detected by the 14 leaf baits and the 10 seed baits were 3.6/17.6 and 2.4/12.2, 3.2/12.7 and 2.3/8.8, 1.9/11.2 and 1.1/7, 1/8.4 and 0.9/6.3, 4/7.1 and 2.6/4.9, 0.9/1.3 and 0.3/0.5, 0.7/2.2 and 0.1/0.4, and 0.3/0.6 and 0.1/0.1 for *Ca. hongkongensis*, *Ca. aconidialis*, *Ca. canadiana*, *Ca. pacifica*, *Ca. kyotensis*, *Ca. chinensis*, *Ca. lichi*, and *Ca. pseudoreteaudii*, respectively (Fig. 5).

The baits that yielded the most genotypes and isolates for *Ca. hongkongensis*, *Ca. aconidialis*, *Ca. canadiana*, *Ca. kyotensis*, *Ca. chinensis*, *Ca. lichi*, and *Ca. pseudoreteaudii* were *F. benjamina* leaves, *M. sativa* seed, *W. filifera* leaves, *Impe. cylindrica* leaves, *Sac. officinarum* leaves/*Ci. camphora* leaves, *Ac. mangium* leaves, and *E. urophylla* hybrid leaves, respectively (Fig. 5). The efficacies of each bait for detecting *Calonectria* spp. from soils differed. For *Ca. kyotensis*, *Impe. cylindrica* leaves yielded the most isolates and genotypes, but for *Ca. canadiana*, *Ca. pacifica*, *Ca. chinensis*, and *Ca. lichi*, leaves of this species were among the baits that yielded the fewest isolates and genotypes. *Glycine max* stems were among the baits that yielded the fewest isolates and genotypes of *Ca. hongkongensis*, *Ca. pacifica*, *Ca. chinensis*, *Ca. lichi*, and *Ca. pseudoreteaudii*. However, for *Ca. kyotensis*, *G. max* stems produced a larger number of isolates and genotypes than most other baits (Fig. 5).

Based on a comprehensive evaluation of NGS and NIS, with the exception of *Ca. pseudoreteaudii*, leaves of *Ro. chinensis*, *Ac. mangium*, *W. filifera*, *Sac. officinarum*, *Syz. jambos*, and *D. caryophyllus* were consistently effective baits for the isolation of each of the *Calonectria* species obtained in this study (Table 4, Fig. 5).

## Discussion

This study compared 26 different baits, including leaf tissues and germinating seeds, for their efficacy in isolating *Calonectria* from forest soils. The baits differed considerably in the number of species, the number of isolates, and the number of genotypes of the *Calonectria* species detected. Overall, the results showed that baits differ in their efficacy in isolating *Calonectria* from forest soils. The NSG and NIG results were significantly different for some baits. Furthermore, they provided evidence that the most effective leaf baits are those from host plants of the *Calonectria* species.

The efficacies of some baits were consistent, both for the genus as a whole and for individual *Calonectria* species. Five leaf baits (*Sac. officinarum*, *Ro. chinensis*, *W. filifera*, *Ac. mangium*, and *D. caryophyllus*) consistently yielded the greatest number of species and the highest number of isolates for the genus as a whole. With the exception of *Ca. pseudoreteaudii*, these baits were most effective in trapping the other seven *Calonectria* spp. found in this study. In contrast, with the exception of *Ca. chinensis*, germinating seeds of *Z. elegans*, *Br. rapa* subsp. *pekinensis*, *Lo. perenne*, and *Sal. hispanica* provided relatively poor results in trapping the eight *Calonectria* spp.

*Medicago sativa* seed has been widely used in previous studies to bait *Calonectria* from soils in several countries (Crous et al. 1997; Pham et al. 2019, 2022a; Liu et al. 2021; Wu and Chen 2021; Liu and Chen 2023). The results of the present study showed that *M. sativa* seed was not the most effective bait for isolating most *Calonectria* species. When considering the genus as a whole, the number of species isolated using germinating *M. sativa* seed was lower than that obtained using *Sac. officinarum* or *D. caryophyllus* leaves, and the number of isolates was also lower than that obtained using *Rh. pulchrum* leaves. For individual species, except for *Ca. aconidialis* and *Ca. pacifica*, for which *M. sativa* seed yielded the highest number of isolates, *M. sativa* seed yielded relatively fewer isolates or genotypes of the other six *Calonectria* species compared with some other baits.

Although germinating *M. sativa* seed did not emerge as the best bait in this study, it will likely remain useful for isolating *Calonectria* from soils. This is because it is readily available and can be easily stored and transported. However, the results showed that germinating *Ly. salicaria* seed performed equally well, detecting the same number of *Calonectria* species as *M. sativa* seed, and, in the case of *Ca. hongkongensis*, *Ca. kyotensis*, and *Ca. pseudoreteaudii*, *Ly. salicaria* seed yielded a greater number of genotypes. Based on the findings of this study, *Ly. salicaria* seed is considered an appropriate alternative bait to *M. sativa* seed.

*Calonectria pseudoreteaudii* was trapped by the lowest number of the tested baits. In this case, only four baits gave successful results, and these included leaves of *E. urophylla*, *Sac. officinarum*, *D. caryophyllus*, and *Ly. salicaria* seeds. This result is relevant because *Ca. pseudoreteaudii* is an important pathogen of *Eucalyptus* trees in China and elsewhere in Asia (Crous et al. 2012; Wu and Chen 2021; Bose et al. 2022; Li et al. 2023; Tarigan et al. 2023). The *E. urophylla* leaves yielded the largest number of isolates of *Ca. pseudoreteaudii*, and they represented several genotypes of the fungus. The result suggests that leaf baits, including a known host of a particular *Calonectria* species, should be included when baiting for this fungus.

## Conclusion

This study compared and evaluated 26 baits for isolating *Calonectria* species from forest soils. Based on the overall results, combining seed baits of *M. sativa* or *Ly. salicaria* and one or more leaf pieces of *Sac. officinarum*, *Ro. chinensis*, *W. filifera*, *Ac. mangium*, and *Rh. pulchrum* would give the most comprehensive results when seeking to isolate *Calonectria* from soils in China. *Calonectria* species in this study were obtained from tropical, subtropical, warm temperate, and mid-temperate zones of five provinces in China. The wide geographic and climatic origins of these fungi, together with their high species and genetic diversity, ensure that the appropriate baits selected in this study should be efficient for isolating *Calonectria* species from soils in diverse geographical and climatic regions in China and other regions of the world.

## Additional information

### Artificial Intelligence (AI) use

The authors accept full responsibility for the content of the manuscript, including the disclosure of any use of AI.

No AI tools were used in the preparation of this manuscript.

### Adherence to national and international regulations

All the fungal strains used in this study have been legally obtained, respecting the Convention on Biological Diversity (Rio Convention).

### Author ORCIDs

H. Zhou https://orcid.org/0009-0002-8514-1082

M.J. Wingfield https://orcid.org/0000-0001-9346-2009

S. Chen https://orcid.org/0000-0002-3920-9982

### Data availability

All sequence data obtained or used in this study are available in GenBank (https://www.ncbi.nlm.nih.gov/genbank/).

## Supplementary materials

Supplementary material 1 Additional tables and figures Data type docx Explanation note **table S1**. Information on the 1282 *Calonectria* isolates obtained and analyzed in this study. **table S2**. The obtained *Calonectria* isolates used for phylogenetic analyses in this study. **table S3**. *Calonectria* spp. and isolates from published studies used for phylogenetic analyses in this study. **fig. S1**. Phylogenetic tree of *Calonectria* spp. based on maximum likelihood (ML) analyses of the combined *tef1*, *tub2*, *cmdA*, and *his3* gene sequence dataset in this study. Bootstrap values ≥ 70% from the analysis are indicated at the nodes. Bootstrap values < 70% or absent are not displayed. Ex-type isolates are indicated with "T". The isolates reported in this study are highlighted in bold and blue. The outgroup taxon was *Curvicladiella cignea* (CBS 109167 and CBS 109168). **fig. S2**. Phylogenetic tree of *Calonectria* spp. based on maximum likelihood (ML) analyses of the *tef1* gene sequence dataset in this study. Bootstrap values ≥ 70% from the analysis are indicated at the nodes. Bootstrap values < 70% or absent are not displayed. Ex-type isolates are indicated with "T". The isolates reported in this study are highlighted in bold and blue. The outgroup taxon was *Curvicladiella cignea* (CBS 109167 and CBS 109168). **fig. S3**. Phylogenetic tree of *Calonectria* spp. based on maximum likelihood (ML) analyses of the *tub2* gene sequence dataset in this study. Bootstrap values ≥ 70% from the analysis are indicated at the nodes. Bootstrap values < 70% or absent are not displayed. Ex-type isolates are indicated with "T". The isolates reported in this study are highlighted in bold and blue. The outgroup taxon was *Curvicladiella cignea* (CBS 109167 and CBS 109168). **fig. S4**. Phylogenetic tree of *Calonectria* spp. based on maximum likelihood (ML) analyses of the *cmdA* gene sequence dataset in this study. Bootstrap values ≥ 70% from the analysis are indicated at the nodes. Bootstrap values < 70% or absent are not displayed. Ex-type isolates are indicated with "T". The isolates reported in this study are highlighted in bold and blue. The outgroup taxon was *Curvicladiella cignea* (CBS 109167 and CBS 109168). **fig. S5**. Phylogenetic tree of *Calonectria* spp. based on maximum likelihood (ML) analyses of the *his3* gene sequence dataset in this study. Bootstrap values ≥ 70% from the analysis are indicated at the nodes. Bootstrap values < 70% or absent are not displayed. Ex-type isolates are indicated with "T". The isolates reported in this study are highlighted in bold and blue. The outgroup taxon was *Curvicladiella cignea* (CBS 109167 and CBS 109168). **fig. S6**. Number of isolates and percentage of each of the eight *Calonectria* recovered from forest soils in each of the five provinces. https://binary.pensoft.net/file/1768317This dataset is made available under the Open Database License (http://opendatacommons.org/licenses/odbl/1.0/). The Open Database License (ODbL) is a license agreement intended to allow users to freely share, modify, and use this Dataset while maintaining this same freedom for others, provided that the original source and author(s) are credited. HuiXia Zhou, HongBin Dong, WenWen Zhou, Michael J. Wingfield, ShuaiFei Chen
