# Supplementary material for: A comparison of twenty-six baits for isolating Calonectria species illustrates differences in their efficacy and relevance
Source: IMA Fungus. 2026 Sep 9;17:e190896. doi: 10.3897/imafungus.17.190896 (PMC13584172; doi:10.3897/imafungus.17.190896)
Supplement: Supplementary file 1 [file imafungus-17-e190896-s001.docx]

**Supplementary material**

**Additional tables and figures**

**Table S1.** Information on the 1282 *Calonectria* isolates obtained and analyzed in this study.

**Table S2.** The obtained *Calonectria* isolates used for phylogenetic analyses in this study.

**Table S3.** *Calonectria* spp. and isolates from published studies used for phylogenetic analyses in this study.

**Figure S1.** Phylogenetic tree of *Calonectria* spp. based on maximum likelihood (ML) analyses of the combined *tef1*, *tub2*, *cmdA*, and *his3* gene sequence dataset in this study. Bootstrap values ≥ 70% from the analysis are indicated at the nodes. Bootstrap values < 70% or absent are not displayed. Ex-type isolates are indicated with "T". The isolates reported in this study are highlighted in bold and blue. The outgroup taxon was *Curvicladiella cignea* (CBS 109167 and CBS 109168).

**Figure S2.** Phylogenetic tree of *Calonectria* spp. based on maximum likelihood (ML) analyses of the *tef1* gene sequence dataset in this study. Bootstrap values ≥ 70% from the analysis are indicated at the nodes. Bootstrap values < 70% or absent are not displayed. Ex-type isolates are indicated with "T". The isolates reported in this study are highlighted in bold and blue. The outgroup taxon was *Curvicladiella cignea* (CBS 109167 and CBS 109168).

**Figure S3.** Phylogenetic tree of *Calonectria* spp. based on maximum likelihood (ML) analyses of the *tub2* gene sequence dataset in this study. Bootstrap values ≥ 70% from the analysis are indicated at the nodes. Bootstrap values < 70% or absent are not displayed. Ex-type isolates are indicated with "T". The isolates reported in this study are highlighted in bold and blue. The outgroup taxon was *Curvicladiella cignea* (CBS 109167 and CBS 109168).

**Figure S4.** Phylogenetic tree of *Calonectria* spp. based on maximum likelihood (ML) analyses of the *cmdA* gene sequence dataset in this study. Bootstrap values ≥ 70% from the analysis are indicated at the nodes. Bootstrap values < 70% or absent are not displayed. Ex-type isolates are indicated with "T". The isolates reported in this study are highlighted in bold and blue. The outgroup taxon was *Curvicladiella cignea* (CBS 109167 and CBS 109168).

**Figure S5.** Phylogenetic tree of *Calonectria* spp. based on maximum likelihood (ML) analyses of the *his3* gene sequence dataset in this study. Bootstrap values ≥ 70% from the analysis are indicated at the nodes. Bootstrap values < 70% or absent are not displayed. Ex-type isolates are indicated with "T". The isolates reported in this study are highlighted in bold and blue. The outgroup taxon was *Curvicladiella* *cignea* (CBS 109167 and CBS 109168).

**Figure S6.** Number of isolates and percentage of each of the eight *Calonectria* recovered from forest soils in each of the five provinces.

**Figure S7.** Column chart indicating average number of species in the genus (NSG) isolated from soil in each sample cup for eight bag of soil collected from five provinces. Horizontal bars represent the standard error of the means. Bars with different letters indicate treatment means that are significantly different (*P* = 0.05).

**Figure S8.** Column chart indicating average number of isolates of the genus (NIG) obtained from soil in each sample cup for eight bag of soil collected from five provinces. Horizontal bars represent the standard error of the means. Bars with different letters indicate treatment means that are significantly different (*P* = 0.05).

**Figure S9.** Column chart indicating average number of species in the genus (NSG) isolated from soil in each sample cup using the 26 differet baits. "*" refers to zero . Horizontal bars represent the standard error of the means. Bars with different letters indicate treatment means that are significantly different (*P* = 0.05).

**Figure S10.** Column chart indicating average number of isolates of the genus (NIG) obtained from soil in each sample cup using the 26 differet baits. "*" refers to zero . Horizontal bars represent the standard error of the means. Bars with different letters indicate treatment means that are significantly different (*P* = 0.05).

**Table S1.** Information on the 1282 *Calonectria* isolates obtained and analyzed in this study.

| **Sampling province** | **Sampling site No.** | **Total soil sample number/The soil sample number of relative province** | **Bait code and bait** | **The soil isolation cup number of relative bait** | **Isolate number^a^** | **Genotype determined by tef1, tub2, his3, and cmdA sequences^b^** | **Code of genotype determined by tef1, tub2, his3, and cmdA sequences^c^** | **Code of genotype determined by tef1, and tub2 sequences^d^** | ***Calonectria* species** | **GenBank accession numbers^e^** | | | |
| --- | --- | --- | --- | --- | --- | --- | --- | --- | --- | --- | --- | --- | --- |
|  |  |  |  |  |  |  |  |  |  | ***tef1*** | ***tub2*** | ***his3*** | ***cmdA*** |
| Fujian | Site 1 | 3/1 | 1_Brassica rapa subsp. pekinensis Seed | (1) | CSFF 27456 | T25_B20_N/A_N/A | N/A | TB genotype 35 | *Ca. kyotensis* | PV756199 | PV757765 | N/A | N/A |
| Fujian | Site 1 | 3/1 | 1_Brassica rapa subsp. pekinensis Seed | (1) | CSFF 27457 | T25_B20_N/A_N/A | N/A | TB genotype 35 | *Ca. kyotensis* | PV756200 | PV757766 | N/A | N/A |
| Fujian | Site 1 | 3/1 | 1_Brassica rapa subsp. pekinensis Seed | (5) | CSFF 27458 | T24_B17_H19_C15 | TBHC genotype 29 | TB genotype 26 | *Ca. kyotensis* | PV756201 | PV757767 | PV757451 | PV757593 |
| Fujian | Site 1 | 3/1 | 1_Brassica rapa subsp. pekinensis Seed | (5) | CSFF 27459 | T24_B17_H19_C15 | TBHC genotype 29 | TB genotype 26 | *Ca. kyotensis* | PV756202 | PV757768 | PV757452 | PV757594 |
| Fujian | Site 1 | 3/1 | 2_Zinnia elegans Seed | (3) | CSFF 27452 | T21_B13_N/A_N/A | N/A | TB genotype 20 | *Ca. hongkongensis* | PV756195 | PV757761 | N/A | N/A |
| Fujian | Site 1 | 3/1 | 2_Zinnia elegans Seed | (3) | CSFF 27453 | T21_B13_N/A_N/A | N/A | TB genotype 20 | *Ca. hongkongensis* | PV756196 | PV757762 | N/A | N/A |
| Fujian | Site 1 | 3/1 | 2_Zinnia elegans Seed | (5) | CSFF 27454 | T21_B13_N/A_N/A | N/A | TB genotype 20 | *Ca. hongkongensis* | PV756197 | PV757763 | N/A | N/A |
| Fujian | Site 1 | 3/1 | 2_Zinnia elegans Seed | (5) | CSFF 27455 | T21_B13_N/A_N/A | N/A | TB genotype 20 | *Ca. hongkongensis* | PV756198 | PV757764 | N/A | N/A |
| Fujian | Site 1 | 3/1 | 3_Lolium perenne Seed | (1) | CSFF 27478 | T24_B16_N/A_N/A | N/A | TB genotype 25 | *Ca. kyotensis* | PV756221 | PV757787 | N/A | N/A |
| Fujian | Site 1 | 3/1 | 3_Lolium perenne Seed | (1) | CSFF 27479 | T24_B16_N/A_N/A | N/A | TB genotype 25 | *Ca. kyotensis* | PV756222 | PV757788 | N/A | N/A |
| Fujian | Site 1 | 3/1 | 3_Lolium perenne Seed | (2) | CSFF 27480 | T25_B20_N/A_N/A | N/A | TB genotype 35 | *Ca. kyotensis* | PV756223 | PV757789 | N/A | N/A |
| Fujian | Site 1 | 3/1 | 3_Lolium perenne Seed | (2) | CSFF 27481 | T25_B20_N/A_N/A | N/A | TB genotype 35 | *Ca. kyotensis* | PV756224 | PV757790 | N/A | N/A |
| Fujian | Site 1 | 3/1 | 3_Lolium perenne Seed | (3) | CSFF 27482 | T21_B13_N/A_N/A | N/A | TB genotype 20 | *Ca. hongkongensis* | PV756225 | PV757791 | N/A | N/A |
| Fujian | Site 1 | 3/1 | 3_Lolium perenne Seed | (3) | CSFF 27483 | T21_B13_N/A_N/A | N/A | TB genotype 20 | *Ca. hongkongensis* | PV756226 | PV757792 | N/A | N/A |
| Fujian | Site 1 | 3/1 | 3_Lolium perenne Seed | (4) | CSFF 27484 | T24_B16_N/A_N/A | N/A | TB genotype 25 | *Ca. kyotensis* | PV756227 | PV757793 | N/A | N/A |
| Fujian | Site 1 | 3/1 | 3_Lolium perenne Seed | (4) | CSFF 27485 | T24_B16_N/A_N/A | N/A | TB genotype 25 | *Ca. kyotensis* | PV756228 | PV757794 | N/A | N/A |
| Fujian | Site 1 | 3/1 | 4_Salvia hispanica Seed | (1) | CSFF 27460 | T21_B13_N/A_N/A | N/A | TB genotype 20 | *Ca. hongkongensis* | PV756203 | PV757769 | N/A | N/A |
| Fujian | Site 1 | 3/1 | 4_Salvia hispanica Seed | (1) | CSFF 27461 | T21_B13_N/A_N/A | N/A | TB genotype 20 | *Ca. hongkongensis* | PV756204 | PV757770 | N/A | N/A |
| Fujian | Site 1 | 3/1 | 4_Salvia hispanica Seed | (2) | CSFF 27462 | T21_B13_N/A_N/A | N/A | TB genotype 20 | *Ca. hongkongensis* | PV756205 | PV757771 | N/A | N/A |
| Fujian | Site 1 | 3/1 | 4_Salvia hispanica Seed | (2) | CSFF 27463 | T21_B13_N/A_N/A | N/A | TB genotype 20 | *Ca. hongkongensis* | PV756206 | PV757772 | N/A | N/A |
| Fujian | Site 1 | 3/1 | 4_Salvia hispanica Seed | (3) | CSFF 27464 | T21_B13_N/A_N/A | N/A | TB genotype 20 | *Ca. hongkongensis* | PV756207 | PV757773 | N/A | N/A |
| Fujian | Site 1 | 3/1 | 4_Salvia hispanica Seed | (3) | CSFF 27465 | T21_B13_N/A_N/A | N/A | TB genotype 20 | *Ca. hongkongensis* | PV756208 | PV757774 | N/A | N/A |
| Fujian | Site 1 | 3/1 | 4_Salvia hispanica Seed | (4) | CSFF 27466 | T24_B16_N/A_N/A | N/A | TB genotype 25 | *Ca. kyotensis* | PV756209 | PV757775 | N/A | N/A |
| Fujian | Site 1 | 3/1 | 4_Salvia hispanica Seed | (4) | CSFF 27467 | T21_B13_N/A_N/A | N/A | TB genotype 20 | *Ca. hongkongensis* | PV756210 | PV757776 | N/A | N/A |
| Fujian | Site 1 | 3/1 | 4_Salvia hispanica Seed | (5) | CSFF 27468 | T21_B13_N/A_N/A | N/A | TB genotype 20 | *Ca. hongkongensis* | PV756211 | PV757777 | N/A | N/A |
| Fujian | Site 1 | 3/1 | 4_Salvia hispanica Seed | (5) | CSFF 27469 | T21_B13_N/A_N/A | N/A | TB genotype 20 | *Ca. hongkongensis* | PV756212 | PV757778 | N/A | N/A |
| Fujian | Site 1 | 3/1 | 5_Impatiens balsamina Seed | (1) | CSFF 27470 | T24_B24_H18_C15 | TBHC genotype 39 | TB genotype 33 | *Ca. kyotensis* | PV756213 | PV757779 | PV757453 | PV757595 |
| Fujian | Site 1 | 3/1 | 5_Impatiens balsamina Seed | (1) | CSFF 27471 | T24_B24_N/A_N/A | N/A | TB genotype 33 | *Ca. kyotensis* | PV756214 | PV757780 | N/A | N/A |
| Fujian | Site 1 | 3/1 | 5_Impatiens balsamina Seed | (2) | CSFF 27472 | T24_B20_N/A_N/A | N/A | TB genotype 29 | *Ca. kyotensis* | PV756215 | PV757781 | N/A | N/A |
| Fujian | Site 1 | 3/1 | 5_Impatiens balsamina Seed | (2) | CSFF 27473 | T24_B20_N/A_N/A | N/A | TB genotype 29 | *Ca. kyotensis* | PV756216 | PV757782 | N/A | N/A |
| Fujian | Site 1 | 3/1 | 5_Impatiens balsamina Seed | (4) | CSFF 27474 | T21_B14_H17_C11 | TBHC genotype 24 | TB genotype 21 | *Ca. hongkongensis* | PV756217 | PV757783 | PV757454 | PV757596 |
| Fujian | Site 1 | 3/1 | 5_Impatiens balsamina Seed | (4) | CSFF 27475 | T21_B14_N/A_N/A | N/A | TB genotype 21 | *Ca. hongkongensis* | PV756218 | PV757784 | N/A | N/A |
| Fujian | Site 1 | 3/1 | 5_Impatiens balsamina Seed | (5) | CSFF 27476 | T25_B20_N/A_N/A | N/A | TB genotype 35 | *Ca. kyotensis* | PV756219 | PV757785 | N/A | N/A |
| Fujian | Site 1 | 3/1 | 5_Impatiens balsamina Seed | (5) | CSFF 27477 | T25_B20_N/A_N/A | N/A | TB genotype 35 | *Ca. kyotensis* | PV756220 | PV757786 | N/A | N/A |
| Fujian | Site 1 | 3/1 | 6_Sesamum indicum Seed | (2) | CSFF 27436 | T24_B21_N/A_N/A | N/A | TB genotype 30 | *Ca. kyotensis* | PV756179 | PV757745 | N/A | N/A |
| Fujian | Site 1 | 3/1 | 6_Sesamum indicum Seed | (2) | CSFF 27437 | T24_B21_N/A_N/A | N/A | TB genotype 30 | *Ca. kyotensis* | PV756180 | PV757746 | N/A | N/A |
| Fujian | Site 1 | 3/1 | 6_Sesamum indicum Seed | (3) | CSFF 27438 | T21_B13_N/A_N/A | N/A | TB genotype 20 | *Ca. hongkongensis* | PV756181 | PV757747 | N/A | N/A |
| Fujian | Site 1 | 3/1 | 6_Sesamum indicum Seed | (3) | CSFF 27439 | T24_B19_N/A_N/A | N/A | TB genotype 28 | *Ca. kyotensis* | PV756182 | PV757748 | N/A | N/A |
| Fujian | Site 1 | 3/1 | 6_Sesamum indicum Seed | (4) | CSFF 27440 | T24_B19_N/A_N/A | N/A | TB genotype 28 | *Ca. kyotensis* | PV756183 | PV757749 | N/A | N/A |
| Fujian | Site 1 | 3/1 | 6_Sesamum indicum Seed | (4) | CSFF 27441 | T24_B19_N/A_N/A | N/A | TB genotype 28 | *Ca. kyotensis* | PV756184 | PV757750 | N/A | N/A |
| Fujian | Site 1 | 3/1 | 6_Sesamum indicum Seed | (5) | CSFF 27442 | T24_B16_N/A_N/A | N/A | TB genotype 25 | *Ca. kyotensis* | PV756185 | PV757751 | N/A | N/A |
| Fujian | Site 1 | 3/1 | 6_Sesamum indicum Seed | (5) | CSFF 27443 | T24_B16_N/A_N/A | N/A | TB genotype 25 | *Ca. kyotensis* | PV756186 | PV757752 | N/A | N/A |
| Fujian | Site 1 | 3/1 | 7_Dianthus caryophyllus Seed | (1) | CSFF 27444 | T24_B16_N/A_N/A | N/A | TB genotype 25 | *Ca. kyotensis* | PV756187 | PV757753 | N/A | N/A |
| Fujian | Site 1 | 3/1 | 7_Dianthus caryophyllus Seed | (1) | CSFF 27445 | T24_B16_N/A_N/A | N/A | TB genotype 25 | *Ca. kyotensis* | PV756188 | PV757754 | N/A | N/A |
| Fujian | Site 1 | 3/1 | 7_Dianthus caryophyllus Seed | (2) | CSFF 27446 | T24_B19_N/A_N/A | N/A | TB genotype 28 | *Ca. kyotensis* | PV756189 | PV757755 | N/A | N/A |
| Fujian | Site 1 | 3/1 | 7_Dianthus caryophyllus Seed | (2) | CSFF 27447 | T24_B19_N/A_N/A | N/A | TB genotype 28 | *Ca. kyotensis* | PV756190 | PV757756 | N/A | N/A |
| Fujian | Site 1 | 3/1 | 7_Dianthus caryophyllus Seed | (3) | CSFF 27448 | T24_B18_H19_C15 | TBHC genotype 31 | TB genotype 27 | *Ca. kyotensis* | PV756191 | PV757757 | PV757449 | PV757591 |
| Fujian | Site 1 | 3/1 | 7_Dianthus caryophyllus Seed | (3) | CSFF 27449 | T24_B18_H19_C15 | TBHC genotype 31 | TB genotype 27 | *Ca. kyotensis* | PV756192 | PV757758 | PV757450 | PV757592 |
| Fujian | Site 1 | 3/1 | 7_Dianthus caryophyllus Seed | (4) | CSFF 27450 | T21_B13_N/A_N/A | N/A | TB genotype 20 | *Ca. hongkongensis* | PV756193 | PV757759 | N/A | N/A |
| Fujian | Site 1 | 3/1 | 7_Dianthus caryophyllus Seed | (4) | CSFF 27451 | T21_B13_N/A_N/A | N/A | TB genotype 20 | *Ca. hongkongensis* | PV756194 | PV757760 | N/A | N/A |
| Fujian | Site 1 | 3/1 | 8_Trifolium pratense Seed | (1) | CSFF 27426 | T24_B20_H18_C14 | TBHC genotype 34 | TB genotype 29 | *Ca. kyotensis* | PV756169 | PV757735 | PV757446 | PV757588 |
| Fujian | Site 1 | 3/1 | 8_Trifolium pratense Seed | (1) | CSFF 27427 | T24_B20_N/A_N/A | N/A | TB genotype 29 | *Ca. kyotensis* | PV756170 | PV757736 | N/A | N/A |
| Fujian | Site 1 | 3/1 | 8_Trifolium pratense Seed | (2) | CSFF 27428 | T21_B13_N/A_N/A | N/A | TB genotype 20 | *Ca. hongkongensis* | PV756171 | PV757737 | N/A | N/A |
| Fujian | Site 1 | 3/1 | 8_Trifolium pratense Seed | (2) | CSFF 27429 | T25_B20_N/A_N/A | N/A | TB genotype 35 | *Ca. kyotensis* | PV756172 | PV757738 | N/A | N/A |
| Fujian | Site 1 | 3/1 | 8_Trifolium pratense Seed | (3) | CSFF 27430 | T24_B21_H20_C13 | TBHC genotype 36 | TB genotype 30 | *Ca. kyotensis* | PV756173 | PV757739 | PV757447 | PV757589 |
| Fujian | Site 1 | 3/1 | 8_Trifolium pratense Seed | (3) | CSFF 27431 | T25_B20_N/A_N/A | N/A | TB genotype 35 | *Ca. kyotensis* | PV756174 | PV757740 | N/A | N/A |
| Fujian | Site 1 | 3/1 | 8_Trifolium pratense Seed | (4) | CSFF 27432 | T24_B16_H18_C15 | TBHC genotype 28 | TB genotype 25 | *Ca. kyotensis* | PV756175 | PV757741 | PV757448 | PV757590 |
| Fujian | Site 1 | 3/1 | 8_Trifolium pratense Seed | (4) | CSFF 27433 | T21_B13_N/A_N/A | N/A | TB genotype 20 | *Ca. hongkongensis* | PV756176 | PV757742 | N/A | N/A |
| Fujian | Site 1 | 3/1 | 8_Trifolium pratense Seed | (5) | CSFF 27434 | T21_B13_N/A_N/A | N/A | TB genotype 20 | *Ca. hongkongensis* | PV756177 | PV757743 | N/A | N/A |
| Fujian | Site 1 | 3/1 | 8_Trifolium pratense Seed | (5) | CSFF 27435 | T21_B13_N/A_N/A | N/A | TB genotype 20 | *Ca. hongkongensis* | PV756178 | PV757744 | N/A | N/A |
| Fujian | Site 1 | 3/1 | 9_Lythrum salicaria Seed | (1) | CSFF 27416 | T24_B19_H18_C15 | TBHC genotype 32 | TB genotype 28 | *Ca. kyotensis* | PV756159 | PV757725 | PV757441 | PV757583 |
| Fujian | Site 1 | 3/1 | 9_Lythrum salicaria Seed | (1) | CSFF 27417 | T23_B13_H17_C11 | TBHC genotype 27 | TB genotype 24 | *Ca. hongkongensis* | PV756160 | PV757726 | PV757442 | PV757584 |
| Fujian | Site 1 | 3/1 | 9_Lythrum salicaria Seed | (2) | CSFF 27418 | T25_B16_H18_C15 | TBHC genotype 41 | TB genotype 34 | *Ca. kyotensis* | PV756161 | PV757727 | PV757443 | PV757585 |
| Fujian | Site 1 | 3/1 | 9_Lythrum salicaria Seed | (2) | CSFF 27419 | T21_B13_H17_C11 | TBHC genotype 23 | TB genotype 20 | *Ca. hongkongensis* | PV756162 | PV757728 | PV757444 | PV757586 |
| Fujian | Site 1 | 3/1 | 9_Lythrum salicaria Seed | (3) | CSFF 27420 | T25_B20_H18_C15 | TBHC genotype 43 | TB genotype 35 | *Ca. kyotensis* | PV756163 | PV757729 | PV757445 | PV757587 |
| Fujian | Site 1 | 3/1 | 9_Lythrum salicaria Seed | (3) | CSFF 27421 | T21_B13_N/A_N/A | N/A | TB genotype 20 | *Ca. hongkongensis* | PV756164 | PV757730 | N/A | N/A |
| Fujian | Site 1 | 3/1 | 9_Lythrum salicaria Seed | (4) | CSFF 27422 | T21_B13_N/A_N/A | N/A | TB genotype 20 | *Ca. hongkongensis* | PV756165 | PV757731 | N/A | N/A |
| Fujian | Site 1 | 3/1 | 9_Lythrum salicaria Seed | (4) | CSFF 27423 | T21_B13_N/A_N/A | N/A | TB genotype 20 | *Ca. hongkongensis* | PV756166 | PV757732 | N/A | N/A |
| Fujian | Site 1 | 3/1 | 9_Lythrum salicaria Seed | (5) | CSFF 27424 | T25_B20_N/A_N/A | N/A | TB genotype 35 | *Ca. kyotensis* | PV756167 | PV757733 | N/A | N/A |
| Fujian | Site 1 | 3/1 | 9_Lythrum salicaria Seed | (5) | CSFF 27425 | T24_B16_N/A_N/A | N/A | TB genotype 25 | *Ca. kyotensis* | PV756168 | PV757734 | N/A | N/A |
| Fujian | Site 1 | 3/1 | 11_Imperata cylindrica Leaf | (1) | CSFF 27566 | T24_B20_N/A_N/A | N/A | TB genotype 29 | *Ca. kyotensis* | PV756309 | PV757875 | N/A | N/A |
| Fujian | Site 1 | 3/1 | 11_Imperata cylindrica Leaf | (1) | CSFF 27567 | T24_B20_N/A_N/A | N/A | TB genotype 29 | *Ca. kyotensis* | PV756310 | PV757876 | N/A | N/A |
| Fujian | Site 1 | 3/1 | 11_Imperata cylindrica Leaf | (2) | CSFF 27568 | T25_B20_N/A_N/A | N/A | TB genotype 35 | *Ca. kyotensis* | PV756311 | PV757877 | N/A | N/A |
| Fujian | Site 1 | 3/1 | 11_Imperata cylindrica Leaf | (2) | CSFF 27569 | T24_B22_H19_C13 | TBHC genotype 37 | TB genotype 31 | *Ca. kyotensis* | PV756312 | PV757878 | PV757472 | PV757614 |
| Fujian | Site 1 | 3/1 | 11_Imperata cylindrica Leaf | (3) | CSFF 27570 | T24_B16_N/A_N/A | N/A | TB genotype 25 | *Ca. kyotensis* | PV756313 | PV757879 | N/A | N/A |
| Fujian | Site 1 | 3/1 | 11_Imperata cylindrica Leaf | (3) | CSFF 27571 | T24_B16_N/A_N/A | N/A | TB genotype 25 | *Ca. kyotensis* | PV756314 | PV757880 | N/A | N/A |
| Fujian | Site 1 | 3/1 | 11_Imperata cylindrica Leaf | (4) | CSFF 27572 | T25_B16_N/A_N/A | N/A | TB genotype 34 | *Ca. kyotensis* | PV756315 | PV757881 | N/A | N/A |
| Fujian | Site 1 | 3/1 | 11_Imperata cylindrica Leaf | (4) | CSFF 27573 | T25_B16_N/A_N/A | N/A | TB genotype 34 | *Ca. kyotensis* | PV756316 | PV757882 | N/A | N/A |
| Fujian | Site 1 | 3/1 | 11_Imperata cylindrica Leaf | (5) | CSFF 27574 | T21_B13_N/A_N/A | N/A | TB genotype 20 | *Ca. hongkongensis* | PV756317 | PV757883 | N/A | N/A |
| Fujian | Site 1 | 3/1 | 11_Imperata cylindrica Leaf | (5) | CSFF 27575 | T21_B13_N/A_N/A | N/A | TB genotype 20 | *Ca. hongkongensis* | PV756318 | PV757884 | N/A | N/A |
| Fujian | Site 1 | 3/1 | 12_Pinus massoniana Leaf | (1) | CSFF 27576 | T24_B19_N/A_N/A | N/A | TB genotype 28 | *Ca. kyotensis* | PV756319 | PV757885 | N/A | N/A |
| Fujian | Site 1 | 3/1 | 12_Pinus massoniana Leaf | (1) | CSFF 27577 | T24_B19_N/A_N/A | N/A | TB genotype 28 | *Ca. kyotensis* | PV756320 | PV757886 | N/A | N/A |
| Fujian | Site 1 | 3/1 | 12_Pinus massoniana Leaf | (2) | CSFF 27578 | T24_B19_N/A_N/A | N/A | TB genotype 28 | *Ca. kyotensis* | PV756321 | PV757887 | N/A | N/A |
| Fujian | Site 1 | 3/1 | 12_Pinus massoniana Leaf | (2) | CSFF 27579 | T21_B13_N/A_N/A | N/A | TB genotype 20 | *Ca. hongkongensis* | PV756322 | PV757888 | N/A | N/A |
| Fujian | Site 1 | 3/1 | 12_Pinus massoniana Leaf | (3) | CSFF 27580 | T24_B19_N/A_N/A | N/A | TB genotype 28 | *Ca. kyotensis* | PV756323 | PV757889 | N/A | N/A |
| Fujian | Site 1 | 3/1 | 12_Pinus massoniana Leaf | (3) | CSFF 27581 | T24_B19_N/A_N/A | N/A | TB genotype 28 | *Ca. kyotensis* | PV756324 | PV757890 | N/A | N/A |
| Fujian | Site 1 | 3/1 | 12_Pinus massoniana Leaf | (4) | CSFF 27582 | T24_B16_N/A_N/A | N/A | TB genotype 25 | *Ca. kyotensis* | PV756325 | PV757891 | N/A | N/A |
| Fujian | Site 1 | 3/1 | 12_Pinus massoniana Leaf | (4) | CSFF 27583 | T24_B16_N/A_N/A | N/A | TB genotype 25 | *Ca. kyotensis* | PV756326 | PV757892 | N/A | N/A |
| Fujian | Site 1 | 3/1 | 12_Pinus massoniana Leaf | (5) | CSFF 27584 | T21_B13_N/A_N/A | N/A | TB genotype 20 | *Ca. hongkongensis* | PV756327 | PV757893 | N/A | N/A |
| Fujian | Site 1 | 3/1 | 12_Pinus massoniana Leaf | (5) | CSFF 27585 | T25_B16_H18_C15 | TBHC genotype 41 | TB genotype 34 | *Ca. kyotensis* | PV756328 | PV757894 | PV757473 | PV757615 |
| Fujian | Site 1 | 3/1 | 13_Eucalyptus urophylla hybrid Leaf | (1) | CSFF 27604 | T24_B16_H18_C15 | TBHC genotype 28 | TB genotype 25 | *Ca. kyotensis* | PV756347 | PV757913 | PV757478 | PV757620 |
| Fujian | Site 1 | 3/1 | 13_Eucalyptus urophylla hybrid Leaf | (1) | CSFF 27605 | T24_B16_N/A_N/A | N/A | TB genotype 25 | *Ca. kyotensis* | PV756348 | PV757914 | N/A | N/A |
| Fujian | Site 1 | 3/1 | 13_Eucalyptus urophylla hybrid Leaf | (2) | CSFF 27606 | T24_B16_N/A_N/A | N/A | TB genotype 25 | *Ca. kyotensis* | PV756349 | PV757915 | N/A | N/A |
| Fujian | Site 1 | 3/1 | 13_Eucalyptus urophylla hybrid Leaf | (2) | CSFF 27607 | T25_B20_H18_C15 | TBHC genotype 43 | TB genotype 35 | *Ca. kyotensis* | PV756350 | PV757916 | PV757479 | PV757621 |
| Fujian | Site 1 | 3/1 | 13_Eucalyptus urophylla hybrid Leaf | (3) | CSFF 27608 | T24_B16_N/A_N/A | N/A | TB genotype 25 | *Ca. kyotensis* | PV756351 | PV757917 | N/A | N/A |
| Fujian | Site 1 | 3/1 | 13_Eucalyptus urophylla hybrid Leaf | (3) | CSFF 27609 | T26_B24_H18_C15 | TBHC genotype 45 | TB genotype 37 | *Ca. kyotensis* | PV756352 | PV757918 | PV757480 | PV757622 |
| Fujian | Site 1 | 3/1 | 13_Eucalyptus urophylla hybrid Leaf | (4) | CSFF 27610 | T21_B13_H17_C11 | TBHC genotype 23 | TB genotype 20 | *Ca. hongkongensis* | PV756353 | PV757919 | PV757481 | PV757623 |
| Fujian | Site 1 | 3/1 | 13_Eucalyptus urophylla hybrid Leaf | (4) | CSFF 27611 | T25_B16_H18_C15 | TBHC genotype 41 | TB genotype 34 | *Ca. kyotensis* | PV756354 | PV757920 | PV757482 | PV757624 |
| Fujian | Site 1 | 3/1 | 13_Eucalyptus urophylla hybrid Leaf | (5) | CSFF 27612 | T21_B13_N/A_N/A | N/A | TB genotype 20 | *Ca. hongkongensis* | PV756355 | PV757921 | N/A | N/A |
| Fujian | Site 1 | 3/1 | 13_Eucalyptus urophylla hybrid Leaf | (5) | CSFF 27613 | T27_B20_H18_C15 | TBHC genotype 46 | TB genotype 38 | *Ca. kyotensis* | PV756356 | PV757922 | PV757483 | PV757625 |
| Fujian | Site 1 | 3/1 | 14_Syzygium jambos Leaf | (1) | CSFF 27546 | T21_B13_N/A_N/A | N/A | TB genotype 20 | *Ca. hongkongensis* | PV756289 | PV757855 | N/A | N/A |
| Fujian | Site 1 | 3/1 | 14_Syzygium jambos Leaf | (1) | CSFF 27547 | T24_B16_N/A_N/A | N/A | TB genotype 25 | *Ca. kyotensis* | PV756290 | PV757856 | N/A | N/A |
| Fujian | Site 1 | 3/1 | 14_Syzygium jambos Leaf | (2) | CSFF 27548 | T24_B16_N/A_N/A | N/A | TB genotype 25 | *Ca. kyotensis* | PV756291 | PV757857 | N/A | N/A |
| Fujian | Site 1 | 3/1 | 14_Syzygium jambos Leaf | (2) | CSFF 27549 | T24_B16_N/A_N/A | N/A | TB genotype 25 | *Ca. kyotensis* | PV756292 | PV757858 | N/A | N/A |
| Fujian | Site 1 | 3/1 | 14_Syzygium jambos Leaf | (3) | CSFF 27550 | T24_B19_H18_C15 | TBHC genotype 32 | TB genotype 28 | *Ca. kyotensis* | PV756293 | PV757859 | PV757468 | PV757610 |
| Fujian | Site 1 | 3/1 | 14_Syzygium jambos Leaf | (3) | CSFF 27551 | T21_B13_N/A_N/A | N/A | TB genotype 20 | *Ca. hongkongensis* | PV756294 | PV757860 | N/A | N/A |
| Fujian | Site 1 | 3/1 | 14_Syzygium jambos Leaf | (4) | CSFF 27552 | T24_B19_N/A_N/A | N/A | TB genotype 28 | *Ca. kyotensis* | PV756295 | PV757861 | N/A | N/A |
| Fujian | Site 1 | 3/1 | 14_Syzygium jambos Leaf | (4) | CSFF 27553 | T24_B19_N/A_N/A | N/A | TB genotype 28 | *Ca. kyotensis* | PV756296 | PV757862 | N/A | N/A |
| Fujian | Site 1 | 3/1 | 14_Syzygium jambos Leaf | (5) | CSFF 27554 | T25_B20_N/A_N/A | N/A | TB genotype 35 | *Ca. kyotensis* | PV756297 | PV757863 | N/A | N/A |
| Fujian | Site 1 | 3/1 | 14_Syzygium jambos Leaf | (5) | CSFF 27555 | T24_B22_H19_C13 | TBHC genotype 37 | TB genotype 31 | *Ca. kyotensis* | PV756298 | PV757864 | PV757469 | PV757611 |
| Fujian | Site 1 | 3/1 | 15_Pennisetum purpureum Leaf | (1) | CSFF 27614 | T21_B13_N/A_N/A | N/A | TB genotype 20 | *Ca. hongkongensis* | PV756357 | PV757923 | N/A | N/A |
| Fujian | Site 1 | 3/1 | 15_Pennisetum purpureum Leaf | (1) | CSFF 27615 | T21_B13_N/A_N/A | N/A | TB genotype 20 | *Ca. hongkongensis* | PV756358 | PV757924 | N/A | N/A |
| Fujian | Site 1 | 3/1 | 15_Pennisetum purpureum Leaf | (2) | CSFF 27616 | T25_B22_H18_C13 | TBHC genotype 44 | TB genotype 36 | *Ca. kyotensis* | PV756359 | PV757925 | PV757484 | PV757626 |
| Fujian | Site 1 | 3/1 | 15_Pennisetum purpureum Leaf | (2) | CSFF 27617 | T24_B20_N/A_N/A | N/A | TB genotype 29 | *Ca. kyotensis* | PV756360 | PV757926 | N/A | N/A |
| Fujian | Site 1 | 3/1 | 15_Pennisetum purpureum Leaf | (3) | CSFF 27618 | T21_B13_N/A_N/A | N/A | TB genotype 20 | *Ca. hongkongensis* | PV756361 | PV757927 | N/A | N/A |
| Fujian | Site 1 | 3/1 | 15_Pennisetum purpureum Leaf | (3) | CSFF 27619 | T21_B13_N/A_N/A | N/A | TB genotype 20 | *Ca. hongkongensis* | PV756362 | PV757928 | N/A | N/A |
| Fujian | Site 1 | 3/1 | 15_Pennisetum purpureum Leaf | (4) | CSFF 27620 | T21_B13_N/A_N/A | N/A | TB genotype 20 | *Ca. hongkongensis* | PV756363 | PV757929 | N/A | N/A |
| Fujian | Site 1 | 3/1 | 15_Pennisetum purpureum Leaf | (4) | CSFF 27621 | T21_B14_H17_C11 | TBHC genotype 24 | TB genotype 21 | *Ca. hongkongensis* | PV756364 | PV757930 | PV757485 | PV757627 |
| Fujian | Site 1 | 3/1 | 15_Pennisetum purpureum Leaf | (5) | CSFF 27622 | T21_B13_N/A_N/A | N/A | TB genotype 20 | *Ca. hongkongensis* | PV756365 | PV757931 | N/A | N/A |
| Fujian | Site 1 | 3/1 | 15_Pennisetum purpureum Leaf | (5) | CSFF 27623 | T21_B13_N/A_N/A | N/A | TB genotype 20 | *Ca. hongkongensis* | PV756366 | PV757932 | N/A | N/A |
| Fujian | Site 1 | 3/1 | 16_Saccharum officinarum Leaf | (1) | CSFF 27526 | T24_B16_N/A_N/A | N/A | TB genotype 25 | *Ca. kyotensis* | PV756269 | PV757835 | N/A | N/A |
| Fujian | Site 1 | 3/1 | 16_Saccharum officinarum Leaf | (1) | CSFF 27527 | T24_B19_N/A_N/A | N/A | TB genotype 28 | *Ca. kyotensis* | PV756270 | PV757836 | N/A | N/A |
| Fujian | Site 1 | 3/1 | 16_Saccharum officinarum Leaf | (2) | CSFF 27528 | T24_B21_H20_C13 | TBHC genotype 36 | TB genotype 30 | *Ca. kyotensis* | PV756271 | PV757837 | PV757463 | PV757605 |
| Fujian | Site 1 | 3/1 | 16_Saccharum officinarum Leaf | (2) | CSFF 27529 | T24_B21_N/A_N/A | N/A | TB genotype 30 | *Ca. kyotensis* | PV756272 | PV757838 | N/A | N/A |
| Fujian | Site 1 | 3/1 | 16_Saccharum officinarum Leaf | (3) | CSFF 27530 | T21_B13_N/A_N/A | N/A | TB genotype 20 | *Ca. hongkongensis* | PV756273 | PV757839 | N/A | N/A |
| Fujian | Site 1 | 3/1 | 16_Saccharum officinarum Leaf | (3) | CSFF 27531 | T21_B13_N/A_N/A | N/A | TB genotype 20 | *Ca. hongkongensis* | PV756274 | PV757840 | N/A | N/A |
| Fujian | Site 1 | 3/1 | 16_Saccharum officinarum Leaf | (4) | CSFF 27532 | T25_B16_N/A_N/A | N/A | TB genotype 34 | *Ca. kyotensis* | PV756275 | PV757841 | N/A | N/A |
| Fujian | Site 1 | 3/1 | 16_Saccharum officinarum Leaf | (4) | CSFF 27533 | T24_B16_N/A_N/A | N/A | TB genotype 25 | *Ca. kyotensis* | PV756276 | PV757842 | N/A | N/A |
| Fujian | Site 1 | 3/1 | 16_Saccharum officinarum Leaf | (5) | CSFF 27534 | T21_B13_N/A_N/A | N/A | TB genotype 20 | *Ca. hongkongensis* | PV756277 | PV757843 | N/A | N/A |
| Fujian | Site 1 | 3/1 | 16_Saccharum officinarum Leaf | (5) | CSFF 27535 | T21_B13_N/A_N/A | N/A | TB genotype 20 | *Ca. hongkongensis* | PV756278 | PV757844 | N/A | N/A |
| Fujian | Site 1 | 3/1 | 17_Rosa chinensis Leaf | (1) | CSFF 27496 | T24_B19_N/A_N/A | N/A | TB genotype 28 | *Ca. kyotensis* | PV756239 | PV757805 | N/A | N/A |
| Fujian | Site 1 | 3/1 | 17_Rosa chinensis Leaf | (1) | CSFF 27497 | T21_B13_N/A_N/A | N/A | TB genotype 20 | *Ca. hongkongensis* | PV756240 | PV757806 | N/A | N/A |
| Fujian | Site 1 | 3/1 | 17_Rosa chinensis Leaf | (2) | CSFF 27498 | T25_B16_N/A_N/A | N/A | TB genotype 34 | *Ca. kyotensis* | PV756241 | PV757807 | N/A | N/A |
| Fujian | Site 1 | 3/1 | 17_Rosa chinensis Leaf | (2) | CSFF 27499 | T24_B20_H18_C15 | TBHC genotype 35 | TB genotype 29 | *Ca. kyotensis* | PV756242 | PV757808 | PV757459 | PV757601 |
| Fujian | Site 1 | 3/1 | 17_Rosa chinensis Leaf | (3) | CSFF 27500 | T24_B20_N/A_N/A | N/A | TB genotype 29 | *Ca. kyotensis* | PV756243 | PV757809 | N/A | N/A |
| Fujian | Site 1 | 3/1 | 17_Rosa chinensis Leaf | (3) | CSFF 27501 | T21_B13_N/A_N/A | N/A | TB genotype 20 | *Ca. hongkongensis* | PV756244 | PV757810 | N/A | N/A |
| Fujian | Site 1 | 3/1 | 17_Rosa chinensis Leaf | (4) | CSFF 27502 | T25_B20_N/A_N/A | N/A | TB genotype 35 | *Ca. kyotensis* | PV756245 | PV757811 | N/A | N/A |
| Fujian | Site 1 | 3/1 | 17_Rosa chinensis Leaf | (4) | CSFF 27503 | T25_B16_N/A_N/A | N/A | TB genotype 34 | *Ca. kyotensis* | PV756246 | PV757812 | N/A | N/A |
| Fujian | Site 1 | 3/1 | 17_Rosa chinensis Leaf | (5) | CSFF 27504 | T24_B18_H18_C15 | TBHC genotype 30 | TB genotype 27 | *Ca. kyotensis* | PV756247 | PV757813 | PV757460 | PV757602 |
| Fujian | Site 1 | 3/1 | 17_Rosa chinensis Leaf | (5) | CSFF 27505 | T24_B20_N/A_N/A | N/A | TB genotype 29 | *Ca. kyotensis* | PV756248 | PV757814 | N/A | N/A |
| Fujian | Site 1 | 3/1 | 18_Acacia mangium Leaf | (1) | CSFF 27536 | T24_B19_N/A_N/A | N/A | TB genotype 28 | *Ca. kyotensis* | PV756279 | PV757845 | N/A | N/A |
| Fujian | Site 1 | 3/1 | 18_Acacia mangium Leaf | (1) | CSFF 27537 | T24_B19_N/A_N/A | N/A | TB genotype 28 | *Ca. kyotensis* | PV756280 | PV757846 | N/A | N/A |
| Fujian | Site 1 | 3/1 | 18_Acacia mangium Leaf | (2) | CSFF 27538 | T24_B19_N/A_N/A | N/A | TB genotype 28 | *Ca. kyotensis* | PV756281 | PV757847 | N/A | N/A |
| Fujian | Site 1 | 3/1 | 18_Acacia mangium Leaf | (2) | CSFF 27539 | T24_B16_N/A_N/A | N/A | TB genotype 25 | *Ca. kyotensis* | PV756282 | PV757848 | N/A | N/A |
| Fujian | Site 1 | 3/1 | 18_Acacia mangium Leaf | (3) | CSFF 27540 | T25_B20_N/A_N/A | N/A | TB genotype 35 | *Ca. kyotensis* | PV756283 | PV757849 | N/A | N/A |
| Fujian | Site 1 | 3/1 | 18_Acacia mangium Leaf | (3) | CSFF 27541 | T24_B24_H19_C13 | TBHC genotype 40 | TB genotype 33 | *Ca. kyotensis* | PV756284 | PV757850 | PV757464 | PV757606 |
| Fujian | Site 1 | 3/1 | 18_Acacia mangium Leaf | (4) | CSFF 27542 | T25_B22_H18_C13 | TBHC genotype 44 | TB genotype 36 | *Ca. kyotensis* | PV756285 | PV757851 | PV757465 | PV757607 |
| Fujian | Site 1 | 3/1 | 18_Acacia mangium Leaf | (4) | CSFF 27543 | T25_B22_H18_C13 | TBHC genotype 44 | TB genotype 36 | *Ca. kyotensis* | PV756286 | PV757852 | PV757466 | PV757608 |
| Fujian | Site 1 | 3/1 | 18_Acacia mangium Leaf | (5) | CSFF 27544 | T24_B20_N/A_N/A | N/A | TB genotype 29 | *Ca. kyotensis* | PV756287 | PV757853 | N/A | N/A |
| Fujian | Site 1 | 3/1 | 18_Acacia mangium Leaf | (5) | CSFF 27545 | T21_B14_H17_C11 | TBHC genotype 24 | TB genotype 21 | *Ca. hongkongensis* | PV756288 | PV757854 | PV757467 | PV757609 |
| Fujian | Site 1 | 3/1 | 19_Dianthus caryophyllus Leaf | (1) | CSFF 27486 | T21_B13_N/A_N/A | N/A | TB genotype 20 | *Ca. hongkongensis* | PV756229 | PV757795 | N/A | N/A |
| Fujian | Site 1 | 3/1 | 19_Dianthus caryophyllus Leaf | (1) | CSFF 27487 | T24_B16_N/A_N/A | N/A | TB genotype 25 | *Ca. kyotensis* | PV756230 | PV757796 | N/A | N/A |
| Fujian | Site 1 | 3/1 | 19_Dianthus caryophyllus Leaf | (2) | CSFF 27488 | T21_B13_N/A_N/A | N/A | TB genotype 20 | *Ca. hongkongensis* | PV756231 | PV757797 | N/A | N/A |
| Fujian | Site 1 | 3/1 | 19_Dianthus caryophyllus Leaf | (2) | CSFF 27489 | T25_B16_H18_C15 | TBHC genotype 41 | TB genotype 34 | *Ca. kyotensis* | PV756232 | PV757798 | PV757455 | PV757597 |
| Fujian | Site 1 | 3/1 | 19_Dianthus caryophyllus Leaf | (3) | CSFF 27490 | T21_B13_N/A_N/A | N/A | TB genotype 20 | *Ca. hongkongensis* | PV756233 | PV757799 | N/A | N/A |
| Fujian | Site 1 | 3/1 | 19_Dianthus caryophyllus Leaf | (3) | CSFF 27491 | T21_B13_N/A_N/A | N/A | TB genotype 20 | *Ca. hongkongensis* | PV756234 | PV757800 | N/A | N/A |
| Fujian | Site 1 | 3/1 | 19_Dianthus caryophyllus Leaf | (4) | CSFF 27492 | T24_B24_H18_C15 | TBHC genotype 39 | TB genotype 33 | *Ca. kyotensis* | PV756235 | PV757801 | PV757456 | PV757598 |
| Fujian | Site 1 | 3/1 | 19_Dianthus caryophyllus Leaf | (4) | CSFF 27493 | T27_B20_H18_C15 | TBHC genotype 46 | TB genotype 38 | *Ca. kyotensis* | PV756236 | PV757802 | PV757457 | PV757599 |
| Fujian | Site 1 | 3/1 | 19_Dianthus caryophyllus Leaf | (5) | CSFF 27494 | T25_B16_N/A_N/A | N/A | TB genotype 34 | *Ca. kyotensis* | PV756237 | PV757803 | N/A | N/A |
| Fujian | Site 1 | 3/1 | 19_Dianthus caryophyllus Leaf | (5) | CSFF 27495 | T24_B24_H18_C15 | TBHC genotype 39 | TB genotype 33 | *Ca. kyotensis* | PV756238 | PV757804 | PV757458 | PV757600 |
| Fujian | Site 1 | 3/1 | 20_Cinnamomum camphora Leaf | (1) | CSFF 27506 | T24_B19_N/A_N/A | N/A | TB genotype 28 | *Ca. kyotensis* | PV756249 | PV757815 | N/A | N/A |
| Fujian | Site 1 | 3/1 | 20_Cinnamomum camphora Leaf | (1) | CSFF 27507 | T24_B16_N/A_N/A | N/A | TB genotype 25 | *Ca. kyotensis* | PV756250 | PV757816 | N/A | N/A |
| Fujian | Site 1 | 3/1 | 20_Cinnamomum camphora Leaf | (2) | CSFF 27508 | T24_B16_N/A_N/A | N/A | TB genotype 25 | *Ca. kyotensis* | PV756251 | PV757817 | N/A | N/A |
| Fujian | Site 1 | 3/1 | 20_Cinnamomum camphora Leaf | (2) | CSFF 27509 | T25_B16_N/A_N/A | N/A | TB genotype 34 | *Ca. kyotensis* | PV756252 | PV757818 | N/A | N/A |
| Fujian | Site 1 | 3/1 | 20_Cinnamomum camphora Leaf | (3) | CSFF 27510 | T21_B13_N/A_N/A | N/A | TB genotype 20 | *Ca. hongkongensis* | PV756253 | PV757819 | N/A | N/A |
| Fujian | Site 1 | 3/1 | 20_Cinnamomum camphora Leaf | (3) | CSFF 27511 | T21_B13_N/A_N/A | N/A | TB genotype 20 | *Ca. hongkongensis* | PV756254 | PV757820 | N/A | N/A |
| Fujian | Site 1 | 3/1 | 20_Cinnamomum camphora Leaf | (4) | CSFF 27512 | T24_B19_N/A_N/A | N/A | TB genotype 28 | *Ca. kyotensis* | PV756255 | PV757821 | N/A | N/A |
| Fujian | Site 1 | 3/1 | 20_Cinnamomum camphora Leaf | (4) | CSFF 27513 | T25_B20_N/A_N/A | N/A | TB genotype 35 | *Ca. kyotensis* | PV756256 | PV757822 | N/A | N/A |
| Fujian | Site 1 | 3/1 | 20_Cinnamomum camphora Leaf | (5) | CSFF 27514 | T21_B13_N/A_N/A | N/A | TB genotype 20 | *Ca. hongkongensis* | PV756257 | PV757823 | N/A | N/A |
| Fujian | Site 1 | 3/1 | 20_Cinnamomum camphora Leaf | (5) | CSFF 27515 | T24_B16_N/A_N/A | N/A | TB genotype 25 | *Ca. kyotensis* | PV756258 | PV757824 | N/A | N/A |
| Fujian | Site 1 | 3/1 | 21_Washingtonia filifera Leaf | (1) | CSFF 27516 | T24_B19_N/A_N/A | N/A | TB genotype 28 | *Ca. kyotensis* | PV756259 | PV757825 | N/A | N/A |
| Fujian | Site 1 | 3/1 | 21_Washingtonia filifera Leaf | (1) | CSFF 27517 | T24_B19_N/A_N/A | N/A | TB genotype 28 | *Ca. kyotensis* | PV756260 | PV757826 | N/A | N/A |
| Fujian | Site 1 | 3/1 | 21_Washingtonia filifera Leaf | (2) | CSFF 27518 | T24_B19_N/A_N/A | N/A | TB genotype 28 | *Ca. kyotensis* | PV756261 | PV757827 | N/A | N/A |
| Fujian | Site 1 | 3/1 | 21_Washingtonia filifera Leaf | (2) | CSFF 27519 | T24_B21_H20_C13 | TBHC genotype 36 | TB genotype 30 | *Ca. kyotensis* | PV756262 | PV757828 | PV757461 | PV757603 |
| Fujian | Site 1 | 3/1 | 21_Washingtonia filifera Leaf | (3) | CSFF 27520 | T24_B21_N/A_N/A | N/A | TB genotype 30 | *Ca. kyotensis* | PV756263 | PV757829 | N/A | N/A |
| Fujian | Site 1 | 3/1 | 21_Washingtonia filifera Leaf | (3) | CSFF 27521 | T24_B22_H19_C13 | TBHC genotype 37 | TB genotype 31 | *Ca. kyotensis* | PV756264 | PV757830 | PV757462 | PV757604 |
| Fujian | Site 1 | 3/1 | 21_Washingtonia filifera Leaf | (4) | CSFF 27522 | T24_B21_N/A_N/A | N/A | TB genotype 30 | *Ca. kyotensis* | PV756265 | PV757831 | N/A | N/A |
| Fujian | Site 1 | 3/1 | 21_Washingtonia filifera Leaf | (4) | CSFF 27523 | T24_B21_N/A_N/A | N/A | TB genotype 30 | *Ca. kyotensis* | PV756266 | PV757832 | N/A | N/A |
| Fujian | Site 1 | 3/1 | 21_Washingtonia filifera Leaf | (5) | CSFF 27524 | T24_B21_N/A_N/A | N/A | TB genotype 30 | *Ca. kyotensis* | PV756267 | PV757833 | N/A | N/A |
| Fujian | Site 1 | 3/1 | 21_Washingtonia filifera Leaf | (5) | CSFF 27525 | T21_B13_N/A_N/A | N/A | TB genotype 20 | *Ca. hongkongensis* | PV756268 | PV757834 | N/A | N/A |
| Fujian | Site 1 | 3/1 | 22_Ficus benjamina Leaf | (1) | CSFF 27556 | T21_B13_N/A_N/A | N/A | TB genotype 20 | *Ca. hongkongensis* | PV756299 | PV757865 | N/A | N/A |
| Fujian | Site 1 | 3/1 | 22_Ficus benjamina Leaf | (1) | CSFF 27557 | T21_B13_N/A_N/A | N/A | TB genotype 20 | *Ca. hongkongensis* | PV756300 | PV757866 | N/A | N/A |
| Fujian | Site 1 | 3/1 | 22_Ficus benjamina Leaf | (2) | CSFF 27558 | T21_B13_N/A_N/A | N/A | TB genotype 20 | *Ca. hongkongensis* | PV756301 | PV757867 | N/A | N/A |
| Fujian | Site 1 | 3/1 | 22_Ficus benjamina Leaf | (2) | CSFF 27559 | T21_B13_N/A_N/A | N/A | TB genotype 20 | *Ca. hongkongensis* | PV756302 | PV757868 | N/A | N/A |
| Fujian | Site 1 | 3/1 | 22_Ficus benjamina Leaf | (3) | CSFF 27560 | T25_B22_H18_C13 | TBHC genotype 44 | TB genotype 36 | *Ca. kyotensis* | PV756303 | PV757869 | PV757470 | PV757612 |
| Fujian | Site 1 | 3/1 | 22_Ficus benjamina Leaf | (3) | CSFF 27561 | T25_B22_N/A_N/A | N/A | TB genotype 36 | *Ca. kyotensis* | PV756304 | PV757870 | N/A | N/A |
| Fujian | Site 1 | 3/1 | 22_Ficus benjamina Leaf | (4) | CSFF 27562 | T24_B16_N/A_N/A | N/A | TB genotype 25 | *Ca. kyotensis* | PV756305 | PV757871 | N/A | N/A |
| Fujian | Site 1 | 3/1 | 22_Ficus benjamina Leaf | (4) | CSFF 27563 | T24_B16_N/A_N/A | N/A | TB genotype 25 | *Ca. kyotensis* | PV756306 | PV757872 | N/A | N/A |
| Fujian | Site 1 | 3/1 | 22_Ficus benjamina Leaf | (5) | CSFF 27564 | T24_B16_N/A_N/A | N/A | TB genotype 25 | *Ca. kyotensis* | PV756307 | PV757873 | N/A | N/A |
| Fujian | Site 1 | 3/1 | 22_Ficus benjamina Leaf | (5) | CSFF 27565 | T24_B22_H19_C13 | TBHC genotype 37 | TB genotype 31 | *Ca. kyotensis* | PV756308 | PV757874 | PV757471 | PV757613 |
| Fujian | Site 1 | 3/1 | 23_Rhododendron pulchrum Leaf | (1) | CSFF 27586 | T25_B20_H18_C15 | TBHC genotype 43 | TB genotype 35 | *Ca. kyotensis* | PV756329 | PV757895 | PV757474 | PV757616 |
| Fujian | Site 1 | 3/1 | 23_Rhododendron pulchrum Leaf | (1) | CSFF 27587 | T25_B20_N/A_N/A | N/A | TB genotype 35 | *Ca. kyotensis* | PV756330 | PV757896 | N/A | N/A |
| Fujian | Site 1 | 3/1 | 23_Rhododendron pulchrum Leaf | (2) | CSFF 27588 | T24_B16_N/A_N/A | N/A | TB genotype 25 | *Ca. kyotensis* | PV756331 | PV757897 | N/A | N/A |
| Fujian | Site 1 | 3/1 | 23_Rhododendron pulchrum Leaf | (2) | CSFF 27589 | T24_B16_N/A_N/A | N/A | TB genotype 25 | *Ca. kyotensis* | PV756332 | PV757898 | N/A | N/A |
| Fujian | Site 1 | 3/1 | 23_Rhododendron pulchrum Leaf | (3) | CSFF 27590 | T24_B20_H18_C15 | TBHC genotype 35 | TB genotype 29 | *Ca. kyotensis* | PV756333 | PV757899 | PV757475 | PV757617 |
| Fujian | Site 1 | 3/1 | 23_Rhododendron pulchrum Leaf | (3) | CSFF 27591 | T24_B20_N/A_N/A | N/A | TB genotype 29 | *Ca. kyotensis* | PV756334 | PV757900 | N/A | N/A |
| Fujian | Site 1 | 3/1 | 23_Rhododendron pulchrum Leaf | (4) | CSFF 27592 | T24_B16_N/A_N/A | N/A | TB genotype 25 | *Ca. kyotensis* | PV756335 | PV757901 | N/A | N/A |
| Fujian | Site 1 | 3/1 | 23_Rhododendron pulchrum Leaf | (4) | CSFF 27593 | T21_B13_N/A_N/A | N/A | TB genotype 20 | *Ca. hongkongensis* | PV756336 | PV757902 | N/A | N/A |
| Fujian | Site 1 | 3/1 | 23_Rhododendron pulchrum Leaf | (5) | CSFF 27594 | T21_B13_N/A_N/A | N/A | TB genotype 20 | *Ca. hongkongensis* | PV756337 | PV757903 | N/A | N/A |
| Fujian | Site 1 | 3/1 | 23_Rhododendron pulchrum Leaf | (5) | CSFF 27595 | T21_B13_N/A_N/A | N/A | TB genotype 20 | *Ca. hongkongensis* | PV756338 | PV757904 | N/A | N/A |
| Fujian | Site 1 | 3/1 | 24_Glycine max Stem | (1) | CSFF 27596 | T24_B21_H20_C13 | TBHC genotype 36 | TB genotype 30 | *Ca. kyotensis* | PV756339 | PV757905 | PV757476 | PV757618 |
| Fujian | Site 1 | 3/1 | 24_Glycine max Stem | (1) | CSFF 27597 | T24_B16_N/A_N/A | N/A | TB genotype 25 | *Ca. kyotensis* | PV756340 | PV757906 | N/A | N/A |
| Fujian | Site 1 | 3/1 | 24_Glycine max Stem | (2) | CSFF 27598 | T21_B14_H17_C11 | TBHC genotype 24 | TB genotype 21 | *Ca. hongkongensis* | PV756341 | PV757907 | PV757477 | PV757619 |
| Fujian | Site 1 | 3/1 | 24_Glycine max Stem | (2) | CSFF 27599 | T25_B20_N/A_N/A | N/A | TB genotype 35 | *Ca. kyotensis* | PV756342 | PV757908 | N/A | N/A |
| Fujian | Site 1 | 3/1 | 24_Glycine max Stem | (3) | CSFF 27600 | T24_B16_N/A_N/A | N/A | TB genotype 25 | *Ca. kyotensis* | PV756343 | PV757909 | N/A | N/A |
| Fujian | Site 1 | 3/1 | 24_Glycine max Stem | (3) | CSFF 27601 | T21_B13_N/A_N/A | N/A | TB genotype 20 | *Ca. hongkongensis* | PV756344 | PV757910 | N/A | N/A |
| Fujian | Site 1 | 3/1 | 24_Glycine max Stem | (4) | CSFF 27602 | T24_B20_N/A_N/A | N/A | TB genotype 29 | *Ca. kyotensis* | PV756345 | PV757911 | N/A | N/A |
| Fujian | Site 1 | 3/1 | 24_Glycine max Stem | (4) | CSFF 27603 | T24_B20_N/A_N/A | N/A | TB genotype 29 | *Ca. kyotensis* | PV756346 | PV757912 | N/A | N/A |
| Fujian | Site 1 | 3/1 | 26_Medicago sativa Seed | (1) | CSFF 27624 | T24_B16_N/A_N/A | N/A | TB genotype 25 | *Ca. kyotensis* | PV756367 | PV757933 | N/A | N/A |
| Fujian | Site 1 | 3/1 | 26_Medicago sativa Seed | (1) | CSFF 27625 | T24_B16_N/A_N/A | N/A | TB genotype 25 | *Ca. kyotensis* | PV756368 | PV757934 | N/A | N/A |
| Fujian | Site 1 | 3/1 | 26_Medicago sativa Seed | (2) | CSFF 27626 | T24_B22_N/A_N/A | N/A | TB genotype 31 | *Ca. kyotensis* | PV756369 | PV757935 | N/A | N/A |
| Fujian | Site 1 | 3/1 | 26_Medicago sativa Seed | (2) | CSFF 27627 | T21_B13_N/A_N/A | N/A | TB genotype 20 | *Ca. hongkongensis* | PV756370 | PV757936 | N/A | N/A |
| Fujian | Site 1 | 3/1 | 26_Medicago sativa Seed | (3) | CSFF 27628 | T21_B13_N/A_N/A | N/A | TB genotype 20 | *Ca. hongkongensis* | PV756371 | PV757937 | N/A | N/A |
| Fujian | Site 1 | 3/1 | 26_Medicago sativa Seed | (3) | CSFF 27629 | T21_B13_N/A_N/A | N/A | TB genotype 20 | *Ca. hongkongensis* | PV756372 | PV757938 | N/A | N/A |
| Fujian | Site 1 | 3/1 | 26_Medicago sativa Seed | (4) | CSFF 27630 | T24_B16_N/A_N/A | N/A | TB genotype 25 | *Ca. kyotensis* | PV756373 | PV757939 | N/A | N/A |
| Fujian | Site 1 | 3/1 | 26_Medicago sativa Seed | (4) | CSFF 27631 | T21_B13_N/A_N/A | N/A | TB genotype 20 | *Ca. hongkongensis* | PV756374 | PV757940 | N/A | N/A |
| Fujian | Site 1 | 3/1 | 26_Medicago sativa Seed | (5) | CSFF 27632 | T21_B13_N/A_N/A | N/A | TB genotype 20 | *Ca. hongkongensis* | PV756375 | PV757941 | N/A | N/A |
| Fujian | Site 1 | 3/1 | 26_Medicago sativa Seed | (5) | CSFF 27633 | T24_B16_N/A_N/A | N/A | TB genotype 25 | *Ca. kyotensis* | PV756376 | PV757942 | N/A | N/A |
| Fujian | Site 2 | 3/2 | 2_Zinnia elegans Seed | (3) | CSFF 27670 | T14_B33_N/A_N/A | N/A | TB genotype 7 | *Ca. pacifica* | PV756413 | PV757979 | N/A | N/A |
| Fujian | Site 2 | 3/2 | 2_Zinnia elegans Seed | (3) | CSFF 27671 | T14_B33_N/A_N/A | N/A | TB genotype 7 | *Ca. pacifica* | PV756414 | PV757980 | N/A | N/A |
| Fujian | Site 2 | 3/2 | 2_Zinnia elegans Seed | (4) | CSFF 27672 | T14_B33_N/A_N/A | N/A | TB genotype 7 | *Ca. pacifica* | PV756415 | PV757981 | N/A | N/A |
| Fujian | Site 2 | 3/2 | 2_Zinnia elegans Seed | (4) | CSFF 27673 | T14_B33_N/A_N/A | N/A | TB genotype 7 | *Ca. pacifica* | PV756416 | PV757982 | N/A | N/A |
| Fujian | Site 2 | 3/2 | 2_Zinnia elegans Seed | (5) | CSFF 27674 | T14_B33_N/A_N/A | N/A | TB genotype 7 | *Ca. pacifica* | PV756417 | PV757983 | N/A | N/A |
| Fujian | Site 2 | 3/2 | 2_Zinnia elegans Seed | (5) | CSFF 27675 | T14_B33_N/A_N/A | N/A | TB genotype 7 | *Ca. pacifica* | PV756418 | PV757984 | N/A | N/A |
| Fujian | Site 2 | 3/2 | 3_Lolium perenne Seed | (1) | CSFF 27684 | T14_B33_N/A_N/A | N/A | TB genotype 7 | *Ca. pacifica* | PV756427 | PV757993 | N/A | N/A |
| Fujian | Site 2 | 3/2 | 3_Lolium perenne Seed | (1) | CSFF 27685 | T14_B33_N/A_N/A | N/A | TB genotype 7 | *Ca. pacifica* | PV756428 | PV757994 | N/A | N/A |
| Fujian | Site 2 | 3/2 | 3_Lolium perenne Seed | (5) | CSFF 27686 | T14_B33_N/A_N/A | N/A | TB genotype 7 | *Ca. pacifica* | PV756429 | PV757995 | N/A | N/A |
| Fujian | Site 2 | 3/2 | 3_Lolium perenne Seed | (5) | CSFF 27687 | T14_B33_N/A_N/A | N/A | TB genotype 7 | *Ca. pacifica* | PV756430 | PV757996 | N/A | N/A |
| Fujian | Site 2 | 3/2 | 4_Salvia hispanica Seed | (3) | CSFF 27676 | T14_B33_N/A_N/A | N/A | TB genotype 7 | *Ca. pacifica* | PV756419 | PV757985 | N/A | N/A |
| Fujian | Site 2 | 3/2 | 4_Salvia hispanica Seed | (3) | CSFF 27677 | T14_B33_N/A_N/A | N/A | TB genotype 7 | *Ca. pacifica* | PV756420 | PV757986 | N/A | N/A |
| Fujian | Site 2 | 3/2 | 4_Salvia hispanica Seed | (5) | CSFF 27678 | T14_B33_N/A_N/A | N/A | TB genotype 7 | *Ca. pacifica* | PV756421 | PV757987 | N/A | N/A |
| Fujian | Site 2 | 3/2 | 4_Salvia hispanica Seed | (5) | CSFF 27679 | T14_B33_N/A_N/A | N/A | TB genotype 7 | *Ca. pacifica* | PV756422 | PV757988 | N/A | N/A |
| Fujian | Site 2 | 3/2 | 5_Impatiens balsamina Seed | (1) | CSFF 27680 | T14_B33_N/A_N/A | N/A | TB genotype 7 | *Ca. pacifica* | PV756423 | PV757989 | N/A | N/A |
| Fujian | Site 2 | 3/2 | 5_Impatiens balsamina Seed | (1) | CSFF 27681 | T14_B33_N/A_N/A | N/A | TB genotype 7 | *Ca. pacifica* | PV756424 | PV757990 | N/A | N/A |
| Fujian | Site 2 | 3/2 | 5_Impatiens balsamina Seed | (3) | CSFF 27682 | T14_B33_N/A_N/A | N/A | TB genotype 7 | *Ca. pacifica* | PV756425 | PV757991 | N/A | N/A |
| Fujian | Site 2 | 3/2 | 5_Impatiens balsamina Seed | (3) | CSFF 27683 | T14_B33_N/A_N/A | N/A | TB genotype 7 | *Ca. pacifica* | PV756426 | PV757992 | N/A | N/A |
| Fujian | Site 2 | 3/2 | 6_Sesamum indicum Seed | (1) | CSFF 27654 | T14_B33_N/A_N/A | N/A | TB genotype 7 | *Ca. pacifica* | PV756397 | PV757963 | N/A | N/A |
| Fujian | Site 2 | 3/2 | 6_Sesamum indicum Seed | (1) | CSFF 27655 | T14_B33_N/A_N/A | N/A | TB genotype 7 | *Ca. pacifica* | PV756398 | PV757964 | N/A | N/A |
| Fujian | Site 2 | 3/2 | 6_Sesamum indicum Seed | (2) | CSFF 27656 | T14_B33_N/A_N/A | N/A | TB genotype 7 | *Ca. pacifica* | PV756399 | PV757965 | N/A | N/A |
| Fujian | Site 2 | 3/2 | 6_Sesamum indicum Seed | (2) | CSFF 27657 | T14_B33_N/A_N/A | N/A | TB genotype 7 | *Ca. pacifica* | PV756400 | PV757966 | N/A | N/A |
| Fujian | Site 2 | 3/2 | 6_Sesamum indicum Seed | (3) | CSFF 27658 | T14_B33_N/A_N/A | N/A | TB genotype 7 | *Ca. pacifica* | PV756401 | PV757967 | N/A | N/A |
| Fujian | Site 2 | 3/2 | 6_Sesamum indicum Seed | (3) | CSFF 27659 | T14_B33_N/A_N/A | N/A | TB genotype 7 | *Ca. pacifica* | PV756402 | PV757968 | N/A | N/A |
| Fujian | Site 2 | 3/2 | 6_Sesamum indicum Seed | (4) | CSFF 27660 | T14_B33_N/A_N/A | N/A | TB genotype 7 | *Ca. pacifica* | PV756403 | PV757969 | N/A | N/A |
| Fujian | Site 2 | 3/2 | 6_Sesamum indicum Seed | (4) | CSFF 27661 | T14_B33_N/A_N/A | N/A | TB genotype 7 | *Ca. pacifica* | PV756404 | PV757970 | N/A | N/A |
| Fujian | Site 2 | 3/2 | 7_Dianthus caryophyllus Seed | (1) | CSFF 27662 | T14_B33_N/A_N/A | N/A | TB genotype 7 | *Ca. pacifica* | PV756405 | PV757971 | N/A | N/A |
| Fujian | Site 2 | 3/2 | 7_Dianthus caryophyllus Seed | (1) | CSFF 27663 | T14_B33_N/A_N/A | N/A | TB genotype 7 | *Ca. pacifica* | PV756406 | PV757972 | N/A | N/A |
| Fujian | Site 2 | 3/2 | 7_Dianthus caryophyllus Seed | (2) | CSFF 27664 | T14_B33_N/A_N/A | N/A | TB genotype 7 | *Ca. pacifica* | PV756407 | PV757973 | N/A | N/A |
| Fujian | Site 2 | 3/2 | 7_Dianthus caryophyllus Seed | (2) | CSFF 27665 | T14_B33_N/A_N/A | N/A | TB genotype 7 | *Ca. pacifica* | PV756408 | PV757974 | N/A | N/A |
| Fujian | Site 2 | 3/2 | 7_Dianthus caryophyllus Seed | (3) | CSFF 27666 | T14_B33_N/A_N/A | N/A | TB genotype 7 | *Ca. pacifica* | PV756409 | PV757975 | N/A | N/A |
| Fujian | Site 2 | 3/2 | 7_Dianthus caryophyllus Seed | (3) | CSFF 27667 | T14_B33_N/A_N/A | N/A | TB genotype 7 | *Ca. pacifica* | PV756410 | PV757976 | N/A | N/A |
| Fujian | Site 2 | 3/2 | 7_Dianthus caryophyllus Seed | (4) | CSFF 27668 | T14_B33_N/A_N/A | N/A | TB genotype 7 | *Ca. pacifica* | PV756411 | PV757977 | N/A | N/A |
| Fujian | Site 2 | 3/2 | 7_Dianthus caryophyllus Seed | (4) | CSFF 27669 | T14_B33_N/A_N/A | N/A | TB genotype 7 | *Ca. pacifica* | PV756412 | PV757978 | N/A | N/A |
| Fujian | Site 2 | 3/2 | 8_Trifolium pratense Seed | (1) | CSFF 27644 | T14_B33_N/A_N/A | N/A | TB genotype 7 | *Ca. pacifica* | PV756387 | PV757953 | N/A | N/A |
| Fujian | Site 2 | 3/2 | 8_Trifolium pratense Seed | (1) | CSFF 27645 | T14_B33_N/A_N/A | N/A | TB genotype 7 | *Ca. pacifica* | PV756388 | PV757954 | N/A | N/A |
| Fujian | Site 2 | 3/2 | 8_Trifolium pratense Seed | (2) | CSFF 27646 | T14_B33_N/A_N/A | N/A | TB genotype 7 | *Ca. pacifica* | PV756389 | PV757955 | N/A | N/A |
| Fujian | Site 2 | 3/2 | 8_Trifolium pratense Seed | (2) | CSFF 27647 | T14_B33_N/A_N/A | N/A | TB genotype 7 | *Ca. pacifica* | PV756390 | PV757956 | N/A | N/A |
| Fujian | Site 2 | 3/2 | 8_Trifolium pratense Seed | (3) | CSFF 27648 | T14_B33_N/A_N/A | N/A | TB genotype 7 | *Ca. pacifica* | PV756391 | PV757957 | N/A | N/A |
| Fujian | Site 2 | 3/2 | 8_Trifolium pratense Seed | (3) | CSFF 27649 | T14_B33_N/A_N/A | N/A | TB genotype 7 | *Ca. pacifica* | PV756392 | PV757958 | N/A | N/A |
| Fujian | Site 2 | 3/2 | 8_Trifolium pratense Seed | (4) | CSFF 27650 | T14_B33_N/A_N/A | N/A | TB genotype 7 | *Ca. pacifica* | PV756393 | PV757959 | N/A | N/A |
| Fujian | Site 2 | 3/2 | 8_Trifolium pratense Seed | (4) | CSFF 27651 | T14_B33_N/A_N/A | N/A | TB genotype 7 | *Ca. pacifica* | PV756394 | PV757960 | N/A | N/A |
| Fujian | Site 2 | 3/2 | 8_Trifolium pratense Seed | (5) | CSFF 27652 | T24_B19_H18_C15 | TBHC genotype 32 | TB genotype 28 | *Ca. kyotensis* | PV756395 | PV757961 | PV757487 | PV757629 |
| Fujian | Site 2 | 3/2 | 8_Trifolium pratense Seed | (5) | CSFF 27653 | T14_B33_N/A_N/A | N/A | TB genotype 7 | *Ca. pacifica* | PV756396 | PV757962 | N/A | N/A |
| Fujian | Site 2 | 3/2 | 9_Lythrum salicaria Seed | (1) | CSFF 27634 | T14_B33_H12_C21 | TBHC genotype 7 | TB genotype 7 | *Ca. pacifica* | PV756377 | PV757943 | PV757486 | PV757628 |
| Fujian | Site 2 | 3/2 | 9_Lythrum salicaria Seed | (1) | CSFF 27635 | T14_B33_N/A_N/A | N/A | TB genotype 7 | *Ca. pacifica* | PV756378 | PV757944 | N/A | N/A |
| Fujian | Site 2 | 3/2 | 9_Lythrum salicaria Seed | (2) | CSFF 27636 | T14_B33_N/A_N/A | N/A | TB genotype 7 | *Ca. pacifica* | PV756379 | PV757945 | N/A | N/A |
| Fujian | Site 2 | 3/2 | 9_Lythrum salicaria Seed | (2) | CSFF 27637 | T14_B33_N/A_N/A | N/A | TB genotype 7 | *Ca. pacifica* | PV756380 | PV757946 | N/A | N/A |
| Fujian | Site 2 | 3/2 | 9_Lythrum salicaria Seed | (3) | CSFF 27638 | T14_B33_N/A_N/A | N/A | TB genotype 7 | *Ca. pacifica* | PV756381 | PV757947 | N/A | N/A |
| Fujian | Site 2 | 3/2 | 9_Lythrum salicaria Seed | (3) | CSFF 27639 | T14_B33_N/A_N/A | N/A | TB genotype 7 | *Ca. pacifica* | PV756382 | PV757948 | N/A | N/A |
| Fujian | Site 2 | 3/2 | 9_Lythrum salicaria Seed | (4) | CSFF 27640 | T14_B33_N/A_N/A | N/A | TB genotype 7 | *Ca. pacifica* | PV756383 | PV757949 | N/A | N/A |
| Fujian | Site 2 | 3/2 | 9_Lythrum salicaria Seed | (4) | CSFF 27641 | T14_B33_N/A_N/A | N/A | TB genotype 7 | *Ca. pacifica* | PV756384 | PV757950 | N/A | N/A |
| Fujian | Site 2 | 3/2 | 9_Lythrum salicaria Seed | (5) | CSFF 27642 | T14_B33_N/A_N/A | N/A | TB genotype 7 | *Ca. pacifica* | PV756385 | PV757951 | N/A | N/A |
| Fujian | Site 2 | 3/2 | 9_Lythrum salicaria Seed | (5) | CSFF 27643 | T14_B33_N/A_N/A | N/A | TB genotype 7 | *Ca. pacifica* | PV756386 | PV757952 | N/A | N/A |
| Fujian | Site 2 | 3/2 | 10_Buxus sinica Leaf | (3) | CSFF 27792 | T14_B33_N/A_N/A | N/A | TB genotype 7 | *Ca. pacifica* | PV756535 | PV758101 | N/A | N/A |
| Fujian | Site 2 | 3/2 | 10_Buxus sinica Leaf | (3) | CSFF 27793 | T14_B33_N/A_N/A | N/A | TB genotype 7 | *Ca. pacifica* | PV756536 | PV758102 | N/A | N/A |
| Fujian | Site 2 | 3/2 | 10_Buxus sinica Leaf | (4) | CSFF 27794 | T24_B16_H18_C15 | TBHC genotype 28 | TB genotype 25 | *Ca. kyotensis* | PV756537 | PV758103 | PV757494 | PV757636 |
| Fujian | Site 2 | 3/2 | 10_Buxus sinica Leaf | (4) | CSFF 27795 | T14_B33_N/A_N/A | N/A | TB genotype 7 | *Ca. pacifica* | PV756538 | PV758104 | N/A | N/A |
| Fujian | Site 2 | 3/2 | 10_Buxus sinica Leaf | (5) | CSFF 27796 | T14_B33_N/A_N/A | N/A | TB genotype 7 | *Ca. pacifica* | PV756539 | PV758105 | N/A | N/A |
| Fujian | Site 2 | 3/2 | 10_Buxus sinica Leaf | (5) | CSFF 27797 | T14_B33_N/A_N/A | N/A | TB genotype 7 | *Ca. pacifica* | PV756540 | PV758106 | N/A | N/A |
| Fujian | Site 2 | 3/2 | 11_Imperata cylindrica Leaf | (3) | CSFF 27768 | T24_B23_H18_C14 | TBHC genotype 38 | TB genotype 32 | *Ca. kyotensis* | PV756511 | PV758077 | PV757492 | PV757634 |
| Fujian | Site 2 | 3/2 | 11_Imperata cylindrica Leaf | (3) | CSFF 27769 | T24_B23_H18_C14 | TBHC genotype 38 | TB genotype 32 | *Ca. kyotensis* | PV756512 | PV758078 | PV757493 | PV757635 |
| Fujian | Site 2 | 3/2 | 11_Imperata cylindrica Leaf | (4) | CSFF 27770 | T14_B33_N/A_N/A | N/A | TB genotype 7 | *Ca. pacifica* | PV756513 | PV758079 | N/A | N/A |
| Fujian | Site 2 | 3/2 | 11_Imperata cylindrica Leaf | (4) | CSFF 27771 | T14_B33_N/A_N/A | N/A | TB genotype 7 | *Ca. pacifica* | PV756514 | PV758080 | N/A | N/A |
| Fujian | Site 2 | 3/2 | 12_Pinus massoniana Leaf | (1) | CSFF 27772 | T14_B33_N/A_N/A | N/A | TB genotype 7 | *Ca. pacifica* | PV756515 | PV758081 | N/A | N/A |
| Fujian | Site 2 | 3/2 | 12_Pinus massoniana Leaf | (1) | CSFF 27773 | T14_B33_N/A_N/A | N/A | TB genotype 7 | *Ca. pacifica* | PV756516 | PV758082 | N/A | N/A |
| Fujian | Site 2 | 3/2 | 12_Pinus massoniana Leaf | (2) | CSFF 27774 | T14_B33_N/A_N/A | N/A | TB genotype 7 | *Ca. pacifica* | PV756517 | PV758083 | N/A | N/A |
| Fujian | Site 2 | 3/2 | 12_Pinus massoniana Leaf | (2) | CSFF 27775 | T14_B33_N/A_N/A | N/A | TB genotype 7 | *Ca. pacifica* | PV756518 | PV758084 | N/A | N/A |
| Fujian | Site 2 | 3/2 | 12_Pinus massoniana Leaf | (3) | CSFF 27776 | T14_B33_N/A_N/A | N/A | TB genotype 7 | *Ca. pacifica* | PV756519 | PV758085 | N/A | N/A |
| Fujian | Site 2 | 3/2 | 12_Pinus massoniana Leaf | (3) | CSFF 27777 | T14_B33_N/A_N/A | N/A | TB genotype 7 | *Ca. pacifica* | PV756520 | PV758086 | N/A | N/A |
| Fujian | Site 2 | 3/2 | 12_Pinus massoniana Leaf | (4) | CSFF 27778 | T14_B33_N/A_N/A | N/A | TB genotype 7 | *Ca. pacifica* | PV756521 | PV758087 | N/A | N/A |
| Fujian | Site 2 | 3/2 | 12_Pinus massoniana Leaf | (4) | CSFF 27779 | T14_B33_N/A_N/A | N/A | TB genotype 7 | *Ca. pacifica* | PV756522 | PV758088 | N/A | N/A |
| Fujian | Site 2 | 3/2 | 12_Pinus massoniana Leaf | (5) | CSFF 27780 | T14_B33_N/A_N/A | N/A | TB genotype 7 | *Ca. pacifica* | PV756523 | PV758089 | N/A | N/A |
| Fujian | Site 2 | 3/2 | 12_Pinus massoniana Leaf | (5) | CSFF 27781 | T14_B33_N/A_N/A | N/A | TB genotype 7 | *Ca. pacifica* | PV756524 | PV758090 | N/A | N/A |
| Fujian | Site 2 | 3/2 | 13_Eucalyptus urophylla hybrid Leaf | (1) | CSFF 27802 | T14_B33_H12_C21 | TBHC genotype 7 | TB genotype 7 | *Ca. pacifica* | PV756545 | PV758111 | PV757496 | PV757638 |
| Fujian | Site 2 | 3/2 | 13_Eucalyptus urophylla hybrid Leaf | (1) | CSFF 27803 | T14_B33_N/A_N/A | N/A | TB genotype 7 | *Ca. pacifica* | PV756546 | PV758112 | N/A | N/A |
| Fujian | Site 2 | 3/2 | 13_Eucalyptus urophylla hybrid Leaf | (2) | CSFF 27804 | T14_B33_N/A_N/A | N/A | TB genotype 7 | *Ca. pacifica* | PV756547 | PV758113 | N/A | N/A |
| Fujian | Site 2 | 3/2 | 13_Eucalyptus urophylla hybrid Leaf | (2) | CSFF 27805 | T14_B33_N/A_N/A | N/A | TB genotype 7 | *Ca. pacifica* | PV756548 | PV758114 | N/A | N/A |
| Fujian | Site 2 | 3/2 | 13_Eucalyptus urophylla hybrid Leaf | (5) | CSFF 27806 | T14_B33_N/A_N/A | N/A | TB genotype 7 | *Ca. pacifica* | PV756549 | PV758115 | N/A | N/A |
| Fujian | Site 2 | 3/2 | 13_Eucalyptus urophylla hybrid Leaf | (5) | CSFF 27807 | T14_B33_N/A_N/A | N/A | TB genotype 7 | *Ca. pacifica* | PV756550 | PV758116 | N/A | N/A |
| Fujian | Site 2 | 3/2 | 14_Syzygium jambos Leaf | (1) | CSFF 27748 | T14_B33_N/A_N/A | N/A | TB genotype 7 | *Ca. pacifica* | PV756491 | PV758057 | N/A | N/A |
| Fujian | Site 2 | 3/2 | 14_Syzygium jambos Leaf | (1) | CSFF 27749 | T14_B33_N/A_N/A | N/A | TB genotype 7 | *Ca. pacifica* | PV756492 | PV758058 | N/A | N/A |
| Fujian | Site 2 | 3/2 | 14_Syzygium jambos Leaf | (2) | CSFF 27750 | T14_B33_N/A_N/A | N/A | TB genotype 7 | *Ca. pacifica* | PV756493 | PV758059 | N/A | N/A |
| Fujian | Site 2 | 3/2 | 14_Syzygium jambos Leaf | (2) | CSFF 27751 | T14_B33_N/A_N/A | N/A | TB genotype 7 | *Ca. pacifica* | PV756494 | PV758060 | N/A | N/A |
| Fujian | Site 2 | 3/2 | 14_Syzygium jambos Leaf | (3) | CSFF 27752 | T14_B33_N/A_N/A | N/A | TB genotype 7 | *Ca. pacifica* | PV756495 | PV758061 | N/A | N/A |
| Fujian | Site 2 | 3/2 | 14_Syzygium jambos Leaf | (3) | CSFF 27753 | T14_B33_N/A_N/A | N/A | TB genotype 7 | *Ca. pacifica* | PV756496 | PV758062 | N/A | N/A |
| Fujian | Site 2 | 3/2 | 14_Syzygium jambos Leaf | (4) | CSFF 27754 | T14_B33_N/A_N/A | N/A | TB genotype 7 | *Ca. pacifica* | PV756497 | PV758063 | N/A | N/A |
| Fujian | Site 2 | 3/2 | 14_Syzygium jambos Leaf | (4) | CSFF 27755 | T14_B33_N/A_N/A | N/A | TB genotype 7 | *Ca. pacifica* | PV756498 | PV758064 | N/A | N/A |
| Fujian | Site 2 | 3/2 | 14_Syzygium jambos Leaf | (5) | CSFF 27756 | T14_B33_N/A_N/A | N/A | TB genotype 7 | *Ca. pacifica* | PV756499 | PV758065 | N/A | N/A |
| Fujian | Site 2 | 3/2 | 14_Syzygium jambos Leaf | (5) | CSFF 27757 | T14_B33_N/A_N/A | N/A | TB genotype 7 | *Ca. pacifica* | PV756500 | PV758066 | N/A | N/A |
| Fujian | Site 2 | 3/2 | 15_Pennisetum purpureum Leaf | (1) | CSFF 27808 | T14_B33_N/A_N/A | N/A | TB genotype 7 | *Ca. pacifica* | PV756551 | PV758117 | N/A | N/A |
| Fujian | Site 2 | 3/2 | 15_Pennisetum purpureum Leaf | (1) | CSFF 27809 | T14_B33_N/A_N/A | N/A | TB genotype 7 | *Ca. pacifica* | PV756552 | PV758118 | N/A | N/A |
| Fujian | Site 2 | 3/2 | 15_Pennisetum purpureum Leaf | (2) | CSFF 27810 | T14_B33_N/A_N/A | N/A | TB genotype 7 | *Ca. pacifica* | PV756553 | PV758119 | N/A | N/A |
| Fujian | Site 2 | 3/2 | 15_Pennisetum purpureum Leaf | (2) | CSFF 27811 | T14_B33_N/A_N/A | N/A | TB genotype 7 | *Ca. pacifica* | PV756554 | PV758120 | N/A | N/A |
| Fujian | Site 2 | 3/2 | 15_Pennisetum purpureum Leaf | (3) | CSFF 27812 | T14_B33_N/A_N/A | N/A | TB genotype 7 | *Ca. pacifica* | PV756555 | PV758121 | N/A | N/A |
| Fujian | Site 2 | 3/2 | 15_Pennisetum purpureum Leaf | (3) | CSFF 27813 | T24_B16_N/A_N/A | N/A | TB genotype 25 | *Ca. kyotensis* | PV756556 | PV758122 | N/A | N/A |
| Fujian | Site 2 | 3/2 | 15_Pennisetum purpureum Leaf | (4) | CSFF 27814 | T14_B33_N/A_N/A | N/A | TB genotype 7 | *Ca. pacifica* | PV756557 | PV758123 | N/A | N/A |
| Fujian | Site 2 | 3/2 | 15_Pennisetum purpureum Leaf | (4) | CSFF 27815 | T14_B33_N/A_N/A | N/A | TB genotype 7 | *Ca. pacifica* | PV756558 | PV758124 | N/A | N/A |
| Fujian | Site 2 | 3/2 | 15_Pennisetum purpureum Leaf | (5) | CSFF 27816 | T14_B33_N/A_N/A | N/A | TB genotype 7 | *Ca. pacifica* | PV756559 | PV758125 | N/A | N/A |
| Fujian | Site 2 | 3/2 | 15_Pennisetum purpureum Leaf | (5) | CSFF 27817 | T14_B33_N/A_N/A | N/A | TB genotype 7 | *Ca. pacifica* | PV756560 | PV758126 | N/A | N/A |
| Fujian | Site 2 | 3/2 | 16_Saccharum officinarum Leaf | (1) | CSFF 27728 | T14_B33_N/A_N/A | N/A | TB genotype 7 | *Ca. pacifica* | PV756471 | PV758037 | N/A | N/A |
| Fujian | Site 2 | 3/2 | 16_Saccharum officinarum Leaf | (1) | CSFF 27729 | T14_B33_N/A_N/A | N/A | TB genotype 7 | *Ca. pacifica* | PV756472 | PV758038 | N/A | N/A |
| Fujian | Site 2 | 3/2 | 16_Saccharum officinarum Leaf | (2) | CSFF 27730 | T14_B33_N/A_N/A | N/A | TB genotype 7 | *Ca. pacifica* | PV756473 | PV758039 | N/A | N/A |
| Fujian | Site 2 | 3/2 | 16_Saccharum officinarum Leaf | (2) | CSFF 27731 | T14_B33_N/A_N/A | N/A | TB genotype 7 | *Ca. pacifica* | PV756474 | PV758040 | N/A | N/A |
| Fujian | Site 2 | 3/2 | 16_Saccharum officinarum Leaf | (3) | CSFF 27732 | T14_B33_N/A_N/A | N/A | TB genotype 7 | *Ca. pacifica* | PV756475 | PV758041 | N/A | N/A |
| Fujian | Site 2 | 3/2 | 16_Saccharum officinarum Leaf | (3) | CSFF 27733 | T14_B33_N/A_N/A | N/A | TB genotype 7 | *Ca. pacifica* | PV756476 | PV758042 | N/A | N/A |
| Fujian | Site 2 | 3/2 | 16_Saccharum officinarum Leaf | (4) | CSFF 27734 | T21_B13_N/A_N/A | N/A | TB genotype 20 | *Ca. hongkongensis* | PV756477 | PV758043 | N/A | N/A |
| Fujian | Site 2 | 3/2 | 16_Saccharum officinarum Leaf | (4) | CSFF 27735 | T14_B33_N/A_N/A | N/A | TB genotype 7 | *Ca. pacifica* | PV756478 | PV758044 | N/A | N/A |
| Fujian | Site 2 | 3/2 | 16_Saccharum officinarum Leaf | (5) | CSFF 27736 | T14_B33_N/A_N/A | N/A | TB genotype 7 | *Ca. pacifica* | PV756479 | PV758045 | N/A | N/A |
| Fujian | Site 2 | 3/2 | 16_Saccharum officinarum Leaf | (5) | CSFF 27737 | T14_B33_N/A_N/A | N/A | TB genotype 7 | *Ca. pacifica* | PV756480 | PV758046 | N/A | N/A |
| Fujian | Site 2 | 3/2 | 17_Rosa chinensis Leaf | (1) | CSFF 27698 | T14_B33_N/A_N/A | N/A | TB genotype 7 | *Ca. pacifica* | PV756441 | PV758007 | N/A | N/A |
| Fujian | Site 2 | 3/2 | 17_Rosa chinensis Leaf | (1) | CSFF 27699 | T21_B13_H17_C11 | TBHC genotype 23 | TB genotype 20 | *Ca. hongkongensis* | PV756442 | PV758008 | PV757490 | PV757632 |
| Fujian | Site 2 | 3/2 | 17_Rosa chinensis Leaf | (2) | CSFF 27700 | T14_B33_N/A_N/A | N/A | TB genotype 7 | *Ca. pacifica* | PV756443 | PV758009 | N/A | N/A |
| Fujian | Site 2 | 3/2 | 17_Rosa chinensis Leaf | (2) | CSFF 27701 | T14_B33_N/A_N/A | N/A | TB genotype 7 | *Ca. pacifica* | PV756444 | PV758010 | N/A | N/A |
| Fujian | Site 2 | 3/2 | 17_Rosa chinensis Leaf | (3) | CSFF 27702 | T14_B33_N/A_N/A | N/A | TB genotype 7 | *Ca. pacifica* | PV756445 | PV758011 | N/A | N/A |
| Fujian | Site 2 | 3/2 | 17_Rosa chinensis Leaf | (3) | CSFF 27703 | T14_B33_N/A_N/A | N/A | TB genotype 7 | *Ca. pacifica* | PV756446 | PV758012 | N/A | N/A |
| Fujian | Site 2 | 3/2 | 17_Rosa chinensis Leaf | (4) | CSFF 27704 | T14_B33_N/A_N/A | N/A | TB genotype 7 | *Ca. pacifica* | PV756447 | PV758013 | N/A | N/A |
| Fujian | Site 2 | 3/2 | 17_Rosa chinensis Leaf | (4) | CSFF 27705 | T14_B33_N/A_N/A | N/A | TB genotype 7 | *Ca. pacifica* | PV756448 | PV758014 | N/A | N/A |
| Fujian | Site 2 | 3/2 | 17_Rosa chinensis Leaf | (5) | CSFF 27706 | T14_B33_N/A_N/A | N/A | TB genotype 7 | *Ca. pacifica* | PV756449 | PV758015 | N/A | N/A |
| Fujian | Site 2 | 3/2 | 17_Rosa chinensis Leaf | (5) | CSFF 27707 | T14_B33_N/A_N/A | N/A | TB genotype 7 | *Ca. pacifica* | PV756450 | PV758016 | N/A | N/A |
| Fujian | Site 2 | 3/2 | 18_Acacia mangium Leaf | (1) | CSFF 27738 | T14_B33_N/A_N/A | N/A | TB genotype 7 | *Ca. pacifica* | PV756481 | PV758047 | N/A | N/A |
| Fujian | Site 2 | 3/2 | 18_Acacia mangium Leaf | (1) | CSFF 27739 | T14_B33_N/A_N/A | N/A | TB genotype 7 | *Ca. pacifica* | PV756482 | PV758048 | N/A | N/A |
| Fujian | Site 2 | 3/2 | 18_Acacia mangium Leaf | (2) | CSFF 27740 | T14_B33_N/A_N/A | N/A | TB genotype 7 | *Ca. pacifica* | PV756483 | PV758049 | N/A | N/A |
| Fujian | Site 2 | 3/2 | 18_Acacia mangium Leaf | (2) | CSFF 27741 | T14_B33_N/A_N/A | N/A | TB genotype 7 | *Ca. pacifica* | PV756484 | PV758050 | N/A | N/A |
| Fujian | Site 2 | 3/2 | 18_Acacia mangium Leaf | (3) | CSFF 27742 | T14_B33_N/A_N/A | N/A | TB genotype 7 | *Ca. pacifica* | PV756485 | PV758051 | N/A | N/A |
| Fujian | Site 2 | 3/2 | 18_Acacia mangium Leaf | (3) | CSFF 27743 | T14_B33_N/A_N/A | N/A | TB genotype 7 | *Ca. pacifica* | PV756486 | PV758052 | N/A | N/A |
| Fujian | Site 2 | 3/2 | 18_Acacia mangium Leaf | (4) | CSFF 27744 | T14_B33_N/A_N/A | N/A | TB genotype 7 | *Ca. pacifica* | PV756487 | PV758053 | N/A | N/A |
| Fujian | Site 2 | 3/2 | 18_Acacia mangium Leaf | (4) | CSFF 27745 | T14_B33_N/A_N/A | N/A | TB genotype 7 | *Ca. pacifica* | PV756488 | PV758054 | N/A | N/A |
| Fujian | Site 2 | 3/2 | 18_Acacia mangium Leaf | (5) | CSFF 27746 | T14_B33_N/A_N/A | N/A | TB genotype 7 | *Ca. pacifica* | PV756489 | PV758055 | N/A | N/A |
| Fujian | Site 2 | 3/2 | 18_Acacia mangium Leaf | (5) | CSFF 27747 | T14_B33_N/A_N/A | N/A | TB genotype 7 | *Ca. pacifica* | PV756490 | PV758056 | N/A | N/A |
| Fujian | Site 2 | 3/2 | 19_Dianthus caryophyllus Leaf | (1) | CSFF 27688 | T14_B33_H12_C21 | TBHC genotype 7 | TB genotype 7 | *Ca. pacifica* | PV756431 | PV757997 | PV757488 | PV757630 |
| Fujian | Site 2 | 3/2 | 19_Dianthus caryophyllus Leaf | (1) | CSFF 27689 | T14_B33_N/A_N/A | N/A | TB genotype 7 | *Ca. pacifica* | PV756432 | PV757998 | N/A | N/A |
| Fujian | Site 2 | 3/2 | 19_Dianthus caryophyllus Leaf | (2) | CSFF 27690 | T14_B33_N/A_N/A | N/A | TB genotype 7 | *Ca. pacifica* | PV756433 | PV757999 | N/A | N/A |
| Fujian | Site 2 | 3/2 | 19_Dianthus caryophyllus Leaf | (2) | CSFF 27691 | T14_B33_N/A_N/A | N/A | TB genotype 7 | *Ca. pacifica* | PV756434 | PV758000 | N/A | N/A |
| Fujian | Site 2 | 3/2 | 19_Dianthus caryophyllus Leaf | (3) | CSFF 27692 | T14_B33_N/A_N/A | N/A | TB genotype 7 | *Ca. pacifica* | PV756435 | PV758001 | N/A | N/A |
| Fujian | Site 2 | 3/2 | 19_Dianthus caryophyllus Leaf | (3) | CSFF 27693 | T24_B16_H18_C15 | TBHC genotype 28 | TB genotype 25 | *Ca. kyotensis* | PV756436 | PV758002 | PV757489 | PV757631 |
| Fujian | Site 2 | 3/2 | 19_Dianthus caryophyllus Leaf | (4) | CSFF 27694 | T14_B33_N/A_N/A | N/A | TB genotype 7 | *Ca. pacifica* | PV756437 | PV758003 | N/A | N/A |
| Fujian | Site 2 | 3/2 | 19_Dianthus caryophyllus Leaf | (4) | CSFF 27695 | T14_B33_N/A_N/A | N/A | TB genotype 7 | *Ca. pacifica* | PV756438 | PV758004 | N/A | N/A |
| Fujian | Site 2 | 3/2 | 19_Dianthus caryophyllus Leaf | (5) | CSFF 27696 | T14_B33_N/A_N/A | N/A | TB genotype 7 | *Ca. pacifica* | PV756439 | PV758005 | N/A | N/A |
| Fujian | Site 2 | 3/2 | 19_Dianthus caryophyllus Leaf | (5) | CSFF 27697 | T14_B33_N/A_N/A | N/A | TB genotype 7 | *Ca. pacifica* | PV756440 | PV758006 | N/A | N/A |
| Fujian | Site 2 | 3/2 | 20_Cinnamomum camphora Leaf | (1) | CSFF 27708 | T14_B33_N/A_N/A | N/A | TB genotype 7 | *Ca. pacifica* | PV756451 | PV758017 | N/A | N/A |
| Fujian | Site 2 | 3/2 | 20_Cinnamomum camphora Leaf | (1) | CSFF 27709 | T14_B33_N/A_N/A | N/A | TB genotype 7 | *Ca. pacifica* | PV756452 | PV758018 | N/A | N/A |
| Fujian | Site 2 | 3/2 | 20_Cinnamomum camphora Leaf | (2) | CSFF 27710 | T14_B33_N/A_N/A | N/A | TB genotype 7 | *Ca. pacifica* | PV756453 | PV758019 | N/A | N/A |
| Fujian | Site 2 | 3/2 | 20_Cinnamomum camphora Leaf | (2) | CSFF 27711 | T14_B33_N/A_N/A | N/A | TB genotype 7 | *Ca. pacifica* | PV756454 | PV758020 | N/A | N/A |
| Fujian | Site 2 | 3/2 | 20_Cinnamomum camphora Leaf | (3) | CSFF 27712 | T14_B33_N/A_N/A | N/A | TB genotype 7 | *Ca. pacifica* | PV756455 | PV758021 | N/A | N/A |
| Fujian | Site 2 | 3/2 | 20_Cinnamomum camphora Leaf | (3) | CSFF 27713 | T14_B33_N/A_N/A | N/A | TB genotype 7 | *Ca. pacifica* | PV756456 | PV758022 | N/A | N/A |
| Fujian | Site 2 | 3/2 | 20_Cinnamomum camphora Leaf | (4) | CSFF 27714 | T14_B33_N/A_N/A | N/A | TB genotype 7 | *Ca. pacifica* | PV756457 | PV758023 | N/A | N/A |
| Fujian | Site 2 | 3/2 | 20_Cinnamomum camphora Leaf | (4) | CSFF 27715 | T14_B33_N/A_N/A | N/A | TB genotype 7 | *Ca. pacifica* | PV756458 | PV758024 | N/A | N/A |
| Fujian | Site 2 | 3/2 | 20_Cinnamomum camphora Leaf | (5) | CSFF 27716 | T14_B33_N/A_N/A | N/A | TB genotype 7 | *Ca. pacifica* | PV756459 | PV758025 | N/A | N/A |
| Fujian | Site 2 | 3/2 | 20_Cinnamomum camphora Leaf | (5) | CSFF 27717 | T14_B33_N/A_N/A | N/A | TB genotype 7 | *Ca. pacifica* | PV756460 | PV758026 | N/A | N/A |
| Fujian | Site 2 | 3/2 | 21_Washingtonia filifera Leaf | (1) | CSFF 27718 | T14_B33_N/A_N/A | N/A | TB genotype 7 | *Ca. pacifica* | PV756461 | PV758027 | N/A | N/A |
| Fujian | Site 2 | 3/2 | 21_Washingtonia filifera Leaf | (1) | CSFF 27719 | T24_B16_N/A_N/A | N/A | TB genotype 25 | *Ca. kyotensis* | PV756462 | PV758028 | N/A | N/A |
| Fujian | Site 2 | 3/2 | 21_Washingtonia filifera Leaf | (2) | CSFF 27720 | T14_B33_N/A_N/A | N/A | TB genotype 7 | *Ca. pacifica* | PV756463 | PV758029 | N/A | N/A |
| Fujian | Site 2 | 3/2 | 21_Washingtonia filifera Leaf | (2) | CSFF 27721 | T14_B33_N/A_N/A | N/A | TB genotype 7 | *Ca. pacifica* | PV756464 | PV758030 | N/A | N/A |
| Fujian | Site 2 | 3/2 | 21_Washingtonia filifera Leaf | (3) | CSFF 27722 | T14_B33_N/A_N/A | N/A | TB genotype 7 | *Ca. pacifica* | PV756465 | PV758031 | N/A | N/A |
| Fujian | Site 2 | 3/2 | 21_Washingtonia filifera Leaf | (3) | CSFF 27723 | T14_B33_N/A_N/A | N/A | TB genotype 7 | *Ca. pacifica* | PV756466 | PV758032 | N/A | N/A |
| Fujian | Site 2 | 3/2 | 21_Washingtonia filifera Leaf | (4) | CSFF 27724 | T14_B33_N/A_N/A | N/A | TB genotype 7 | *Ca. pacifica* | PV756467 | PV758033 | N/A | N/A |
| Fujian | Site 2 | 3/2 | 21_Washingtonia filifera Leaf | (4) | CSFF 27725 | T14_B33_N/A_N/A | N/A | TB genotype 7 | *Ca. pacifica* | PV756468 | PV758034 | N/A | N/A |
| Fujian | Site 2 | 3/2 | 21_Washingtonia filifera Leaf | (5) | CSFF 27726 | T14_B33_N/A_N/A | N/A | TB genotype 7 | *Ca. pacifica* | PV756469 | PV758035 | N/A | N/A |
| Fujian | Site 2 | 3/2 | 21_Washingtonia filifera Leaf | (5) | CSFF 27727 | T14_B33_N/A_N/A | N/A | TB genotype 7 | *Ca. pacifica* | PV756470 | PV758036 | N/A | N/A |
| Fujian | Site 2 | 3/2 | 22_Ficus benjamina Leaf | (1) | CSFF 27758 | T14_B33_N/A_N/A | N/A | TB genotype 7 | *Ca. pacifica* | PV756501 | PV758067 | N/A | N/A |
| Fujian | Site 2 | 3/2 | 22_Ficus benjamina Leaf | (1) | CSFF 27759 | T14_B33_N/A_N/A | N/A | TB genotype 7 | *Ca. pacifica* | PV756502 | PV758068 | N/A | N/A |
| Fujian | Site 2 | 3/2 | 22_Ficus benjamina Leaf | (2) | CSFF 27760 | T14_B33_N/A_N/A | N/A | TB genotype 7 | *Ca. pacifica* | PV756503 | PV758069 | N/A | N/A |
| Fujian | Site 2 | 3/2 | 22_Ficus benjamina Leaf | (2) | CSFF 27761 | T14_B33_N/A_N/A | N/A | TB genotype 7 | *Ca. pacifica* | PV756504 | PV758070 | N/A | N/A |
| Fujian | Site 2 | 3/2 | 22_Ficus benjamina Leaf | (3) | CSFF 27762 | T14_B33_N/A_N/A | N/A | TB genotype 7 | *Ca. pacifica* | PV756505 | PV758071 | N/A | N/A |
| Fujian | Site 2 | 3/2 | 22_Ficus benjamina Leaf | (3) | CSFF 27763 | T14_B33_N/A_N/A | N/A | TB genotype 7 | *Ca. pacifica* | PV756506 | PV758072 | N/A | N/A |
| Fujian | Site 2 | 3/2 | 22_Ficus benjamina Leaf | (4) | CSFF 27764 | T14_B33_N/A_N/A | N/A | TB genotype 7 | *Ca. pacifica* | PV756507 | PV758073 | N/A | N/A |
| Fujian | Site 2 | 3/2 | 22_Ficus benjamina Leaf | (4) | CSFF 27765 | T14_B33_N/A_N/A | N/A | TB genotype 7 | *Ca. pacifica* | PV756508 | PV758074 | N/A | N/A |
| Fujian | Site 2 | 3/2 | 22_Ficus benjamina Leaf | (5) | CSFF 27766 | T14_B33_N/A_N/A | N/A | TB genotype 7 | *Ca. pacifica* | PV756509 | PV758075 | N/A | N/A |
| Fujian | Site 2 | 3/2 | 22_Ficus benjamina Leaf | (5) | CSFF 27767 | T24_B19_H18_C15 | TBHC genotype 32 | TB genotype 28 | *Ca. kyotensis* | PV756510 | PV758076 | PV757491 | PV757633 |
| Fujian | Site 2 | 3/2 | 23_Rhododendron pulchrum Leaf | (1) | CSFF 27782 | T14_B33_N/A_N/A | N/A | TB genotype 7 | *Ca. pacifica* | PV756525 | PV758091 | N/A | N/A |
| Fujian | Site 2 | 3/2 | 23_Rhododendron pulchrum Leaf | (1) | CSFF 27783 | T14_B33_N/A_N/A | N/A | TB genotype 7 | *Ca. pacifica* | PV756526 | PV758092 | N/A | N/A |
| Fujian | Site 2 | 3/2 | 23_Rhododendron pulchrum Leaf | (2) | CSFF 27784 | T14_B33_N/A_N/A | N/A | TB genotype 7 | *Ca. pacifica* | PV756527 | PV758093 | N/A | N/A |
| Fujian | Site 2 | 3/2 | 23_Rhododendron pulchrum Leaf | (2) | CSFF 27785 | T14_B33_N/A_N/A | N/A | TB genotype 7 | *Ca. pacifica* | PV756528 | PV758094 | N/A | N/A |
| Fujian | Site 2 | 3/2 | 23_Rhododendron pulchrum Leaf | (3) | CSFF 27786 | T14_B33_N/A_N/A | N/A | TB genotype 7 | *Ca. pacifica* | PV756529 | PV758095 | N/A | N/A |
| Fujian | Site 2 | 3/2 | 23_Rhododendron pulchrum Leaf | (3) | CSFF 27787 | T14_B33_N/A_N/A | N/A | TB genotype 7 | *Ca. pacifica* | PV756530 | PV758096 | N/A | N/A |
| Fujian | Site 2 | 3/2 | 23_Rhododendron pulchrum Leaf | (4) | CSFF 27788 | T14_B33_N/A_N/A | N/A | TB genotype 7 | *Ca. pacifica* | PV756531 | PV758097 | N/A | N/A |
| Fujian | Site 2 | 3/2 | 23_Rhododendron pulchrum Leaf | (4) | CSFF 27789 | T14_B33_N/A_N/A | N/A | TB genotype 7 | *Ca. pacifica* | PV756532 | PV758098 | N/A | N/A |
| Fujian | Site 2 | 3/2 | 23_Rhododendron pulchrum Leaf | (5) | CSFF 27790 | T14_B33_N/A_N/A | N/A | TB genotype 7 | *Ca. pacifica* | PV756533 | PV758099 | N/A | N/A |
| Fujian | Site 2 | 3/2 | 23_Rhododendron pulchrum Leaf | (5) | CSFF 27791 | T14_B33_N/A_N/A | N/A | TB genotype 7 | *Ca. pacifica* | PV756534 | PV758100 | N/A | N/A |
| Fujian | Site 2 | 3/2 | 24_Glycine max Stem | (3) | CSFF 27798 | T24_B16_N/A_N/A | N/A | TB genotype 25 | *Ca. kyotensis* | PV756541 | PV758107 | N/A | N/A |
| Fujian | Site 2 | 3/2 | 24_Glycine max Stem | (3) | CSFF 27799 | T14_B33_N/A_N/A | N/A | TB genotype 7 | *Ca. pacifica* | PV756542 | PV758108 | N/A | N/A |
| Fujian | Site 2 | 3/2 | 24_Glycine max Stem | (5) | CSFF 27800 | T25_B20_H18_C13 | TBHC genotype 42 | TB genotype 35 | *Ca. kyotensis* | PV756543 | PV758109 | PV757495 | PV757637 |
| Fujian | Site 2 | 3/2 | 24_Glycine max Stem | (5) | CSFF 27801 | T25_B20_N/A_N/A | N/A | TB genotype 35 | *Ca. kyotensis* | PV756544 | PV758110 | N/A | N/A |
| Fujian | Site 2 | 3/2 | 26_Medicago sativa Seed | (1) | CSFF 27818 | T14_B33_H12_C21 | TBHC genotype 7 | TB genotype 7 | *Ca. pacifica* | PV756561 | PV758127 | PV757497 | PV757639 |
| Fujian | Site 2 | 3/2 | 26_Medicago sativa Seed | (1) | CSFF 27819 | T14_B33_N/A_N/A | N/A | TB genotype 7 | *Ca. pacifica* | PV756562 | PV758128 | N/A | N/A |
| Fujian | Site 2 | 3/2 | 26_Medicago sativa Seed | (2) | CSFF 27820 | T14_B33_N/A_N/A | N/A | TB genotype 7 | *Ca. pacifica* | PV756563 | PV758129 | N/A | N/A |
| Fujian | Site 2 | 3/2 | 26_Medicago sativa Seed | (2) | CSFF 27821 | T14_B33_N/A_N/A | N/A | TB genotype 7 | *Ca. pacifica* | PV756564 | PV758130 | N/A | N/A |
| Fujian | Site 2 | 3/2 | 26_Medicago sativa Seed | (3) | CSFF 27822 | T14_B33_N/A_N/A | N/A | TB genotype 7 | *Ca. pacifica* | PV756565 | PV758131 | N/A | N/A |
| Fujian | Site 2 | 3/2 | 26_Medicago sativa Seed | (3) | CSFF 27823 | T14_B33_N/A_N/A | N/A | TB genotype 7 | *Ca. pacifica* | PV756566 | PV758132 | N/A | N/A |
| Fujian | Site 2 | 3/2 | 26_Medicago sativa Seed | (4) | CSFF 27824 | T14_B33_N/A_N/A | N/A | TB genotype 7 | *Ca. pacifica* | PV756567 | PV758133 | N/A | N/A |
| Fujian | Site 2 | 3/2 | 26_Medicago sativa Seed | (4) | CSFF 27825 | T14_B33_N/A_N/A | N/A | TB genotype 7 | *Ca. pacifica* | PV756568 | PV758134 | N/A | N/A |
| Fujian | Site 2 | 3/2 | 26_Medicago sativa Seed | (5) | CSFF 27826 | T14_B33_N/A_N/A | N/A | TB genotype 7 | *Ca. pacifica* | PV756569 | PV758135 | N/A | N/A |
| Fujian | Site 2 | 3/2 | 26_Medicago sativa Seed | (5) | CSFF 27827 | T14_B33_N/A_N/A | N/A | TB genotype 7 | *Ca. pacifica* | PV756570 | PV758136 | N/A | N/A |
| Fujian | Site 3 | 3/3 | 1_Brassica rapa subsp. pekinensis Seed | (1) | CSFF 27862 | T11_B25_N/A_N/A | N/A | TB genotype 1 | *Ca. aconidialis* | PV756605 | PV758171 | N/A | N/A |
| Fujian | Site 3 | 3/3 | 1_Brassica rapa subsp. pekinensis Seed | (1) | CSFF 27863 | T11_B25_N/A_N/A | N/A | TB genotype 1 | *Ca. aconidialis* | PV756606 | PV758172 | N/A | N/A |
| Fujian | Site 3 | 3/3 | 1_Brassica rapa subsp. pekinensis Seed | (3) | CSFF 27864 | T11_B25_N/A_N/A | N/A | TB genotype 1 | *Ca. aconidialis* | PV756607 | PV758173 | N/A | N/A |
| Fujian | Site 3 | 3/3 | 1_Brassica rapa subsp. pekinensis Seed | (3) | CSFF 27865 | T11_B25_N/A_N/A | N/A | TB genotype 1 | *Ca. aconidialis* | PV756608 | PV758174 | N/A | N/A |
| Fujian | Site 3 | 3/3 | 1_Brassica rapa subsp. pekinensis Seed | (4) | CSFF 27866 | T11_B25_N/A_N/A | N/A | TB genotype 1 | *Ca. aconidialis* | PV756609 | PV758175 | N/A | N/A |
| Fujian | Site 3 | 3/3 | 1_Brassica rapa subsp. pekinensis Seed | (4) | CSFF 27867 | T11_B25_N/A_N/A | N/A | TB genotype 1 | *Ca. aconidialis* | PV756610 | PV758176 | N/A | N/A |
| Fujian | Site 3 | 3/3 | 2_Zinnia elegans Seed | (3) | CSFF 27858 | T11_B28_N/A_N/A | N/A | TB genotype 3 | *Ca. aconidialis* | PV756601 | PV758167 | N/A | N/A |
| Fujian | Site 3 | 3/3 | 2_Zinnia elegans Seed | (3) | CSFF 27859 | T11_B28_N/A_N/A | N/A | TB genotype 3 | *Ca. aconidialis* | PV756602 | PV758168 | N/A | N/A |
| Fujian | Site 3 | 3/3 | 2_Zinnia elegans Seed | (4) | CSFF 27860 | T11_B25_N/A_N/A | N/A | TB genotype 1 | *Ca. aconidialis* | PV756603 | PV758169 | N/A | N/A |
| Fujian | Site 3 | 3/3 | 2_Zinnia elegans Seed | (4) | CSFF 27861 | T11_B25_N/A_N/A | N/A | TB genotype 1 | *Ca. aconidialis* | PV756604 | PV758170 | N/A | N/A |
| Fujian | Site 3 | 3/3 | 3_Lolium perenne Seed | (1) | CSFF 27882 | T11_B25_N/A_N/A | N/A | TB genotype 1 | *Ca. aconidialis* | PV756625 | PV758191 | N/A | N/A |
| Fujian | Site 3 | 3/3 | 3_Lolium perenne Seed | (1) | CSFF 27883 | T11_B25_N/A_N/A | N/A | TB genotype 1 | *Ca. aconidialis* | PV756626 | PV758192 | N/A | N/A |
| Fujian | Site 3 | 3/3 | 3_Lolium perenne Seed | (4) | CSFF 27884 | T11_B25_N/A_N/A | N/A | TB genotype 1 | *Ca. aconidialis* | PV756627 | PV758193 | N/A | N/A |
| Fujian | Site 3 | 3/3 | 3_Lolium perenne Seed | (4) | CSFF 27885 | T11_B25_N/A_N/A | N/A | TB genotype 1 | *Ca. aconidialis* | PV756628 | PV758194 | N/A | N/A |
| Fujian | Site 3 | 3/3 | 3_Lolium perenne Seed | (5) | CSFF 27886 | T11_B25_N/A_N/A | N/A | TB genotype 1 | *Ca. aconidialis* | PV756629 | PV758195 | N/A | N/A |
| Fujian | Site 3 | 3/3 | 3_Lolium perenne Seed | (5) | CSFF 27887 | T11_B25_N/A_N/A | N/A | TB genotype 1 | *Ca. aconidialis* | PV756630 | PV758196 | N/A | N/A |
| Fujian | Site 3 | 3/3 | 4_Salvia hispanica Seed | (1) | CSFF 27868 | T11_B25_N/A_N/A | N/A | TB genotype 1 | *Ca. aconidialis* | PV756611 | PV758177 | N/A | N/A |
| Fujian | Site 3 | 3/3 | 4_Salvia hispanica Seed | (1) | CSFF 27869 | T11_B25_N/A_N/A | N/A | TB genotype 1 | *Ca. aconidialis* | PV756612 | PV758178 | N/A | N/A |
| Fujian | Site 3 | 3/3 | 4_Salvia hispanica Seed | (3) | CSFF 27870 | T11_B25_N/A_N/A | N/A | TB genotype 1 | *Ca. aconidialis* | PV756613 | PV758179 | N/A | N/A |
| Fujian | Site 3 | 3/3 | 4_Salvia hispanica Seed | (3) | CSFF 27871 | T13_B25_H11_C20 | TBHC genotype 6 | TB genotype 6 | *Ca. aconidialis* | PV756614 | PV758180 | PV757503 | PV757645 |
| Fujian | Site 3 | 3/3 | 4_Salvia hispanica Seed | (4) | CSFF 27872 | T11_B26_N/A_N/A | N/A | TB genotype 2 | *Ca. aconidialis* | PV756615 | PV758181 | N/A | N/A |
| Fujian | Site 3 | 3/3 | 4_Salvia hispanica Seed | (4) | CSFF 27873 | T11_B26_N/A_N/A | N/A | TB genotype 2 | *Ca. aconidialis* | PV756616 | PV758182 | N/A | N/A |
| Fujian | Site 3 | 3/3 | 4_Salvia hispanica Seed | (5) | CSFF 27874 | T11_B25_N/A_N/A | N/A | TB genotype 1 | *Ca. aconidialis* | PV756617 | PV758183 | N/A | N/A |
| Fujian | Site 3 | 3/3 | 4_Salvia hispanica Seed | (5) | CSFF 27875 | T11_B25_N/A_N/A | N/A | TB genotype 1 | *Ca. aconidialis* | PV756618 | PV758184 | N/A | N/A |
| Fujian | Site 3 | 3/3 | 5_Impatiens balsamina Seed | (1) | CSFF 27876 | T11_B25_N/A_N/A | N/A | TB genotype 1 | *Ca. aconidialis* | PV756619 | PV758185 | N/A | N/A |
| Fujian | Site 3 | 3/3 | 5_Impatiens balsamina Seed | (1) | CSFF 27877 | T11_B25_N/A_N/A | N/A | TB genotype 1 | *Ca. aconidialis* | PV756620 | PV758186 | N/A | N/A |
| Fujian | Site 3 | 3/3 | 5_Impatiens balsamina Seed | (2) | CSFF 27878 | T11_B28_N/A_N/A | N/A | TB genotype 3 | *Ca. aconidialis* | PV756621 | PV758187 | N/A | N/A |
| Fujian | Site 3 | 3/3 | 5_Impatiens balsamina Seed | (2) | CSFF 27879 | T11_B28_N/A_N/A | N/A | TB genotype 3 | *Ca. aconidialis* | PV756622 | PV758188 | N/A | N/A |
| Fujian | Site 3 | 3/3 | 5_Impatiens balsamina Seed | (5) | CSFF 27880 | T11_B25_N/A_N/A | N/A | TB genotype 1 | *Ca. aconidialis* | PV756623 | PV758189 | N/A | N/A |
| Fujian | Site 3 | 3/3 | 5_Impatiens balsamina Seed | (5) | CSFF 27881 | T11_B25_N/A_N/A | N/A | TB genotype 1 | *Ca. aconidialis* | PV756624 | PV758190 | N/A | N/A |
| Fujian | Site 3 | 3/3 | 6_Sesamum indicum Seed | (1) | CSFF 27844 | T24_B20_H18_C13 | TBHC genotype 33 | TB genotype 29 | *Ca. kyotensis* | PV756587 | PV758153 | PV757501 | PV757643 |
| Fujian | Site 3 | 3/3 | 6_Sesamum indicum Seed | (1) | CSFF 27845 | T24_B20_N/A_N/A | N/A | TB genotype 29 | *Ca. kyotensis* | PV756588 | PV758154 | N/A | N/A |
| Fujian | Site 3 | 3/3 | 6_Sesamum indicum Seed | (4) | CSFF 27846 | T11_B25_N/A_N/A | N/A | TB genotype 1 | *Ca. aconidialis* | PV756589 | PV758155 | N/A | N/A |
| Fujian | Site 3 | 3/3 | 6_Sesamum indicum Seed | (4) | CSFF 27847 | T11_B25_N/A_N/A | N/A | TB genotype 1 | *Ca. aconidialis* | PV756590 | PV758156 | N/A | N/A |
| Fujian | Site 3 | 3/3 | 6_Sesamum indicum Seed | (5) | CSFF 27848 | T11_B25_N/A_N/A | N/A | TB genotype 1 | *Ca. aconidialis* | PV756591 | PV758157 | N/A | N/A |
| Fujian | Site 3 | 3/3 | 6_Sesamum indicum Seed | (5) | CSFF 27849 | T11_B25_N/A_N/A | N/A | TB genotype 1 | *Ca. aconidialis* | PV756592 | PV758158 | N/A | N/A |
| Fujian | Site 3 | 3/3 | 7_Dianthus caryophyllus Seed | (1) | CSFF 27850 | T11_B26_H11_C20 | TBHC genotype 2 | TB genotype 2 | *Ca. aconidialis* | PV756593 | PV758159 | PV757502 | PV757644 |
| Fujian | Site 3 | 3/3 | 7_Dianthus caryophyllus Seed | (1) | CSFF 27851 | T11_B26_N/A_N/A | N/A | TB genotype 2 | *Ca. aconidialis* | PV756594 | PV758160 | N/A | N/A |
| Fujian | Site 3 | 3/3 | 7_Dianthus caryophyllus Seed | (2) | CSFF 27852 | T11_B25_N/A_N/A | N/A | TB genotype 1 | *Ca. aconidialis* | PV756595 | PV758161 | N/A | N/A |
| Fujian | Site 3 | 3/3 | 7_Dianthus caryophyllus Seed | (2) | CSFF 27853 | T11_B25_N/A_N/A | N/A | TB genotype 1 | *Ca. aconidialis* | PV756596 | PV758162 | N/A | N/A |
| Fujian | Site 3 | 3/3 | 7_Dianthus caryophyllus Seed | (3) | CSFF 27854 | T11_B25_N/A_N/A | N/A | TB genotype 1 | *Ca. aconidialis* | PV756597 | PV758163 | N/A | N/A |
| Fujian | Site 3 | 3/3 | 7_Dianthus caryophyllus Seed | (3) | CSFF 27855 | T11_B25_N/A_N/A | N/A | TB genotype 1 | *Ca. aconidialis* | PV756598 | PV758164 | N/A | N/A |
| Fujian | Site 3 | 3/3 | 7_Dianthus caryophyllus Seed | (4) | CSFF 27856 | T11_B25_N/A_N/A | N/A | TB genotype 1 | *Ca. aconidialis* | PV756599 | PV758165 | N/A | N/A |
| Fujian | Site 3 | 3/3 | 7_Dianthus caryophyllus Seed | (4) | CSFF 27857 | T11_B25_N/A_N/A | N/A | TB genotype 1 | *Ca. aconidialis* | PV756600 | PV758166 | N/A | N/A |
| Fujian | Site 3 | 3/3 | 8_Trifolium pratense Seed | (1) | CSFF 27834 | T11_B25_N/A_N/A | N/A | TB genotype 1 | *Ca. aconidialis* | PV756577 | PV758143 | N/A | N/A |
| Fujian | Site 3 | 3/3 | 8_Trifolium pratense Seed | (1) | CSFF 27835 | T11_B25_N/A_N/A | N/A | TB genotype 1 | *Ca. aconidialis* | PV756578 | PV758144 | N/A | N/A |
| Fujian | Site 3 | 3/3 | 8_Trifolium pratense Seed | (2) | CSFF 27836 | T11_B25_N/A_N/A | N/A | TB genotype 1 | *Ca. aconidialis* | PV756579 | PV758145 | N/A | N/A |
| Fujian | Site 3 | 3/3 | 8_Trifolium pratense Seed | (2) | CSFF 27837 | T11_B25_N/A_N/A | N/A | TB genotype 1 | *Ca. aconidialis* | PV756580 | PV758146 | N/A | N/A |
| Fujian | Site 3 | 3/3 | 8_Trifolium pratense Seed | (3) | CSFF 27838 | T11_B25_N/A_N/A | N/A | TB genotype 1 | *Ca. aconidialis* | PV756581 | PV758147 | N/A | N/A |
| Fujian | Site 3 | 3/3 | 8_Trifolium pratense Seed | (3) | CSFF 27839 | T11_B25_N/A_N/A | N/A | TB genotype 1 | *Ca. aconidialis* | PV756582 | PV758148 | N/A | N/A |
| Fujian | Site 3 | 3/3 | 8_Trifolium pratense Seed | (4) | CSFF 27840 | T11_B25_N/A_N/A | N/A | TB genotype 1 | *Ca. aconidialis* | PV756583 | PV758149 | N/A | N/A |
| Fujian | Site 3 | 3/3 | 8_Trifolium pratense Seed | (4) | CSFF 27841 | T11_B25_N/A_N/A | N/A | TB genotype 1 | *Ca. aconidialis* | PV756584 | PV758150 | N/A | N/A |
| Fujian | Site 3 | 3/3 | 8_Trifolium pratense Seed | (5) | CSFF 27842 | T11_B28_N/A_N/A | N/A | TB genotype 3 | *Ca. aconidialis* | PV756585 | PV758151 | N/A | N/A |
| Fujian | Site 3 | 3/3 | 8_Trifolium pratense Seed | (5) | CSFF 27843 | T11_B26_H11_C20 | TBHC genotype 2 | TB genotype 2 | *Ca. aconidialis* | PV756586 | PV758152 | PV757500 | PV757642 |
| Fujian | Site 3 | 3/3 | 9_Lythrum salicaria Seed | (2) | CSFF 27828 | T11_B25_H11_C20 | TBHC genotype 1 | TB genotype 1 | *Ca. aconidialis* | PV756571 | PV758137 | PV757498 | PV757640 |
| Fujian | Site 3 | 3/3 | 9_Lythrum salicaria Seed | (2) | CSFF 27829 | T11_B25_N/A_N/A | N/A | TB genotype 1 | *Ca. aconidialis* | PV756572 | PV758138 | N/A | N/A |
| Fujian | Site 3 | 3/3 | 9_Lythrum salicaria Seed | (4) | CSFF 27830 | T11_B28_H11_C20 | TBHC genotype 3 | TB genotype 3 | *Ca. aconidialis* | PV756573 | PV758139 | PV757499 | PV757641 |
| Fujian | Site 3 | 3/3 | 9_Lythrum salicaria Seed | (4) | CSFF 27831 | T11_B25_N/A_N/A | N/A | TB genotype 1 | *Ca. aconidialis* | PV756574 | PV758140 | N/A | N/A |
| Fujian | Site 3 | 3/3 | 9_Lythrum salicaria Seed | (5) | CSFF 27832 | T11_B28_N/A_N/A | N/A | TB genotype 3 | *Ca. aconidialis* | PV756575 | PV758141 | N/A | N/A |
| Fujian | Site 3 | 3/3 | 9_Lythrum salicaria Seed | (5) | CSFF 27833 | T11_B28_N/A_N/A | N/A | TB genotype 3 | *Ca. aconidialis* | PV756576 | PV758142 | N/A | N/A |
| Fujian | Site 3 | 3/3 | 10_Buxus sinica Leaf | (3) | CSFF 27992 | T11_B25_N/A_N/A | N/A | TB genotype 1 | *Ca. aconidialis* | PV756735 | PV758301 | N/A | N/A |
| Fujian | Site 3 | 3/3 | 10_Buxus sinica Leaf | (3) | CSFF 27993 | T11_B25_N/A_N/A | N/A | TB genotype 1 | *Ca. aconidialis* | PV756736 | PV758302 | N/A | N/A |
| Fujian | Site 3 | 3/3 | 10_Buxus sinica Leaf | (4) | CSFF 27994 | T11_B25_N/A_N/A | N/A | TB genotype 1 | *Ca. aconidialis* | PV756737 | PV758303 | N/A | N/A |
| Fujian | Site 3 | 3/3 | 10_Buxus sinica Leaf | (4) | CSFF 27995 | T11_B25_N/A_N/A | N/A | TB genotype 1 | *Ca. aconidialis* | PV756738 | PV758304 | N/A | N/A |
| Fujian | Site 3 | 3/3 | 11_Imperata cylindrica Leaf | (1) | CSFF 27968 | T11_B25_N/A_N/A | N/A | TB genotype 1 | *Ca. aconidialis* | PV756711 | PV758277 | N/A | N/A |
| Fujian | Site 3 | 3/3 | 11_Imperata cylindrica Leaf | (1) | CSFF 27969 | T11_B28_N/A_N/A | N/A | TB genotype 3 | *Ca. aconidialis* | PV756712 | PV758278 | N/A | N/A |
| Fujian | Site 3 | 3/3 | 11_Imperata cylindrica Leaf | (2) | CSFF 27970 | T11_B25_N/A_N/A | N/A | TB genotype 1 | *Ca. aconidialis* | PV756713 | PV758279 | N/A | N/A |
| Fujian | Site 3 | 3/3 | 11_Imperata cylindrica Leaf | (2) | CSFF 27971 | T11_B28_N/A_N/A | N/A | TB genotype 3 | *Ca. aconidialis* | PV756714 | PV758280 | N/A | N/A |
| Fujian | Site 3 | 3/3 | 11_Imperata cylindrica Leaf | (3) | CSFF 27972 | T11_B25_N/A_N/A | N/A | TB genotype 1 | *Ca. aconidialis* | PV756715 | PV758281 | N/A | N/A |
| Fujian | Site 3 | 3/3 | 11_Imperata cylindrica Leaf | (3) | CSFF 27973 | T11_B25_N/A_N/A | N/A | TB genotype 1 | *Ca. aconidialis* | PV756716 | PV758282 | N/A | N/A |
| Fujian | Site 3 | 3/3 | 11_Imperata cylindrica Leaf | (4) | CSFF 27974 | T11_B25_N/A_N/A | N/A | TB genotype 1 | *Ca. aconidialis* | PV756717 | PV758283 | N/A | N/A |
| Fujian | Site 3 | 3/3 | 11_Imperata cylindrica Leaf | (4) | CSFF 27975 | T11_B25_N/A_N/A | N/A | TB genotype 1 | *Ca. aconidialis* | PV756718 | PV758284 | N/A | N/A |
| Fujian | Site 3 | 3/3 | 12_Pinus massoniana Leaf | (2) | CSFF 27976 | T11_B28_N/A_N/A | N/A | TB genotype 3 | *Ca. aconidialis* | PV756719 | PV758285 | N/A | N/A |
| Fujian | Site 3 | 3/3 | 12_Pinus massoniana Leaf | (2) | CSFF 27977 | T11_B28_N/A_N/A | N/A | TB genotype 3 | *Ca. aconidialis* | PV756720 | PV758286 | N/A | N/A |
| Fujian | Site 3 | 3/3 | 12_Pinus massoniana Leaf | (3) | CSFF 27978 | T11_B28_N/A_N/A | N/A | TB genotype 3 | *Ca. aconidialis* | PV756721 | PV758287 | N/A | N/A |
| Fujian | Site 3 | 3/3 | 12_Pinus massoniana Leaf | (3) | CSFF 27979 | T11_B28_N/A_N/A | N/A | TB genotype 3 | *Ca. aconidialis* | PV756722 | PV758288 | N/A | N/A |
| Fujian | Site 3 | 3/3 | 12_Pinus massoniana Leaf | (4) | CSFF 27980 | T11_B26_N/A_N/A | N/A | TB genotype 2 | *Ca. aconidialis* | PV756723 | PV758289 | N/A | N/A |
| Fujian | Site 3 | 3/3 | 12_Pinus massoniana Leaf | (4) | CSFF 27981 | T11_B26_N/A_N/A | N/A | TB genotype 2 | *Ca. aconidialis* | PV756724 | PV758290 | N/A | N/A |
| Fujian | Site 3 | 3/3 | 13_Eucalyptus urophylla hybrid Leaf | (1) | CSFF 28004 | T11_B25_H11_C20 | TBHC genotype 1 | TB genotype 1 | *Ca. aconidialis* | PV756747 | PV758313 | PV757511 | PV757653 |
| Fujian | Site 3 | 3/3 | 13_Eucalyptus urophylla hybrid Leaf | (1) | CSFF 28005 | T11_B25_N/A_N/A | N/A | TB genotype 1 | *Ca. aconidialis* | PV756748 | PV758314 | N/A | N/A |
| Fujian | Site 3 | 3/3 | 13_Eucalyptus urophylla hybrid Leaf | (2) | CSFF 28006 | T11_B25_N/A_N/A | N/A | TB genotype 1 | *Ca. aconidialis* | PV756749 | PV758315 | N/A | N/A |
| Fujian | Site 3 | 3/3 | 13_Eucalyptus urophylla hybrid Leaf | (2) | CSFF 28007 | T11_B25_N/A_N/A | N/A | TB genotype 1 | *Ca. aconidialis* | PV756750 | PV758316 | N/A | N/A |
| Fujian | Site 3 | 3/3 | 13_Eucalyptus urophylla hybrid Leaf | (3) | CSFF 28008 | T11_B25_N/A_N/A | N/A | TB genotype 1 | *Ca. aconidialis* | PV756751 | PV758317 | N/A | N/A |
| Fujian | Site 3 | 3/3 | 13_Eucalyptus urophylla hybrid Leaf | (3) | CSFF 28009 | T11_B25_N/A_N/A | N/A | TB genotype 1 | *Ca. aconidialis* | PV756752 | PV758318 | N/A | N/A |
| Fujian | Site 3 | 3/3 | 13_Eucalyptus urophylla hybrid Leaf | (4) | CSFF 28010 | T11_B25_N/A_N/A | N/A | TB genotype 1 | *Ca. aconidialis* | PV756753 | PV758319 | N/A | N/A |
| Fujian | Site 3 | 3/3 | 13_Eucalyptus urophylla hybrid Leaf | (4) | CSFF 28011 | T11_B25_N/A_N/A | N/A | TB genotype 1 | *Ca. aconidialis* | PV756754 | PV758320 | N/A | N/A |
| Fujian | Site 3 | 3/3 | 13_Eucalyptus urophylla hybrid Leaf | (5) | CSFF 28012 | T11_B25_N/A_N/A | N/A | TB genotype 1 | *Ca. aconidialis* | PV756755 | PV758321 | N/A | N/A |
| Fujian | Site 3 | 3/3 | 13_Eucalyptus urophylla hybrid Leaf | (5) | CSFF 28013 | T11_B25_N/A_N/A | N/A | TB genotype 1 | *Ca. aconidialis* | PV756756 | PV758322 | N/A | N/A |
| Fujian | Site 3 | 3/3 | 14_Syzygium jambos Leaf | (1) | CSFF 27948 | T11_B25_N/A_N/A | N/A | TB genotype 1 | *Ca. aconidialis* | PV756691 | PV758257 | N/A | N/A |
| Fujian | Site 3 | 3/3 | 14_Syzygium jambos Leaf | (1) | CSFF 27949 | T11_B25_N/A_N/A | N/A | TB genotype 1 | *Ca. aconidialis* | PV756692 | PV758258 | N/A | N/A |
| Fujian | Site 3 | 3/3 | 14_Syzygium jambos Leaf | (2) | CSFF 27950 | T11_B25_N/A_N/A | N/A | TB genotype 1 | *Ca. aconidialis* | PV756693 | PV758259 | N/A | N/A |
| Fujian | Site 3 | 3/3 | 14_Syzygium jambos Leaf | (2) | CSFF 27951 | T11_B25_N/A_N/A | N/A | TB genotype 1 | *Ca. aconidialis* | PV756694 | PV758260 | N/A | N/A |
| Fujian | Site 3 | 3/3 | 14_Syzygium jambos Leaf | (3) | CSFF 27952 | T11_B25_N/A_N/A | N/A | TB genotype 1 | *Ca. aconidialis* | PV756695 | PV758261 | N/A | N/A |
| Fujian | Site 3 | 3/3 | 14_Syzygium jambos Leaf | (3) | CSFF 27953 | T11_B25_N/A_N/A | N/A | TB genotype 1 | *Ca. aconidialis* | PV756696 | PV758262 | N/A | N/A |
| Fujian | Site 3 | 3/3 | 14_Syzygium jambos Leaf | (4) | CSFF 27954 | T11_B25_N/A_N/A | N/A | TB genotype 1 | *Ca. aconidialis* | PV756697 | PV758263 | N/A | N/A |
| Fujian | Site 3 | 3/3 | 14_Syzygium jambos Leaf | (4) | CSFF 27955 | T11_B25_N/A_N/A | N/A | TB genotype 1 | *Ca. aconidialis* | PV756698 | PV758264 | N/A | N/A |
| Fujian | Site 3 | 3/3 | 14_Syzygium jambos Leaf | (5) | CSFF 27956 | T11_B28_H11_C20 | TBHC genotype 3 | TB genotype 3 | *Ca. aconidialis* | PV756699 | PV758265 | PV757510 | PV757652 |
| Fujian | Site 3 | 3/3 | 14_Syzygium jambos Leaf | (5) | CSFF 27957 | T11_B25_N/A_N/A | N/A | TB genotype 1 | *Ca. aconidialis* | PV756700 | PV758266 | N/A | N/A |
| Fujian | Site 3 | 3/3 | 15_Pennisetum purpureum Leaf | (1) | CSFF 28014 | T11_B25_N/A_N/A | N/A | TB genotype 1 | *Ca. aconidialis* | PV756757 | PV758323 | N/A | N/A |
| Fujian | Site 3 | 3/3 | 15_Pennisetum purpureum Leaf | (1) | CSFF 28015 | T11_B28_N/A_N/A | N/A | TB genotype 3 | *Ca. aconidialis* | PV756758 | PV758324 | N/A | N/A |
| Fujian | Site 3 | 3/3 | 15_Pennisetum purpureum Leaf | (2) | CSFF 28016 | T11_B25_N/A_N/A | N/A | TB genotype 1 | *Ca. aconidialis* | PV756759 | PV758325 | N/A | N/A |
| Fujian | Site 3 | 3/3 | 15_Pennisetum purpureum Leaf | (2) | CSFF 28017 | T11_B25_N/A_N/A | N/A | TB genotype 1 | *Ca. aconidialis* | PV756760 | PV758326 | N/A | N/A |
| Fujian | Site 3 | 3/3 | 15_Pennisetum purpureum Leaf | (3) | CSFF 28018 | T11_B25_N/A_N/A | N/A | TB genotype 1 | *Ca. aconidialis* | PV756761 | PV758327 | N/A | N/A |
| Fujian | Site 3 | 3/3 | 15_Pennisetum purpureum Leaf | (3) | CSFF 28019 | T11_B25_N/A_N/A | N/A | TB genotype 1 | *Ca. aconidialis* | PV756762 | PV758328 | N/A | N/A |
| Fujian | Site 3 | 3/3 | 15_Pennisetum purpureum Leaf | (4) | CSFF 28020 | T11_B26_N/A_N/A | N/A | TB genotype 2 | *Ca. aconidialis* | PV756763 | PV758329 | N/A | N/A |
| Fujian | Site 3 | 3/3 | 15_Pennisetum purpureum Leaf | (4) | CSFF 28021 | T11_B25_N/A_N/A | N/A | TB genotype 1 | *Ca. aconidialis* | PV756764 | PV758330 | N/A | N/A |
| Fujian | Site 3 | 3/3 | 15_Pennisetum purpureum Leaf | (5) | CSFF 28022 | T11_B25_N/A_N/A | N/A | TB genotype 1 | *Ca. aconidialis* | PV756765 | PV758331 | N/A | N/A |
| Fujian | Site 3 | 3/3 | 15_Pennisetum purpureum Leaf | (5) | CSFF 28023 | T11_B26_N/A_N/A | N/A | TB genotype 2 | *Ca. aconidialis* | PV756766 | PV758332 | N/A | N/A |
| Fujian | Site 3 | 3/3 | 16_Saccharum officinarum Leaf | (1) | CSFF 27928 | T11_B25_N/A_N/A | N/A | TB genotype 1 | *Ca. aconidialis* | PV756671 | PV758237 | N/A | N/A |
| Fujian | Site 3 | 3/3 | 16_Saccharum officinarum Leaf | (1) | CSFF 27929 | T11_B26_H11_C20 | TBHC genotype 2 | TB genotype 2 | *Ca. aconidialis* | PV756672 | PV758238 | PV757509 | PV757651 |
| Fujian | Site 3 | 3/3 | 16_Saccharum officinarum Leaf | (2) | CSFF 27930 | T11_B28_N/A_N/A | N/A | TB genotype 3 | *Ca. aconidialis* | PV756673 | PV758239 | N/A | N/A |
| Fujian | Site 3 | 3/3 | 16_Saccharum officinarum Leaf | (2) | CSFF 27931 | T11_B25_N/A_N/A | N/A | TB genotype 1 | *Ca. aconidialis* | PV756674 | PV758240 | N/A | N/A |
| Fujian | Site 3 | 3/3 | 16_Saccharum officinarum Leaf | (3) | CSFF 27932 | T11_B28_N/A_N/A | N/A | TB genotype 3 | *Ca. aconidialis* | PV756675 | PV758241 | N/A | N/A |
| Fujian | Site 3 | 3/3 | 16_Saccharum officinarum Leaf | (3) | CSFF 27933 | T11_B28_N/A_N/A | N/A | TB genotype 3 | *Ca. aconidialis* | PV756676 | PV758242 | N/A | N/A |
| Fujian | Site 3 | 3/3 | 16_Saccharum officinarum Leaf | (4) | CSFF 27934 | T11_B25_N/A_N/A | N/A | TB genotype 1 | *Ca. aconidialis* | PV756677 | PV758243 | N/A | N/A |
| Fujian | Site 3 | 3/3 | 16_Saccharum officinarum Leaf | (4) | CSFF 27935 | T11_B26_N/A_N/A | N/A | TB genotype 2 | *Ca. aconidialis* | PV756678 | PV758244 | N/A | N/A |
| Fujian | Site 3 | 3/3 | 16_Saccharum officinarum Leaf | (5) | CSFF 27936 | T11_B28_N/A_N/A | N/A | TB genotype 3 | *Ca. aconidialis* | PV756679 | PV758245 | N/A | N/A |
| Fujian | Site 3 | 3/3 | 16_Saccharum officinarum Leaf | (5) | CSFF 27937 | T11_B28_N/A_N/A | N/A | TB genotype 3 | *Ca. aconidialis* | PV756680 | PV758246 | N/A | N/A |
| Fujian | Site 3 | 3/3 | 17_Rosa chinensis Leaf | (1) | CSFF 27898 | T11_B26_H11_C20 | TBHC genotype 2 | TB genotype 2 | *Ca. aconidialis* | PV756641 | PV758207 | PV757506 | PV757648 |
| Fujian | Site 3 | 3/3 | 17_Rosa chinensis Leaf | (1) | CSFF 27899 | T11_B28_N/A_N/A | N/A | TB genotype 3 | *Ca. aconidialis* | PV756642 | PV758208 | N/A | N/A |
| Fujian | Site 3 | 3/3 | 17_Rosa chinensis Leaf | (2) | CSFF 27900 | T11_B25_N/A_N/A | N/A | TB genotype 1 | *Ca. aconidialis* | PV756643 | PV758209 | N/A | N/A |
| Fujian | Site 3 | 3/3 | 17_Rosa chinensis Leaf | (2) | CSFF 27901 | T11_B25_N/A_N/A | N/A | TB genotype 1 | *Ca. aconidialis* | PV756644 | PV758210 | N/A | N/A |
| Fujian | Site 3 | 3/3 | 17_Rosa chinensis Leaf | (3) | CSFF 27902 | T11_B28_N/A_N/A | N/A | TB genotype 3 | *Ca. aconidialis* | PV756645 | PV758211 | N/A | N/A |
| Fujian | Site 3 | 3/3 | 17_Rosa chinensis Leaf | (3) | CSFF 27903 | T11_B28_N/A_N/A | N/A | TB genotype 3 | *Ca. aconidialis* | PV756646 | PV758212 | N/A | N/A |
| Fujian | Site 3 | 3/3 | 17_Rosa chinensis Leaf | (4) | CSFF 27904 | T11_B25_N/A_N/A | N/A | TB genotype 1 | *Ca. aconidialis* | PV756647 | PV758213 | N/A | N/A |
| Fujian | Site 3 | 3/3 | 17_Rosa chinensis Leaf | (4) | CSFF 27905 | T11_B25_N/A_N/A | N/A | TB genotype 1 | *Ca. aconidialis* | PV756648 | PV758214 | N/A | N/A |
| Fujian | Site 3 | 3/3 | 17_Rosa chinensis Leaf | (5) | CSFF 27906 | T11_B28_N/A_N/A | N/A | TB genotype 3 | *Ca. aconidialis* | PV756649 | PV758215 | N/A | N/A |
| Fujian | Site 3 | 3/3 | 17_Rosa chinensis Leaf | (5) | CSFF 27907 | T11_B28_N/A_N/A | N/A | TB genotype 3 | *Ca. aconidialis* | PV756650 | PV758216 | N/A | N/A |
| Fujian | Site 3 | 3/3 | 18_Acacia mangium Leaf | (1) | CSFF 27938 | T11_B25_N/A_N/A | N/A | TB genotype 1 | *Ca. aconidialis* | PV756681 | PV758247 | N/A | N/A |
| Fujian | Site 3 | 3/3 | 18_Acacia mangium Leaf | (1) | CSFF 27939 | T11_B25_N/A_N/A | N/A | TB genotype 1 | *Ca. aconidialis* | PV756682 | PV758248 | N/A | N/A |
| Fujian | Site 3 | 3/3 | 18_Acacia mangium Leaf | (2) | CSFF 27940 | T11_B25_N/A_N/A | N/A | TB genotype 1 | *Ca. aconidialis* | PV756683 | PV758249 | N/A | N/A |
| Fujian | Site 3 | 3/3 | 18_Acacia mangium Leaf | (2) | CSFF 27941 | T11_B25_N/A_N/A | N/A | TB genotype 1 | *Ca. aconidialis* | PV756684 | PV758250 | N/A | N/A |
| Fujian | Site 3 | 3/3 | 18_Acacia mangium Leaf | (3) | CSFF 27942 | T11_B25_N/A_N/A | N/A | TB genotype 1 | *Ca. aconidialis* | PV756685 | PV758251 | N/A | N/A |
| Fujian | Site 3 | 3/3 | 18_Acacia mangium Leaf | (3) | CSFF 27943 | T11_B25_N/A_N/A | N/A | TB genotype 1 | *Ca. aconidialis* | PV756686 | PV758252 | N/A | N/A |
| Fujian | Site 3 | 3/3 | 18_Acacia mangium Leaf | (4) | CSFF 27944 | T11_B28_N/A_N/A | N/A | TB genotype 3 | *Ca. aconidialis* | PV756687 | PV758253 | N/A | N/A |
| Fujian | Site 3 | 3/3 | 18_Acacia mangium Leaf | (4) | CSFF 27945 | T11_B28_N/A_N/A | N/A | TB genotype 3 | *Ca. aconidialis* | PV756688 | PV758254 | N/A | N/A |
| Fujian | Site 3 | 3/3 | 18_Acacia mangium Leaf | (5) | CSFF 27946 | T11_B25_N/A_N/A | N/A | TB genotype 1 | *Ca. aconidialis* | PV756689 | PV758255 | N/A | N/A |
| Fujian | Site 3 | 3/3 | 18_Acacia mangium Leaf | (5) | CSFF 27947 | T11_B25_N/A_N/A | N/A | TB genotype 1 | *Ca. aconidialis* | PV756690 | PV758256 | N/A | N/A |
| Fujian | Site 3 | 3/3 | 19_Dianthus caryophyllus Leaf | (1) | CSFF 27888 | T11_B25_H11_C20 | TBHC genotype 1 | TB genotype 1 | *Ca. aconidialis* | PV756631 | PV758197 | PV757504 | PV757646 |
| Fujian | Site 3 | 3/3 | 19_Dianthus caryophyllus Leaf | (1) | CSFF 27889 | T11_B25_N/A_N/A | N/A | TB genotype 1 | *Ca. aconidialis* | PV756632 | PV758198 | N/A | N/A |
| Fujian | Site 3 | 3/3 | 19_Dianthus caryophyllus Leaf | (2) | CSFF 27890 | T11_B25_N/A_N/A | N/A | TB genotype 1 | *Ca. aconidialis* | PV756633 | PV758199 | N/A | N/A |
| Fujian | Site 3 | 3/3 | 19_Dianthus caryophyllus Leaf | (2) | CSFF 27891 | T11_B28_H11_C20 | TBHC genotype 3 | TB genotype 3 | *Ca. aconidialis* | PV756634 | PV758200 | PV757505 | PV757647 |
| Fujian | Site 3 | 3/3 | 19_Dianthus caryophyllus Leaf | (3) | CSFF 27892 | T11_B25_N/A_N/A | N/A | TB genotype 1 | *Ca. aconidialis* | PV756635 | PV758201 | N/A | N/A |
| Fujian | Site 3 | 3/3 | 19_Dianthus caryophyllus Leaf | (3) | CSFF 27893 | T11_B25_N/A_N/A | N/A | TB genotype 1 | *Ca. aconidialis* | PV756636 | PV758202 | N/A | N/A |
| Fujian | Site 3 | 3/3 | 19_Dianthus caryophyllus Leaf | (4) | CSFF 27894 | T11_B25_N/A_N/A | N/A | TB genotype 1 | *Ca. aconidialis* | PV756637 | PV758203 | N/A | N/A |
| Fujian | Site 3 | 3/3 | 19_Dianthus caryophyllus Leaf | (4) | CSFF 27895 | T11_B25_N/A_N/A | N/A | TB genotype 1 | *Ca. aconidialis* | PV756638 | PV758204 | N/A | N/A |
| Fujian | Site 3 | 3/3 | 19_Dianthus caryophyllus Leaf | (5) | CSFF 27896 | T11_B25_N/A_N/A | N/A | TB genotype 1 | *Ca. aconidialis* | PV756639 | PV758205 | N/A | N/A |
| Fujian | Site 3 | 3/3 | 19_Dianthus caryophyllus Leaf | (5) | CSFF 27897 | T11_B25_N/A_N/A | N/A | TB genotype 1 | *Ca. aconidialis* | PV756640 | PV758206 | N/A | N/A |
| Fujian | Site 3 | 3/3 | 20_Cinnamomum camphora Leaf | (1) | CSFF 27908 | T11_B25_N/A_N/A | N/A | TB genotype 1 | *Ca. aconidialis* | PV756651 | PV758217 | N/A | N/A |
| Fujian | Site 3 | 3/3 | 20_Cinnamomum camphora Leaf | (1) | CSFF 27909 | T11_B25_N/A_N/A | N/A | TB genotype 1 | *Ca. aconidialis* | PV756652 | PV758218 | N/A | N/A |
| Fujian | Site 3 | 3/3 | 20_Cinnamomum camphora Leaf | (2) | CSFF 27910 | T11_B25_N/A_N/A | N/A | TB genotype 1 | *Ca. aconidialis* | PV756653 | PV758219 | N/A | N/A |
| Fujian | Site 3 | 3/3 | 20_Cinnamomum camphora Leaf | (2) | CSFF 27911 | T11_B25_N/A_N/A | N/A | TB genotype 1 | *Ca. aconidialis* | PV756654 | PV758220 | N/A | N/A |
| Fujian | Site 3 | 3/3 | 20_Cinnamomum camphora Leaf | (3) | CSFF 27912 | T11_B28_N/A_N/A | N/A | TB genotype 3 | *Ca. aconidialis* | PV756655 | PV758221 | N/A | N/A |
| Fujian | Site 3 | 3/3 | 20_Cinnamomum camphora Leaf | (3) | CSFF 27913 | T11_B25_N/A_N/A | N/A | TB genotype 1 | *Ca. aconidialis* | PV756656 | PV758222 | N/A | N/A |
| Fujian | Site 3 | 3/3 | 20_Cinnamomum camphora Leaf | (4) | CSFF 27914 | T21_B13_N/A_N/A | N/A | TB genotype 20 | *Ca. hongkongensis* | PV756657 | PV758223 | N/A | N/A |
| Fujian | Site 3 | 3/3 | 20_Cinnamomum camphora Leaf | (4) | CSFF 27915 | T21_B13_H17_C11 | TBHC genotype 23 | TB genotype 20 | *Ca. hongkongensis* | PV756658 | PV758224 | PV757507 | PV757649 |
| Fujian | Site 3 | 3/3 | 20_Cinnamomum camphora Leaf | (5) | CSFF 27916 | T15_B25_H11_C20 | TBHC genotype 8 | TB genotype 8 | *Ca. aconidialis* | PV756659 | PV758225 | PV757508 | PV757650 |
| Fujian | Site 3 | 3/3 | 20_Cinnamomum camphora Leaf | (5) | CSFF 27917 | T11_B25_N/A_N/A | N/A | TB genotype 1 | *Ca. aconidialis* | PV756660 | PV758226 | N/A | N/A |
| Fujian | Site 3 | 3/3 | 21_Washingtonia filifera Leaf | (1) | CSFF 27918 | T11_B25_N/A_N/A | N/A | TB genotype 1 | *Ca. aconidialis* | PV756661 | PV758227 | N/A | N/A |
| Fujian | Site 3 | 3/3 | 21_Washingtonia filifera Leaf | (1) | CSFF 27919 | T11_B25_N/A_N/A | N/A | TB genotype 1 | *Ca. aconidialis* | PV756662 | PV758228 | N/A | N/A |
| Fujian | Site 3 | 3/3 | 21_Washingtonia filifera Leaf | (2) | CSFF 27920 | T11_B25_N/A_N/A | N/A | TB genotype 1 | *Ca. aconidialis* | PV756663 | PV758229 | N/A | N/A |
| Fujian | Site 3 | 3/3 | 21_Washingtonia filifera Leaf | (2) | CSFF 27921 | T11_B25_N/A_N/A | N/A | TB genotype 1 | *Ca. aconidialis* | PV756664 | PV758230 | N/A | N/A |
| Fujian | Site 3 | 3/3 | 21_Washingtonia filifera Leaf | (3) | CSFF 27922 | T11_B25_N/A_N/A | N/A | TB genotype 1 | *Ca. aconidialis* | PV756665 | PV758231 | N/A | N/A |
| Fujian | Site 3 | 3/3 | 21_Washingtonia filifera Leaf | (3) | CSFF 27923 | T11_B25_N/A_N/A | N/A | TB genotype 1 | *Ca. aconidialis* | PV756666 | PV758232 | N/A | N/A |
| Fujian | Site 3 | 3/3 | 21_Washingtonia filifera Leaf | (4) | CSFF 27924 | T11_B28_N/A_N/A | N/A | TB genotype 3 | *Ca. aconidialis* | PV756667 | PV758233 | N/A | N/A |
| Fujian | Site 3 | 3/3 | 21_Washingtonia filifera Leaf | (4) | CSFF 27925 | T11_B25_N/A_N/A | N/A | TB genotype 1 | *Ca. aconidialis* | PV756668 | PV758234 | N/A | N/A |
| Fujian | Site 3 | 3/3 | 21_Washingtonia filifera Leaf | (5) | CSFF 27926 | T11_B25_N/A_N/A | N/A | TB genotype 1 | *Ca. aconidialis* | PV756669 | PV758235 | N/A | N/A |
| Fujian | Site 3 | 3/3 | 21_Washingtonia filifera Leaf | (5) | CSFF 27927 | T11_B25_N/A_N/A | N/A | TB genotype 1 | *Ca. aconidialis* | PV756670 | PV758236 | N/A | N/A |
| Fujian | Site 3 | 3/3 | 22_Ficus benjamina Leaf | (1) | CSFF 27958 | T11_B25_N/A_N/A | N/A | TB genotype 1 | *Ca. aconidialis* | PV756701 | PV758267 | N/A | N/A |
| Fujian | Site 3 | 3/3 | 22_Ficus benjamina Leaf | (1) | CSFF 27959 | T11_B25_N/A_N/A | N/A | TB genotype 1 | *Ca. aconidialis* | PV756702 | PV758268 | N/A | N/A |
| Fujian | Site 3 | 3/3 | 22_Ficus benjamina Leaf | (2) | CSFF 27960 | T11_B28_N/A_N/A | N/A | TB genotype 3 | *Ca. aconidialis* | PV756703 | PV758269 | N/A | N/A |
| Fujian | Site 3 | 3/3 | 22_Ficus benjamina Leaf | (2) | CSFF 27961 | T11_B26_N/A_N/A | N/A | TB genotype 2 | *Ca. aconidialis* | PV756704 | PV758270 | N/A | N/A |
| Fujian | Site 3 | 3/3 | 22_Ficus benjamina Leaf | (3) | CSFF 27962 | T11_B25_N/A_N/A | N/A | TB genotype 1 | *Ca. aconidialis* | PV756705 | PV758271 | N/A | N/A |
| Fujian | Site 3 | 3/3 | 22_Ficus benjamina Leaf | (3) | CSFF 27963 | T11_B28_N/A_N/A | N/A | TB genotype 3 | *Ca. aconidialis* | PV756706 | PV758272 | N/A | N/A |
| Fujian | Site 3 | 3/3 | 22_Ficus benjamina Leaf | (4) | CSFF 27964 | T11_B25_N/A_N/A | N/A | TB genotype 1 | *Ca. aconidialis* | PV756707 | PV758273 | N/A | N/A |
| Fujian | Site 3 | 3/3 | 22_Ficus benjamina Leaf | (4) | CSFF 27965 | T11_B25_N/A_N/A | N/A | TB genotype 1 | *Ca. aconidialis* | PV756708 | PV758274 | N/A | N/A |
| Fujian | Site 3 | 3/3 | 22_Ficus benjamina Leaf | (5) | CSFF 27966 | T11_B25_N/A_N/A | N/A | TB genotype 1 | *Ca. aconidialis* | PV756709 | PV758275 | N/A | N/A |
| Fujian | Site 3 | 3/3 | 22_Ficus benjamina Leaf | (5) | CSFF 27967 | T11_B25_N/A_N/A | N/A | TB genotype 1 | *Ca. aconidialis* | PV756710 | PV758276 | N/A | N/A |
| Fujian | Site 3 | 3/3 | 23_Rhododendron pulchrum Leaf | (1) | CSFF 27982 | T11_B25_N/A_N/A | N/A | TB genotype 1 | *Ca. aconidialis* | PV756725 | PV758291 | N/A | N/A |
| Fujian | Site 3 | 3/3 | 23_Rhododendron pulchrum Leaf | (1) | CSFF 27983 | T11_B25_N/A_N/A | N/A | TB genotype 1 | *Ca. aconidialis* | PV756726 | PV758292 | N/A | N/A |
| Fujian | Site 3 | 3/3 | 23_Rhododendron pulchrum Leaf | (2) | CSFF 27984 | T11_B25_N/A_N/A | N/A | TB genotype 1 | *Ca. aconidialis* | PV756727 | PV758293 | N/A | N/A |
| Fujian | Site 3 | 3/3 | 23_Rhododendron pulchrum Leaf | (2) | CSFF 27985 | T11_B25_N/A_N/A | N/A | TB genotype 1 | *Ca. aconidialis* | PV756728 | PV758294 | N/A | N/A |
| Fujian | Site 3 | 3/3 | 23_Rhododendron pulchrum Leaf | (3) | CSFF 27986 | T11_B25_N/A_N/A | N/A | TB genotype 1 | *Ca. aconidialis* | PV756729 | PV758295 | N/A | N/A |
| Fujian | Site 3 | 3/3 | 23_Rhododendron pulchrum Leaf | (3) | CSFF 27987 | T11_B25_N/A_N/A | N/A | TB genotype 1 | *Ca. aconidialis* | PV756730 | PV758296 | N/A | N/A |
| Fujian | Site 3 | 3/3 | 23_Rhododendron pulchrum Leaf | (4) | CSFF 27988 | T11_B25_N/A_N/A | N/A | TB genotype 1 | *Ca. aconidialis* | PV756731 | PV758297 | N/A | N/A |
| Fujian | Site 3 | 3/3 | 23_Rhododendron pulchrum Leaf | (4) | CSFF 27989 | T11_B25_N/A_N/A | N/A | TB genotype 1 | *Ca. aconidialis* | PV756732 | PV758298 | N/A | N/A |
| Fujian | Site 3 | 3/3 | 23_Rhododendron pulchrum Leaf | (5) | CSFF 27990 | T11_B25_N/A_N/A | N/A | TB genotype 1 | *Ca. aconidialis* | PV756733 | PV758299 | N/A | N/A |
| Fujian | Site 3 | 3/3 | 23_Rhododendron pulchrum Leaf | (5) | CSFF 27991 | T11_B25_N/A_N/A | N/A | TB genotype 1 | *Ca. aconidialis* | PV756734 | PV758300 | N/A | N/A |
| Fujian | Site 3 | 3/3 | 24_Glycine max Stem | (1) | CSFF 27996 | T11_B25_N/A_N/A | N/A | TB genotype 1 | *Ca. aconidialis* | PV756739 | PV758305 | N/A | N/A |
| Fujian | Site 3 | 3/3 | 24_Glycine max Stem | (1) | CSFF 27997 | T11_B25_N/A_N/A | N/A | TB genotype 1 | *Ca. aconidialis* | PV756740 | PV758306 | N/A | N/A |
| Fujian | Site 3 | 3/3 | 24_Glycine max Stem | (2) | CSFF 27998 | T11_B26_N/A_N/A | N/A | TB genotype 2 | *Ca. aconidialis* | PV756741 | PV758307 | N/A | N/A |
| Fujian | Site 3 | 3/3 | 24_Glycine max Stem | (2) | CSFF 27999 | T11_B25_N/A_N/A | N/A | TB genotype 1 | *Ca. aconidialis* | PV756742 | PV758308 | N/A | N/A |
| Fujian | Site 3 | 3/3 | 24_Glycine max Stem | (3) | CSFF 28000 | T11_B25_N/A_N/A | N/A | TB genotype 1 | *Ca. aconidialis* | PV756743 | PV758309 | N/A | N/A |
| Fujian | Site 3 | 3/3 | 24_Glycine max Stem | (3) | CSFF 28001 | T11_B25_N/A_N/A | N/A | TB genotype 1 | *Ca. aconidialis* | PV756744 | PV758310 | N/A | N/A |
| Fujian | Site 3 | 3/3 | 24_Glycine max Stem | (5) | CSFF 28002 | T11_B25_N/A_N/A | N/A | TB genotype 1 | *Ca. aconidialis* | PV756745 | PV758311 | N/A | N/A |
| Fujian | Site 3 | 3/3 | 24_Glycine max Stem | (5) | CSFF 28003 | T11_B25_N/A_N/A | N/A | TB genotype 1 | *Ca. aconidialis* | PV756746 | PV758312 | N/A | N/A |
| Fujian | Site 3 | 3/3 | 26_Medicago sativa Seed | (1) | CSFF 28024 | T11_B25_H11_C20 | TBHC genotype 1 | TB genotype 1 | *Ca. aconidialis* | PV756767 | PV758333 | PV757512 | PV757654 |
| Fujian | Site 3 | 3/3 | 26_Medicago sativa Seed | (1) | CSFF 28025 | T11_B28_H11_C20 | TBHC genotype 3 | TB genotype 3 | *Ca. aconidialis* | PV756768 | PV758334 | PV757513 | PV757655 |
| Fujian | Site 3 | 3/3 | 26_Medicago sativa Seed | (2) | CSFF 28026 | T11_B25_N/A_N/A | N/A | TB genotype 1 | *Ca. aconidialis* | PV756769 | PV758335 | N/A | N/A |
| Fujian | Site 3 | 3/3 | 26_Medicago sativa Seed | (2) | CSFF 28027 | T11_B28_N/A_N/A | N/A | TB genotype 3 | *Ca. aconidialis* | PV756770 | PV758336 | N/A | N/A |
| Fujian | Site 3 | 3/3 | 26_Medicago sativa Seed | (3) | CSFF 28028 | T11_B25_N/A_N/A | N/A | TB genotype 1 | *Ca. aconidialis* | PV756771 | PV758337 | N/A | N/A |
| Fujian | Site 3 | 3/3 | 26_Medicago sativa Seed | (3) | CSFF 28029 | T11_B28_N/A_N/A | N/A | TB genotype 3 | *Ca. aconidialis* | PV756772 | PV758338 | N/A | N/A |
| Fujian | Site 3 | 3/3 | 26_Medicago sativa Seed | (4) | CSFF 28030 | T11_B28_N/A_N/A | N/A | TB genotype 3 | *Ca. aconidialis* | PV756773 | PV758339 | N/A | N/A |
| Fujian | Site 3 | 3/3 | 26_Medicago sativa Seed | (4) | CSFF 28031 | T11_B25_N/A_N/A | N/A | TB genotype 1 | *Ca. aconidialis* | PV756774 | PV758340 | N/A | N/A |
| Fujian | Site 3 | 3/3 | 26_Medicago sativa Seed | (5) | CSFF 28032 | T11_B28_N/A_N/A | N/A | TB genotype 3 | *Ca. aconidialis* | PV756775 | PV758341 | N/A | N/A |
| Fujian | Site 3 | 3/3 | 26_Medicago sativa Seed | (5) | CSFF 28033 | T11_B28_N/A_N/A | N/A | TB genotype 3 | *Ca. aconidialis* | PV756776 | PV758342 | N/A | N/A |
| Hainan | Site 4 | 2/1 | 2_Zinnia elegans Seed | (1) | CSFF 28058 | T22_B11_N/A_N/A | N/A | TB genotype 23 | *Ca. hongkongensis* | PV756801 | PV758367 | N/A | N/A |
| Hainan | Site 4 | 2/1 | 2_Zinnia elegans Seed | (1) | CSFF 28059 | T22_B11_N/A_N/A | N/A | TB genotype 23 | *Ca. hongkongensis* | PV756802 | PV758368 | N/A | N/A |
| Hainan | Site 4 | 2/1 | 2_Zinnia elegans Seed | (4) | CSFF 28060 | T22_B11_N/A_N/A | N/A | TB genotype 23 | *Ca. hongkongensis* | PV756803 | PV758369 | N/A | N/A |
| Hainan | Site 4 | 2/1 | 2_Zinnia elegans Seed | (4) | CSFF 28061 | T22_B11_N/A_N/A | N/A | TB genotype 23 | *Ca. hongkongensis* | PV756804 | PV758370 | N/A | N/A |
| Hainan | Site 4 | 2/1 | 3_Lolium perenne Seed | (2) | CSFF 28064 | T21_B13_N/A_N/A | N/A | TB genotype 20 | *Ca. hongkongensis* | PV756807 | PV758373 | N/A | N/A |
| Hainan | Site 4 | 2/1 | 3_Lolium perenne Seed | (2) | CSFF 28065 | T21_B13_N/A_N/A | N/A | TB genotype 20 | *Ca. hongkongensis* | PV756808 | PV758374 | N/A | N/A |
| Hainan | Site 4 | 2/1 | 5_Impatiens balsamina Seed | (4) | CSFF 28062 | T21_B13_N/A_N/A | N/A | TB genotype 20 | *Ca. hongkongensis* | PV756805 | PV758371 | N/A | N/A |
| Hainan | Site 4 | 2/1 | 5_Impatiens balsamina Seed | (4) | CSFF 28063 | T21_B13_N/A_N/A | N/A | TB genotype 20 | *Ca. hongkongensis* | PV756806 | PV758372 | N/A | N/A |
| Hainan | Site 4 | 2/1 | 7_Dianthus caryophyllus Seed | (1) | CSFF 28054 | T22_B11_N/A_N/A | N/A | TB genotype 23 | *Ca. hongkongensis* | PV756797 | PV758363 | N/A | N/A |
| Hainan | Site 4 | 2/1 | 7_Dianthus caryophyllus Seed | (1) | CSFF 28055 | T22_B11_N/A_N/A | N/A | TB genotype 23 | *Ca. hongkongensis* | PV756798 | PV758364 | N/A | N/A |
| Hainan | Site 4 | 2/1 | 7_Dianthus caryophyllus Seed | (4) | CSFF 28056 | T22_B11_N/A_N/A | N/A | TB genotype 23 | *Ca. hongkongensis* | PV756799 | PV758365 | N/A | N/A |
| Hainan | Site 4 | 2/1 | 7_Dianthus caryophyllus Seed | (4) | CSFF 28057 | T22_B11_N/A_N/A | N/A | TB genotype 23 | *Ca. hongkongensis* | PV756800 | PV758366 | N/A | N/A |
| Hainan | Site 4 | 2/1 | 8_Trifolium pratense Seed | (1) | CSFF 28044 | T22_B11_N/A_N/A | N/A | TB genotype 23 | *Ca. hongkongensis* | PV756787 | PV758353 | N/A | N/A |
| Hainan | Site 4 | 2/1 | 8_Trifolium pratense Seed | (1) | CSFF 28045 | T22_B11_N/A_N/A | N/A | TB genotype 23 | *Ca. hongkongensis* | PV756788 | PV758354 | N/A | N/A |
| Hainan | Site 4 | 2/1 | 8_Trifolium pratense Seed | (2) | CSFF 28046 | T22_B11_N/A_N/A | N/A | TB genotype 23 | *Ca. hongkongensis* | PV756789 | PV758355 | N/A | N/A |
| Hainan | Site 4 | 2/1 | 8_Trifolium pratense Seed | (2) | CSFF 28047 | T22_B11_N/A_N/A | N/A | TB genotype 23 | *Ca. hongkongensis* | PV756790 | PV758356 | N/A | N/A |
| Hainan | Site 4 | 2/1 | 8_Trifolium pratense Seed | (3) | CSFF 28048 | T22_B11_N/A_N/A | N/A | TB genotype 23 | *Ca. hongkongensis* | PV756791 | PV758357 | N/A | N/A |
| Hainan | Site 4 | 2/1 | 8_Trifolium pratense Seed | (3) | CSFF 28049 | T22_B11_N/A_N/A | N/A | TB genotype 23 | *Ca. hongkongensis* | PV756792 | PV758358 | N/A | N/A |
| Hainan | Site 4 | 2/1 | 8_Trifolium pratense Seed | (4) | CSFF 28050 | T21_B13_H17_C11 | TBHC genotype 23 | TB genotype 20 | *Ca. hongkongensis* | PV756793 | PV758359 | PV757517 | PV757659 |
| Hainan | Site 4 | 2/1 | 8_Trifolium pratense Seed | (4) | CSFF 28051 | T21_B13_N/A_N/A | N/A | TB genotype 20 | *Ca. hongkongensis* | PV756794 | PV758360 | N/A | N/A |
| Hainan | Site 4 | 2/1 | 8_Trifolium pratense Seed | (5) | CSFF 28052 | T22_B11_N/A_N/A | N/A | TB genotype 23 | *Ca. hongkongensis* | PV756795 | PV758361 | N/A | N/A |
| Hainan | Site 4 | 2/1 | 8_Trifolium pratense Seed | (5) | CSFF 28053 | T22_B11_N/A_N/A | N/A | TB genotype 23 | *Ca. hongkongensis* | PV756796 | PV758362 | N/A | N/A |
| Hainan | Site 4 | 2/1 | 9_Lythrum salicaria Seed | (1) | CSFF 28034 | T22_B11_H17_C11 | TBHC genotype 26 | TB genotype 23 | *Ca. hongkongensis* | PV756777 | PV758343 | PV757514 | PV757656 |
| Hainan | Site 4 | 2/1 | 9_Lythrum salicaria Seed | (1) | CSFF 28035 | T22_B11_N/A_N/A | N/A | TB genotype 23 | *Ca. hongkongensis* | PV756778 | PV758344 | N/A | N/A |
| Hainan | Site 4 | 2/1 | 9_Lythrum salicaria Seed | (2) | CSFF 28036 | T22_B11_N/A_N/A | N/A | TB genotype 23 | *Ca. hongkongensis* | PV756779 | PV758345 | N/A | N/A |
| Hainan | Site 4 | 2/1 | 9_Lythrum salicaria Seed | (2) | CSFF 28037 | T22_B11_N/A_N/A | N/A | TB genotype 23 | *Ca. hongkongensis* | PV756780 | PV758346 | N/A | N/A |
| Hainan | Site 4 | 2/1 | 9_Lythrum salicaria Seed | (3) | CSFF 28038 | T22_B11_N/A_N/A | N/A | TB genotype 23 | *Ca. hongkongensis* | PV756781 | PV758347 | N/A | N/A |
| Hainan | Site 4 | 2/1 | 9_Lythrum salicaria Seed | (3) | CSFF 28039 | T16_B34_H23_C23 | TBHC genotype 9 | TB genotype 9 | *Ca. pseudoreteaudii* | PV756782 | PV758348 | PV757515 | PV757657 |
| Hainan | Site 4 | 2/1 | 9_Lythrum salicaria Seed | (4) | CSFF 28040 | T22_B11_N/A_N/A | N/A | TB genotype 23 | *Ca. hongkongensis* | PV756783 | PV758349 | N/A | N/A |
| Hainan | Site 4 | 2/1 | 9_Lythrum salicaria Seed | (4) | CSFF 28041 | T22_B11_N/A_N/A | N/A | TB genotype 23 | *Ca. hongkongensis* | PV756784 | PV758350 | N/A | N/A |
| Hainan | Site 4 | 2/1 | 9_Lythrum salicaria Seed | (5) | CSFF 28042 | T21_B12_H17_C12 | TBHC genotype 22 | TB genotype 19 | *Ca. hongkongensis* | PV756785 | PV758351 | PV757516 | PV757658 |
| Hainan | Site 4 | 2/1 | 9_Lythrum salicaria Seed | (5) | CSFF 28043 | T21_B12_N/A_N/A | N/A | TB genotype 19 | *Ca. hongkongensis* | PV756786 | PV758352 | N/A | N/A |
| Hainan | Site 4 | 2/1 | 10_Buxus sinica Leaf | (2) | CSFF 28152 | T22_B11_N/A_N/A | N/A | TB genotype 23 | *Ca. hongkongensis* | PV756895 | PV758461 | N/A | N/A |
| Hainan | Site 4 | 2/1 | 10_Buxus sinica Leaf | (2) | CSFF 28153 | T22_B11_N/A_N/A | N/A | TB genotype 23 | *Ca. hongkongensis* | PV756896 | PV758462 | N/A | N/A |
| Hainan | Site 4 | 2/1 | 11_Imperata cylindrica Leaf | (5) | CSFF 28134 | T22_B11_N/A_N/A | N/A | TB genotype 23 | *Ca. hongkongensis* | PV756877 | PV758443 | N/A | N/A |
| Hainan | Site 4 | 2/1 | 11_Imperata cylindrica Leaf | (5) | CSFF 28135 | T22_B11_N/A_N/A | N/A | TB genotype 23 | *Ca. hongkongensis* | PV756878 | PV758444 | N/A | N/A |
| Hainan | Site 4 | 2/1 | 12_Pinus massoniana Leaf | (1) | CSFF 28136 | T22_B11_N/A_N/A | N/A | TB genotype 23 | *Ca. hongkongensis* | PV756879 | PV758445 | N/A | N/A |
| Hainan | Site 4 | 2/1 | 12_Pinus massoniana Leaf | (1) | CSFF 28137 | T22_B11_N/A_N/A | N/A | TB genotype 23 | *Ca. hongkongensis* | PV756880 | PV758446 | N/A | N/A |
| Hainan | Site 4 | 2/1 | 12_Pinus massoniana Leaf | (2) | CSFF 28138 | T21_B13_N/A_N/A | N/A | TB genotype 20 | *Ca. hongkongensis* | PV756881 | PV758447 | N/A | N/A |
| Hainan | Site 4 | 2/1 | 12_Pinus massoniana Leaf | (2) | CSFF 28139 | T21_B13_N/A_N/A | N/A | TB genotype 20 | *Ca. hongkongensis* | PV756882 | PV758448 | N/A | N/A |
| Hainan | Site 4 | 2/1 | 12_Pinus massoniana Leaf | (4) | CSFF 28140 | T22_B11_N/A_N/A | N/A | TB genotype 23 | *Ca. hongkongensis* | PV756883 | PV758449 | N/A | N/A |
| Hainan | Site 4 | 2/1 | 12_Pinus massoniana Leaf | (4) | CSFF 28141 | T22_B11_N/A_N/A | N/A | TB genotype 23 | *Ca. hongkongensis* | PV756884 | PV758450 | N/A | N/A |
| Hainan | Site 4 | 2/1 | 13_Eucalyptus urophylla hybrid Leaf | (1) | CSFF 28154 | T16_B34_H23_C23 | TBHC genotype 9 | TB genotype 9 | *Ca. pseudoreteaudii* | PV756897 | PV758463 | PV757526 | PV757668 |
| Hainan | Site 4 | 2/1 | 13_Eucalyptus urophylla hybrid Leaf | (1) | CSFF 28155 | T16_B34_N/A_N/A | N/A | TB genotype 9 | *Ca. pseudoreteaudii* | PV756898 | PV758464 | N/A | N/A |
| Hainan | Site 4 | 2/1 | 13_Eucalyptus urophylla hybrid Leaf | (2) | CSFF 28156 | T16_B34_N/A_N/A | N/A | TB genotype 9 | *Ca. pseudoreteaudii* | PV756899 | PV758465 | N/A | N/A |
| Hainan | Site 4 | 2/1 | 13_Eucalyptus urophylla hybrid Leaf | (2) | CSFF 28157 | T21_B13_N/A_N/A | N/A | TB genotype 20 | *Ca. hongkongensis* | PV756900 | PV758466 | N/A | N/A |
| Hainan | Site 4 | 2/1 | 13_Eucalyptus urophylla hybrid Leaf | (3) | CSFF 28158 | T17_B34_H23_C23 | TBHC genotype 10 | TB genotype 10 | *Ca. pseudoreteaudii* | PV756901 | PV758467 | PV757527 | PV757669 |
| Hainan | Site 4 | 2/1 | 13_Eucalyptus urophylla hybrid Leaf | (3) | CSFF 28159 | T22_B11_H17_C11 | TBHC genotype 26 | TB genotype 23 | *Ca. hongkongensis* | PV756902 | PV758468 | PV757528 | PV757670 |
| Hainan | Site 4 | 2/1 | 13_Eucalyptus urophylla hybrid Leaf | (4) | CSFF 28160 | T22_B11_N/A_N/A | N/A | TB genotype 23 | *Ca. hongkongensis* | PV756903 | PV758469 | N/A | N/A |
| Hainan | Site 4 | 2/1 | 13_Eucalyptus urophylla hybrid Leaf | (4) | CSFF 28161 | T21_B15_H17_C11 | TBHC genotype 25 | TB genotype 22 | *Ca. hongkongensis* | PV756904 | PV758470 | PV757529 | PV757671 |
| Hainan | Site 4 | 2/1 | 13_Eucalyptus urophylla hybrid Leaf | (5) | CSFF 28162 | T22_B11_N/A_N/A | N/A | TB genotype 23 | *Ca. hongkongensis* | PV756905 | PV758471 | N/A | N/A |
| Hainan | Site 4 | 2/1 | 13_Eucalyptus urophylla hybrid Leaf | (5) | CSFF 28163 | T22_B11_N/A_N/A | N/A | TB genotype 23 | *Ca. hongkongensis* | PV756906 | PV758472 | N/A | N/A |
| Hainan | Site 4 | 2/1 | 14_Syzygium jambos Leaf | (4) | CSFF 28120 | T22_B11_N/A_N/A | N/A | TB genotype 23 | *Ca. hongkongensis* | PV756863 | PV758429 | N/A | N/A |
| Hainan | Site 4 | 2/1 | 14_Syzygium jambos Leaf | (4) | CSFF 28121 | T22_B11_N/A_N/A | N/A | TB genotype 23 | *Ca. hongkongensis* | PV756864 | PV758430 | N/A | N/A |
| Hainan | Site 4 | 2/1 | 14_Syzygium jambos Leaf | (5) | CSFF 28122 | T22_B11_N/A_N/A | N/A | TB genotype 23 | *Ca. hongkongensis* | PV756865 | PV758431 | N/A | N/A |
| Hainan | Site 4 | 2/1 | 14_Syzygium jambos Leaf | (5) | CSFF 28123 | T22_B11_N/A_N/A | N/A | TB genotype 23 | *Ca. hongkongensis* | PV756866 | PV758432 | N/A | N/A |
| Hainan | Site 4 | 2/1 | 15_Pennisetum purpureum Leaf | (1) | CSFF 28164 | T22_B11_N/A_N/A | N/A | TB genotype 23 | *Ca. hongkongensis* | PV756907 | PV758473 | N/A | N/A |
| Hainan | Site 4 | 2/1 | 15_Pennisetum purpureum Leaf | (1) | CSFF 28165 | T22_B11_N/A_N/A | N/A | TB genotype 23 | *Ca. hongkongensis* | PV756908 | PV758474 | N/A | N/A |
| Hainan | Site 4 | 2/1 | 15_Pennisetum purpureum Leaf | (2) | CSFF 28166 | T22_B11_N/A_N/A | N/A | TB genotype 23 | *Ca. hongkongensis* | PV756909 | PV758475 | N/A | N/A |
| Hainan | Site 4 | 2/1 | 15_Pennisetum purpureum Leaf | (2) | CSFF 28167 | T22_B11_N/A_N/A | N/A | TB genotype 23 | *Ca. hongkongensis* | PV756910 | PV758476 | N/A | N/A |
| Hainan | Site 4 | 2/1 | 15_Pennisetum purpureum Leaf | (5) | CSFF 28168 | T22_B11_N/A_N/A | N/A | TB genotype 23 | *Ca. hongkongensis* | PV756911 | PV758477 | N/A | N/A |
| Hainan | Site 4 | 2/1 | 15_Pennisetum purpureum Leaf | (5) | CSFF 28169 | T22_B11_N/A_N/A | N/A | TB genotype 23 | *Ca. hongkongensis* | PV756912 | PV758478 | N/A | N/A |
| Hainan | Site 4 | 2/1 | 16_Saccharum officinarum Leaf | (1) | CSFF 28100 | T22_B11_N/A_N/A | N/A | TB genotype 23 | *Ca. hongkongensis* | PV756843 | PV758409 | N/A | N/A |
| Hainan | Site 4 | 2/1 | 16_Saccharum officinarum Leaf | (1) | CSFF 28101 | T22_B11_N/A_N/A | N/A | TB genotype 23 | *Ca. hongkongensis* | PV756844 | PV758410 | N/A | N/A |
| Hainan | Site 4 | 2/1 | 16_Saccharum officinarum Leaf | (2) | CSFF 28102 | T22_B11_N/A_N/A | N/A | TB genotype 23 | *Ca. hongkongensis* | PV756845 | PV758411 | N/A | N/A |
| Hainan | Site 4 | 2/1 | 16_Saccharum officinarum Leaf | (2) | CSFF 28103 | T22_B11_N/A_N/A | N/A | TB genotype 23 | *Ca. hongkongensis* | PV756846 | PV758412 | N/A | N/A |
| Hainan | Site 4 | 2/1 | 16_Saccharum officinarum Leaf | (3) | CSFF 28104 | T22_B11_N/A_N/A | N/A | TB genotype 23 | *Ca. hongkongensis* | PV756847 | PV758413 | N/A | N/A |
| Hainan | Site 4 | 2/1 | 16_Saccharum officinarum Leaf | (3) | CSFF 28105 | T22_B11_N/A_N/A | N/A | TB genotype 23 | *Ca. hongkongensis* | PV756848 | PV758414 | N/A | N/A |
| Hainan | Site 4 | 2/1 | 16_Saccharum officinarum Leaf | (4) | CSFF 28106 | T16_B34_H23_C23 | TBHC genotype 9 | TB genotype 9 | *Ca. pseudoreteaudii* | PV756849 | PV758415 | PV757522 | PV757664 |
| Hainan | Site 4 | 2/1 | 16_Saccharum officinarum Leaf | (4) | CSFF 28107 | T16_B34_N/A_N/A | N/A | TB genotype 9 | *Ca. pseudoreteaudii* | PV756850 | PV758416 | N/A | N/A |
| Hainan | Site 4 | 2/1 | 16_Saccharum officinarum Leaf | (5) | CSFF 28108 | T22_B11_N/A_N/A | N/A | TB genotype 23 | *Ca. hongkongensis* | PV756851 | PV758417 | N/A | N/A |
| Hainan | Site 4 | 2/1 | 16_Saccharum officinarum Leaf | (5) | CSFF 28109 | T22_B11_N/A_N/A | N/A | TB genotype 23 | *Ca. hongkongensis* | PV756852 | PV758418 | N/A | N/A |
| Hainan | Site 4 | 2/1 | 17_Rosa chinensis Leaf | (1) | CSFF 28074 | T22_B11_N/A_N/A | N/A | TB genotype 23 | *Ca. hongkongensis* | PV756817 | PV758383 | N/A | N/A |
| Hainan | Site 4 | 2/1 | 17_Rosa chinensis Leaf | (1) | CSFF 28075 | T22_B11_N/A_N/A | N/A | TB genotype 23 | *Ca. hongkongensis* | PV756818 | PV758384 | N/A | N/A |
| Hainan | Site 4 | 2/1 | 17_Rosa chinensis Leaf | (2) | CSFF 28076 | T21_B15_H17_C11 | TBHC genotype 25 | TB genotype 22 | *Ca. hongkongensis* | PV756819 | PV758385 | PV757520 | PV757662 |
| Hainan | Site 4 | 2/1 | 17_Rosa chinensis Leaf | (2) | CSFF 28077 | T21_B15_H17_C11 | TBHC genotype 25 | TB genotype 22 | *Ca. hongkongensis* | PV756820 | PV758386 | PV757521 | PV757663 |
| Hainan | Site 4 | 2/1 | 17_Rosa chinensis Leaf | (4) | CSFF 28078 | T22_B11_N/A_N/A | N/A | TB genotype 23 | *Ca. hongkongensis* | PV756821 | PV758387 | N/A | N/A |
| Hainan | Site 4 | 2/1 | 17_Rosa chinensis Leaf | (4) | CSFF 28079 | T22_B11_N/A_N/A | N/A | TB genotype 23 | *Ca. hongkongensis* | PV756822 | PV758388 | N/A | N/A |
| Hainan | Site 4 | 2/1 | 18_Acacia mangium Leaf | (1) | CSFF 28110 | T22_B11_N/A_N/A | N/A | TB genotype 23 | *Ca. hongkongensis* | PV756853 | PV758419 | N/A | N/A |
| Hainan | Site 4 | 2/1 | 18_Acacia mangium Leaf | (1) | CSFF 28111 | T22_B11_N/A_N/A | N/A | TB genotype 23 | *Ca. hongkongensis* | PV756854 | PV758420 | N/A | N/A |
| Hainan | Site 4 | 2/1 | 18_Acacia mangium Leaf | (2) | CSFF 28112 | T22_B11_N/A_N/A | N/A | TB genotype 23 | *Ca. hongkongensis* | PV756855 | PV758421 | N/A | N/A |
| Hainan | Site 4 | 2/1 | 18_Acacia mangium Leaf | (2) | CSFF 28113 | T22_B11_N/A_N/A | N/A | TB genotype 23 | *Ca. hongkongensis* | PV756856 | PV758422 | N/A | N/A |
| Hainan | Site 4 | 2/1 | 18_Acacia mangium Leaf | (3) | CSFF 28114 | T22_B11_N/A_N/A | N/A | TB genotype 23 | *Ca. hongkongensis* | PV756857 | PV758423 | N/A | N/A |
| Hainan | Site 4 | 2/1 | 18_Acacia mangium Leaf | (3) | CSFF 28115 | T22_B11_N/A_N/A | N/A | TB genotype 23 | *Ca. hongkongensis* | PV756858 | PV758424 | N/A | N/A |
| Hainan | Site 4 | 2/1 | 18_Acacia mangium Leaf | (4) | CSFF 28116 | T22_B11_N/A_N/A | N/A | TB genotype 23 | *Ca. hongkongensis* | PV756859 | PV758425 | N/A | N/A |
| Hainan | Site 4 | 2/1 | 18_Acacia mangium Leaf | (4) | CSFF 28117 | T22_B11_N/A_N/A | N/A | TB genotype 23 | *Ca. hongkongensis* | PV756860 | PV758426 | N/A | N/A |
| Hainan | Site 4 | 2/1 | 18_Acacia mangium Leaf | (5) | CSFF 28118 | T22_B11_N/A_N/A | N/A | TB genotype 23 | *Ca. hongkongensis* | PV756861 | PV758427 | N/A | N/A |
| Hainan | Site 4 | 2/1 | 18_Acacia mangium Leaf | (5) | CSFF 28119 | T22_B11_N/A_N/A | N/A | TB genotype 23 | *Ca. hongkongensis* | PV756862 | PV758428 | N/A | N/A |
| Hainan | Site 4 | 2/1 | 19_Dianthus caryophyllus Leaf | (1) | CSFF 28066 | T21_B13_N/A_N/A | N/A | TB genotype 20 | *Ca. hongkongensis* | PV756809 | PV758375 | N/A | N/A |
| Hainan | Site 4 | 2/1 | 19_Dianthus caryophyllus Leaf | (1) | CSFF 28067 | T21_B13_N/A_N/A | N/A | TB genotype 20 | *Ca. hongkongensis* | PV756810 | PV758376 | N/A | N/A |
| Hainan | Site 4 | 2/1 | 19_Dianthus caryophyllus Leaf | (3) | CSFF 28068 | T22_B11_H17_C11 | TBHC genotype 26 | TB genotype 23 | *Ca. hongkongensis* | PV756811 | PV758377 | PV757518 | PV757660 |
| Hainan | Site 4 | 2/1 | 19_Dianthus caryophyllus Leaf | (3) | CSFF 28069 | T22_B11_N/A_N/A | N/A | TB genotype 23 | *Ca. hongkongensis* | PV756812 | PV758378 | N/A | N/A |
| Hainan | Site 4 | 2/1 | 19_Dianthus caryophyllus Leaf | (4) | CSFF 28070 | T16_B34_H23_C23 | TBHC genotype 9 | TB genotype 9 | *Ca. pseudoreteaudii* | PV756813 | PV758379 | PV757519 | PV757661 |
| Hainan | Site 4 | 2/1 | 19_Dianthus caryophyllus Leaf | (4) | CSFF 28071 | T16_B34_N/A_N/A | N/A | TB genotype 9 | *Ca. pseudoreteaudii* | PV756814 | PV758380 | N/A | N/A |
| Hainan | Site 4 | 2/1 | 19_Dianthus caryophyllus Leaf | (5) | CSFF 28072 | T22_B11_N/A_N/A | N/A | TB genotype 23 | *Ca. hongkongensis* | PV756815 | PV758381 | N/A | N/A |
| Hainan | Site 4 | 2/1 | 19_Dianthus caryophyllus Leaf | (5) | CSFF 28073 | T22_B11_N/A_N/A | N/A | TB genotype 23 | *Ca. hongkongensis* | PV756816 | PV758382 | N/A | N/A |
| Hainan | Site 4 | 2/1 | 20_Cinnamomum camphora Leaf | (1) | CSFF 28080 | T21_B13_N/A_N/A | N/A | TB genotype 20 | *Ca. hongkongensis* | PV756823 | PV758389 | N/A | N/A |
| Hainan | Site 4 | 2/1 | 20_Cinnamomum camphora Leaf | (1) | CSFF 28081 | T21_B13_N/A_N/A | N/A | TB genotype 20 | *Ca. hongkongensis* | PV756824 | PV758390 | N/A | N/A |
| Hainan | Site 4 | 2/1 | 20_Cinnamomum camphora Leaf | (2) | CSFF 28082 | T22_B11_N/A_N/A | N/A | TB genotype 23 | *Ca. hongkongensis* | PV756825 | PV758391 | N/A | N/A |
| Hainan | Site 4 | 2/1 | 20_Cinnamomum camphora Leaf | (2) | CSFF 28083 | T22_B11_N/A_N/A | N/A | TB genotype 23 | *Ca. hongkongensis* | PV756826 | PV758392 | N/A | N/A |
| Hainan | Site 4 | 2/1 | 20_Cinnamomum camphora Leaf | (3) | CSFF 28084 | T22_B11_N/A_N/A | N/A | TB genotype 23 | *Ca. hongkongensis* | PV756827 | PV758393 | N/A | N/A |
| Hainan | Site 4 | 2/1 | 20_Cinnamomum camphora Leaf | (3) | CSFF 28085 | T22_B11_N/A_N/A | N/A | TB genotype 23 | *Ca. hongkongensis* | PV756828 | PV758394 | N/A | N/A |
| Hainan | Site 4 | 2/1 | 20_Cinnamomum camphora Leaf | (4) | CSFF 28086 | T21_B13_N/A_N/A | N/A | TB genotype 20 | *Ca. hongkongensis* | PV756829 | PV758395 | N/A | N/A |
| Hainan | Site 4 | 2/1 | 20_Cinnamomum camphora Leaf | (4) | CSFF 28087 | T21_B13_N/A_N/A | N/A | TB genotype 20 | *Ca. hongkongensis* | PV756830 | PV758396 | N/A | N/A |
| Hainan | Site 4 | 2/1 | 20_Cinnamomum camphora Leaf | (5) | CSFF 28088 | T21_B13_N/A_N/A | N/A | TB genotype 20 | *Ca. hongkongensis* | PV756831 | PV758397 | N/A | N/A |
| Hainan | Site 4 | 2/1 | 20_Cinnamomum camphora Leaf | (5) | CSFF 28089 | T21_B13_N/A_N/A | N/A | TB genotype 20 | *Ca. hongkongensis* | PV756832 | PV758398 | N/A | N/A |
| Hainan | Site 4 | 2/1 | 21_Washingtonia filifera Leaf | (1) | CSFF 28090 | T22_B11_N/A_N/A | N/A | TB genotype 23 | *Ca. hongkongensis* | PV756833 | PV758399 | N/A | N/A |
| Hainan | Site 4 | 2/1 | 21_Washingtonia filifera Leaf | (1) | CSFF 28091 | T22_B11_N/A_N/A | N/A | TB genotype 23 | *Ca. hongkongensis* | PV756834 | PV758400 | N/A | N/A |
| Hainan | Site 4 | 2/1 | 21_Washingtonia filifera Leaf | (2) | CSFF 28092 | T22_B11_N/A_N/A | N/A | TB genotype 23 | *Ca. hongkongensis* | PV756835 | PV758401 | N/A | N/A |
| Hainan | Site 4 | 2/1 | 21_Washingtonia filifera Leaf | (2) | CSFF 28093 | T22_B11_N/A_N/A | N/A | TB genotype 23 | *Ca. hongkongensis* | PV756836 | PV758402 | N/A | N/A |
| Hainan | Site 4 | 2/1 | 21_Washingtonia filifera Leaf | (3) | CSFF 28094 | T21_B12_N/A_N/A | N/A | TB genotype 19 | *Ca. hongkongensis* | PV756837 | PV758403 | N/A | N/A |
| Hainan | Site 4 | 2/1 | 21_Washingtonia filifera Leaf | (3) | CSFF 28095 | T21_B12_N/A_N/A | N/A | TB genotype 19 | *Ca. hongkongensis* | PV756838 | PV758404 | N/A | N/A |
| Hainan | Site 4 | 2/1 | 21_Washingtonia filifera Leaf | (4) | CSFF 28096 | T22_B11_N/A_N/A | N/A | TB genotype 23 | *Ca. hongkongensis* | PV756839 | PV758405 | N/A | N/A |
| Hainan | Site 4 | 2/1 | 21_Washingtonia filifera Leaf | (4) | CSFF 28097 | T22_B11_N/A_N/A | N/A | TB genotype 23 | *Ca. hongkongensis* | PV756840 | PV758406 | N/A | N/A |
| Hainan | Site 4 | 2/1 | 21_Washingtonia filifera Leaf | (5) | CSFF 28098 | T22_B11_N/A_N/A | N/A | TB genotype 23 | *Ca. hongkongensis* | PV756841 | PV758407 | N/A | N/A |
| Hainan | Site 4 | 2/1 | 21_Washingtonia filifera Leaf | (5) | CSFF 28099 | T22_B11_N/A_N/A | N/A | TB genotype 23 | *Ca. hongkongensis* | PV756842 | PV758408 | N/A | N/A |
| Hainan | Site 4 | 2/1 | 22_Ficus benjamina Leaf | (1) | CSFF 28124 | T21_B12_N/A_N/A | N/A | TB genotype 19 | *Ca. hongkongensis* | PV756867 | PV758433 | N/A | N/A |
| Hainan | Site 4 | 2/1 | 22_Ficus benjamina Leaf | (1) | CSFF 28125 | T22_B11_N/A_N/A | N/A | TB genotype 23 | *Ca. hongkongensis* | PV756868 | PV758434 | N/A | N/A |
| Hainan | Site 4 | 2/1 | 22_Ficus benjamina Leaf | (2) | CSFF 28126 | T22_B11_N/A_N/A | N/A | TB genotype 23 | *Ca. hongkongensis* | PV756869 | PV758435 | N/A | N/A |
| Hainan | Site 4 | 2/1 | 22_Ficus benjamina Leaf | (2) | CSFF 28127 | T22_B11_N/A_N/A | N/A | TB genotype 23 | *Ca. hongkongensis* | PV756870 | PV758436 | N/A | N/A |
| Hainan | Site 4 | 2/1 | 22_Ficus benjamina Leaf | (3) | CSFF 28128 | T21_B15_H17_C11 | TBHC genotype 25 | TB genotype 22 | *Ca. hongkongensis* | PV756871 | PV758437 | PV757523 | PV757665 |
| Hainan | Site 4 | 2/1 | 22_Ficus benjamina Leaf | (3) | CSFF 28129 | T22_B11_N/A_N/A | N/A | TB genotype 23 | *Ca. hongkongensis* | PV756872 | PV758438 | N/A | N/A |
| Hainan | Site 4 | 2/1 | 22_Ficus benjamina Leaf | (4) | CSFF 28130 | T21_B13_N/A_N/A | N/A | TB genotype 20 | *Ca. hongkongensis* | PV756873 | PV758439 | N/A | N/A |
| Hainan | Site 4 | 2/1 | 22_Ficus benjamina Leaf | (4) | CSFF 28131 | T21_B13_N/A_N/A | N/A | TB genotype 20 | *Ca. hongkongensis* | PV756874 | PV758440 | N/A | N/A |
| Hainan | Site 4 | 2/1 | 22_Ficus benjamina Leaf | (5) | CSFF 28132 | T22_B11_N/A_N/A | N/A | TB genotype 23 | *Ca. hongkongensis* | PV756875 | PV758441 | N/A | N/A |
| Hainan | Site 4 | 2/1 | 22_Ficus benjamina Leaf | (5) | CSFF 28133 | T22_B11_N/A_N/A | N/A | TB genotype 23 | *Ca. hongkongensis* | PV756876 | PV758442 | N/A | N/A |
| Hainan | Site 4 | 2/1 | 23_Rhododendron pulchrum Leaf | (1) | CSFF 28142 | T22_B11_N/A_N/A | N/A | TB genotype 23 | *Ca. hongkongensis* | PV756885 | PV758451 | N/A | N/A |
| Hainan | Site 4 | 2/1 | 23_Rhododendron pulchrum Leaf | (1) | CSFF 28143 | T22_B11_N/A_N/A | N/A | TB genotype 23 | *Ca. hongkongensis* | PV756886 | PV758452 | N/A | N/A |
| Hainan | Site 4 | 2/1 | 23_Rhododendron pulchrum Leaf | (2) | CSFF 28144 | T21_B13_H17_C11 | TBHC genotype 23 | TB genotype 20 | *Ca. hongkongensis* | PV756887 | PV758453 | PV757524 | PV757666 |
| Hainan | Site 4 | 2/1 | 23_Rhododendron pulchrum Leaf | (2) | CSFF 28145 | T21_B13_N/A_N/A | N/A | TB genotype 20 | *Ca. hongkongensis* | PV756888 | PV758454 | N/A | N/A |
| Hainan | Site 4 | 2/1 | 23_Rhododendron pulchrum Leaf | (3) | CSFF 28146 | T21_B13_N/A_N/A | N/A | TB genotype 20 | *Ca. hongkongensis* | PV756889 | PV758455 | N/A | N/A |
| Hainan | Site 4 | 2/1 | 23_Rhododendron pulchrum Leaf | (3) | CSFF 28147 | T21_B13_N/A_N/A | N/A | TB genotype 20 | *Ca. hongkongensis* | PV756890 | PV758456 | N/A | N/A |
| Hainan | Site 4 | 2/1 | 23_Rhododendron pulchrum Leaf | (4) | CSFF 28148 | T22_B11_N/A_N/A | N/A | TB genotype 23 | *Ca. hongkongensis* | PV756891 | PV758457 | N/A | N/A |
| Hainan | Site 4 | 2/1 | 23_Rhododendron pulchrum Leaf | (4) | CSFF 28149 | T22_B11_N/A_N/A | N/A | TB genotype 23 | *Ca. hongkongensis* | PV756892 | PV758458 | N/A | N/A |
| Hainan | Site 4 | 2/1 | 23_Rhododendron pulchrum Leaf | (5) | CSFF 28150 | T22_B11_N/A_N/A | N/A | TB genotype 23 | *Ca. hongkongensis* | PV756893 | PV758459 | N/A | N/A |
| Hainan | Site 4 | 2/1 | 23_Rhododendron pulchrum Leaf | (5) | CSFF 28151 | T21_B12_H17_C12 | TBHC genotype 22 | TB genotype 19 | *Ca. hongkongensis* | PV756894 | PV758460 | PV757525 | PV757667 |
| Hainan | Site 4 | 2/1 | 26_Medicago sativa Seed | (1) | CSFF 28170 | T22_B11_H17_C11 | TBHC genotype 26 | TB genotype 23 | *Ca. hongkongensis* | PV756913 | PV758479 | PV757530 | PV757672 |
| Hainan | Site 4 | 2/1 | 26_Medicago sativa Seed | (1) | CSFF 28171 | T22_B11_N/A_N/A | N/A | TB genotype 23 | *Ca. hongkongensis* | PV756914 | PV758480 | N/A | N/A |
| Hainan | Site 4 | 2/1 | 26_Medicago sativa Seed | (2) | CSFF 28172 | T21_B13_N/A_N/A | N/A | TB genotype 20 | *Ca. hongkongensis* | PV756915 | PV758481 | N/A | N/A |
| Hainan | Site 4 | 2/1 | 26_Medicago sativa Seed | (2) | CSFF 28173 | T22_B11_N/A_N/A | N/A | TB genotype 23 | *Ca. hongkongensis* | PV756916 | PV758482 | N/A | N/A |
| Hainan | Site 4 | 2/1 | 26_Medicago sativa Seed | (3) | CSFF 28174 | T22_B11_N/A_N/A | N/A | TB genotype 23 | *Ca. hongkongensis* | PV756917 | PV758483 | N/A | N/A |
| Hainan | Site 4 | 2/1 | 26_Medicago sativa Seed | (3) | CSFF 28175 | T22_B11_N/A_N/A | N/A | TB genotype 23 | *Ca. hongkongensis* | PV756918 | PV758484 | N/A | N/A |
| Hainan | Site 4 | 2/1 | 26_Medicago sativa Seed | (4) | CSFF 28176 | T22_B11_N/A_N/A | N/A | TB genotype 23 | *Ca. hongkongensis* | PV756919 | PV758485 | N/A | N/A |
| Hainan | Site 4 | 2/1 | 26_Medicago sativa Seed | (4) | CSFF 28177 | T22_B11_N/A_N/A | N/A | TB genotype 23 | *Ca. hongkongensis* | PV756920 | PV758486 | N/A | N/A |
| Hainan | Site 4 | 2/1 | 26_Medicago sativa Seed | (5) | CSFF 28178 | T22_B11_N/A_N/A | N/A | TB genotype 23 | *Ca. hongkongensis* | PV756921 | PV758487 | N/A | N/A |
| Hainan | Site 4 | 2/1 | 26_Medicago sativa Seed | (5) | CSFF 28179 | T22_B11_N/A_N/A | N/A | TB genotype 23 | *Ca. hongkongensis* | PV756922 | PV758488 | N/A | N/A |
| Hainan | Site 5 | 2/2 | 1_Brassica rapa subsp. pekinensis Seed | (3) | CSFF 28214 | T20_B30_H21_C17 | TBHC genotype 19 | TB genotype 17 | *Ca. chinensis* | PV756957 | PV758523 | PV757534 | PV757676 |
| Hainan | Site 5 | 2/2 | 1_Brassica rapa subsp. pekinensis Seed | (3) | CSFF 28215 | T20_B30_N/A_N/A | N/A | TB genotype 17 | *Ca. chinensis* | PV756958 | PV758524 | N/A | N/A |
| Hainan | Site 5 | 2/2 | 3_Lolium perenne Seed | (5) | CSFF 28220 | T21_B11_N/A_N/A | N/A | TB genotype 18 | *Ca. hongkongensis* | PV756963 | PV758529 | N/A | N/A |
| Hainan | Site 5 | 2/2 | 3_Lolium perenne Seed | (5) | CSFF 28221 | T21_B11_N/A_N/A | N/A | TB genotype 18 | *Ca. hongkongensis* | PV756964 | PV758530 | N/A | N/A |
| Hainan | Site 5 | 2/2 | 5_Impatiens balsamina Seed | (1) | CSFF 28216 | T21_B11_N/A_N/A | N/A | TB genotype 18 | *Ca. hongkongensis* | PV756959 | PV758525 | N/A | N/A |
| Hainan | Site 5 | 2/2 | 5_Impatiens balsamina Seed | (1) | CSFF 28217 | T21_B11_N/A_N/A | N/A | TB genotype 18 | *Ca. hongkongensis* | PV756960 | PV758526 | N/A | N/A |
| Hainan | Site 5 | 2/2 | 5_Impatiens balsamina Seed | (2) | CSFF 28218 | T21_B13_N/A_N/A | N/A | TB genotype 20 | *Ca. hongkongensis* | PV756961 | PV758527 | N/A | N/A |
| Hainan | Site 5 | 2/2 | 5_Impatiens balsamina Seed | (2) | CSFF 28219 | T21_B13_N/A_N/A | N/A | TB genotype 20 | *Ca. hongkongensis* | PV756962 | PV758528 | N/A | N/A |
| Hainan | Site 5 | 2/2 | 6_Sesamum indicum Seed | (1) | CSFF 28200 | T21_B11_N/A_N/A | N/A | TB genotype 18 | *Ca. hongkongensis* | PV756943 | PV758509 | N/A | N/A |
| Hainan | Site 5 | 2/2 | 6_Sesamum indicum Seed | (1) | CSFF 28201 | T21_B11_N/A_N/A | N/A | TB genotype 18 | *Ca. hongkongensis* | PV756944 | PV758510 | N/A | N/A |
| Hainan | Site 5 | 2/2 | 6_Sesamum indicum Seed | (3) | CSFF 28202 | T21_B11_N/A_N/A | N/A | TB genotype 18 | *Ca. hongkongensis* | PV756945 | PV758511 | N/A | N/A |
| Hainan | Site 5 | 2/2 | 6_Sesamum indicum Seed | (3) | CSFF 28203 | T21_B11_N/A_N/A | N/A | TB genotype 18 | *Ca. hongkongensis* | PV756946 | PV758512 | N/A | N/A |
| Hainan | Site 5 | 2/2 | 6_Sesamum indicum Seed | (4) | CSFF 28204 | T21_B11_N/A_N/A | N/A | TB genotype 18 | *Ca. hongkongensis* | PV756947 | PV758513 | N/A | N/A |
| Hainan | Site 5 | 2/2 | 6_Sesamum indicum Seed | (4) | CSFF 28205 | T21_B11_N/A_N/A | N/A | TB genotype 18 | *Ca. hongkongensis* | PV756948 | PV758514 | N/A | N/A |
| Hainan | Site 5 | 2/2 | 6_Sesamum indicum Seed | (5) | CSFF 28206 | T21_B11_N/A_N/A | N/A | TB genotype 18 | *Ca. hongkongensis* | PV756949 | PV758515 | N/A | N/A |
| Hainan | Site 5 | 2/2 | 6_Sesamum indicum Seed | (5) | CSFF 28207 | T21_B11_N/A_N/A | N/A | TB genotype 18 | *Ca. hongkongensis* | PV756950 | PV758516 | N/A | N/A |
| Hainan | Site 5 | 2/2 | 7_Dianthus caryophyllus Seed | (2) | CSFF 28208 | T21_B11_N/A_N/A | N/A | TB genotype 18 | *Ca. hongkongensis* | PV756951 | PV758517 | N/A | N/A |
| Hainan | Site 5 | 2/2 | 7_Dianthus caryophyllus Seed | (2) | CSFF 28209 | T21_B11_N/A_N/A | N/A | TB genotype 18 | *Ca. hongkongensis* | PV756952 | PV758518 | N/A | N/A |
| Hainan | Site 5 | 2/2 | 7_Dianthus caryophyllus Seed | (3) | CSFF 28210 | T21_B11_N/A_N/A | N/A | TB genotype 18 | *Ca. hongkongensis* | PV756953 | PV758519 | N/A | N/A |
| Hainan | Site 5 | 2/2 | 7_Dianthus caryophyllus Seed | (3) | CSFF 28211 | T21_B11_N/A_N/A | N/A | TB genotype 18 | *Ca. hongkongensis* | PV756954 | PV758520 | N/A | N/A |
| Hainan | Site 5 | 2/2 | 7_Dianthus caryophyllus Seed | (4) | CSFF 28212 | T21_B11_N/A_N/A | N/A | TB genotype 18 | *Ca. hongkongensis* | PV756955 | PV758521 | N/A | N/A |
| Hainan | Site 5 | 2/2 | 7_Dianthus caryophyllus Seed | (4) | CSFF 28213 | T21_B11_N/A_N/A | N/A | TB genotype 18 | *Ca. hongkongensis* | PV756956 | PV758522 | N/A | N/A |
| Hainan | Site 5 | 2/2 | 8_Trifolium pratense Seed | (1) | CSFF 28190 | T21_B11_N/A_N/A | N/A | TB genotype 18 | *Ca. hongkongensis* | PV756933 | PV758499 | N/A | N/A |
| Hainan | Site 5 | 2/2 | 8_Trifolium pratense Seed | (1) | CSFF 28191 | T21_B11_N/A_N/A | N/A | TB genotype 18 | *Ca. hongkongensis* | PV756934 | PV758500 | N/A | N/A |
| Hainan | Site 5 | 2/2 | 8_Trifolium pratense Seed | (2) | CSFF 28192 | T21_B11_N/A_N/A | N/A | TB genotype 18 | *Ca. hongkongensis* | PV756935 | PV758501 | N/A | N/A |
| Hainan | Site 5 | 2/2 | 8_Trifolium pratense Seed | (2) | CSFF 28193 | T21_B11_N/A_N/A | N/A | TB genotype 18 | *Ca. hongkongensis* | PV756936 | PV758502 | N/A | N/A |
| Hainan | Site 5 | 2/2 | 8_Trifolium pratense Seed | (3) | CSFF 28194 | T21_B11_N/A_N/A | N/A | TB genotype 18 | *Ca. hongkongensis* | PV756937 | PV758503 | N/A | N/A |
| Hainan | Site 5 | 2/2 | 8_Trifolium pratense Seed | (3) | CSFF 28195 | T21_B13_N/A_N/A | N/A | TB genotype 20 | *Ca. hongkongensis* | PV756938 | PV758504 | N/A | N/A |
| Hainan | Site 5 | 2/2 | 8_Trifolium pratense Seed | (4) | CSFF 28196 | T21_B11_N/A_N/A | N/A | TB genotype 18 | *Ca. hongkongensis* | PV756939 | PV758505 | N/A | N/A |
| Hainan | Site 5 | 2/2 | 8_Trifolium pratense Seed | (4) | CSFF 28197 | T21_B11_N/A_N/A | N/A | TB genotype 18 | *Ca. hongkongensis* | PV756940 | PV758506 | N/A | N/A |
| Hainan | Site 5 | 2/2 | 8_Trifolium pratense Seed | (5) | CSFF 28198 | T21_B11_N/A_N/A | N/A | TB genotype 18 | *Ca. hongkongensis* | PV756941 | PV758507 | N/A | N/A |
| Hainan | Site 5 | 2/2 | 8_Trifolium pratense Seed | (5) | CSFF 28199 | T21_B11_N/A_N/A | N/A | TB genotype 18 | *Ca. hongkongensis* | PV756942 | PV758508 | N/A | N/A |
| Hainan | Site 5 | 2/2 | 9_Lythrum salicaria Seed | (1) | CSFF 28180 | T21_B11_H17_C11 | TBHC genotype 20 | TB genotype 18 | *Ca. hongkongensis* | PV756923 | PV758489 | PV757531 | PV757673 |
| Hainan | Site 5 | 2/2 | 9_Lythrum salicaria Seed | (1) | CSFF 28181 | T21_B11_N/A_N/A | N/A | TB genotype 18 | *Ca. hongkongensis* | PV756924 | PV758490 | N/A | N/A |
| Hainan | Site 5 | 2/2 | 9_Lythrum salicaria Seed | (2) | CSFF 28182 | T21_B11_N/A_N/A | N/A | TB genotype 18 | *Ca. hongkongensis* | PV756925 | PV758491 | N/A | N/A |
| Hainan | Site 5 | 2/2 | 9_Lythrum salicaria Seed | (2) | CSFF 28183 | T21_B13_H17_C11 | TBHC genotype 23 | TB genotype 20 | *Ca. hongkongensis* | PV756926 | PV758492 | PV757532 | PV757674 |
| Hainan | Site 5 | 2/2 | 9_Lythrum salicaria Seed | (3) | CSFF 28184 | T21_B11_N/A_N/A | N/A | TB genotype 18 | *Ca. hongkongensis* | PV756927 | PV758493 | N/A | N/A |
| Hainan | Site 5 | 2/2 | 9_Lythrum salicaria Seed | (3) | CSFF 28185 | T21_B13_N/A_N/A | N/A | TB genotype 20 | *Ca. hongkongensis* | PV756928 | PV758494 | N/A | N/A |
| Hainan | Site 5 | 2/2 | 9_Lythrum salicaria Seed | (4) | CSFF 28186 | T21_B13_N/A_N/A | N/A | TB genotype 20 | *Ca. hongkongensis* | PV756929 | PV758495 | N/A | N/A |
| Hainan | Site 5 | 2/2 | 9_Lythrum salicaria Seed | (4) | CSFF 28187 | T19_B30_H22_C16 | TBHC genotype 17 | TB genotype 15 | *Ca. chinensis* | PV756930 | PV758496 | PV757533 | PV757675 |
| Hainan | Site 5 | 2/2 | 9_Lythrum salicaria Seed | (5) | CSFF 28188 | T21_B11_N/A_N/A | N/A | TB genotype 18 | *Ca. hongkongensis* | PV756931 | PV758497 | N/A | N/A |
| Hainan | Site 5 | 2/2 | 9_Lythrum salicaria Seed | (5) | CSFF 28189 | T21_B11_N/A_N/A | N/A | TB genotype 18 | *Ca. hongkongensis* | PV756932 | PV758498 | N/A | N/A |
| Hainan | Site 5 | 2/2 | 10_Buxus sinica Leaf | (1) | CSFF 28324 | T21_B11_N/A_N/A | N/A | TB genotype 18 | *Ca. hongkongensis* | PV757067 | PV758633 | N/A | N/A |
| Hainan | Site 5 | 2/2 | 10_Buxus sinica Leaf | (1) | CSFF 28325 | T21_B11_N/A_N/A | N/A | TB genotype 18 | *Ca. hongkongensis* | PV757068 | PV758634 | N/A | N/A |
| Hainan | Site 5 | 2/2 | 10_Buxus sinica Leaf | (2) | CSFF 28326 | T21_B12_H17_C11 | TBHC genotype 21 | TB genotype 19 | *Ca. hongkongensis* | PV757069 | PV758635 | PV757542 | PV757684 |
| Hainan | Site 5 | 2/2 | 10_Buxus sinica Leaf | (2) | CSFF 28327 | T21_B12_N/A_N/A | N/A | TB genotype 19 | *Ca. hongkongensis* | PV757070 | PV758636 | N/A | N/A |
| Hainan | Site 5 | 2/2 | 10_Buxus sinica Leaf | (4) | CSFF 28328 | T21_B11_N/A_N/A | N/A | TB genotype 18 | *Ca. hongkongensis* | PV757071 | PV758637 | N/A | N/A |
| Hainan | Site 5 | 2/2 | 10_Buxus sinica Leaf | (4) | CSFF 28329 | T21_B11_N/A_N/A | N/A | TB genotype 18 | *Ca. hongkongensis* | PV757072 | PV758638 | N/A | N/A |
| Hainan | Site 5 | 2/2 | 11_Imperata cylindrica Leaf | (3) | CSFF 28300 | T21_B11_N/A_N/A | N/A | TB genotype 18 | *Ca. hongkongensis* | PV757043 | PV758609 | N/A | N/A |
| Hainan | Site 5 | 2/2 | 11_Imperata cylindrica Leaf | (3) | CSFF 28301 | T21_B11_N/A_N/A | N/A | TB genotype 18 | *Ca. hongkongensis* | PV757044 | PV758610 | N/A | N/A |
| Hainan | Site 5 | 2/2 | 11_Imperata cylindrica Leaf | (4) | CSFF 28302 | T21_B13_N/A_N/A | N/A | TB genotype 20 | *Ca. hongkongensis* | PV757045 | PV758611 | N/A | N/A |
| Hainan | Site 5 | 2/2 | 11_Imperata cylindrica Leaf | (4) | CSFF 28303 | T21_B13_N/A_N/A | N/A | TB genotype 20 | *Ca. hongkongensis* | PV757046 | PV758612 | N/A | N/A |
| Hainan | Site 5 | 2/2 | 12_Pinus massoniana Leaf | (1) | CSFF 28304 | T21_B11_N/A_N/A | N/A | TB genotype 18 | *Ca. hongkongensis* | PV757047 | PV758613 | N/A | N/A |
| Hainan | Site 5 | 2/2 | 12_Pinus massoniana Leaf | (1) | CSFF 28305 | T19_B30_H22_C16 | TBHC genotype 17 | TB genotype 15 | *Ca. chinensis* | PV757048 | PV758614 | PV757541 | PV757683 |
| Hainan | Site 5 | 2/2 | 12_Pinus massoniana Leaf | (2) | CSFF 28306 | T21_B11_N/A_N/A | N/A | TB genotype 18 | *Ca. hongkongensis* | PV757049 | PV758615 | N/A | N/A |
| Hainan | Site 5 | 2/2 | 12_Pinus massoniana Leaf | (2) | CSFF 28307 | T21_B11_N/A_N/A | N/A | TB genotype 18 | *Ca. hongkongensis* | PV757050 | PV758616 | N/A | N/A |
| Hainan | Site 5 | 2/2 | 12_Pinus massoniana Leaf | (3) | CSFF 28308 | T21_B13_N/A_N/A | N/A | TB genotype 20 | *Ca. hongkongensis* | PV757051 | PV758617 | N/A | N/A |
| Hainan | Site 5 | 2/2 | 12_Pinus massoniana Leaf | (3) | CSFF 28309 | T21_B13_N/A_N/A | N/A | TB genotype 20 | *Ca. hongkongensis* | PV757052 | PV758618 | N/A | N/A |
| Hainan | Site 5 | 2/2 | 12_Pinus massoniana Leaf | (4) | CSFF 28310 | T21_B11_N/A_N/A | N/A | TB genotype 18 | *Ca. hongkongensis* | PV757053 | PV758619 | N/A | N/A |
| Hainan | Site 5 | 2/2 | 12_Pinus massoniana Leaf | (4) | CSFF 28311 | T21_B11_N/A_N/A | N/A | TB genotype 18 | *Ca. hongkongensis* | PV757054 | PV758620 | N/A | N/A |
| Hainan | Site 5 | 2/2 | 12_Pinus massoniana Leaf | (5) | CSFF 28312 | T21_B11_N/A_N/A | N/A | TB genotype 18 | *Ca. hongkongensis* | PV757055 | PV758621 | N/A | N/A |
| Hainan | Site 5 | 2/2 | 12_Pinus massoniana Leaf | (5) | CSFF 28313 | T21_B11_N/A_N/A | N/A | TB genotype 18 | *Ca. hongkongensis* | PV757056 | PV758622 | N/A | N/A |
| Hainan | Site 5 | 2/2 | 13_Eucalyptus urophylla hybrid Leaf | (1) | CSFF 28330 | T21_B11_H17_C11 | TBHC genotype 20 | TB genotype 18 | *Ca. hongkongensis* | PV757073 | PV758639 | PV757543 | PV757685 |
| Hainan | Site 5 | 2/2 | 13_Eucalyptus urophylla hybrid Leaf | (1) | CSFF 28331 | T21_B11_N/A_N/A | N/A | TB genotype 18 | *Ca. hongkongensis* | PV757074 | PV758640 | N/A | N/A |
| Hainan | Site 5 | 2/2 | 13_Eucalyptus urophylla hybrid Leaf | (2) | CSFF 28332 | T21_B11_N/A_N/A | N/A | TB genotype 18 | *Ca. hongkongensis* | PV757075 | PV758641 | N/A | N/A |
| Hainan | Site 5 | 2/2 | 13_Eucalyptus urophylla hybrid Leaf | (2) | CSFF 28333 | T21_B11_N/A_N/A | N/A | TB genotype 18 | *Ca. hongkongensis* | PV757076 | PV758642 | N/A | N/A |
| Hainan | Site 5 | 2/2 | 13_Eucalyptus urophylla hybrid Leaf | (3) | CSFF 28334 | T19_B30_H22_C16 | TBHC genotype 17 | TB genotype 15 | *Ca. chinensis* | PV757077 | PV758643 | PV757544 | PV757686 |
| Hainan | Site 5 | 2/2 | 13_Eucalyptus urophylla hybrid Leaf | (3) | CSFF 28335 | T19_B30_N/A_N/A | N/A | TB genotype 15 | *Ca. chinensis* | PV757078 | PV758644 | N/A | N/A |
| Hainan | Site 5 | 2/2 | 13_Eucalyptus urophylla hybrid Leaf | (5) | CSFF 28336 | T21_B11_N/A_N/A | N/A | TB genotype 18 | *Ca. hongkongensis* | PV757079 | PV758645 | N/A | N/A |
| Hainan | Site 5 | 2/2 | 13_Eucalyptus urophylla hybrid Leaf | (5) | CSFF 28337 | T21_B11_N/A_N/A | N/A | TB genotype 18 | *Ca. hongkongensis* | PV757080 | PV758646 | N/A | N/A |
| Hainan | Site 5 | 2/2 | 14_Syzygium jambos Leaf | (2) | CSFF 28282 | T21_B11_N/A_N/A | N/A | TB genotype 18 | *Ca. hongkongensis* | PV757025 | PV758591 | N/A | N/A |
| Hainan | Site 5 | 2/2 | 14_Syzygium jambos Leaf | (2) | CSFF 28283 | T21_B11_N/A_N/A | N/A | TB genotype 18 | *Ca. hongkongensis* | PV757026 | PV758592 | N/A | N/A |
| Hainan | Site 5 | 2/2 | 14_Syzygium jambos Leaf | (3) | CSFF 28284 | T21_B11_N/A_N/A | N/A | TB genotype 18 | *Ca. hongkongensis* | PV757027 | PV758593 | N/A | N/A |
| Hainan | Site 5 | 2/2 | 14_Syzygium jambos Leaf | (3) | CSFF 28285 | T21_B11_N/A_N/A | N/A | TB genotype 18 | *Ca. hongkongensis* | PV757028 | PV758594 | N/A | N/A |
| Hainan | Site 5 | 2/2 | 14_Syzygium jambos Leaf | (4) | CSFF 28286 | T19_B30_N/A_N/A | N/A | TB genotype 15 | *Ca. chinensis* | PV757029 | PV758595 | N/A | N/A |
| Hainan | Site 5 | 2/2 | 14_Syzygium jambos Leaf | (4) | CSFF 28287 | T21_B11_N/A_N/A | N/A | TB genotype 18 | *Ca. hongkongensis* | PV757030 | PV758596 | N/A | N/A |
| Hainan | Site 5 | 2/2 | 14_Syzygium jambos Leaf | (5) | CSFF 28288 | T21_B11_N/A_N/A | N/A | TB genotype 18 | *Ca. hongkongensis* | PV757031 | PV758597 | N/A | N/A |
| Hainan | Site 5 | 2/2 | 14_Syzygium jambos Leaf | (5) | CSFF 28289 | T21_B11_N/A_N/A | N/A | TB genotype 18 | *Ca. hongkongensis* | PV757032 | PV758598 | N/A | N/A |
| Hainan | Site 5 | 2/2 | 15_Pennisetum purpureum Leaf | (1) | CSFF 28338 | T21_B11_N/A_N/A | N/A | TB genotype 18 | *Ca. hongkongensis* | PV757081 | PV758647 | N/A | N/A |
| Hainan | Site 5 | 2/2 | 15_Pennisetum purpureum Leaf | (1) | CSFF 28339 | T21_B11_N/A_N/A | N/A | TB genotype 18 | *Ca. hongkongensis* | PV757082 | PV758648 | N/A | N/A |
| Hainan | Site 5 | 2/2 | 15_Pennisetum purpureum Leaf | (2) | CSFF 28340 | T21_B11_N/A_N/A | N/A | TB genotype 18 | *Ca. hongkongensis* | PV757083 | PV758649 | N/A | N/A |
| Hainan | Site 5 | 2/2 | 15_Pennisetum purpureum Leaf | (2) | CSFF 28341 | T21_B11_N/A_N/A | N/A | TB genotype 18 | *Ca. hongkongensis* | PV757084 | PV758650 | N/A | N/A |
| Hainan | Site 5 | 2/2 | 15_Pennisetum purpureum Leaf | (3) | CSFF 28342 | T21_B11_N/A_N/A | N/A | TB genotype 18 | *Ca. hongkongensis* | PV757085 | PV758651 | N/A | N/A |
| Hainan | Site 5 | 2/2 | 15_Pennisetum purpureum Leaf | (3) | CSFF 28343 | T21_B11_N/A_N/A | N/A | TB genotype 18 | *Ca. hongkongensis* | PV757086 | PV758652 | N/A | N/A |
| Hainan | Site 5 | 2/2 | 15_Pennisetum purpureum Leaf | (4) | CSFF 28344 | T21_B11_N/A_N/A | N/A | TB genotype 18 | *Ca. hongkongensis* | PV757087 | PV758653 | N/A | N/A |
| Hainan | Site 5 | 2/2 | 15_Pennisetum purpureum Leaf | (4) | CSFF 28345 | T21_B11_N/A_N/A | N/A | TB genotype 18 | *Ca. hongkongensis* | PV757088 | PV758654 | N/A | N/A |
| Hainan | Site 5 | 2/2 | 15_Pennisetum purpureum Leaf | (5) | CSFF 28346 | T21_B13_N/A_N/A | N/A | TB genotype 20 | *Ca. hongkongensis* | PV757089 | PV758655 | N/A | N/A |
| Hainan | Site 5 | 2/2 | 15_Pennisetum purpureum Leaf | (5) | CSFF 28347 | T21_B11_N/A_N/A | N/A | TB genotype 18 | *Ca. hongkongensis* | PV757090 | PV758656 | N/A | N/A |
| Hainan | Site 5 | 2/2 | 16_Saccharum officinarum Leaf | (1) | CSFF 28262 | T21_B11_N/A_N/A | N/A | TB genotype 18 | *Ca. hongkongensis* | PV757005 | PV758571 | N/A | N/A |
| Hainan | Site 5 | 2/2 | 16_Saccharum officinarum Leaf | (1) | CSFF 28263 | T21_B11_N/A_N/A | N/A | TB genotype 18 | *Ca. hongkongensis* | PV757006 | PV758572 | N/A | N/A |
| Hainan | Site 5 | 2/2 | 16_Saccharum officinarum Leaf | (2) | CSFF 28264 | T21_B11_N/A_N/A | N/A | TB genotype 18 | *Ca. hongkongensis* | PV757007 | PV758573 | N/A | N/A |
| Hainan | Site 5 | 2/2 | 16_Saccharum officinarum Leaf | (2) | CSFF 28265 | T19_B31_H21_C16 | TBHC genotype 18 | TB genotype 16 | *Ca. chinensis* | PV757008 | PV758574 | PV757538 | PV757680 |
| Hainan | Site 5 | 2/2 | 16_Saccharum officinarum Leaf | (3) | CSFF 28266 | T20_B30_H21_C17 | TBHC genotype 19 | TB genotype 17 | *Ca. chinensis* | PV757009 | PV758575 | PV757539 | PV757681 |
| Hainan | Site 5 | 2/2 | 16_Saccharum officinarum Leaf | (3) | CSFF 28267 | T20_B30_N/A_N/A | N/A | TB genotype 17 | *Ca. chinensis* | PV757010 | PV758576 | N/A | N/A |
| Hainan | Site 5 | 2/2 | 16_Saccharum officinarum Leaf | (4) | CSFF 28268 | T21_B11_N/A_N/A | N/A | TB genotype 18 | *Ca. hongkongensis* | PV757011 | PV758577 | N/A | N/A |
| Hainan | Site 5 | 2/2 | 16_Saccharum officinarum Leaf | (4) | CSFF 28269 | T20_B30_N/A_N/A | N/A | TB genotype 17 | *Ca. chinensis* | PV757012 | PV758578 | N/A | N/A |
| Hainan | Site 5 | 2/2 | 16_Saccharum officinarum Leaf | (5) | CSFF 28270 | T21_B13_N/A_N/A | N/A | TB genotype 20 | *Ca. hongkongensis* | PV757013 | PV758579 | N/A | N/A |
| Hainan | Site 5 | 2/2 | 16_Saccharum officinarum Leaf | (5) | CSFF 28271 | T21_B11_N/A_N/A | N/A | TB genotype 18 | *Ca. hongkongensis* | PV757014 | PV758580 | N/A | N/A |
| Hainan | Site 5 | 2/2 | 17_Rosa chinensis Leaf | (1) | CSFF 28232 | T21_B11_N/A_N/A | N/A | TB genotype 18 | *Ca. hongkongensis* | PV756975 | PV758541 | N/A | N/A |
| Hainan | Site 5 | 2/2 | 17_Rosa chinensis Leaf | (1) | CSFF 28233 | T19_B30_H22_C16 | TBHC genotype 17 | TB genotype 15 | *Ca. chinensis* | PV756976 | PV758542 | PV757537 | PV757679 |
| Hainan | Site 5 | 2/2 | 17_Rosa chinensis Leaf | (2) | CSFF 28234 | T21_B13_N/A_N/A | N/A | TB genotype 20 | *Ca. hongkongensis* | PV756977 | PV758543 | N/A | N/A |
| Hainan | Site 5 | 2/2 | 17_Rosa chinensis Leaf | (2) | CSFF 28235 | T21_B11_N/A_N/A | N/A | TB genotype 18 | *Ca. hongkongensis* | PV756978 | PV758544 | N/A | N/A |
| Hainan | Site 5 | 2/2 | 17_Rosa chinensis Leaf | (3) | CSFF 28236 | T21_B12_N/A_N/A | N/A | TB genotype 19 | *Ca. hongkongensis* | PV756979 | PV758545 | N/A | N/A |
| Hainan | Site 5 | 2/2 | 17_Rosa chinensis Leaf | (3) | CSFF 28237 | T21_B12_N/A_N/A | N/A | TB genotype 19 | *Ca. hongkongensis* | PV756980 | PV758546 | N/A | N/A |
| Hainan | Site 5 | 2/2 | 17_Rosa chinensis Leaf | (4) | CSFF 28238 | T21_B13_N/A_N/A | N/A | TB genotype 20 | *Ca. hongkongensis* | PV756981 | PV758547 | N/A | N/A |
| Hainan | Site 5 | 2/2 | 17_Rosa chinensis Leaf | (4) | CSFF 28239 | T21_B11_N/A_N/A | N/A | TB genotype 18 | *Ca. hongkongensis* | PV756982 | PV758548 | N/A | N/A |
| Hainan | Site 5 | 2/2 | 17_Rosa chinensis Leaf | (5) | CSFF 28240 | T21_B11_N/A_N/A | N/A | TB genotype 18 | *Ca. hongkongensis* | PV756983 | PV758549 | N/A | N/A |
| Hainan | Site 5 | 2/2 | 17_Rosa chinensis Leaf | (5) | CSFF 28241 | T21_B11_N/A_N/A | N/A | TB genotype 18 | *Ca. hongkongensis* | PV756984 | PV758550 | N/A | N/A |
| Hainan | Site 5 | 2/2 | 18_Acacia mangium Leaf | (1) | CSFF 28272 | T21_B11_N/A_N/A | N/A | TB genotype 18 | *Ca. hongkongensis* | PV757015 | PV758581 | N/A | N/A |
| Hainan | Site 5 | 2/2 | 18_Acacia mangium Leaf | (1) | CSFF 28273 | T21_B11_N/A_N/A | N/A | TB genotype 18 | *Ca. hongkongensis* | PV757016 | PV758582 | N/A | N/A |
| Hainan | Site 5 | 2/2 | 18_Acacia mangium Leaf | (2) | CSFF 28274 | T21_B11_N/A_N/A | N/A | TB genotype 18 | *Ca. hongkongensis* | PV757017 | PV758583 | N/A | N/A |
| Hainan | Site 5 | 2/2 | 18_Acacia mangium Leaf | (2) | CSFF 28275 | T21_B11_N/A_N/A | N/A | TB genotype 18 | *Ca. hongkongensis* | PV757018 | PV758584 | N/A | N/A |
| Hainan | Site 5 | 2/2 | 18_Acacia mangium Leaf | (3) | CSFF 28276 | T21_B11_N/A_N/A | N/A | TB genotype 18 | *Ca. hongkongensis* | PV757019 | PV758585 | N/A | N/A |
| Hainan | Site 5 | 2/2 | 18_Acacia mangium Leaf | (3) | CSFF 28277 | T21_B11_N/A_N/A | N/A | TB genotype 18 | *Ca. hongkongensis* | PV757020 | PV758586 | N/A | N/A |
| Hainan | Site 5 | 2/2 | 18_Acacia mangium Leaf | (4) | CSFF 28278 | T19_B30_N/A_N/A | N/A | TB genotype 15 | *Ca. chinensis* | PV757021 | PV758587 | N/A | N/A |
| Hainan | Site 5 | 2/2 | 18_Acacia mangium Leaf | (4) | CSFF 28279 | T21_B13_N/A_N/A | N/A | TB genotype 20 | *Ca. hongkongensis* | PV757022 | PV758588 | N/A | N/A |
| Hainan | Site 5 | 2/2 | 18_Acacia mangium Leaf | (5) | CSFF 28280 | T21_B11_N/A_N/A | N/A | TB genotype 18 | *Ca. hongkongensis* | PV757023 | PV758589 | N/A | N/A |
| Hainan | Site 5 | 2/2 | 18_Acacia mangium Leaf | (5) | CSFF 28281 | T21_B11_N/A_N/A | N/A | TB genotype 18 | *Ca. hongkongensis* | PV757024 | PV758590 | N/A | N/A |
| Hainan | Site 5 | 2/2 | 19_Dianthus caryophyllus Leaf | (1) | CSFF 28222 | T21_B11_H17_C11 | TBHC genotype 20 | TB genotype 18 | *Ca. hongkongensis* | PV756965 | PV758531 | PV757535 | PV757677 |
| Hainan | Site 5 | 2/2 | 19_Dianthus caryophyllus Leaf | (1) | CSFF 28223 | T21_B11_N/A_N/A | N/A | TB genotype 18 | *Ca. hongkongensis* | PV756966 | PV758532 | N/A | N/A |
| Hainan | Site 5 | 2/2 | 19_Dianthus caryophyllus Leaf | (2) | CSFF 28224 | T21_B11_N/A_N/A | N/A | TB genotype 18 | *Ca. hongkongensis* | PV756967 | PV758533 | N/A | N/A |
| Hainan | Site 5 | 2/2 | 19_Dianthus caryophyllus Leaf | (2) | CSFF 28225 | T21_B11_N/A_N/A | N/A | TB genotype 18 | *Ca. hongkongensis* | PV756968 | PV758534 | N/A | N/A |
| Hainan | Site 5 | 2/2 | 19_Dianthus caryophyllus Leaf | (3) | CSFF 28226 | T21_B11_N/A_N/A | N/A | TB genotype 18 | *Ca. hongkongensis* | PV756969 | PV758535 | N/A | N/A |
| Hainan | Site 5 | 2/2 | 19_Dianthus caryophyllus Leaf | (3) | CSFF 28227 | T21_B13_N/A_N/A | N/A | TB genotype 20 | *Ca. hongkongensis* | PV756970 | PV758536 | N/A | N/A |
| Hainan | Site 5 | 2/2 | 19_Dianthus caryophyllus Leaf | (4) | CSFF 28228 | T21_B11_N/A_N/A | N/A | TB genotype 18 | *Ca. hongkongensis* | PV756971 | PV758537 | N/A | N/A |
| Hainan | Site 5 | 2/2 | 19_Dianthus caryophyllus Leaf | (4) | CSFF 28229 | T20_B30_H21_C17 | TBHC genotype 19 | TB genotype 17 | *Ca. chinensis* | PV756972 | PV758538 | PV757536 | PV757678 |
| Hainan | Site 5 | 2/2 | 19_Dianthus caryophyllus Leaf | (5) | CSFF 28230 | T21_B13_N/A_N/A | N/A | TB genotype 20 | *Ca. hongkongensis* | PV756973 | PV758539 | N/A | N/A |
| Hainan | Site 5 | 2/2 | 19_Dianthus caryophyllus Leaf | (5) | CSFF 28231 | T21_B13_N/A_N/A | N/A | TB genotype 20 | *Ca. hongkongensis* | PV756974 | PV758540 | N/A | N/A |
| Hainan | Site 5 | 2/2 | 20_Cinnamomum camphora Leaf | (1) | CSFF 28242 | T20_B30_N/A_N/A | N/A | TB genotype 17 | *Ca. chinensis* | PV756985 | PV758551 | N/A | N/A |
| Hainan | Site 5 | 2/2 | 20_Cinnamomum camphora Leaf | (1) | CSFF 28243 | T20_B30_N/A_N/A | N/A | TB genotype 17 | *Ca. chinensis* | PV756986 | PV758552 | N/A | N/A |
| Hainan | Site 5 | 2/2 | 20_Cinnamomum camphora Leaf | (2) | CSFF 28244 | T19_B30_N/A_N/A | N/A | TB genotype 15 | *Ca. chinensis* | PV756987 | PV758553 | N/A | N/A |
| Hainan | Site 5 | 2/2 | 20_Cinnamomum camphora Leaf | (2) | CSFF 28245 | T19_B30_N/A_N/A | N/A | TB genotype 15 | *Ca. chinensis* | PV756988 | PV758554 | N/A | N/A |
| Hainan | Site 5 | 2/2 | 20_Cinnamomum camphora Leaf | (3) | CSFF 28246 | T21_B11_N/A_N/A | N/A | TB genotype 18 | *Ca. hongkongensis* | PV756989 | PV758555 | N/A | N/A |
| Hainan | Site 5 | 2/2 | 20_Cinnamomum camphora Leaf | (3) | CSFF 28247 | T21_B11_N/A_N/A | N/A | TB genotype 18 | *Ca. hongkongensis* | PV756990 | PV758556 | N/A | N/A |
| Hainan | Site 5 | 2/2 | 20_Cinnamomum camphora Leaf | (4) | CSFF 28248 | T21_B11_N/A_N/A | N/A | TB genotype 18 | *Ca. hongkongensis* | PV756991 | PV758557 | N/A | N/A |
| Hainan | Site 5 | 2/2 | 20_Cinnamomum camphora Leaf | (4) | CSFF 28249 | T21_B11_N/A_N/A | N/A | TB genotype 18 | *Ca. hongkongensis* | PV756992 | PV758558 | N/A | N/A |
| Hainan | Site 5 | 2/2 | 20_Cinnamomum camphora Leaf | (5) | CSFF 28250 | T21_B11_N/A_N/A | N/A | TB genotype 18 | *Ca. hongkongensis* | PV756993 | PV758559 | N/A | N/A |
| Hainan | Site 5 | 2/2 | 20_Cinnamomum camphora Leaf | (5) | CSFF 28251 | T21_B11_N/A_N/A | N/A | TB genotype 18 | *Ca. hongkongensis* | PV756994 | PV758560 | N/A | N/A |
| Hainan | Site 5 | 2/2 | 21_Washingtonia filifera Leaf | (1) | CSFF 28252 | T21_B11_N/A_N/A | N/A | TB genotype 18 | *Ca. hongkongensis* | PV756995 | PV758561 | N/A | N/A |
| Hainan | Site 5 | 2/2 | 21_Washingtonia filifera Leaf | (1) | CSFF 28253 | T21_B11_N/A_N/A | N/A | TB genotype 18 | *Ca. hongkongensis* | PV756996 | PV758562 | N/A | N/A |
| Hainan | Site 5 | 2/2 | 21_Washingtonia filifera Leaf | (2) | CSFF 28254 | T21_B11_N/A_N/A | N/A | TB genotype 18 | *Ca. hongkongensis* | PV756997 | PV758563 | N/A | N/A |
| Hainan | Site 5 | 2/2 | 21_Washingtonia filifera Leaf | (2) | CSFF 28255 | T21_B11_N/A_N/A | N/A | TB genotype 18 | *Ca. hongkongensis* | PV756998 | PV758564 | N/A | N/A |
| Hainan | Site 5 | 2/2 | 21_Washingtonia filifera Leaf | (3) | CSFF 28256 | T21_B11_N/A_N/A | N/A | TB genotype 18 | *Ca. hongkongensis* | PV756999 | PV758565 | N/A | N/A |
| Hainan | Site 5 | 2/2 | 21_Washingtonia filifera Leaf | (3) | CSFF 28257 | T19_B30_N/A_N/A | N/A | TB genotype 15 | *Ca. chinensis* | PV757000 | PV758566 | N/A | N/A |
| Hainan | Site 5 | 2/2 | 21_Washingtonia filifera Leaf | (4) | CSFF 28258 | T21_B12_N/A_N/A | N/A | TB genotype 19 | *Ca. hongkongensis* | PV757001 | PV758567 | N/A | N/A |
| Hainan | Site 5 | 2/2 | 21_Washingtonia filifera Leaf | (4) | CSFF 28259 | T19_B30_N/A_N/A | N/A | TB genotype 15 | *Ca. chinensis* | PV757002 | PV758568 | N/A | N/A |
| Hainan | Site 5 | 2/2 | 21_Washingtonia filifera Leaf | (5) | CSFF 28260 | T21_B11_N/A_N/A | N/A | TB genotype 18 | *Ca. hongkongensis* | PV757003 | PV758569 | N/A | N/A |
| Hainan | Site 5 | 2/2 | 21_Washingtonia filifera Leaf | (5) | CSFF 28261 | T20_B30_N/A_N/A | N/A | TB genotype 17 | *Ca. chinensis* | PV757004 | PV758570 | N/A | N/A |
| Hainan | Site 5 | 2/2 | 22_Ficus benjamina Leaf | (1) | CSFF 28290 | T21_B11_N/A_N/A | N/A | TB genotype 18 | *Ca. hongkongensis* | PV757033 | PV758599 | N/A | N/A |
| Hainan | Site 5 | 2/2 | 22_Ficus benjamina Leaf | (1) | CSFF 28291 | T21_B11_N/A_N/A | N/A | TB genotype 18 | *Ca. hongkongensis* | PV757034 | PV758600 | N/A | N/A |
| Hainan | Site 5 | 2/2 | 22_Ficus benjamina Leaf | (2) | CSFF 28292 | T21_B12_H17_C11 | TBHC genotype 21 | TB genotype 19 | *Ca. hongkongensis* | PV757035 | PV758601 | PV757540 | PV757682 |
| Hainan | Site 5 | 2/2 | 22_Ficus benjamina Leaf | (2) | CSFF 28293 | T21_B11_N/A_N/A | N/A | TB genotype 18 | *Ca. hongkongensis* | PV757036 | PV758602 | N/A | N/A |
| Hainan | Site 5 | 2/2 | 22_Ficus benjamina Leaf | (3) | CSFF 28294 | T21_B13_N/A_N/A | N/A | TB genotype 20 | *Ca. hongkongensis* | PV757037 | PV758603 | N/A | N/A |
| Hainan | Site 5 | 2/2 | 22_Ficus benjamina Leaf | (3) | CSFF 28295 | T21_B11_N/A_N/A | N/A | TB genotype 18 | *Ca. hongkongensis* | PV757038 | PV758604 | N/A | N/A |
| Hainan | Site 5 | 2/2 | 22_Ficus benjamina Leaf | (4) | CSFF 28296 | T21_B11_N/A_N/A | N/A | TB genotype 18 | *Ca. hongkongensis* | PV757039 | PV758605 | N/A | N/A |
| Hainan | Site 5 | 2/2 | 22_Ficus benjamina Leaf | (4) | CSFF 28297 | T21_B11_N/A_N/A | N/A | TB genotype 18 | *Ca. hongkongensis* | PV757040 | PV758606 | N/A | N/A |
| Hainan | Site 5 | 2/2 | 22_Ficus benjamina Leaf | (5) | CSFF 28298 | T21_B13_N/A_N/A | N/A | TB genotype 20 | *Ca. hongkongensis* | PV757041 | PV758607 | N/A | N/A |
| Hainan | Site 5 | 2/2 | 22_Ficus benjamina Leaf | (5) | CSFF 28299 | T21_B13_N/A_N/A | N/A | TB genotype 20 | *Ca. hongkongensis* | PV757042 | PV758608 | N/A | N/A |
| Hainan | Site 5 | 2/2 | 23_Rhododendron pulchrum Leaf | (1) | CSFF 28314 | T21_B11_N/A_N/A | N/A | TB genotype 18 | *Ca. hongkongensis* | PV757057 | PV758623 | N/A | N/A |
| Hainan | Site 5 | 2/2 | 23_Rhododendron pulchrum Leaf | (1) | CSFF 28315 | T21_B11_N/A_N/A | N/A | TB genotype 18 | *Ca. hongkongensis* | PV757058 | PV758624 | N/A | N/A |
| Hainan | Site 5 | 2/2 | 23_Rhododendron pulchrum Leaf | (2) | CSFF 28316 | T21_B11_N/A_N/A | N/A | TB genotype 18 | *Ca. hongkongensis* | PV757059 | PV758625 | N/A | N/A |
| Hainan | Site 5 | 2/2 | 23_Rhododendron pulchrum Leaf | (2) | CSFF 28317 | T21_B11_N/A_N/A | N/A | TB genotype 18 | *Ca. hongkongensis* | PV757060 | PV758626 | N/A | N/A |
| Hainan | Site 5 | 2/2 | 23_Rhododendron pulchrum Leaf | (3) | CSFF 28318 | T21_B11_N/A_N/A | N/A | TB genotype 18 | *Ca. hongkongensis* | PV757061 | PV758627 | N/A | N/A |
| Hainan | Site 5 | 2/2 | 23_Rhododendron pulchrum Leaf | (3) | CSFF 28319 | T21_B11_N/A_N/A | N/A | TB genotype 18 | *Ca. hongkongensis* | PV757062 | PV758628 | N/A | N/A |
| Hainan | Site 5 | 2/2 | 23_Rhododendron pulchrum Leaf | (4) | CSFF 28320 | T21_B11_N/A_N/A | N/A | TB genotype 18 | *Ca. hongkongensis* | PV757063 | PV758629 | N/A | N/A |
| Hainan | Site 5 | 2/2 | 23_Rhododendron pulchrum Leaf | (4) | CSFF 28321 | T21_B11_N/A_N/A | N/A | TB genotype 18 | *Ca. hongkongensis* | PV757064 | PV758630 | N/A | N/A |
| Hainan | Site 5 | 2/2 | 23_Rhododendron pulchrum Leaf | (5) | CSFF 28322 | T21_B11_N/A_N/A | N/A | TB genotype 18 | *Ca. hongkongensis* | PV757065 | PV758631 | N/A | N/A |
| Hainan | Site 5 | 2/2 | 23_Rhododendron pulchrum Leaf | (5) | CSFF 28323 | T21_B11_N/A_N/A | N/A | TB genotype 18 | *Ca. hongkongensis* | PV757066 | PV758632 | N/A | N/A |
| Hainan | Site 5 | 2/2 | 26_Medicago sativa Seed | (1) | CSFF 28348 | T21_B11_H17_C11 | TBHC genotype 20 | TB genotype 18 | *Ca. hongkongensis* | PV757091 | PV758657 | PV757545 | PV757687 |
| Hainan | Site 5 | 2/2 | 26_Medicago sativa Seed | (1) | CSFF 28349 | T21_B11_N/A_N/A | N/A | TB genotype 18 | *Ca. hongkongensis* | PV757092 | PV758658 | N/A | N/A |
| Hainan | Site 5 | 2/2 | 26_Medicago sativa Seed | (2) | CSFF 28350 | T21_B13_H17_C11 | TBHC genotype 23 | TB genotype 20 | *Ca. hongkongensis* | PV757093 | PV758659 | PV757546 | PV757688 |
| Hainan | Site 5 | 2/2 | 26_Medicago sativa Seed | (2) | CSFF 28351 | T21_B13_N/A_N/A | N/A | TB genotype 20 | *Ca. hongkongensis* | PV757094 | PV758660 | N/A | N/A |
| Hainan | Site 5 | 2/2 | 26_Medicago sativa Seed | (3) | CSFF 28352 | T21_B11_N/A_N/A | N/A | TB genotype 18 | *Ca. hongkongensis* | PV757095 | PV758661 | N/A | N/A |
| Hainan | Site 5 | 2/2 | 26_Medicago sativa Seed | (3) | CSFF 28353 | T21_B13_N/A_N/A | N/A | TB genotype 20 | *Ca. hongkongensis* | PV757096 | PV758662 | N/A | N/A |
| Hainan | Site 5 | 2/2 | 26_Medicago sativa Seed | (4) | CSFF 28354 | T21_B13_N/A_N/A | N/A | TB genotype 20 | *Ca. hongkongensis* | PV757097 | PV758663 | N/A | N/A |
| Hainan | Site 5 | 2/2 | 26_Medicago sativa Seed | (4) | CSFF 28355 | T21_B13_N/A_N/A | N/A | TB genotype 20 | *Ca. hongkongensis* | PV757098 | PV758664 | N/A | N/A |
| Hainan | Site 5 | 2/2 | 26_Medicago sativa Seed | (5) | CSFF 28356 | T20_B30_H21_C17 | TBHC genotype 19 | TB genotype 17 | *Ca. chinensis* | PV757099 | PV758665 | PV757547 | PV757689 |
| Hainan | Site 5 | 2/2 | 26_Medicago sativa Seed | (5) | CSFF 28357 | T20_B30_N/A_N/A | N/A | TB genotype 17 | *Ca. chinensis* | PV757100 | PV758666 | N/A | N/A |
| Heilongjiang | Site 6 | 1/1 | 1_Brassica rapa subsp. pekinensis Seed | (2) | CSFF 31520 | T18_B36_N/A_N/A | N/A | TB genotype 12 | *Ca. canadiana* | PV757207 | PV758773 | N/A | N/A |
| Heilongjiang | Site 6 | 1/1 | 1_Brassica rapa subsp. pekinensis Seed | (2) | CSFF 31521 | T18_B36_N/A_N/A | N/A | TB genotype 12 | *Ca. canadiana* | PV757208 | PV758774 | N/A | N/A |
| Heilongjiang | Site 6 | 1/1 | 3_Lolium perenne Seed | (1) | CSFF 31538 | T18_B36_N/A_N/A | N/A | TB genotype 12 | *Ca. canadiana* | PV757225 | PV758791 | N/A | N/A |
| Heilongjiang | Site 6 | 1/1 | 3_Lolium perenne Seed | (1) | CSFF 31539 | T18_B36_N/A_N/A | N/A | TB genotype 12 | *Ca. canadiana* | PV757226 | PV758792 | N/A | N/A |
| Heilongjiang | Site 6 | 1/1 | 4_Salvia hispanica Seed | (1) | CSFF 31522 | T18_B36_N/A_N/A | N/A | TB genotype 12 | *Ca. canadiana* | PV757209 | PV758775 | N/A | N/A |
| Heilongjiang | Site 6 | 1/1 | 4_Salvia hispanica Seed | (1) | CSFF 31523 | T18_B36_N/A_N/A | N/A | TB genotype 12 | *Ca. canadiana* | PV757210 | PV758776 | N/A | N/A |
| Heilongjiang | Site 6 | 1/1 | 4_Salvia hispanica Seed | (2) | CSFF 31524 | T18_B36_N/A_N/A | N/A | TB genotype 12 | *Ca. canadiana* | PV757211 | PV758777 | N/A | N/A |
| Heilongjiang | Site 6 | 1/1 | 4_Salvia hispanica Seed | (2) | CSFF 31525 | T18_B36_N/A_N/A | N/A | TB genotype 12 | *Ca. canadiana* | PV757212 | PV758778 | N/A | N/A |
| Heilongjiang | Site 6 | 1/1 | 4_Salvia hispanica Seed | (4) | CSFF 31526 | T18_B36_N/A_N/A | N/A | TB genotype 12 | *Ca. canadiana* | PV757213 | PV758779 | N/A | N/A |
| Heilongjiang | Site 6 | 1/1 | 4_Salvia hispanica Seed | (4) | CSFF 31527 | T18_B36_N/A_N/A | N/A | TB genotype 12 | *Ca. canadiana* | PV757214 | PV758780 | N/A | N/A |
| Heilongjiang | Site 6 | 1/1 | 4_Salvia hispanica Seed | (5) | CSFF 31528 | T18_B36_N/A_N/A | N/A | TB genotype 12 | *Ca. canadiana* | PV757215 | PV758781 | N/A | N/A |
| Heilongjiang | Site 6 | 1/1 | 4_Salvia hispanica Seed | (5) | CSFF 31529 | T18_B36_N/A_N/A | N/A | TB genotype 12 | *Ca. canadiana* | PV757216 | PV758782 | N/A | N/A |
| Heilongjiang | Site 6 | 1/1 | 5_Impatiens balsamina Seed | (1) | CSFF 31530 | T18_B36_N/A_N/A | N/A | TB genotype 12 | *Ca. canadiana* | PV757217 | PV758783 | N/A | N/A |
| Heilongjiang | Site 6 | 1/1 | 5_Impatiens balsamina Seed | (1) | CSFF 31531 | T18_B36_N/A_N/A | N/A | TB genotype 12 | *Ca. canadiana* | PV757218 | PV758784 | N/A | N/A |
| Heilongjiang | Site 6 | 1/1 | 5_Impatiens balsamina Seed | (2) | CSFF 31532 | T18_B36_N/A_N/A | N/A | TB genotype 12 | *Ca. canadiana* | PV757219 | PV758785 | N/A | N/A |
| Heilongjiang | Site 6 | 1/1 | 5_Impatiens balsamina Seed | (2) | CSFF 31533 | T18_B36_N/A_N/A | N/A | TB genotype 12 | *Ca. canadiana* | PV757220 | PV758786 | N/A | N/A |
| Heilongjiang | Site 6 | 1/1 | 5_Impatiens balsamina Seed | (3) | CSFF 31534 | T18_B36_N/A_N/A | N/A | TB genotype 12 | *Ca. canadiana* | PV757221 | PV758787 | N/A | N/A |
| Heilongjiang | Site 6 | 1/1 | 5_Impatiens balsamina Seed | (3) | CSFF 31535 | T18_B36_N/A_N/A | N/A | TB genotype 12 | *Ca. canadiana* | PV757222 | PV758788 | N/A | N/A |
| Heilongjiang | Site 6 | 1/1 | 5_Impatiens balsamina Seed | (5) | CSFF 31536 | T18_B36_N/A_N/A | N/A | TB genotype 12 | *Ca. canadiana* | PV757223 | PV758789 | N/A | N/A |
| Heilongjiang | Site 6 | 1/1 | 5_Impatiens balsamina Seed | (5) | CSFF 31537 | T18_B36_N/A_N/A | N/A | TB genotype 12 | *Ca. canadiana* | PV757224 | PV758790 | N/A | N/A |
| Heilongjiang | Site 6 | 1/1 | 6_Sesamum indicum Seed | (1) | CSFF 31506 | T18_B36_N/A_N/A | N/A | TB genotype 12 | *Ca. canadiana* | PV757193 | PV758759 | N/A | N/A |
| Heilongjiang | Site 6 | 1/1 | 6_Sesamum indicum Seed | (1) | CSFF 31507 | T18_B36_N/A_N/A | N/A | TB genotype 12 | *Ca. canadiana* | PV757194 | PV758760 | N/A | N/A |
| Heilongjiang | Site 6 | 1/1 | 6_Sesamum indicum Seed | (5) | CSFF 31508 | T18_B36_N/A_N/A | N/A | TB genotype 12 | *Ca. canadiana* | PV757195 | PV758761 | N/A | N/A |
| Heilongjiang | Site 6 | 1/1 | 6_Sesamum indicum Seed | (5) | CSFF 31509 | T18_B36_N/A_N/A | N/A | TB genotype 12 | *Ca. canadiana* | PV757196 | PV758762 | N/A | N/A |
| Heilongjiang | Site 6 | 1/1 | 7_Dianthus caryophyllus Seed | (1) | CSFF 31510 | T18_B36_N/A_N/A | N/A | TB genotype 12 | *Ca. canadiana* | PV757197 | PV758763 | N/A | N/A |
| Heilongjiang | Site 6 | 1/1 | 7_Dianthus caryophyllus Seed | (1) | CSFF 31511 | T18_B36_N/A_N/A | N/A | TB genotype 12 | *Ca. canadiana* | PV757198 | PV758764 | N/A | N/A |
| Heilongjiang | Site 6 | 1/1 | 7_Dianthus caryophyllus Seed | (2) | CSFF 31512 | T18_B36_N/A_N/A | N/A | TB genotype 12 | *Ca. canadiana* | PV757199 | PV758765 | N/A | N/A |
| Heilongjiang | Site 6 | 1/1 | 7_Dianthus caryophyllus Seed | (2) | CSFF 31513 | T18_B36_N/A_N/A | N/A | TB genotype 12 | *Ca. canadiana* | PV757200 | PV758766 | N/A | N/A |
| Heilongjiang | Site 6 | 1/1 | 7_Dianthus caryophyllus Seed | (3) | CSFF 31514 | T18_B36_N/A_N/A | N/A | TB genotype 12 | *Ca. canadiana* | PV757201 | PV758767 | N/A | N/A |
| Heilongjiang | Site 6 | 1/1 | 7_Dianthus caryophyllus Seed | (3) | CSFF 31515 | T18_B36_N/A_N/A | N/A | TB genotype 12 | *Ca. canadiana* | PV757202 | PV758768 | N/A | N/A |
| Heilongjiang | Site 6 | 1/1 | 7_Dianthus caryophyllus Seed | (4) | CSFF 31516 | T18_B36_N/A_N/A | N/A | TB genotype 12 | *Ca. canadiana* | PV757203 | PV758769 | N/A | N/A |
| Heilongjiang | Site 6 | 1/1 | 7_Dianthus caryophyllus Seed | (4) | CSFF 31517 | T18_B36_N/A_N/A | N/A | TB genotype 12 | *Ca. canadiana* | PV757204 | PV758770 | N/A | N/A |
| Heilongjiang | Site 6 | 1/1 | 7_Dianthus caryophyllus Seed | (5) | CSFF 31518 | T18_B36_N/A_N/A | N/A | TB genotype 12 | *Ca. canadiana* | PV757205 | PV758771 | N/A | N/A |
| Heilongjiang | Site 6 | 1/1 | 7_Dianthus caryophyllus Seed | (5) | CSFF 31519 | T18_B36_N/A_N/A | N/A | TB genotype 12 | *Ca. canadiana* | PV757206 | PV758772 | N/A | N/A |
| Heilongjiang | Site 6 | 1/1 | 8_Trifolium pratense Seed | (1) | CSFF 31496 | T18_B36_N/A_N/A | N/A | TB genotype 12 | *Ca. canadiana* | PV757183 | PV758749 | N/A | N/A |
| Heilongjiang | Site 6 | 1/1 | 8_Trifolium pratense Seed | (1) | CSFF 31497 | T18_B36_N/A_N/A | N/A | TB genotype 12 | *Ca. canadiana* | PV757184 | PV758750 | N/A | N/A |
| Heilongjiang | Site 6 | 1/1 | 8_Trifolium pratense Seed | (2) | CSFF 31498 | T18_B36_N/A_N/A | N/A | TB genotype 12 | *Ca. canadiana* | PV757185 | PV758751 | N/A | N/A |
| Heilongjiang | Site 6 | 1/1 | 8_Trifolium pratense Seed | (2) | CSFF 31499 | T18_B36_N/A_N/A | N/A | TB genotype 12 | *Ca. canadiana* | PV757186 | PV758752 | N/A | N/A |
| Heilongjiang | Site 6 | 1/1 | 8_Trifolium pratense Seed | (3) | CSFF 31500 | T18_B36_N/A_N/A | N/A | TB genotype 12 | *Ca. canadiana* | PV757187 | PV758753 | N/A | N/A |
| Heilongjiang | Site 6 | 1/1 | 8_Trifolium pratense Seed | (3) | CSFF 31501 | T18_B36_N/A_N/A | N/A | TB genotype 12 | *Ca. canadiana* | PV757188 | PV758754 | N/A | N/A |
| Heilongjiang | Site 6 | 1/1 | 8_Trifolium pratense Seed | (4) | CSFF 31502 | T18_B36_N/A_N/A | N/A | TB genotype 12 | *Ca. canadiana* | PV757189 | PV758755 | N/A | N/A |
| Heilongjiang | Site 6 | 1/1 | 8_Trifolium pratense Seed | (4) | CSFF 31503 | T18_B36_N/A_N/A | N/A | TB genotype 12 | *Ca. canadiana* | PV757190 | PV758756 | N/A | N/A |
| Heilongjiang | Site 6 | 1/1 | 8_Trifolium pratense Seed | (5) | CSFF 31504 | T18_B36_N/A_N/A | N/A | TB genotype 12 | *Ca. canadiana* | PV757191 | PV758757 | N/A | N/A |
| Heilongjiang | Site 6 | 1/1 | 8_Trifolium pratense Seed | (5) | CSFF 31505 | T18_B36_N/A_N/A | N/A | TB genotype 12 | *Ca. canadiana* | PV757192 | PV758758 | N/A | N/A |
| Heilongjiang | Site 6 | 1/1 | 9_Lythrum salicaria Seed | (1) | CSFF 31486 | T18_B36_H14_C18 | TBHC genotype 13 | TB genotype 12 | *Ca. canadiana* | PV757173 | PV758739 | PV757556 | PV757698 |
| Heilongjiang | Site 6 | 1/1 | 9_Lythrum salicaria Seed | (1) | CSFF 31487 | T18_B36_N/A_N/A | N/A | TB genotype 12 | *Ca. canadiana* | PV757174 | PV758740 | N/A | N/A |
| Heilongjiang | Site 6 | 1/1 | 9_Lythrum salicaria Seed | (2) | CSFF 31488 | T18_B36_N/A_N/A | N/A | TB genotype 12 | *Ca. canadiana* | PV757175 | PV758741 | N/A | N/A |
| Heilongjiang | Site 6 | 1/1 | 9_Lythrum salicaria Seed | (2) | CSFF 31489 | T18_B36_N/A_N/A | N/A | TB genotype 12 | *Ca. canadiana* | PV757176 | PV758742 | N/A | N/A |
| Heilongjiang | Site 6 | 1/1 | 9_Lythrum salicaria Seed | (3) | CSFF 31490 | T18_B36_N/A_N/A | N/A | TB genotype 12 | *Ca. canadiana* | PV757177 | PV758743 | N/A | N/A |
| Heilongjiang | Site 6 | 1/1 | 9_Lythrum salicaria Seed | (3) | CSFF 31491 | T18_B36_N/A_N/A | N/A | TB genotype 12 | *Ca. canadiana* | PV757178 | PV758744 | N/A | N/A |
| Heilongjiang | Site 6 | 1/1 | 9_Lythrum salicaria Seed | (4) | CSFF 31492 | T18_B36_N/A_N/A | N/A | TB genotype 12 | *Ca. canadiana* | PV757179 | PV758745 | N/A | N/A |
| Heilongjiang | Site 6 | 1/1 | 9_Lythrum salicaria Seed | (4) | CSFF 31493 | T18_B36_N/A_N/A | N/A | TB genotype 12 | *Ca. canadiana* | PV757180 | PV758746 | N/A | N/A |
| Heilongjiang | Site 6 | 1/1 | 9_Lythrum salicaria Seed | (5) | CSFF 31494 | T18_B36_N/A_N/A | N/A | TB genotype 12 | *Ca. canadiana* | PV757181 | PV758747 | N/A | N/A |
| Heilongjiang | Site 6 | 1/1 | 9_Lythrum salicaria Seed | (5) | CSFF 31495 | T18_B36_N/A_N/A | N/A | TB genotype 12 | *Ca. canadiana* | PV757182 | PV758748 | N/A | N/A |
| Heilongjiang | Site 6 | 1/1 | 10_Buxus sinica Leaf | (2) | CSFF 31636 | T18_B36_N/A_N/A | N/A | TB genotype 12 | *Ca. canadiana* | PV757323 | PV758889 | N/A | N/A |
| Heilongjiang | Site 6 | 1/1 | 10_Buxus sinica Leaf | (2) | CSFF 31637 | T18_B36_N/A_N/A | N/A | TB genotype 12 | *Ca. canadiana* | PV757324 | PV758890 | N/A | N/A |
| Heilongjiang | Site 6 | 1/1 | 10_Buxus sinica Leaf | (4) | CSFF 31638 | T18_B36_N/A_N/A | N/A | TB genotype 12 | *Ca. canadiana* | PV757325 | PV758891 | N/A | N/A |
| Heilongjiang | Site 6 | 1/1 | 10_Buxus sinica Leaf | (4) | CSFF 31639 | T18_B36_N/A_N/A | N/A | TB genotype 12 | *Ca. canadiana* | PV757326 | PV758892 | N/A | N/A |
| Heilongjiang | Site 6 | 1/1 | 11_Imperata cylindrica Leaf | (3) | CSFF 31620 | T18_B36_N/A_N/A | N/A | TB genotype 12 | *Ca. canadiana* | PV757307 | PV758873 | N/A | N/A |
| Heilongjiang | Site 6 | 1/1 | 11_Imperata cylindrica Leaf | (3) | CSFF 31621 | T18_B36_N/A_N/A | N/A | TB genotype 12 | *Ca. canadiana* | PV757308 | PV758874 | N/A | N/A |
| Heilongjiang | Site 6 | 1/1 | 11_Imperata cylindrica Leaf | (5) | CSFF 31622 | T18_B36_N/A_N/A | N/A | TB genotype 12 | *Ca. canadiana* | PV757309 | PV758875 | N/A | N/A |
| Heilongjiang | Site 6 | 1/1 | 11_Imperata cylindrica Leaf | (5) | CSFF 31623 | T18_B36_N/A_N/A | N/A | TB genotype 12 | *Ca. canadiana* | PV757310 | PV758876 | N/A | N/A |
| Heilongjiang | Site 6 | 1/1 | 12_Pinus massoniana Leaf | (4) | CSFF 31624 | T18_B36_N/A_N/A | N/A | TB genotype 12 | *Ca. canadiana* | PV757311 | PV758877 | N/A | N/A |
| Heilongjiang | Site 6 | 1/1 | 12_Pinus massoniana Leaf | (4) | CSFF 31625 | T18_B36_N/A_N/A | N/A | TB genotype 12 | *Ca. canadiana* | PV757312 | PV758878 | N/A | N/A |
| Heilongjiang | Site 6 | 1/1 | 13_Eucalyptus urophylla hybrid Leaf | (1) | CSFF 31646 | T18_B36_H14_C18 | TBHC genotype 13 | TB genotype 12 | *Ca. canadiana* | PV757333 | PV758899 | PV757563 | PV757705 |
| Heilongjiang | Site 6 | 1/1 | 13_Eucalyptus urophylla hybrid Leaf | (1) | CSFF 31647 | T18_B36_N/A_N/A | N/A | TB genotype 12 | *Ca. canadiana* | PV757334 | PV758900 | N/A | N/A |
| Heilongjiang | Site 6 | 1/1 | 13_Eucalyptus urophylla hybrid Leaf | (2) | CSFF 31648 | T18_B36_N/A_N/A | N/A | TB genotype 12 | *Ca. canadiana* | PV757335 | PV758901 | N/A | N/A |
| Heilongjiang | Site 6 | 1/1 | 13_Eucalyptus urophylla hybrid Leaf | (2) | CSFF 31649 | T18_B36_N/A_N/A | N/A | TB genotype 12 | *Ca. canadiana* | PV757336 | PV758902 | N/A | N/A |
| Heilongjiang | Site 6 | 1/1 | 13_Eucalyptus urophylla hybrid Leaf | (3) | CSFF 31650 | T18_B36_N/A_N/A | N/A | TB genotype 12 | *Ca. canadiana* | PV757337 | PV758903 | N/A | N/A |
| Heilongjiang | Site 6 | 1/1 | 13_Eucalyptus urophylla hybrid Leaf | (3) | CSFF 31651 | T18_B36_N/A_N/A | N/A | TB genotype 12 | *Ca. canadiana* | PV757338 | PV758904 | N/A | N/A |
| Heilongjiang | Site 6 | 1/1 | 13_Eucalyptus urophylla hybrid Leaf | (4) | CSFF 31652 | T18_B36_N/A_N/A | N/A | TB genotype 12 | *Ca. canadiana* | PV757339 | PV758905 | N/A | N/A |
| Heilongjiang | Site 6 | 1/1 | 13_Eucalyptus urophylla hybrid Leaf | (4) | CSFF 31653 | T18_B36_N/A_N/A | N/A | TB genotype 12 | *Ca. canadiana* | PV757340 | PV758906 | N/A | N/A |
| Heilongjiang | Site 6 | 1/1 | 13_Eucalyptus urophylla hybrid Leaf | (5) | CSFF 31654 | T18_B36_N/A_N/A | N/A | TB genotype 12 | *Ca. canadiana* | PV757341 | PV758907 | N/A | N/A |
| Heilongjiang | Site 6 | 1/1 | 13_Eucalyptus urophylla hybrid Leaf | (5) | CSFF 31655 | T18_B36_N/A_N/A | N/A | TB genotype 12 | *Ca. canadiana* | PV757342 | PV758908 | N/A | N/A |
| Heilongjiang | Site 6 | 1/1 | 14_Syzygium jambos Leaf | (1) | CSFF 31600 | T18_B36_N/A_N/A | N/A | TB genotype 12 | *Ca. canadiana* | PV757287 | PV758853 | N/A | N/A |
| Heilongjiang | Site 6 | 1/1 | 14_Syzygium jambos Leaf | (1) | CSFF 31601 | T18_B36_N/A_N/A | N/A | TB genotype 12 | *Ca. canadiana* | PV757288 | PV758854 | N/A | N/A |
| Heilongjiang | Site 6 | 1/1 | 14_Syzygium jambos Leaf | (2) | CSFF 31602 | T18_B36_N/A_N/A | N/A | TB genotype 12 | *Ca. canadiana* | PV757289 | PV758855 | N/A | N/A |
| Heilongjiang | Site 6 | 1/1 | 14_Syzygium jambos Leaf | (2) | CSFF 31603 | T18_B36_N/A_N/A | N/A | TB genotype 12 | *Ca. canadiana* | PV757290 | PV758856 | N/A | N/A |
| Heilongjiang | Site 6 | 1/1 | 14_Syzygium jambos Leaf | (3) | CSFF 31604 | T18_B36_N/A_N/A | N/A | TB genotype 12 | *Ca. canadiana* | PV757291 | PV758857 | N/A | N/A |
| Heilongjiang | Site 6 | 1/1 | 14_Syzygium jambos Leaf | (3) | CSFF 31605 | T18_B36_N/A_N/A | N/A | TB genotype 12 | *Ca. canadiana* | PV757292 | PV758858 | N/A | N/A |
| Heilongjiang | Site 6 | 1/1 | 14_Syzygium jambos Leaf | (4) | CSFF 31606 | T18_B36_N/A_N/A | N/A | TB genotype 12 | *Ca. canadiana* | PV757293 | PV758859 | N/A | N/A |
| Heilongjiang | Site 6 | 1/1 | 14_Syzygium jambos Leaf | (4) | CSFF 31607 | T18_B36_N/A_N/A | N/A | TB genotype 12 | *Ca. canadiana* | PV757294 | PV758860 | N/A | N/A |
| Heilongjiang | Site 6 | 1/1 | 14_Syzygium jambos Leaf | (5) | CSFF 31608 | T18_B36_N/A_N/A | N/A | TB genotype 12 | *Ca. canadiana* | PV757295 | PV758861 | N/A | N/A |
| Heilongjiang | Site 6 | 1/1 | 14_Syzygium jambos Leaf | (5) | CSFF 31609 | T18_B36_N/A_N/A | N/A | TB genotype 12 | *Ca. canadiana* | PV757296 | PV758862 | N/A | N/A |
| Heilongjiang | Site 6 | 1/1 | 15_Pennisetum purpureum Leaf | (1) | CSFF 31656 | T18_B36_N/A_N/A | N/A | TB genotype 12 | *Ca. canadiana* | PV757343 | PV758909 | N/A | N/A |
| Heilongjiang | Site 6 | 1/1 | 15_Pennisetum purpureum Leaf | (1) | CSFF 31657 | T18_B36_N/A_N/A | N/A | TB genotype 12 | *Ca. canadiana* | PV757344 | PV758910 | N/A | N/A |
| Heilongjiang | Site 6 | 1/1 | 15_Pennisetum purpureum Leaf | (2) | CSFF 31658 | T18_B36_N/A_N/A | N/A | TB genotype 12 | *Ca. canadiana* | PV757345 | PV758911 | N/A | N/A |
| Heilongjiang | Site 6 | 1/1 | 15_Pennisetum purpureum Leaf | (2) | CSFF 31659 | T18_B36_N/A_N/A | N/A | TB genotype 12 | *Ca. canadiana* | PV757346 | PV758912 | N/A | N/A |
| Heilongjiang | Site 6 | 1/1 | 15_Pennisetum purpureum Leaf | (3) | CSFF 31660 | T18_B36_N/A_N/A | N/A | TB genotype 12 | *Ca. canadiana* | PV757347 | PV758913 | N/A | N/A |
| Heilongjiang | Site 6 | 1/1 | 15_Pennisetum purpureum Leaf | (3) | CSFF 31661 | T18_B36_N/A_N/A | N/A | TB genotype 12 | *Ca. canadiana* | PV757348 | PV758914 | N/A | N/A |
| Heilongjiang | Site 6 | 1/1 | 15_Pennisetum purpureum Leaf | (4) | CSFF 31662 | T18_B36_N/A_N/A | N/A | TB genotype 12 | *Ca. canadiana* | PV757349 | PV758915 | N/A | N/A |
| Heilongjiang | Site 6 | 1/1 | 15_Pennisetum purpureum Leaf | (4) | CSFF 31663 | T18_B36_N/A_N/A | N/A | TB genotype 12 | *Ca. canadiana* | PV757350 | PV758916 | N/A | N/A |
| Heilongjiang | Site 6 | 1/1 | 15_Pennisetum purpureum Leaf | (5) | CSFF 31664 | T18_B36_N/A_N/A | N/A | TB genotype 12 | *Ca. canadiana* | PV757351 | PV758917 | N/A | N/A |
| Heilongjiang | Site 6 | 1/1 | 15_Pennisetum purpureum Leaf | (5) | CSFF 31665 | T18_B36_N/A_N/A | N/A | TB genotype 12 | *Ca. canadiana* | PV757352 | PV758918 | N/A | N/A |
| Heilongjiang | Site 6 | 1/1 | 16_Saccharum officinarum Leaf | (1) | CSFF 31580 | T18_B36_N/A_N/A | N/A | TB genotype 12 | *Ca. canadiana* | PV757267 | PV758833 | N/A | N/A |
| Heilongjiang | Site 6 | 1/1 | 16_Saccharum officinarum Leaf | (1) | CSFF 31581 | T18_B36_N/A_N/A | N/A | TB genotype 12 | *Ca. canadiana* | PV757268 | PV758834 | N/A | N/A |
| Heilongjiang | Site 6 | 1/1 | 16_Saccharum officinarum Leaf | (2) | CSFF 31582 | T18_B36_N/A_N/A | N/A | TB genotype 12 | *Ca. canadiana* | PV757269 | PV758835 | N/A | N/A |
| Heilongjiang | Site 6 | 1/1 | 16_Saccharum officinarum Leaf | (2) | CSFF 31583 | T18_B36_N/A_N/A | N/A | TB genotype 12 | *Ca. canadiana* | PV757270 | PV758836 | N/A | N/A |
| Heilongjiang | Site 6 | 1/1 | 16_Saccharum officinarum Leaf | (3) | CSFF 31584 | T18_B36_N/A_N/A | N/A | TB genotype 12 | *Ca. canadiana* | PV757271 | PV758837 | N/A | N/A |
| Heilongjiang | Site 6 | 1/1 | 16_Saccharum officinarum Leaf | (3) | CSFF 31585 | T18_B36_N/A_N/A | N/A | TB genotype 12 | *Ca. canadiana* | PV757272 | PV758838 | N/A | N/A |
| Heilongjiang | Site 6 | 1/1 | 16_Saccharum officinarum Leaf | (4) | CSFF 31586 | T18_B36_N/A_N/A | N/A | TB genotype 12 | *Ca. canadiana* | PV757273 | PV758839 | N/A | N/A |
| Heilongjiang | Site 6 | 1/1 | 16_Saccharum officinarum Leaf | (4) | CSFF 31587 | T18_B36_N/A_N/A | N/A | TB genotype 12 | *Ca. canadiana* | PV757274 | PV758840 | N/A | N/A |
| Heilongjiang | Site 6 | 1/1 | 16_Saccharum officinarum Leaf | (5) | CSFF 31588 | T18_B36_N/A_N/A | N/A | TB genotype 12 | *Ca. canadiana* | PV757275 | PV758841 | N/A | N/A |
| Heilongjiang | Site 6 | 1/1 | 16_Saccharum officinarum Leaf | (5) | CSFF 31589 | T18_B36_N/A_N/A | N/A | TB genotype 12 | *Ca. canadiana* | PV757276 | PV758842 | N/A | N/A |
| Heilongjiang | Site 6 | 1/1 | 17_Rosa chinensis Leaf | (1) | CSFF 31550 | T18_B36_N/A_N/A | N/A | TB genotype 12 | *Ca. canadiana* | PV757237 | PV758803 | N/A | N/A |
| Heilongjiang | Site 6 | 1/1 | 17_Rosa chinensis Leaf | (1) | CSFF 31551 | T18_B36_N/A_N/A | N/A | TB genotype 12 | *Ca. canadiana* | PV757238 | PV758804 | N/A | N/A |
| Heilongjiang | Site 6 | 1/1 | 17_Rosa chinensis Leaf | (2) | CSFF 31552 | T18_B36_N/A_N/A | N/A | TB genotype 12 | *Ca. canadiana* | PV757239 | PV758805 | N/A | N/A |
| Heilongjiang | Site 6 | 1/1 | 17_Rosa chinensis Leaf | (2) | CSFF 31553 | T18_B36_N/A_N/A | N/A | TB genotype 12 | *Ca. canadiana* | PV757240 | PV758806 | N/A | N/A |
| Heilongjiang | Site 6 | 1/1 | 17_Rosa chinensis Leaf | (3) | CSFF 31554 | T18_B36_N/A_N/A | N/A | TB genotype 12 | *Ca. canadiana* | PV757241 | PV758807 | N/A | N/A |
| Heilongjiang | Site 6 | 1/1 | 17_Rosa chinensis Leaf | (3) | CSFF 31555 | T18_B36_N/A_N/A | N/A | TB genotype 12 | *Ca. canadiana* | PV757242 | PV758808 | N/A | N/A |
| Heilongjiang | Site 6 | 1/1 | 17_Rosa chinensis Leaf | (4) | CSFF 31556 | T18_B36_N/A_N/A | N/A | TB genotype 12 | *Ca. canadiana* | PV757243 | PV758809 | N/A | N/A |
| Heilongjiang | Site 6 | 1/1 | 17_Rosa chinensis Leaf | (4) | CSFF 31557 | T18_B36_N/A_N/A | N/A | TB genotype 12 | *Ca. canadiana* | PV757244 | PV758810 | N/A | N/A |
| Heilongjiang | Site 6 | 1/1 | 17_Rosa chinensis Leaf | (5) | CSFF 31558 | T18_B36_N/A_N/A | N/A | TB genotype 12 | *Ca. canadiana* | PV757245 | PV758811 | N/A | N/A |
| Heilongjiang | Site 6 | 1/1 | 17_Rosa chinensis Leaf | (5) | CSFF 31559 | T18_B36_N/A_N/A | N/A | TB genotype 12 | *Ca. canadiana* | PV757246 | PV758812 | N/A | N/A |
| Heilongjiang | Site 6 | 1/1 | 18_Acacia mangium Leaf | (1) | CSFF 31590 | T18_B36_N/A_N/A | N/A | TB genotype 12 | *Ca. canadiana* | PV757277 | PV758843 | N/A | N/A |
| Heilongjiang | Site 6 | 1/1 | 18_Acacia mangium Leaf | (1) | CSFF 31591 | T18_B36_N/A_N/A | N/A | TB genotype 12 | *Ca. canadiana* | PV757278 | PV758844 | N/A | N/A |
| Heilongjiang | Site 6 | 1/1 | 18_Acacia mangium Leaf | (2) | CSFF 31592 | T18_B36_N/A_N/A | N/A | TB genotype 12 | *Ca. canadiana* | PV757279 | PV758845 | N/A | N/A |
| Heilongjiang | Site 6 | 1/1 | 18_Acacia mangium Leaf | (2) | CSFF 31593 | T18_B36_N/A_N/A | N/A | TB genotype 12 | *Ca. canadiana* | PV757280 | PV758846 | N/A | N/A |
| Heilongjiang | Site 6 | 1/1 | 18_Acacia mangium Leaf | (3) | CSFF 31594 | T18_B36_N/A_N/A | N/A | TB genotype 12 | *Ca. canadiana* | PV757281 | PV758847 | N/A | N/A |
| Heilongjiang | Site 6 | 1/1 | 18_Acacia mangium Leaf | (3) | CSFF 31595 | T18_B36_N/A_N/A | N/A | TB genotype 12 | *Ca. canadiana* | PV757282 | PV758848 | N/A | N/A |
| Heilongjiang | Site 6 | 1/1 | 18_Acacia mangium Leaf | (4) | CSFF 31596 | T18_B36_N/A_N/A | N/A | TB genotype 12 | *Ca. canadiana* | PV757283 | PV758849 | N/A | N/A |
| Heilongjiang | Site 6 | 1/1 | 18_Acacia mangium Leaf | (4) | CSFF 31597 | T30_B32_H16_C22 | TBHC genotype 49 | TB genotype 41 | *Ca. lichi* | PV757284 | PV758850 | PV757562 | PV757704 |
| Heilongjiang | Site 6 | 1/1 | 18_Acacia mangium Leaf | (5) | CSFF 31598 | T18_B36_N/A_N/A | N/A | TB genotype 12 | *Ca. canadiana* | PV757285 | PV758851 | N/A | N/A |
| Heilongjiang | Site 6 | 1/1 | 18_Acacia mangium Leaf | (5) | CSFF 31599 | T18_B36_N/A_N/A | N/A | TB genotype 12 | *Ca. canadiana* | PV757286 | PV758852 | N/A | N/A |
| Heilongjiang | Site 6 | 1/1 | 19_Dianthus caryophyllus Leaf | (1) | CSFF 31540 | T18_B36_H14_C18 | TBHC genotype 13 | TB genotype 12 | *Ca. canadiana* | PV757227 | PV758793 | PV757557 | PV757699 |
| Heilongjiang | Site 6 | 1/1 | 19_Dianthus caryophyllus Leaf | (1) | CSFF 31541 | T18_B36_N/A_N/A | N/A | TB genotype 12 | *Ca. canadiana* | PV757228 | PV758794 | N/A | N/A |
| Heilongjiang | Site 6 | 1/1 | 19_Dianthus caryophyllus Leaf | (2) | CSFF 31542 | T18_B36_N/A_N/A | N/A | TB genotype 12 | *Ca. canadiana* | PV757229 | PV758795 | N/A | N/A |
| Heilongjiang | Site 6 | 1/1 | 19_Dianthus caryophyllus Leaf | (2) | CSFF 31543 | T18_B36_N/A_N/A | N/A | TB genotype 12 | *Ca. canadiana* | PV757230 | PV758796 | N/A | N/A |
| Heilongjiang | Site 6 | 1/1 | 19_Dianthus caryophyllus Leaf | (3) | CSFF 31544 | T18_B36_N/A_N/A | N/A | TB genotype 12 | *Ca. canadiana* | PV757231 | PV758797 | N/A | N/A |
| Heilongjiang | Site 6 | 1/1 | 19_Dianthus caryophyllus Leaf | (3) | CSFF 31545 | T18_B36_N/A_N/A | N/A | TB genotype 12 | *Ca. canadiana* | PV757232 | PV758798 | N/A | N/A |
| Heilongjiang | Site 6 | 1/1 | 19_Dianthus caryophyllus Leaf | (4) | CSFF 31546 | T18_B35_H13_C18 | TBHC genotype 11 | TB genotype 11 | *Ca. canadiana* | PV757233 | PV758799 | PV757558 | PV757700 |
| Heilongjiang | Site 6 | 1/1 | 19_Dianthus caryophyllus Leaf | (4) | CSFF 31547 | T18_B35_H13_C18 | TBHC genotype 11 | TB genotype 11 | *Ca. canadiana* | PV757234 | PV758800 | PV757559 | PV757701 |
| Heilongjiang | Site 6 | 1/1 | 19_Dianthus caryophyllus Leaf | (5) | CSFF 31548 | T18_B36_N/A_N/A | N/A | TB genotype 12 | *Ca. canadiana* | PV757235 | PV758801 | N/A | N/A |
| Heilongjiang | Site 6 | 1/1 | 19_Dianthus caryophyllus Leaf | (5) | CSFF 31549 | T18_B36_N/A_N/A | N/A | TB genotype 12 | *Ca. canadiana* | PV757236 | PV758802 | N/A | N/A |
| Heilongjiang | Site 6 | 1/1 | 20_Cinnamomum camphora Leaf | (1) | CSFF 31560 | T18_B36_N/A_N/A | N/A | TB genotype 12 | *Ca. canadiana* | PV757247 | PV758813 | N/A | N/A |
| Heilongjiang | Site 6 | 1/1 | 20_Cinnamomum camphora Leaf | (1) | CSFF 31561 | T18_B36_N/A_N/A | N/A | TB genotype 12 | *Ca. canadiana* | PV757248 | PV758814 | N/A | N/A |
| Heilongjiang | Site 6 | 1/1 | 20_Cinnamomum camphora Leaf | (2) | CSFF 31562 | T18_B35_H13_C18 | TBHC genotype 11 | TB genotype 11 | *Ca. canadiana* | PV757249 | PV758815 | PV757560 | PV757702 |
| Heilongjiang | Site 6 | 1/1 | 20_Cinnamomum camphora Leaf | (2) | CSFF 31563 | T18_B36_N/A_N/A | N/A | TB genotype 12 | *Ca. canadiana* | PV757250 | PV758816 | N/A | N/A |
| Heilongjiang | Site 6 | 1/1 | 20_Cinnamomum camphora Leaf | (3) | CSFF 31564 | T18_B36_N/A_N/A | N/A | TB genotype 12 | *Ca. canadiana* | PV757251 | PV758817 | N/A | N/A |
| Heilongjiang | Site 6 | 1/1 | 20_Cinnamomum camphora Leaf | (3) | CSFF 31565 | T18_B36_N/A_N/A | N/A | TB genotype 12 | *Ca. canadiana* | PV757252 | PV758818 | N/A | N/A |
| Heilongjiang | Site 6 | 1/1 | 20_Cinnamomum camphora Leaf | (4) | CSFF 31566 | T18_B36_N/A_N/A | N/A | TB genotype 12 | *Ca. canadiana* | PV757253 | PV758819 | N/A | N/A |
| Heilongjiang | Site 6 | 1/1 | 20_Cinnamomum camphora Leaf | (4) | CSFF 31567 | T18_B36_N/A_N/A | N/A | TB genotype 12 | *Ca. canadiana* | PV757254 | PV758820 | N/A | N/A |
| Heilongjiang | Site 6 | 1/1 | 20_Cinnamomum camphora Leaf | (5) | CSFF 31568 | T18_B36_N/A_N/A | N/A | TB genotype 12 | *Ca. canadiana* | PV757255 | PV758821 | N/A | N/A |
| Heilongjiang | Site 6 | 1/1 | 20_Cinnamomum camphora Leaf | (5) | CSFF 31569 | T18_B36_N/A_N/A | N/A | TB genotype 12 | *Ca. canadiana* | PV757256 | PV758822 | N/A | N/A |
| Heilongjiang | Site 6 | 1/1 | 21_Washingtonia filifera Leaf | (1) | CSFF 31570 | T18_B36_N/A_N/A | N/A | TB genotype 12 | *Ca. canadiana* | PV757257 | PV758823 | N/A | N/A |
| Heilongjiang | Site 6 | 1/1 | 21_Washingtonia filifera Leaf | (1) | CSFF 31571 | T18_B36_N/A_N/A | N/A | TB genotype 12 | *Ca. canadiana* | PV757258 | PV758824 | N/A | N/A |
| Heilongjiang | Site 6 | 1/1 | 21_Washingtonia filifera Leaf | (2) | CSFF 31572 | T18_B36_N/A_N/A | N/A | TB genotype 12 | *Ca. canadiana* | PV757259 | PV758825 | N/A | N/A |
| Heilongjiang | Site 6 | 1/1 | 21_Washingtonia filifera Leaf | (2) | CSFF 31573 | T18_B36_N/A_N/A | N/A | TB genotype 12 | *Ca. canadiana* | PV757260 | PV758826 | N/A | N/A |
| Heilongjiang | Site 6 | 1/1 | 21_Washingtonia filifera Leaf | (3) | CSFF 31574 | T18_B36_N/A_N/A | N/A | TB genotype 12 | *Ca. canadiana* | PV757261 | PV758827 | N/A | N/A |
| Heilongjiang | Site 6 | 1/1 | 21_Washingtonia filifera Leaf | (3) | CSFF 31575 | T18_B35_H13_C18 | TBHC genotype 11 | TB genotype 11 | *Ca. canadiana* | PV757262 | PV758828 | PV757561 | PV757703 |
| Heilongjiang | Site 6 | 1/1 | 21_Washingtonia filifera Leaf | (4) | CSFF 31576 | T18_B36_N/A_N/A | N/A | TB genotype 12 | *Ca. canadiana* | PV757263 | PV758829 | N/A | N/A |
| Heilongjiang | Site 6 | 1/1 | 21_Washingtonia filifera Leaf | (4) | CSFF 31577 | T18_B36_N/A_N/A | N/A | TB genotype 12 | *Ca. canadiana* | PV757264 | PV758830 | N/A | N/A |
| Heilongjiang | Site 6 | 1/1 | 21_Washingtonia filifera Leaf | (5) | CSFF 31578 | T18_B36_N/A_N/A | N/A | TB genotype 12 | *Ca. canadiana* | PV757265 | PV758831 | N/A | N/A |
| Heilongjiang | Site 6 | 1/1 | 21_Washingtonia filifera Leaf | (5) | CSFF 31579 | T18_B36_N/A_N/A | N/A | TB genotype 12 | *Ca. canadiana* | PV757266 | PV758832 | N/A | N/A |
| Heilongjiang | Site 6 | 1/1 | 22_Ficus benjamina Leaf | (1) | CSFF 31610 | T18_B36_N/A_N/A | N/A | TB genotype 12 | *Ca. canadiana* | PV757297 | PV758863 | N/A | N/A |
| Heilongjiang | Site 6 | 1/1 | 22_Ficus benjamina Leaf | (1) | CSFF 31611 | T18_B36_N/A_N/A | N/A | TB genotype 12 | *Ca. canadiana* | PV757298 | PV758864 | N/A | N/A |
| Heilongjiang | Site 6 | 1/1 | 22_Ficus benjamina Leaf | (2) | CSFF 31612 | T18_B36_N/A_N/A | N/A | TB genotype 12 | *Ca. canadiana* | PV757299 | PV758865 | N/A | N/A |
| Heilongjiang | Site 6 | 1/1 | 22_Ficus benjamina Leaf | (2) | CSFF 31613 | T18_B36_N/A_N/A | N/A | TB genotype 12 | *Ca. canadiana* | PV757300 | PV758866 | N/A | N/A |
| Heilongjiang | Site 6 | 1/1 | 22_Ficus benjamina Leaf | (3) | CSFF 31614 | T18_B36_N/A_N/A | N/A | TB genotype 12 | *Ca. canadiana* | PV757301 | PV758867 | N/A | N/A |
| Heilongjiang | Site 6 | 1/1 | 22_Ficus benjamina Leaf | (3) | CSFF 31615 | T18_B36_N/A_N/A | N/A | TB genotype 12 | *Ca. canadiana* | PV757302 | PV758868 | N/A | N/A |
| Heilongjiang | Site 6 | 1/1 | 22_Ficus benjamina Leaf | (4) | CSFF 31616 | T18_B36_N/A_N/A | N/A | TB genotype 12 | *Ca. canadiana* | PV757303 | PV758869 | N/A | N/A |
| Heilongjiang | Site 6 | 1/1 | 22_Ficus benjamina Leaf | (4) | CSFF 31617 | T18_B36_N/A_N/A | N/A | TB genotype 12 | *Ca. canadiana* | PV757304 | PV758870 | N/A | N/A |
| Heilongjiang | Site 6 | 1/1 | 22_Ficus benjamina Leaf | (5) | CSFF 31618 | T18_B36_N/A_N/A | N/A | TB genotype 12 | *Ca. canadiana* | PV757305 | PV758871 | N/A | N/A |
| Heilongjiang | Site 6 | 1/1 | 22_Ficus benjamina Leaf | (5) | CSFF 31619 | T18_B36_N/A_N/A | N/A | TB genotype 12 | *Ca. canadiana* | PV757306 | PV758872 | N/A | N/A |
| Heilongjiang | Site 6 | 1/1 | 23_Rhododendron pulchrum Leaf | (1) | CSFF 31626 | T18_B36_N/A_N/A | N/A | TB genotype 12 | *Ca. canadiana* | PV757313 | PV758879 | N/A | N/A |
| Heilongjiang | Site 6 | 1/1 | 23_Rhododendron pulchrum Leaf | (1) | CSFF 31627 | T18_B36_N/A_N/A | N/A | TB genotype 12 | *Ca. canadiana* | PV757314 | PV758880 | N/A | N/A |
| Heilongjiang | Site 6 | 1/1 | 23_Rhododendron pulchrum Leaf | (2) | CSFF 31628 | T18_B36_N/A_N/A | N/A | TB genotype 12 | *Ca. canadiana* | PV757315 | PV758881 | N/A | N/A |
| Heilongjiang | Site 6 | 1/1 | 23_Rhododendron pulchrum Leaf | (2) | CSFF 31629 | T18_B36_N/A_N/A | N/A | TB genotype 12 | *Ca. canadiana* | PV757316 | PV758882 | N/A | N/A |
| Heilongjiang | Site 6 | 1/1 | 23_Rhododendron pulchrum Leaf | (3) | CSFF 31630 | T18_B36_N/A_N/A | N/A | TB genotype 12 | *Ca. canadiana* | PV757317 | PV758883 | N/A | N/A |
| Heilongjiang | Site 6 | 1/1 | 23_Rhododendron pulchrum Leaf | (3) | CSFF 31631 | T18_B36_N/A_N/A | N/A | TB genotype 12 | *Ca. canadiana* | PV757318 | PV758884 | N/A | N/A |
| Heilongjiang | Site 6 | 1/1 | 23_Rhododendron pulchrum Leaf | (4) | CSFF 31632 | T18_B36_N/A_N/A | N/A | TB genotype 12 | *Ca. canadiana* | PV757319 | PV758885 | N/A | N/A |
| Heilongjiang | Site 6 | 1/1 | 23_Rhododendron pulchrum Leaf | (4) | CSFF 31633 | T18_B36_N/A_N/A | N/A | TB genotype 12 | *Ca. canadiana* | PV757320 | PV758886 | N/A | N/A |
| Heilongjiang | Site 6 | 1/1 | 23_Rhododendron pulchrum Leaf | (5) | CSFF 31634 | T18_B36_N/A_N/A | N/A | TB genotype 12 | *Ca. canadiana* | PV757321 | PV758887 | N/A | N/A |
| Heilongjiang | Site 6 | 1/1 | 23_Rhododendron pulchrum Leaf | (5) | CSFF 31635 | T18_B36_N/A_N/A | N/A | TB genotype 12 | *Ca. canadiana* | PV757322 | PV758888 | N/A | N/A |
| Heilongjiang | Site 6 | 1/1 | 24_Glycine max Stem | (2) | CSFF 31640 | T18_B36_N/A_N/A | N/A | TB genotype 12 | *Ca. canadiana* | PV757327 | PV758893 | N/A | N/A |
| Heilongjiang | Site 6 | 1/1 | 24_Glycine max Stem | (2) | CSFF 31641 | T18_B36_N/A_N/A | N/A | TB genotype 12 | *Ca. canadiana* | PV757328 | PV758894 | N/A | N/A |
| Heilongjiang | Site 6 | 1/1 | 24_Glycine max Stem | (3) | CSFF 31642 | T18_B36_N/A_N/A | N/A | TB genotype 12 | *Ca. canadiana* | PV757329 | PV758895 | N/A | N/A |
| Heilongjiang | Site 6 | 1/1 | 24_Glycine max Stem | (3) | CSFF 31643 | T18_B36_N/A_N/A | N/A | TB genotype 12 | *Ca. canadiana* | PV757330 | PV758896 | N/A | N/A |
| Heilongjiang | Site 6 | 1/1 | 24_Glycine max Stem | (5) | CSFF 31644 | T18_B36_N/A_N/A | N/A | TB genotype 12 | *Ca. canadiana* | PV757331 | PV758897 | N/A | N/A |
| Heilongjiang | Site 6 | 1/1 | 24_Glycine max Stem | (5) | CSFF 31645 | T18_B36_N/A_N/A | N/A | TB genotype 12 | *Ca. canadiana* | PV757332 | PV758898 | N/A | N/A |
| Heilongjiang | Site 6 | 1/1 | 26_Medicago sativa Seed | (1) | CSFF 31666 | T18_B36_H14_C18 | TBHC genotype 13 | TB genotype 12 | *Ca. canadiana* | PV757353 | PV758919 | PV757564 | PV757706 |
| Heilongjiang | Site 6 | 1/1 | 26_Medicago sativa Seed | (1) | CSFF 31667 | T18_B36_N/A_N/A | N/A | TB genotype 12 | *Ca. canadiana* | PV757354 | PV758920 | N/A | N/A |
| Heilongjiang | Site 6 | 1/1 | 26_Medicago sativa Seed | (2) | CSFF 31668 | T18_B36_N/A_N/A | N/A | TB genotype 12 | *Ca. canadiana* | PV757355 | PV758921 | N/A | N/A |
| Heilongjiang | Site 6 | 1/1 | 26_Medicago sativa Seed | (2) | CSFF 31669 | T18_B36_N/A_N/A | N/A | TB genotype 12 | *Ca. canadiana* | PV757356 | PV758922 | N/A | N/A |
| Heilongjiang | Site 6 | 1/1 | 26_Medicago sativa Seed | (3) | CSFF 31670 | T18_B36_N/A_N/A | N/A | TB genotype 12 | *Ca. canadiana* | PV757357 | PV758923 | N/A | N/A |
| Heilongjiang | Site 6 | 1/1 | 26_Medicago sativa Seed | (3) | CSFF 31671 | T18_B36_N/A_N/A | N/A | TB genotype 12 | *Ca. canadiana* | PV757358 | PV758924 | N/A | N/A |
| Heilongjiang | Site 6 | 1/1 | 26_Medicago sativa Seed | (4) | CSFF 31672 | T18_B36_N/A_N/A | N/A | TB genotype 12 | *Ca. canadiana* | PV757359 | PV758925 | N/A | N/A |
| Heilongjiang | Site 6 | 1/1 | 26_Medicago sativa Seed | (4) | CSFF 31673 | T18_B36_N/A_N/A | N/A | TB genotype 12 | *Ca. canadiana* | PV757360 | PV758926 | N/A | N/A |
| Heilongjiang | Site 6 | 1/1 | 26_Medicago sativa Seed | (5) | CSFF 31674 | T18_B36_N/A_N/A | N/A | TB genotype 12 | *Ca. canadiana* | PV757361 | PV758927 | N/A | N/A |
| Heilongjiang | Site 6 | 1/1 | 26_Medicago sativa Seed | (5) | CSFF 31675 | T18_B36_N/A_N/A | N/A | TB genotype 12 | *Ca. canadiana* | PV757362 | PV758928 | N/A | N/A |
| Henan | Site 7 | 1/1 | 7_Dianthus caryophyllus Seed | (2) | CSFF 31676 | T18_B38_H13_C18 | TBHC genotype 16 | TB genotype 14 | *Ca. canadiana* | PV757363 | PV758929 | PV757565 | PV757707 |
| Henan | Site 7 | 1/1 | 7_Dianthus caryophyllus Seed | (2) | CSFF 31677 | T18_B38_N/A_N/A | N/A | TB genotype 14 | *Ca. canadiana* | PV757364 | PV758930 | N/A | N/A |
| Henan | Site 7 | 1/1 | 7_Dianthus caryophyllus Seed | (3) | CSFF 31678 | T18_B38_N/A_N/A | N/A | TB genotype 14 | *Ca. canadiana* | PV757365 | PV758931 | N/A | N/A |
| Henan | Site 7 | 1/1 | 7_Dianthus caryophyllus Seed | (3) | CSFF 31679 | T18_B38_N/A_N/A | N/A | TB genotype 14 | *Ca. canadiana* | PV757366 | PV758932 | N/A | N/A |
| Henan | Site 7 | 1/1 | 12_Pinus massoniana Leaf | (2) | CSFF 31730 | T18_B38_N/A_N/A | N/A | TB genotype 14 | *Ca. canadiana* | PV757417 | PV758983 | N/A | N/A |
| Henan | Site 7 | 1/1 | 12_Pinus massoniana Leaf | (2) | CSFF 31731 | T18_B38_N/A_N/A | N/A | TB genotype 14 | *Ca. canadiana* | PV757418 | PV758984 | N/A | N/A |
| Henan | Site 7 | 1/1 | 12_Pinus massoniana Leaf | (5) | CSFF 31732 | T28_B29_N/A_N/A | N/A | TB genotype 39 | *Ca. lichi* | PV757419 | PV758985 | N/A | N/A |
| Henan | Site 7 | 1/1 | 12_Pinus massoniana Leaf | (5) | CSFF 31733 | T28_B29_N/A_N/A | N/A | TB genotype 39 | *Ca. lichi* | PV757420 | PV758986 | N/A | N/A |
| Henan | Site 7 | 1/1 | 14_Syzygium jambos Leaf | (1) | CSFF 31714 | T18_B37_H14_C18 | TBHC genotype 15 | TB genotype 13 | *Ca. canadiana* | PV757401 | PV758967 | PV757574 | PV757716 |
| Henan | Site 7 | 1/1 | 14_Syzygium jambos Leaf | (1) | CSFF 31715 | T18_B37_H14_C18 | TBHC genotype 15 | TB genotype 13 | *Ca. canadiana* | PV757402 | PV758968 | PV757575 | PV757717 |
| Henan | Site 7 | 1/1 | 14_Syzygium jambos Leaf | (3) | CSFF 31716 | T18_B38_H13_C18 | TBHC genotype 16 | TB genotype 14 | *Ca. canadiana* | PV757403 | PV758969 | PV757576 | PV757718 |
| Henan | Site 7 | 1/1 | 14_Syzygium jambos Leaf | (3) | CSFF 31717 | T18_B38_N/A_N/A | N/A | TB genotype 14 | *Ca. canadiana* | PV757404 | PV758970 | N/A | N/A |
| Henan | Site 7 | 1/1 | 14_Syzygium jambos Leaf | (4) | CSFF 31718 | T28_B29_H15_C22 | TBHC genotype 47 | TB genotype 39 | *Ca. lichi* | PV757405 | PV758971 | PV757577 | PV757719 |
| Henan | Site 7 | 1/1 | 14_Syzygium jambos Leaf | (4) | CSFF 31719 | T28_B29_N/A_N/A | N/A | TB genotype 39 | *Ca. lichi* | PV757406 | PV758972 | N/A | N/A |
| Henan | Site 7 | 1/1 | 15_Pennisetum purpureum Leaf | (2) | CSFF 31744 | T18_B38_N/A_N/A | N/A | TB genotype 14 | *Ca. canadiana* | PV757431 | PV758997 | N/A | N/A |
| Henan | Site 7 | 1/1 | 15_Pennisetum purpureum Leaf | (2) | CSFF 31745 | T18_B38_N/A_N/A | N/A | TB genotype 14 | *Ca. canadiana* | PV757432 | PV758998 | N/A | N/A |
| Henan | Site 7 | 1/1 | 15_Pennisetum purpureum Leaf | (3) | CSFF 31746 | T18_B38_N/A_N/A | N/A | TB genotype 14 | *Ca. canadiana* | PV757433 | PV758999 | N/A | N/A |
| Henan | Site 7 | 1/1 | 15_Pennisetum purpureum Leaf | (3) | CSFF 31747 | T18_B38_N/A_N/A | N/A | TB genotype 14 | *Ca. canadiana* | PV757434 | PV759000 | N/A | N/A |
| Henan | Site 7 | 1/1 | 16_Saccharum officinarum Leaf | (1) | CSFF 31706 | T28_B29_N/A_N/A | N/A | TB genotype 39 | *Ca. lichi* | PV757393 | PV758959 | N/A | N/A |
| Henan | Site 7 | 1/1 | 16_Saccharum officinarum Leaf | (1) | CSFF 31707 | T28_B29_N/A_N/A | N/A | TB genotype 39 | *Ca. lichi* | PV757394 | PV758960 | N/A | N/A |
| Henan | Site 7 | 1/1 | 16_Saccharum officinarum Leaf | (5) | CSFF 31708 | T18_B37_H13_C18 | TBHC genotype 14 | TB genotype 13 | *Ca. canadiana* | PV757395 | PV758961 | PV757572 | PV757714 |
| Henan | Site 7 | 1/1 | 16_Saccharum officinarum Leaf | (5) | CSFF 31709 | T18_B37_H13_C18 | TBHC genotype 14 | TB genotype 13 | *Ca. canadiana* | PV757396 | PV758962 | PV757573 | PV757715 |
| Henan | Site 7 | 1/1 | 17_Rosa chinensis Leaf | (2) | CSFF 31684 | T18_B38_N/A_N/A | N/A | TB genotype 14 | *Ca. canadiana* | PV757371 | PV758937 | N/A | N/A |
| Henan | Site 7 | 1/1 | 17_Rosa chinensis Leaf | (2) | CSFF 31685 | T18_B38_N/A_N/A | N/A | TB genotype 14 | *Ca. canadiana* | PV757372 | PV758938 | N/A | N/A |
| Henan | Site 7 | 1/1 | 17_Rosa chinensis Leaf | (4) | CSFF 31686 | T28_B29_N/A_N/A | N/A | TB genotype 39 | *Ca. lichi* | PV757373 | PV758939 | N/A | N/A |
| Henan | Site 7 | 1/1 | 17_Rosa chinensis Leaf | (4) | CSFF 31687 | T28_B29_N/A_N/A | N/A | TB genotype 39 | *Ca. lichi* | PV757374 | PV758940 | N/A | N/A |
| Henan | Site 7 | 1/1 | 18_Acacia mangium Leaf | (2) | CSFF 31710 | T28_B29_N/A_N/A | N/A | TB genotype 39 | *Ca. lichi* | PV757397 | PV758963 | N/A | N/A |
| Henan | Site 7 | 1/1 | 18_Acacia mangium Leaf | (2) | CSFF 31711 | T28_B29_N/A_N/A | N/A | TB genotype 39 | *Ca. lichi* | PV757398 | PV758964 | N/A | N/A |
| Henan | Site 7 | 1/1 | 18_Acacia mangium Leaf | (4) | CSFF 31712 | T28_B29_N/A_N/A | N/A | TB genotype 39 | *Ca. lichi* | PV757399 | PV758965 | N/A | N/A |
| Henan | Site 7 | 1/1 | 18_Acacia mangium Leaf | (4) | CSFF 31713 | T28_B29_N/A_N/A | N/A | TB genotype 39 | *Ca. lichi* | PV757400 | PV758966 | N/A | N/A |
| Henan | Site 7 | 1/1 | 19_Dianthus caryophyllus Leaf | (2) | CSFF 31680 | T29_B29_H15_C22 | TBHC genotype 48 | TB genotype 40 | *Ca. lichi* | PV757367 | PV758933 | PV757566 | PV757708 |
| Henan | Site 7 | 1/1 | 19_Dianthus caryophyllus Leaf | (2) | CSFF 31681 | T29_B29_H15_C22 | TBHC genotype 48 | TB genotype 40 | *Ca. lichi* | PV757368 | PV758934 | PV757567 | PV757709 |
| Henan | Site 7 | 1/1 | 19_Dianthus caryophyllus Leaf | (5) | CSFF 31682 | T29_B29_H15_C22 | TBHC genotype 48 | TB genotype 40 | *Ca. lichi* | PV757369 | PV758935 | PV757568 | PV757710 |
| Henan | Site 7 | 1/1 | 19_Dianthus caryophyllus Leaf | (5) | CSFF 31683 | T29_B29_H15_C22 | TBHC genotype 48 | TB genotype 40 | *Ca. lichi* | PV757370 | PV758936 | PV757569 | PV757711 |
| Henan | Site 7 | 1/1 | 20_Cinnamomum camphora Leaf | (1) | CSFF 31688 | T18_B38_N/A_N/A | N/A | TB genotype 14 | *Ca. canadiana* | PV757375 | PV758941 | N/A | N/A |
| Henan | Site 7 | 1/1 | 20_Cinnamomum camphora Leaf | (1) | CSFF 31689 | T18_B38_N/A_N/A | N/A | TB genotype 14 | *Ca. canadiana* | PV757376 | PV758942 | N/A | N/A |
| Henan | Site 7 | 1/1 | 20_Cinnamomum camphora Leaf | (3) | CSFF 31690 | T18_B38_N/A_N/A | N/A | TB genotype 14 | *Ca. canadiana* | PV757377 | PV758943 | N/A | N/A |
| Henan | Site 7 | 1/1 | 20_Cinnamomum camphora Leaf | (3) | CSFF 31691 | T18_B38_N/A_N/A | N/A | TB genotype 14 | *Ca. canadiana* | PV757378 | PV758944 | N/A | N/A |
| Henan | Site 7 | 1/1 | 20_Cinnamomum camphora Leaf | (4) | CSFF 31692 | T18_B36_H13_C19 | TBHC genotype 12 | TB genotype 12 | *Ca. canadiana* | PV757379 | PV758945 | PV757570 | PV757712 |
| Henan | Site 7 | 1/1 | 20_Cinnamomum camphora Leaf | (4) | CSFF 31693 | T18_B36_H13_C19 | TBHC genotype 12 | TB genotype 12 | *Ca. canadiana* | PV757380 | PV758946 | PV757571 | PV757713 |
| Henan | Site 7 | 1/1 | 20_Cinnamomum camphora Leaf | (5) | CSFF 31694 | T18_B38_N/A_N/A | N/A | TB genotype 14 | *Ca. canadiana* | PV757381 | PV758947 | N/A | N/A |
| Henan | Site 7 | 1/1 | 20_Cinnamomum camphora Leaf | (5) | CSFF 31695 | T18_B38_N/A_N/A | N/A | TB genotype 14 | *Ca. canadiana* | PV757382 | PV758948 | N/A | N/A |
| Henan | Site 7 | 1/1 | 21_Washingtonia filifera Leaf | (1) | CSFF 31696 | T28_B29_N/A_N/A | N/A | TB genotype 39 | *Ca. lichi* | PV757383 | PV758949 | N/A | N/A |
| Henan | Site 7 | 1/1 | 21_Washingtonia filifera Leaf | (1) | CSFF 31697 | T18_B38_N/A_N/A | N/A | TB genotype 14 | *Ca. canadiana* | PV757384 | PV758950 | N/A | N/A |
| Henan | Site 7 | 1/1 | 21_Washingtonia filifera Leaf | (2) | CSFF 31698 | T18_B38_N/A_N/A | N/A | TB genotype 14 | *Ca. canadiana* | PV757385 | PV758951 | N/A | N/A |
| Henan | Site 7 | 1/1 | 21_Washingtonia filifera Leaf | (2) | CSFF 31699 | T18_B38_N/A_N/A | N/A | TB genotype 14 | *Ca. canadiana* | PV757386 | PV758952 | N/A | N/A |
| Henan | Site 7 | 1/1 | 21_Washingtonia filifera Leaf | (3) | CSFF 31700 | T18_B38_N/A_N/A | N/A | TB genotype 14 | *Ca. canadiana* | PV757387 | PV758953 | N/A | N/A |
| Henan | Site 7 | 1/1 | 21_Washingtonia filifera Leaf | (3) | CSFF 31701 | T18_B38_N/A_N/A | N/A | TB genotype 14 | *Ca. canadiana* | PV757388 | PV758954 | N/A | N/A |
| Henan | Site 7 | 1/1 | 21_Washingtonia filifera Leaf | (4) | CSFF 31702 | T18_B38_N/A_N/A | N/A | TB genotype 14 | *Ca. canadiana* | PV757389 | PV758955 | N/A | N/A |
| Henan | Site 7 | 1/1 | 21_Washingtonia filifera Leaf | (4) | CSFF 31703 | T18_B38_N/A_N/A | N/A | TB genotype 14 | *Ca. canadiana* | PV757390 | PV758956 | N/A | N/A |
| Henan | Site 7 | 1/1 | 21_Washingtonia filifera Leaf | (5) | CSFF 31704 | T18_B38_N/A_N/A | N/A | TB genotype 14 | *Ca. canadiana* | PV757391 | PV758957 | N/A | N/A |
| Henan | Site 7 | 1/1 | 21_Washingtonia filifera Leaf | (5) | CSFF 31705 | T18_B38_N/A_N/A | N/A | TB genotype 14 | *Ca. canadiana* | PV757392 | PV758958 | N/A | N/A |
| Henan | Site 7 | 1/1 | 22_Ficus benjamina Leaf | (1) | CSFF 31720 | T18_B38_N/A_N/A | N/A | TB genotype 14 | *Ca. canadiana* | PV757407 | PV758973 | N/A | N/A |
| Henan | Site 7 | 1/1 | 22_Ficus benjamina Leaf | (1) | CSFF 31721 | T28_B29_H15_C22 | TBHC genotype 47 | TB genotype 39 | *Ca. lichi* | PV757408 | PV758974 | PV757578 | PV757720 |
| Henan | Site 7 | 1/1 | 22_Ficus benjamina Leaf | (2) | CSFF 31722 | T28_B29_N/A_N/A | N/A | TB genotype 39 | *Ca. lichi* | PV757409 | PV758975 | N/A | N/A |
| Henan | Site 7 | 1/1 | 22_Ficus benjamina Leaf | (2) | CSFF 31723 | T18_B38_N/A_N/A | N/A | TB genotype 14 | *Ca. canadiana* | PV757410 | PV758976 | N/A | N/A |
| Henan | Site 7 | 1/1 | 22_Ficus benjamina Leaf | (3) | CSFF 31724 | T18_B38_N/A_N/A | N/A | TB genotype 14 | *Ca. canadiana* | PV757411 | PV758977 | N/A | N/A |
| Henan | Site 7 | 1/1 | 22_Ficus benjamina Leaf | (3) | CSFF 31725 | T18_B38_N/A_N/A | N/A | TB genotype 14 | *Ca. canadiana* | PV757412 | PV758978 | N/A | N/A |
| Henan | Site 7 | 1/1 | 22_Ficus benjamina Leaf | (4) | CSFF 31726 | T28_B29_N/A_N/A | N/A | TB genotype 39 | *Ca. lichi* | PV757413 | PV758979 | N/A | N/A |
| Henan | Site 7 | 1/1 | 22_Ficus benjamina Leaf | (4) | CSFF 31727 | T28_B29_N/A_N/A | N/A | TB genotype 39 | *Ca. lichi* | PV757414 | PV758980 | N/A | N/A |
| Henan | Site 7 | 1/1 | 22_Ficus benjamina Leaf | (5) | CSFF 31728 | T28_B29_N/A_N/A | N/A | TB genotype 39 | *Ca. lichi* | PV757415 | PV758981 | N/A | N/A |
| Henan | Site 7 | 1/1 | 22_Ficus benjamina Leaf | (5) | CSFF 31729 | T28_B29_N/A_N/A | N/A | TB genotype 39 | *Ca. lichi* | PV757416 | PV758982 | N/A | N/A |
| Henan | Site 7 | 1/1 | 23_Rhododendron pulchrum Leaf | (1) | CSFF 31734 | T28_B29_H15_C22 | TBHC genotype 47 | TB genotype 39 | *Ca. lichi* | PV757421 | PV758987 | PV757579 | PV757721 |
| Henan | Site 7 | 1/1 | 23_Rhododendron pulchrum Leaf | (1) | CSFF 31735 | T18_B38_H13_C18 | TBHC genotype 16 | TB genotype 14 | *Ca. canadiana* | PV757422 | PV758988 | PV757580 | PV757722 |
| Henan | Site 7 | 1/1 | 23_Rhododendron pulchrum Leaf | (2) | CSFF 31736 | T28_B29_N/A_N/A | N/A | TB genotype 39 | *Ca. lichi* | PV757423 | PV758989 | N/A | N/A |
| Henan | Site 7 | 1/1 | 23_Rhododendron pulchrum Leaf | (2) | CSFF 31737 | T28_B29_N/A_N/A | N/A | TB genotype 39 | *Ca. lichi* | PV757424 | PV758990 | N/A | N/A |
| Henan | Site 7 | 1/1 | 23_Rhododendron pulchrum Leaf | (3) | CSFF 31738 | T28_B29_N/A_N/A | N/A | TB genotype 39 | *Ca. lichi* | PV757425 | PV758991 | N/A | N/A |
| Henan | Site 7 | 1/1 | 23_Rhododendron pulchrum Leaf | (3) | CSFF 31739 | T28_B29_N/A_N/A | N/A | TB genotype 39 | *Ca. lichi* | PV757426 | PV758992 | N/A | N/A |
| Henan | Site 7 | 1/1 | 23_Rhododendron pulchrum Leaf | (4) | CSFF 31740 | T18_B38_N/A_N/A | N/A | TB genotype 14 | *Ca. canadiana* | PV757427 | PV758993 | N/A | N/A |
| Henan | Site 7 | 1/1 | 23_Rhododendron pulchrum Leaf | (4) | CSFF 31741 | T18_B38_N/A_N/A | N/A | TB genotype 14 | *Ca. canadiana* | PV757428 | PV758994 | N/A | N/A |
| Henan | Site 7 | 1/1 | 23_Rhododendron pulchrum Leaf | (5) | CSFF 31742 | T28_B29_N/A_N/A | N/A | TB genotype 39 | *Ca. lichi* | PV757429 | PV758995 | N/A | N/A |
| Henan | Site 7 | 1/1 | 23_Rhododendron pulchrum Leaf | (5) | CSFF 31743 | T28_B29_N/A_N/A | N/A | TB genotype 39 | *Ca. lichi* | PV757430 | PV758996 | N/A | N/A |
| Henan | Site 7 | 1/1 | 26_Medicago sativa Seed | (2) | CSFF 31748 | T18_B38_H13_C18 | TBHC genotype 16 | TB genotype 14 | *Ca. canadiana* | PV757435 | PV759001 | PV757581 | PV757723 |
| Henan | Site 7 | 1/1 | 26_Medicago sativa Seed | (2) | CSFF 31749 | T18_B38_N/A_N/A | N/A | TB genotype 14 | *Ca. canadiana* | PV757436 | PV759002 | N/A | N/A |
| Henan | Site 7 | 1/1 | 26_Medicago sativa Seed | (3) | CSFF 31750 | T28_B29_H15_C22 | TBHC genotype 47 | TB genotype 39 | *Ca. lichi* | PV757437 | PV759003 | PV757582 | PV757724 |
| Henan | Site 7 | 1/1 | 26_Medicago sativa Seed | (3) | CSFF 31751 | T28_B29_N/A_N/A | N/A | TB genotype 39 | *Ca. lichi* | PV757438 | PV759004 | N/A | N/A |
| Henan | Site 7 | 1/1 | 26_Medicago sativa Seed | (4) | CSFF 31752 | T28_B29_N/A_N/A | N/A | TB genotype 39 | *Ca. lichi* | PV757439 | PV759005 | N/A | N/A |
| Henan | Site 7 | 1/1 | 26_Medicago sativa Seed | (4) | CSFF 31753 | T28_B29_N/A_N/A | N/A | TB genotype 39 | *Ca. lichi* | PV757440 | PV759006 | N/A | N/A |
| Xizang | Site 8 | 1/1 | 8_Trifolium pratense Seed | (3) | CSFF 28366 | T12_B25_H11_C20 | TBHC genotype 4 | TB genotype 4 | *Ca. aconidialis* | PV757109 | PV758675 | PV757549 | PV757691 |
| Xizang | Site 8 | 1/1 | 8_Trifolium pratense Seed | (3) | CSFF 28367 | T12_B25_N/A_N/A | N/A | TB genotype 4 | *Ca. aconidialis* | PV757110 | PV758676 | N/A | N/A |
| Xizang | Site 8 | 1/1 | 9_Lythrum salicaria Seed | (2) | CSFF 28358 | T12_B27_H11_C20 | TBHC genotype 5 | TB genotype 5 | *Ca. aconidialis* | PV757101 | PV758667 | PV757548 | PV757690 |
| Xizang | Site 8 | 1/1 | 9_Lythrum salicaria Seed | (2) | CSFF 28359 | T12_B27_N/A_N/A | N/A | TB genotype 5 | *Ca. aconidialis* | PV757102 | PV758668 | N/A | N/A |
| Xizang | Site 8 | 1/1 | 9_Lythrum salicaria Seed | (3) | CSFF 28360 | T12_B27_N/A_N/A | N/A | TB genotype 5 | *Ca. aconidialis* | PV757103 | PV758669 | N/A | N/A |
| Xizang | Site 8 | 1/1 | 9_Lythrum salicaria Seed | (3) | CSFF 28361 | T12_B27_N/A_N/A | N/A | TB genotype 5 | *Ca. aconidialis* | PV757104 | PV758670 | N/A | N/A |
| Xizang | Site 8 | 1/1 | 9_Lythrum salicaria Seed | (4) | CSFF 28362 | T12_B27_N/A_N/A | N/A | TB genotype 5 | *Ca. aconidialis* | PV757105 | PV758671 | N/A | N/A |
| Xizang | Site 8 | 1/1 | 9_Lythrum salicaria Seed | (4) | CSFF 28363 | T12_B27_N/A_N/A | N/A | TB genotype 5 | *Ca. aconidialis* | PV757106 | PV758672 | N/A | N/A |
| Xizang | Site 8 | 1/1 | 9_Lythrum salicaria Seed | (5) | CSFF 28364 | T12_B27_N/A_N/A | N/A | TB genotype 5 | *Ca. aconidialis* | PV757107 | PV758673 | N/A | N/A |
| Xizang | Site 8 | 1/1 | 9_Lythrum salicaria Seed | (5) | CSFF 28365 | T12_B27_N/A_N/A | N/A | TB genotype 5 | *Ca. aconidialis* | PV757108 | PV758674 | N/A | N/A |
| Xizang | Site 8 | 1/1 | 10_Buxus sinica Leaf | (5) | CSFF 28414 | T12_B27_N/A_N/A | N/A | TB genotype 5 | *Ca. aconidialis* | PV757157 | PV758723 | N/A | N/A |
| Xizang | Site 8 | 1/1 | 10_Buxus sinica Leaf | (5) | CSFF 28415 | T12_B27_N/A_N/A | N/A | TB genotype 5 | *Ca. aconidialis* | PV757158 | PV758724 | N/A | N/A |
| Xizang | Site 8 | 1/1 | 11_Imperata cylindrica Leaf | (2) | CSFF 28398 | T12_B27_N/A_N/A | N/A | TB genotype 5 | *Ca. aconidialis* | PV757141 | PV758707 | N/A | N/A |
| Xizang | Site 8 | 1/1 | 11_Imperata cylindrica Leaf | (2) | CSFF 28399 | T12_B27_N/A_N/A | N/A | TB genotype 5 | *Ca. aconidialis* | PV757142 | PV758708 | N/A | N/A |
| Xizang | Site 8 | 1/1 | 11_Imperata cylindrica Leaf | (3) | CSFF 28400 | T12_B27_N/A_N/A | N/A | TB genotype 5 | *Ca. aconidialis* | PV757143 | PV758709 | N/A | N/A |
| Xizang | Site 8 | 1/1 | 11_Imperata cylindrica Leaf | (3) | CSFF 28401 | T12_B27_N/A_N/A | N/A | TB genotype 5 | *Ca. aconidialis* | PV757144 | PV758710 | N/A | N/A |
| Xizang | Site 8 | 1/1 | 12_Pinus massoniana Leaf | (1) | CSFF 28402 | T12_B27_N/A_N/A | N/A | TB genotype 5 | *Ca. aconidialis* | PV757145 | PV758711 | N/A | N/A |
| Xizang | Site 8 | 1/1 | 12_Pinus massoniana Leaf | (1) | CSFF 28403 | T12_B27_N/A_N/A | N/A | TB genotype 5 | *Ca. aconidialis* | PV757146 | PV758712 | N/A | N/A |
| Xizang | Site 8 | 1/1 | 12_Pinus massoniana Leaf | (2) | CSFF 28404 | T12_B27_N/A_N/A | N/A | TB genotype 5 | *Ca. aconidialis* | PV757147 | PV758713 | N/A | N/A |
| Xizang | Site 8 | 1/1 | 12_Pinus massoniana Leaf | (2) | CSFF 28405 | T12_B27_N/A_N/A | N/A | TB genotype 5 | *Ca. aconidialis* | PV757148 | PV758714 | N/A | N/A |
| Xizang | Site 8 | 1/1 | 13_Eucalyptus urophylla hybrid Leaf | (5) | CSFF 28416 | T12_B25_H11_C20 | TBHC genotype 4 | TB genotype 4 | *Ca. aconidialis* | PV757159 | PV758725 | PV757553 | PV757695 |
| Xizang | Site 8 | 1/1 | 13_Eucalyptus urophylla hybrid Leaf | (5) | CSFF 28417 | T12_B25_N/A_N/A | N/A | TB genotype 4 | *Ca. aconidialis* | PV757160 | PV758726 | N/A | N/A |
| Xizang | Site 8 | 1/1 | 14_Syzygium jambos Leaf | (1) | CSFF 28392 | T12_B27_H11_C20 | TBHC genotype 5 | TB genotype 5 | *Ca. aconidialis* | PV757135 | PV758701 | PV757551 | PV757693 |
| Xizang | Site 8 | 1/1 | 14_Syzygium jambos Leaf | (1) | CSFF 28393 | T12_B27_N/A_N/A | N/A | TB genotype 5 | *Ca. aconidialis* | PV757136 | PV758702 | N/A | N/A |
| Xizang | Site 8 | 1/1 | 15_Pennisetum purpureum Leaf | (4) | CSFF 28418 | T12_B27_N/A_N/A | N/A | TB genotype 5 | *Ca. aconidialis* | PV757161 | PV758727 | N/A | N/A |
| Xizang | Site 8 | 1/1 | 15_Pennisetum purpureum Leaf | (4) | CSFF 28419 | T12_B27_N/A_N/A | N/A | TB genotype 5 | *Ca. aconidialis* | PV757162 | PV758728 | N/A | N/A |
| Xizang | Site 8 | 1/1 | 16_Saccharum officinarum Leaf | (4) | CSFF 28388 | T12_B27_N/A_N/A | N/A | TB genotype 5 | *Ca. aconidialis* | PV757131 | PV758697 | N/A | N/A |
| Xizang | Site 8 | 1/1 | 16_Saccharum officinarum Leaf | (4) | CSFF 28389 | T12_B27_N/A_N/A | N/A | TB genotype 5 | *Ca. aconidialis* | PV757132 | PV758698 | N/A | N/A |
| Xizang | Site 8 | 1/1 | 17_Rosa chinensis Leaf | (2) | CSFF 28374 | T12_B27_N/A_N/A | N/A | TB genotype 5 | *Ca. aconidialis* | PV757117 | PV758683 | N/A | N/A |
| Xizang | Site 8 | 1/1 | 17_Rosa chinensis Leaf | (2) | CSFF 28375 | T12_B27_N/A_N/A | N/A | TB genotype 5 | *Ca. aconidialis* | PV757118 | PV758684 | N/A | N/A |
| Xizang | Site 8 | 1/1 | 17_Rosa chinensis Leaf | (3) | CSFF 28376 | T12_B27_N/A_N/A | N/A | TB genotype 5 | *Ca. aconidialis* | PV757119 | PV758685 | N/A | N/A |
| Xizang | Site 8 | 1/1 | 17_Rosa chinensis Leaf | (3) | CSFF 28377 | T12_B27_N/A_N/A | N/A | TB genotype 5 | *Ca. aconidialis* | PV757120 | PV758686 | N/A | N/A |
| Xizang | Site 8 | 1/1 | 17_Rosa chinensis Leaf | (5) | CSFF 28378 | T12_B27_N/A_N/A | N/A | TB genotype 5 | *Ca. aconidialis* | PV757121 | PV758687 | N/A | N/A |
| Xizang | Site 8 | 1/1 | 17_Rosa chinensis Leaf | (5) | CSFF 28379 | T12_B27_N/A_N/A | N/A | TB genotype 5 | *Ca. aconidialis* | PV757122 | PV758688 | N/A | N/A |
| Xizang | Site 8 | 1/1 | 18_Acacia mangium Leaf | (2) | CSFF 28390 | T12_B27_N/A_N/A | N/A | TB genotype 5 | *Ca. aconidialis* | PV757133 | PV758699 | N/A | N/A |
| Xizang | Site 8 | 1/1 | 18_Acacia mangium Leaf | (2) | CSFF 28391 | T12_B27_N/A_N/A | N/A | TB genotype 5 | *Ca. aconidialis* | PV757134 | PV758700 | N/A | N/A |
| Xizang | Site 8 | 1/1 | 19_Dianthus caryophyllus Leaf | (1) | CSFF 28368 | T12_B27_H11_C20 | TBHC genotype 5 | TB genotype 5 | *Ca. aconidialis* | PV757111 | PV758677 | PV757550 | PV757692 |
| Xizang | Site 8 | 1/1 | 19_Dianthus caryophyllus Leaf | (1) | CSFF 28369 | T12_B27_N/A_N/A | N/A | TB genotype 5 | *Ca. aconidialis* | PV757112 | PV758678 | N/A | N/A |
| Xizang | Site 8 | 1/1 | 19_Dianthus caryophyllus Leaf | (2) | CSFF 28370 | T12_B27_N/A_N/A | N/A | TB genotype 5 | *Ca. aconidialis* | PV757113 | PV758679 | N/A | N/A |
| Xizang | Site 8 | 1/1 | 19_Dianthus caryophyllus Leaf | (2) | CSFF 28371 | T12_B27_N/A_N/A | N/A | TB genotype 5 | *Ca. aconidialis* | PV757114 | PV758680 | N/A | N/A |
| Xizang | Site 8 | 1/1 | 19_Dianthus caryophyllus Leaf | (5) | CSFF 28372 | T12_B27_N/A_N/A | N/A | TB genotype 5 | *Ca. aconidialis* | PV757115 | PV758681 | N/A | N/A |
| Xizang | Site 8 | 1/1 | 19_Dianthus caryophyllus Leaf | (5) | CSFF 28373 | T12_B27_N/A_N/A | N/A | TB genotype 5 | *Ca. aconidialis* | PV757116 | PV758682 | N/A | N/A |
| Xizang | Site 8 | 1/1 | 20_Cinnamomum camphora Leaf | (3) | CSFF 28380 | T12_B27_N/A_N/A | N/A | TB genotype 5 | *Ca. aconidialis* | PV757123 | PV758689 | N/A | N/A |
| Xizang | Site 8 | 1/1 | 20_Cinnamomum camphora Leaf | (3) | CSFF 28381 | T12_B27_N/A_N/A | N/A | TB genotype 5 | *Ca. aconidialis* | PV757124 | PV758690 | N/A | N/A |
| Xizang | Site 8 | 1/1 | 20_Cinnamomum camphora Leaf | (4) | CSFF 28382 | T12_B27_N/A_N/A | N/A | TB genotype 5 | *Ca. aconidialis* | PV757125 | PV758691 | N/A | N/A |
| Xizang | Site 8 | 1/1 | 20_Cinnamomum camphora Leaf | (4) | CSFF 28383 | T12_B27_N/A_N/A | N/A | TB genotype 5 | *Ca. aconidialis* | PV757126 | PV758692 | N/A | N/A |
| Xizang | Site 8 | 1/1 | 21_Washingtonia filifera Leaf | (4) | CSFF 28384 | T12_B27_N/A_N/A | N/A | TB genotype 5 | *Ca. aconidialis* | PV757127 | PV758693 | N/A | N/A |
| Xizang | Site 8 | 1/1 | 21_Washingtonia filifera Leaf | (4) | CSFF 28385 | T12_B27_N/A_N/A | N/A | TB genotype 5 | *Ca. aconidialis* | PV757128 | PV758694 | N/A | N/A |
| Xizang | Site 8 | 1/1 | 21_Washingtonia filifera Leaf | (5) | CSFF 28386 | T12_B27_N/A_N/A | N/A | TB genotype 5 | *Ca. aconidialis* | PV757129 | PV758695 | N/A | N/A |
| Xizang | Site 8 | 1/1 | 21_Washingtonia filifera Leaf | (5) | CSFF 28387 | T12_B27_N/A_N/A | N/A | TB genotype 5 | *Ca. aconidialis* | PV757130 | PV758696 | N/A | N/A |
| Xizang | Site 8 | 1/1 | 22_Ficus benjamina Leaf | (1) | CSFF 28394 | T12_B27_N/A_N/A | N/A | TB genotype 5 | *Ca. aconidialis* | PV757137 | PV758703 | N/A | N/A |
| Xizang | Site 8 | 1/1 | 22_Ficus benjamina Leaf | (1) | CSFF 28395 | T12_B27_N/A_N/A | N/A | TB genotype 5 | *Ca. aconidialis* | PV757138 | PV758704 | N/A | N/A |
| Xizang | Site 8 | 1/1 | 22_Ficus benjamina Leaf | (4) | CSFF 28396 | T12_B27_N/A_N/A | N/A | TB genotype 5 | *Ca. aconidialis* | PV757139 | PV758705 | N/A | N/A |
| Xizang | Site 8 | 1/1 | 22_Ficus benjamina Leaf | (4) | CSFF 28397 | T12_B27_N/A_N/A | N/A | TB genotype 5 | *Ca. aconidialis* | PV757140 | PV758706 | N/A | N/A |
| Xizang | Site 8 | 1/1 | 23_Rhododendron pulchrum Leaf | (1) | CSFF 28406 | T12_B25_H11_C20 | TBHC genotype 4 | TB genotype 4 | *Ca. aconidialis* | PV757149 | PV758715 | PV757552 | PV757694 |
| Xizang | Site 8 | 1/1 | 23_Rhododendron pulchrum Leaf | (1) | CSFF 28407 | T12_B25_N/A_N/A | N/A | TB genotype 4 | *Ca. aconidialis* | PV757150 | PV758716 | N/A | N/A |
| Xizang | Site 8 | 1/1 | 23_Rhododendron pulchrum Leaf | (2) | CSFF 28408 | T12_B27_N/A_N/A | N/A | TB genotype 5 | *Ca. aconidialis* | PV757151 | PV758717 | N/A | N/A |
| Xizang | Site 8 | 1/1 | 23_Rhododendron pulchrum Leaf | (2) | CSFF 28409 | T12_B27_N/A_N/A | N/A | TB genotype 5 | *Ca. aconidialis* | PV757152 | PV758718 | N/A | N/A |
| Xizang | Site 8 | 1/1 | 23_Rhododendron pulchrum Leaf | (3) | CSFF 28410 | T12_B27_N/A_N/A | N/A | TB genotype 5 | *Ca. aconidialis* | PV757153 | PV758719 | N/A | N/A |
| Xizang | Site 8 | 1/1 | 23_Rhododendron pulchrum Leaf | (3) | CSFF 28411 | T12_B27_N/A_N/A | N/A | TB genotype 5 | *Ca. aconidialis* | PV757154 | PV758720 | N/A | N/A |
| Xizang | Site 8 | 1/1 | 23_Rhododendron pulchrum Leaf | (5) | CSFF 28412 | T12_B27_N/A_N/A | N/A | TB genotype 5 | *Ca. aconidialis* | PV757155 | PV758721 | N/A | N/A |
| Xizang | Site 8 | 1/1 | 23_Rhododendron pulchrum Leaf | (5) | CSFF 28413 | T12_B27_N/A_N/A | N/A | TB genotype 5 | *Ca. aconidialis* | PV757156 | PV758722 | N/A | N/A |
| Xizang | Site 8 | 1/1 | 26_Medicago sativa Seed | (1) | CSFF 28420 | T12_B25_H11_C20 | TBHC genotype 4 | TB genotype 4 | *Ca. aconidialis* | PV757163 | PV758729 | PV757554 | PV757696 |
| Xizang | Site 8 | 1/1 | 26_Medicago sativa Seed | (1) | CSFF 28421 | T12_B27_H11_C20 | TBHC genotype 5 | TB genotype 5 | *Ca. aconidialis* | PV757164 | PV758730 | PV757555 | PV757697 |
| Xizang | Site 8 | 1/1 | 26_Medicago sativa Seed | (2) | CSFF 28422 | T12_B27_N/A_N/A | N/A | TB genotype 5 | *Ca. aconidialis* | PV757165 | PV758731 | N/A | N/A |
| Xizang | Site 8 | 1/1 | 26_Medicago sativa Seed | (2) | CSFF 28423 | T12_B27_N/A_N/A | N/A | TB genotype 5 | *Ca. aconidialis* | PV757166 | PV758732 | N/A | N/A |
| Xizang | Site 8 | 1/1 | 26_Medicago sativa Seed | (3) | CSFF 28424 | T12_B25_N/A_N/A | N/A | TB genotype 4 | *Ca. aconidialis* | PV757167 | PV758733 | N/A | N/A |
| Xizang | Site 8 | 1/1 | 26_Medicago sativa Seed | (3) | CSFF 28425 | T12_B25_N/A_N/A | N/A | TB genotype 4 | *Ca. aconidialis* | PV757168 | PV758734 | N/A | N/A |
| Xizang | Site 8 | 1/1 | 26_Medicago sativa Seed | (4) | CSFF 28426 | T12_B27_N/A_N/A | N/A | TB genotype 5 | *Ca. aconidialis* | PV757169 | PV758735 | N/A | N/A |
| Xizang | Site 8 | 1/1 | 26_Medicago sativa Seed | (4) | CSFF 28427 | T12_B27_N/A_N/A | N/A | TB genotype 5 | *Ca. aconidialis* | PV757170 | PV758736 | N/A | N/A |
| Xizang | Site 8 | 1/1 | 26_Medicago sativa Seed | (5) | CSFF 28428 | T12_B27_N/A_N/A | N/A | TB genotype 5 | *Ca. aconidialis* | PV757171 | PV758737 | N/A | N/A |
| Xizang | Site 8 | 1/1 | 26_Medicago sativa Seed | (5) | CSFF 28429 | T12_B27_N/A_N/A | N/A | TB genotype 5 | *Ca. aconidialis* | PV757172 | PV758738 | N/A | N/A |

^a^ CSFF: Culture Collection of the Forest Pathogen Center (FPC), College of Forestry, Fujan Agricultural and Forestry University (FAFU) in Fuzhou, Fujian Province, China.

^b^ T, B, H, C indicated tef1, tub2, his3, and cmdA these four gene regions, respectively. Different numbers indicated different genotypes by each gene region sequences. N/A: information is not available.

^c^ T, B, H, C indicated tef1, tub2, his3, and cmdA these four gene regions, respectively. Different numbers indicated different genotypes by the combination of four gene region sequences. N/A: information is not available.

^d^ T, B indicated tef1, and tub2 these two gene regions. Different numbers indicated different genotypes by the combination of two gene region sequences.

^e^ tef1: translation elongation factor 1-alpha; tub2: β-tubulin; his3: histone H3; cmdA: calmodulin.

**Table S2.** The obtained *Calonectria* isolates used for phylogenetic analyses in this study.

| ***Calonectria* species** | **Genotype determined by *tef1*, *tub2*, *his3*, and *cmdA* sequences^a^** | **Code of genotype determined by *tef1*, *tub2*, *his3*, and *cmdA* sequences^b^** | **Code of genotype determined by *tef1*, and *tub2* sequences^c^** | **Sampling province** | **Total soil sample number/The soil sample number of relative province** | **Bait (ID number & species)** | **The soil isolationcup number of relative bait** | **Isolate Number^d^** | **GenBank accession numbers^e^** | | | |
| --- | --- | --- | --- | --- | --- | --- | --- | --- | --- | --- | --- | --- |
|  |  |  |  |  |  |  |  |  | ***tef1*** | ***tub2*** | ***his3*** | ***cmdA*** |
| ***Calonectria colhounii* species complex** | | | | | | | | | | | | |
| *Ca. lichi* | T28_B29_H15_C22 | TBHC genotype 47 | TB genotype 39 | Henan | 1/1 | 14_Syzygium jambos Leaf | (4) | CSFF 31718 | PV757405 | PV758971 | PV757577 | PV757719 |
| *Ca. lichi* | T28_B29_H15_C22 | TBHC genotype 47 | TB genotype 39 | Henan | 1/1 | 22_Ficus benjamina Leaf | (1) | CSFF 31721 | PV757408 | PV758974 | PV757578 | PV757720 |
| *Ca. lichi* | T28_B29_H15_C22 | TBHC genotype 47 | TB genotype 39 | Henan | 1/1 | 23_Rhododendron pulchrum Leaf | (1) | CSFF 31734 | PV757421 | PV758987 | PV757579 | PV757721 |
| *Ca. lichi* | T28_B29_H15_C22 | TBHC genotype 47 | TB genotype 39 | Henan | 1/1 | 26_Medicago sativa Seed | (3) | CSFF 31750 | PV757437 | PV759003 | PV757582 | PV757724 |
| *Ca. lichi* | T29_B29_H15_C22 | TBHC genotype 48 | TB genotype 40 | Henan | 1/1 | 19_Dianthus caryophyllus Leaf | (2) | CSFF 31680 | PV757367 | PV758933 | PV757566 | PV757708 |
| *Ca. lichi* | T29_B29_H15_C22 | TBHC genotype 48 | TB genotype 40 | Henan | 1/1 | 19_Dianthus caryophyllus Leaf | (2) | CSFF 31681 | PV757368 | PV758934 | PV757567 | PV757709 |
| *Ca. lichi* | T29_B29_H15_C22 | TBHC genotype 48 | TB genotype 40 | Henan | 1/1 | 19_Dianthus caryophyllus Leaf | (5) | CSFF 31682 | PV757369 | PV758935 | PV757568 | PV757710 |
| *Ca. lichi* | T29_B29_H15_C22 | TBHC genotype 48 | TB genotype 40 | Henan | 1/1 | 19_Dianthus caryophyllus Leaf | (5) | CSFF 31683 | PV757370 | PV758936 | PV757569 | PV757711 |
| *Ca. lichi* | T30_B32_H16_C22 | TBHC genotype 49 | TB genotype 41 | Heilongjiang | 1/1 | 18_Acacia mangium Leaf | (4) | CSFF 31597 | PV757284 | PV758850 | PV757562 | PV757704 |
| ***Ca. kyotensis* species complex** | | | | | | | | | | | | |
| *Ca. aconidialis* | T11_B25_H11_C20 | TBHC genotype 1 | TB genotype 1 | Fujian | 3/3 | 9_Lythrum salicaria Seed | (2) | CSFF 27828 | PV756571 | PV758137 | PV757498 | PV757640 |
| *Ca. aconidialis* | T11_B25_H11_C20 | TBHC genotype 1 | TB genotype 1 | Fujian | 3/3 | 13_Eucalyptus urophylla hybrid Leaf | (1) | CSFF 28004 | PV756747 | PV758313 | PV757511 | PV757653 |
| *Ca. aconidialis* | T11_B25_H11_C20 | TBHC genotype 1 | TB genotype 1 | Fujian | 3/3 | 19_Dianthus caryophyllus Leaf | (1) | CSFF 27888 | PV756631 | PV758197 | PV757504 | PV757646 |
| *Ca. aconidialis* | T11_B25_H11_C20 | TBHC genotype 1 | TB genotype 1 | Fujian | 3/3 | 26_Medicago sativa Seed | (1) | CSFF 28024 | PV756767 | PV758333 | PV757512 | PV757654 |
| *Ca. aconidialis* | T11_B26_H11_C20 | TBHC genotype 2 | TB genotype 2 | Fujian | 3/3 | 7_Dianthus caryophyllus Seed | (1) | CSFF 27850 | PV756593 | PV758159 | PV757502 | PV757644 |
| *Ca. aconidialis* | T11_B26_H11_C20 | TBHC genotype 2 | TB genotype 2 | Fujian | 3/3 | 8_Trifolium pratense Seed | (5) | CSFF 27843 | PV756586 | PV758152 | PV757500 | PV757642 |
| *Ca. aconidialis* | T11_B26_H11_C20 | TBHC genotype 2 | TB genotype 2 | Fujian | 3/3 | 16_Saccharum officinarum Leaf | (1) | CSFF 27929 | PV756672 | PV758238 | PV757509 | PV757651 |
| *Ca. aconidialis* | T11_B26_H11_C20 | TBHC genotype 2 | TB genotype 2 | Fujian | 3/3 | 17_Rosa chinensis Leaf | (1) | CSFF 27898 | PV756641 | PV758207 | PV757506 | PV757648 |
| *Ca. aconidialis* | T11_B28_H11_C20 | TBHC genotype 3 | TB genotype 3 | Fujian | 3/3 | 9_Lythrum salicaria Seed | (4) | CSFF 27830 | PV756573 | PV758139 | PV757499 | PV757641 |
| *Ca. aconidialis* | T11_B28_H11_C20 | TBHC genotype 3 | TB genotype 3 | Fujian | 3/3 | 14_Syzygium jambos Leaf | (5) | CSFF 27956 | PV756699 | PV758265 | PV757510 | PV757652 |
| *Ca. aconidialis* | T11_B28_H11_C20 | TBHC genotype 3 | TB genotype 3 | Fujian | 3/3 | 19_Dianthus caryophyllus Leaf | (2) | CSFF 27891 | PV756634 | PV758200 | PV757505 | PV757647 |
| *Ca. aconidialis* | T11_B28_H11_C20 | TBHC genotype 3 | TB genotype 3 | Fujian | 3/3 | 26_Medicago sativa Seed | (1) | CSFF 28025 | PV756768 | PV758334 | PV757513 | PV757655 |
| *Ca. aconidialis* | T12_B25_H11_C20 | TBHC genotype 4 | TB genotype 4 | Xizang | 1/1 | 8_Trifolium pratense Seed | (3) | CSFF 28366 | PV757109 | PV758675 | PV757549 | PV757691 |
| *Ca. aconidialis* | T12_B25_H11_C20 | TBHC genotype 4 | TB genotype 4 | Xizang | 1/1 | 13_Eucalyptus urophylla hybrid Leaf | (5) | CSFF 28416 | PV757159 | PV758725 | PV757553 | PV757695 |
| *Ca. aconidialis* | T12_B25_H11_C20 | TBHC genotype 4 | TB genotype 4 | Xizang | 1/1 | 23_Rhododendron pulchrum Leaf | (1) | CSFF 28406 | PV757149 | PV758715 | PV757552 | PV757694 |
| *Ca. aconidialis* | T12_B25_H11_C20 | TBHC genotype 4 | TB genotype 4 | Xizang | 1/1 | 26_Medicago sativa Seed | (1) | CSFF 28420 | PV757163 | PV758729 | PV757554 | PV757696 |
| *Ca. aconidialis* | T12_B27_H11_C20 | TBHC genotype 5 | TB genotype 5 | Xizang | 1/1 | 9_Lythrum salicaria Seed | (2) | CSFF 28358 | PV757101 | PV758667 | PV757548 | PV757690 |
| *Ca. aconidialis* | T12_B27_H11_C20 | TBHC genotype 5 | TB genotype 5 | Xizang | 1/1 | 14_Syzygium jambos Leaf | (1) | CSFF 28392 | PV757135 | PV758701 | PV757551 | PV757693 |
| *Ca. aconidialis* | T12_B27_H11_C20 | TBHC genotype 5 | TB genotype 5 | Xizang | 1/1 | 19_Dianthus caryophyllus Leaf | (1) | CSFF 28368 | PV757111 | PV758677 | PV757550 | PV757692 |
| *Ca. aconidialis* | T12_B27_H11_C20 | TBHC genotype 5 | TB genotype 5 | Xizang | 1/1 | 26_Medicago sativa Seed | (1) | CSFF 28421 | PV757164 | PV758730 | PV757555 | PV757697 |
| *Ca. aconidialis* | T13_B25_H11_C20 | TBHC genotype 6 | TB genotype 6 | Fujian | 3/3 | 4_Salvia hispanica Seed | (3) | CSFF 27871 | PV756614 | PV758180 | PV757503 | PV757645 |
| *Ca. aconidialis* | T15_B25_H11_C20 | TBHC genotype 8 | TB genotype 8 | Fujian | 3/3 | 20_Cinnamomum camphora Leaf | (5) | CSFF 27916 | PV756659 | PV758225 | PV757508 | PV757650 |
| *Ca. canadiana* | T18_B35_H13_C18 | TBHC genotype 11 | TB genotype 11 | Heilongjiang | 1/1 | 19_Dianthus caryophyllus Leaf | (4) | CSFF 31546 | PV757233 | PV758799 | PV757558 | PV757700 |
| *Ca. canadiana* | T18_B35_H13_C18 | TBHC genotype 11 | TB genotype 11 | Heilongjiang | 1/1 | 19_Dianthus caryophyllus Leaf | (4) | CSFF 31547 | PV757234 | PV758800 | PV757559 | PV757701 |
| *Ca. canadiana* | T18_B35_H13_C18 | TBHC genotype 11 | TB genotype 11 | Heilongjiang | 1/1 | 20_Cinnamomum camphora Leaf | (2) | CSFF 31562 | PV757249 | PV758815 | PV757560 | PV757702 |
| *Ca. canadiana* | T18_B35_H13_C18 | TBHC genotype 11 | TB genotype 11 | Heilongjiang | 1/1 | 21_Washingtonia filifera Leaf | (3) | CSFF 31575 | PV757262 | PV758828 | PV757561 | PV757703 |
| *Ca. canadiana* | T18_B36_H13_C19 | TBHC genotype 12 | TB genotype 12 | Henan | 1/1 | 20_Cinnamomum camphora Leaf | (4) | CSFF 31692 | PV757379 | PV758945 | PV757570 | PV757712 |
| *Ca. canadiana* | T18_B36_H13_C19 | TBHC genotype 12 | TB genotype 12 | Henan | 1/1 | 20_Cinnamomum camphora Leaf | (4) | CSFF 31693 | PV757380 | PV758946 | PV757571 | PV757713 |
| *Ca. canadiana* | T18_B36_H14_C18 | TBHC genotype 13 | TB genotype 12 | Heilongjiang | 1/1 | 9_Lythrum salicaria Seed | (1) | CSFF 31486 | PV757173 | PV758739 | PV757556 | PV757698 |
| *Ca. canadiana* | T18_B36_H14_C18 | TBHC genotype 13 | TB genotype 12 | Heilongjiang | 1/1 | 13_Eucalyptus urophylla hybrid Leaf | (1) | CSFF 31646 | PV757333 | PV758899 | PV757563 | PV757705 |
| *Ca. canadiana* | T18_B36_H14_C18 | TBHC genotype 13 | TB genotype 12 | Heilongjiang | 1/1 | 19_Dianthus caryophyllus Leaf | (1) | CSFF 31540 | PV757227 | PV758793 | PV757557 | PV757699 |
| *Ca. canadiana* | T18_B36_H14_C18 | TBHC genotype 13 | TB genotype 12 | Heilongjiang | 1/1 | 26_Medicago sativa Seed | (1) | CSFF 31666 | PV757353 | PV758919 | PV757564 | PV757706 |
| *Ca. canadiana* | T18_B37_H13_C18 | TBHC genotype 14 | TB genotype 13 | Henan | 1/1 | 16_Saccharum officinarum Leaf | (5) | CSFF 31708 | PV757395 | PV758961 | PV757572 | PV757714 |
| *Ca. canadiana* | T18_B37_H13_C18 | TBHC genotype 14 | TB genotype 13 | Henan | 1/1 | 16_Saccharum officinarum Leaf | (5) | CSFF 31709 | PV757396 | PV758962 | PV757573 | PV757715 |
| *Ca. canadiana* | T18_B37_H14_C18 | TBHC genotype 15 | TB genotype 13 | Henan | 1/1 | 14_Syzygium jambos Leaf | (1) | CSFF 31714 | PV757401 | PV758967 | PV757574 | PV757716 |
| *Ca. canadiana* | T18_B37_H14_C18 | TBHC genotype 15 | TB genotype 13 | Henan | 1/1 | 14_Syzygium jambos Leaf | (1) | CSFF 31715 | PV757402 | PV758968 | PV757575 | PV757717 |
| *Ca. canadiana* | T18_B38_H13_C18 | TBHC genotype 16 | TB genotype 14 | Henan | 1/1 | 7_Dianthus caryophyllus Seed | (2) | CSFF 31676 | PV757363 | PV758929 | PV757565 | PV757707 |
| *Ca. canadiana* | T18_B38_H13_C18 | TBHC genotype 16 | TB genotype 14 | Henan | 1/1 | 14_Syzygium jambos Leaf | (3) | CSFF 31716 | PV757403 | PV758969 | PV757576 | PV757718 |
| *Ca. canadiana* | T18_B38_H13_C18 | TBHC genotype 16 | TB genotype 14 | Henan | 1/1 | 23_Rhododendron pulchrum Leaf | (1) | CSFF 31735 | PV757422 | PV758988 | PV757580 | PV757722 |
| *Ca. canadiana* | T18_B38_H13_C18 | TBHC genotype 16 | TB genotype 14 | Henan | 1/1 | 26_Medicago sativa Seed | (2) | CSFF 31748 | PV757435 | PV759001 | PV757581 | PV757723 |
| *Ca. chinensis* | T19_B30_H22_C16 | TBHC genotype 17 | TB genotype 15 | Hainan | 2/2 | 9_Lythrum salicaria Seed | (4) | CSFF 28187 | PV756930 | PV758496 | PV757533 | PV757675 |
| *Ca. chinensis* | T19_B30_H22_C16 | TBHC genotype 17 | TB genotype 15 | Hainan | 2/2 | 12_Pinus massoniana Leaf | (1) | CSFF 28305 | PV757048 | PV758614 | PV757541 | PV757683 |
| *Ca. chinensis* | T19_B30_H22_C16 | TBHC genotype 17 | TB genotype 15 | Hainan | 2/2 | 13_Eucalyptus urophylla hybrid Leaf | (3) | CSFF 28334 | PV757077 | PV758643 | PV757544 | PV757686 |
| *Ca. chinensis* | T19_B30_H22_C16 | TBHC genotype 17 | TB genotype 15 | Hainan | 2/2 | 17_Rosa chinensis Leaf | (1) | CSFF 28233 | PV756976 | PV758542 | PV757537 | PV757679 |
| *Ca. chinensis* | T19_B31_H21_C16 | TBHC genotype 18 | TB genotype 16 | Hainan | 2/2 | 16_Saccharum officinarum Leaf | (2) | CSFF 28265 | PV757008 | PV758574 | PV757538 | PV757680 |
| *Ca. chinensis* | T20_B30_H21_C17 | TBHC genotype 19 | TB genotype 17 | Hainan | 2/2 | 1_Brassica rapa subsp. pekinensis Seed | (3) | CSFF 28214 | PV756957 | PV758523 | PV757534 | PV757676 |
| *Ca. chinensis* | T20_B30_H21_C17 | TBHC genotype 19 | TB genotype 17 | Hainan | 2/2 | 16_Saccharum officinarum Leaf | (3) | CSFF 28266 | PV757009 | PV758575 | PV757539 | PV757681 |
| *Ca. chinensis* | T20_B30_H21_C17 | TBHC genotype 19 | TB genotype 17 | Hainan | 2/2 | 19_Dianthus caryophyllus Leaf | (4) | CSFF 28229 | PV756972 | PV758538 | PV757536 | PV757678 |
| *Ca. chinensis* | T20_B30_H21_C17 | TBHC genotype 19 | TB genotype 17 | Hainan | 2/2 | 26_Medicago sativa Seed | (5) | CSFF 28356 | PV757099 | PV758665 | PV757547 | PV757689 |
| *Ca. hongkongensis* | T21_B11_H17_C11 | TBHC genotype 20 | TB genotype 18 | Hainan | 2/2 | 9_Lythrum salicaria Seed | (1) | CSFF 28180 | PV756923 | PV758489 | PV757531 | PV757673 |
| *Ca. hongkongensis* | T21_B11_H17_C11 | TBHC genotype 20 | TB genotype 18 | Hainan | 2/2 | 13_Eucalyptus urophylla hybrid Leaf | (1) | CSFF 28330 | PV757073 | PV758639 | PV757543 | PV757685 |
| *Ca. hongkongensis* | T21_B11_H17_C11 | TBHC genotype 20 | TB genotype 18 | Hainan | 2/2 | 19_Dianthus caryophyllus Leaf | (1) | CSFF 28222 | PV756965 | PV758531 | PV757535 | PV757677 |
| *Ca. hongkongensis* | T21_B11_H17_C11 | TBHC genotype 20 | TB genotype 18 | Hainan | 2/2 | 26_Medicago sativa Seed | (1) | CSFF 28348 | PV757091 | PV758657 | PV757545 | PV757687 |
| *Ca. hongkongensis* | T21_B12_H17_C11 | TBHC genotype 21 | TB genotype 19 | Hainan | 2/2 | 10_Buxus sinica Leaf | (2) | CSFF 28326 | PV757069 | PV758635 | PV757542 | PV757684 |
| *Ca. hongkongensis* | T21_B12_H17_C11 | TBHC genotype 21 | TB genotype 19 | Hainan | 2/2 | 22_Ficus benjamina Leaf | (2) | CSFF 28292 | PV757035 | PV758601 | PV757540 | PV757682 |
| *Ca. hongkongensis* | T21_B12_H17_C12 | TBHC genotype 22 | TB genotype 19 | Hainan | 2/1 | 9_Lythrum salicaria Seed | (5) | CSFF 28042 | PV756785 | PV758351 | PV757516 | PV757658 |
| *Ca. hongkongensis* | T21_B12_H17_C12 | TBHC genotype 22 | TB genotype 19 | Hainan | 2/1 | 23_Rhododendron pulchrum Leaf | (5) | CSFF 28151 | PV756894 | PV758460 | PV757525 | PV757667 |
| *Ca. hongkongensis* | T21_B13_H17_C11 | TBHC genotype 23 | TB genotype 20 | Fujian | 3/1 | 9_Lythrum salicaria Seed | (2) | CSFF 27419 | PV756162 | PV757728 | PV757444 | PV757586 |
| *Ca. hongkongensis* | T21_B13_H17_C11 | TBHC genotype 23 | TB genotype 20 | Fujian | 3/1 | 13_Eucalyptus urophylla hybrid Leaf | (4) | CSFF 27610 | PV756353 | PV757919 | PV757481 | PV757623 |
| *Ca. hongkongensis* | T21_B13_H17_C11 | TBHC genotype 23 | TB genotype 20 | Fujian | 3/2 | 17_Rosa chinensis Leaf | (1) | CSFF 27699 | PV756442 | PV758008 | PV757490 | PV757632 |
| *Ca. hongkongensis* | T21_B13_H17_C11 | TBHC genotype 23 | TB genotype 20 | Fujian | 3/3 | 20_Cinnamomum camphora Leaf | (4) | CSFF 27915 | PV756658 | PV758224 | PV757507 | PV757649 |
| *Ca. hongkongensis* | T21_B13_H17_C11 | TBHC genotype 23 | TB genotype 20 | Hainan | 2/1 | 8_Trifolium pratense Seed | (4) | CSFF 28050 | PV756793 | PV758359 | PV757517 | PV757659 |
| *Ca. hongkongensis* | T21_B13_H17_C11 | TBHC genotype 23 | TB genotype 20 | Hainan | 2/1 | 23_Rhododendron pulchrum Leaf | (2) | CSFF 28144 | PV756887 | PV758453 | PV757524 | PV757666 |
| *Ca. hongkongensis* | T21_B13_H17_C11 | TBHC genotype 23 | TB genotype 20 | Hainan | 2/2 | 9_Lythrum salicaria Seed | (2) | CSFF 28183 | PV756926 | PV758492 | PV757532 | PV757674 |
| *Ca. hongkongensis* | T21_B13_H17_C11 | TBHC genotype 23 | TB genotype 20 | Hainan | 2/2 | 26_Medicago sativa Seed | (2) | CSFF 28350 | PV757093 | PV758659 | PV757546 | PV757688 |
| *Ca. hongkongensis* | T21_B14_H17_C11 | TBHC genotype 24 | TB genotype 21 | Fujian | 3/1 | 5_Impatiens balsamina Seed | (4) | CSFF 27474 | PV756217 | PV757783 | PV757454 | PV757596 |
| *Ca. hongkongensis* | T21_B14_H17_C11 | TBHC genotype 24 | TB genotype 21 | Fujian | 3/1 | 15_Pennisetum purpureum Leaf | (4) | CSFF 27621 | PV756364 | PV757930 | PV757485 | PV757627 |
| *Ca. hongkongensis* | T21_B14_H17_C11 | TBHC genotype 24 | TB genotype 21 | Fujian | 3/1 | 18_Acacia mangium Leaf | (5) | CSFF 27545 | PV756288 | PV757854 | PV757467 | PV757609 |
| *Ca. hongkongensis* | T21_B14_H17_C11 | TBHC genotype 24 | TB genotype 21 | Fujian | 3/1 | 24_Glycine max Stem | (2) | CSFF 27598 | PV756341 | PV757907 | PV757477 | PV757619 |
| *Ca. hongkongensis* | T21_B15_H17_C11 | TBHC genotype 25 | TB genotype 22 | Hainan | 2/1 | 13_Eucalyptus urophylla hybrid Leaf | (4) | CSFF 28161 | PV756904 | PV758470 | PV757529 | PV757671 |
| *Ca. hongkongensis* | T21_B15_H17_C11 | TBHC genotype 25 | TB genotype 22 | Hainan | 2/1 | 17_Rosa chinensis Leaf | (2) | CSFF 28076 | PV756819 | PV758385 | PV757520 | PV757662 |
| *Ca. hongkongensis* | T21_B15_H17_C11 | TBHC genotype 25 | TB genotype 22 | Hainan | 2/1 | 17_Rosa chinensis Leaf | (2) | CSFF 28077 | PV756820 | PV758386 | PV757521 | PV757663 |
| *Ca. hongkongensis* | T21_B15_H17_C11 | TBHC genotype 25 | TB genotype 22 | Hainan | 2/1 | 22_Ficus benjamina Leaf | (3) | CSFF 28128 | PV756871 | PV758437 | PV757523 | PV757665 |
| *Ca. hongkongensis* | T22_B11_H17_C11 | TBHC genotype 26 | TB genotype 23 | Hainan | 2/1 | 9_Lythrum salicaria Seed | (1) | CSFF 28034 | PV756777 | PV758343 | PV757514 | PV757656 |
| *Ca. hongkongensis* | T22_B11_H17_C11 | TBHC genotype 26 | TB genotype 23 | Hainan | 2/1 | 13_Eucalyptus urophylla hybrid Leaf | (3) | CSFF 28159 | PV756902 | PV758468 | PV757528 | PV757670 |
| *Ca. hongkongensis* | T22_B11_H17_C11 | TBHC genotype 26 | TB genotype 23 | Hainan | 2/1 | 19_Dianthus caryophyllus Leaf | (3) | CSFF 28068 | PV756811 | PV758377 | PV757518 | PV757660 |
| *Ca. hongkongensis* | T22_B11_H17_C11 | TBHC genotype 26 | TB genotype 23 | Hainan | 2/1 | 26_Medicago sativa Seed | (1) | CSFF 28170 | PV756913 | PV758479 | PV757530 | PV757672 |
| *Ca. hongkongensis* | T23_B13_H17_C11 | TBHC genotype 27 | TB genotype 24 | Fujian | 3/1 | 9_Lythrum salicaria Seed | (1) | CSFF 27417 | PV756160 | PV757726 | PV757442 | PV757584 |
| *Ca. kyotensis* | T24_B16_H18_C15 | TBHC genotype 28 | TB genotype 25 | Fujian | 3/1 | 8_Trifolium pratense Seed | (4) | CSFF 27432 | PV756175 | PV757741 | PV757448 | PV757590 |
| *Ca. kyotensis* | T24_B16_H18_C15 | TBHC genotype 28 | TB genotype 25 | Fujian | 3/1 | 13_Eucalyptus urophylla hybrid Leaf | (1) | CSFF 27604 | PV756347 | PV757913 | PV757478 | PV757620 |
| *Ca. kyotensis* | T24_B16_H18_C15 | TBHC genotype 28 | TB genotype 25 | Fujian | 3/2 | 10_Buxus sinica Leaf | (4) | CSFF 27794 | PV756537 | PV758103 | PV757494 | PV757636 |
| *Ca. kyotensis* | T24_B16_H18_C15 | TBHC genotype 28 | TB genotype 25 | Fujian | 3/2 | 19_Dianthus caryophyllus Leaf | (3) | CSFF 27693 | PV756436 | PV758002 | PV757489 | PV757631 |
| *Ca. kyotensis* | T24_B17_H19_C15 | TBHC genotype 29 | TB genotype 26 | Fujian | 3/1 | 1_Brassica rapa subsp. pekinensis Seed | (5) | CSFF 27458 | PV756201 | PV757767 | PV757451 | PV757593 |
| *Ca. kyotensis* | T24_B17_H19_C15 | TBHC genotype 29 | TB genotype 26 | Fujian | 3/1 | 1_Brassica rapa subsp. pekinensis Seed | (5) | CSFF 27459 | PV756202 | PV757768 | PV757452 | PV757594 |
| *Ca. kyotensis* | T24_B18_H18_C15 | TBHC genotype 30 | TB genotype 27 | Fujian | 3/1 | 17_Rosa chinensis Leaf | (5) | CSFF 27504 | PV756247 | PV757813 | PV757460 | PV757602 |
| *Ca. kyotensis* | T24_B18_H19_C15 | TBHC genotype 31 | TB genotype 27 | Fujian | 3/1 | 7_Dianthus caryophyllus Seed | (3) | CSFF 27448 | PV756191 | PV757757 | PV757449 | PV757591 |
| *Ca. kyotensis* | T24_B18_H19_C15 | TBHC genotype 31 | TB genotype 27 | Fujian | 3/1 | 7_Dianthus caryophyllus Seed | (3) | CSFF 27449 | PV756192 | PV757758 | PV757450 | PV757592 |
| *Ca. kyotensis* | T24_B19_H18_C15 | TBHC genotype 32 | TB genotype 28 | Fujian | 3/1 | 9_Lythrum salicaria Seed | (1) | CSFF 27416 | PV756159 | PV757725 | PV757441 | PV757583 |
| *Ca. kyotensis* | T24_B19_H18_C15 | TBHC genotype 32 | TB genotype 28 | Fujian | 3/1 | 14_Syzygium jambos Leaf | (3) | CSFF 27550 | PV756293 | PV757859 | PV757468 | PV757610 |
| *Ca. kyotensis* | T24_B19_H18_C15 | TBHC genotype 32 | TB genotype 28 | Fujian | 3/2 | 8_Trifolium pratense Seed | (5) | CSFF 27652 | PV756395 | PV757961 | PV757487 | PV757629 |
| *Ca. kyotensis* | T24_B19_H18_C15 | TBHC genotype 32 | TB genotype 28 | Fujian | 3/2 | 22_Ficus benjamina Leaf | (5) | CSFF 27767 | PV756510 | PV758076 | PV757491 | PV757633 |
| *Ca. kyotensis* | T24_B20_H18_C13 | TBHC genotype 33 | TB genotype 29 | Fujian | 3/3 | 6_Sesamum indicum Seed | (1) | CSFF 27844 | PV756587 | PV758153 | PV757501 | PV757643 |
| *Ca. kyotensis* | T24_B20_H18_C14 | TBHC genotype 34 | TB genotype 29 | Fujian | 3/1 | 8_Trifolium pratense Seed | (1) | CSFF 27426 | PV756169 | PV757735 | PV757446 | PV757588 |
| *Ca. kyotensis* | T24_B20_H18_C15 | TBHC genotype 35 | TB genotype 29 | Fujian | 3/1 | 17_Rosa chinensis Leaf | (2) | CSFF 27499 | PV756242 | PV757808 | PV757459 | PV757601 |
| *Ca. kyotensis* | T24_B20_H18_C15 | TBHC genotype 35 | TB genotype 29 | Fujian | 3/1 | 23_Rhododendron pulchrum Leaf | (3) | CSFF 27590 | PV756333 | PV757899 | PV757475 | PV757617 |
| *Ca. kyotensis* | T24_B21_H20_C13 | TBHC genotype 36 | TB genotype 30 | Fujian | 3/1 | 8_Trifolium pratense Seed | (3) | CSFF 27430 | PV756173 | PV757739 | PV757447 | PV757589 |
| *Ca. kyotensis* | T24_B21_H20_C13 | TBHC genotype 36 | TB genotype 30 | Fujian | 3/1 | 16_Saccharum officinarum Leaf | (2) | CSFF 27528 | PV756271 | PV757837 | PV757463 | PV757605 |
| *Ca. kyotensis* | T24_B21_H20_C13 | TBHC genotype 36 | TB genotype 30 | Fujian | 3/1 | 21_Washingtonia filifera Leaf | (2) | CSFF 27519 | PV756262 | PV757828 | PV757461 | PV757603 |
| *Ca. kyotensis* | T24_B21_H20_C13 | TBHC genotype 36 | TB genotype 30 | Fujian | 3/1 | 24_Glycine max Stem | (1) | CSFF 27596 | PV756339 | PV757905 | PV757476 | PV757618 |
| *Ca. kyotensis* | T24_B22_H19_C13 | TBHC genotype 37 | TB genotype 31 | Fujian | 3/1 | 11_Imperata cylindrica Leaf | (2) | CSFF 27569 | PV756312 | PV757878 | PV757472 | PV757614 |
| *Ca. kyotensis* | T24_B22_H19_C13 | TBHC genotype 37 | TB genotype 31 | Fujian | 3/1 | 14_Syzygium jambos Leaf | (5) | CSFF 27555 | PV756298 | PV757864 | PV757469 | PV757611 |
| *Ca. kyotensis* | T24_B22_H19_C13 | TBHC genotype 37 | TB genotype 31 | Fujian | 3/1 | 21_Washingtonia filifera Leaf | (3) | CSFF 27521 | PV756264 | PV757830 | PV757462 | PV757604 |
| *Ca. kyotensis* | T24_B22_H19_C13 | TBHC genotype 37 | TB genotype 31 | Fujian | 3/1 | 22_Ficus benjamina Leaf | (5) | CSFF 27565 | PV756308 | PV757874 | PV757471 | PV757613 |
| *Ca. kyotensis* | T24_B23_H18_C14 | TBHC genotype 38 | TB genotype 32 | Fujian | 3/2 | 11_Imperata cylindrica Leaf | (3) | CSFF 27768 | PV756511 | PV758077 | PV757492 | PV757634 |
| *Ca. kyotensis* | T24_B23_H18_C14 | TBHC genotype 38 | TB genotype 32 | Fujian | 3/2 | 11_Imperata cylindrica Leaf | (3) | CSFF 27769 | PV756512 | PV758078 | PV757493 | PV757635 |
| *Ca. kyotensis* | T24_B24_H18_C15 | TBHC genotype 39 | TB genotype 33 | Fujian | 3/1 | 5_Impatiens balsamina Seed | (1) | CSFF 27470 | PV756213 | PV757779 | PV757453 | PV757595 |
| *Ca. kyotensis* | T24_B24_H18_C15 | TBHC genotype 39 | TB genotype 33 | Fujian | 3/1 | 19_Dianthus caryophyllus Leaf | (4) | CSFF 27492 | PV756235 | PV757801 | PV757456 | PV757598 |
| *Ca. kyotensis* | T24_B24_H18_C15 | TBHC genotype 39 | TB genotype 33 | Fujian | 3/1 | 19_Dianthus caryophyllus Leaf | (5) | CSFF 27495 | PV756238 | PV757804 | PV757458 | PV757600 |
| *Ca. kyotensis* | T24_B24_H19_C13 | TBHC genotype 40 | TB genotype 33 | Fujian | 3/1 | 18_Acacia mangium Leaf | (3) | CSFF 27541 | PV756284 | PV757850 | PV757464 | PV757606 |
| *Ca. kyotensis* | T25_B16_H18_C15 | TBHC genotype 41 | TB genotype 34 | Fujian | 3/1 | 9_Lythrum salicaria Seed | (2) | CSFF 27418 | PV756161 | PV757727 | PV757443 | PV757585 |
| *Ca. kyotensis* | T25_B16_H18_C15 | TBHC genotype 41 | TB genotype 34 | Fujian | 3/1 | 12_Pinus massoniana Leaf | (5) | CSFF 27585 | PV756328 | PV757894 | PV757473 | PV757615 |
| *Ca. kyotensis* | T25_B16_H18_C15 | TBHC genotype 41 | TB genotype 34 | Fujian | 3/1 | 13_Eucalyptus urophylla hybrid Leaf | (4) | CSFF 27611 | PV756354 | PV757920 | PV757482 | PV757624 |
| *Ca. kyotensis* | T25_B16_H18_C15 | TBHC genotype 41 | TB genotype 34 | Fujian | 3/1 | 19_Dianthus caryophyllus Leaf | (2) | CSFF 27489 | PV756232 | PV757798 | PV757455 | PV757597 |
| *Ca. kyotensis* | T25_B20_H18_C13 | TBHC genotype 42 | TB genotype 35 | Fujian | 3/2 | 24_Glycine max Stem | (5) | CSFF 27800 | PV756543 | PV758109 | PV757495 | PV757637 |
| *Ca. kyotensis* | T25_B20_H18_C15 | TBHC genotype 43 | TB genotype 35 | Fujian | 3/1 | 9_Lythrum salicaria Seed | (3) | CSFF 27420 | PV756163 | PV757729 | PV757445 | PV757587 |
| *Ca. kyotensis* | T25_B20_H18_C15 | TBHC genotype 43 | TB genotype 35 | Fujian | 3/1 | 13_Eucalyptus urophylla hybrid Leaf | (2) | CSFF 27607 | PV756350 | PV757916 | PV757479 | PV757621 |
| *Ca. kyotensis* | T25_B20_H18_C15 | TBHC genotype 43 | TB genotype 35 | Fujian | 3/1 | 23_Rhododendron pulchrum Leaf | (1) | CSFF 27586 | PV756329 | PV757895 | PV757474 | PV757616 |
| *Ca. kyotensis* | T25_B22_H18_C13 | TBHC genotype 44 | TB genotype 36 | Fujian | 3/1 | 15_Pennisetum purpureum Leaf | (2) | CSFF 27616 | PV756359 | PV757925 | PV757484 | PV757626 |
| *Ca. kyotensis* | T25_B22_H18_C13 | TBHC genotype 44 | TB genotype 36 | Fujian | 3/1 | 18_Acacia mangium Leaf | (4) | CSFF 27542 | PV756285 | PV757851 | PV757465 | PV757607 |
| *Ca. kyotensis* | T25_B22_H18_C13 | TBHC genotype 44 | TB genotype 36 | Fujian | 3/1 | 18_Acacia mangium Leaf | (4) | CSFF 27543 | PV756286 | PV757852 | PV757466 | PV757608 |
| *Ca. kyotensis* | T25_B22_H18_C13 | TBHC genotype 44 | TB genotype 36 | Fujian | 3/1 | 22_Ficus benjamina Leaf | (3) | CSFF 27560 | PV756303 | PV757869 | PV757470 | PV757612 |
| *Ca. kyotensis* | T26_B24_H18_C15 | TBHC genotype 45 | TB genotype 37 | Fujian | 3/1 | 13_Eucalyptus urophylla hybrid Leaf | (3) | CSFF 27609 | PV756352 | PV757918 | PV757480 | PV757622 |
| *Ca. kyotensis* | T27_B20_H18_C15 | TBHC genotype 46 | TB genotype 38 | Fujian | 3/1 | 13_Eucalyptus urophylla hybrid Leaf | (5) | CSFF 27613 | PV756356 | PV757922 | PV757483 | PV757625 |
| *Ca. kyotensis* | T27_B20_H18_C15 | TBHC genotype 46 | TB genotype 38 | Fujian | 3/1 | 19_Dianthus caryophyllus Leaf | (4) | CSFF 27493 | PV756236 | PV757802 | PV757457 | PV757599 |
| *Ca. pacifica* | T14_B33_H12_C21 | TBHC genotype 7 | TB genotype 7 | Fujian | 3/2 | 9_Lythrum salicaria Seed | (1) | CSFF 27634 | PV756377 | PV757943 | PV757486 | PV757628 |
| *Ca. pacifica* | T14_B33_H12_C21 | TBHC genotype 7 | TB genotype 7 | Fujian | 3/2 | 13_Eucalyptus urophylla hybrid Leaf | (1) | CSFF 27802 | PV756545 | PV758111 | PV757496 | PV757638 |
| *Ca. pacifica* | T14_B33_H12_C21 | TBHC genotype 7 | TB genotype 7 | Fujian | 3/2 | 19_Dianthus caryophyllus Leaf | (1) | CSFF 27688 | PV756431 | PV757997 | PV757488 | PV757630 |
| *Ca. pacifica* | T14_B33_H12_C21 | TBHC genotype 7 | TB genotype 7 | Fujian | 3/2 | 26_Medicago sativa Seed | (1) | CSFF 27818 | PV756561 | PV758127 | PV757497 | PV757639 |
| ***Ca. reteaudii* species complex** | | | | | | | | | | | | |
| *Ca. pseudoreteaudii* | T16_B34_H23_C23 | TBHC genotype 9 | TB genotype 9 | Hainan | 2/1 | 9_Lythrum salicaria Seed | (3) | CSFF 28039 | PV756782 | PV758348 | PV757515 | PV757657 |
| *Ca. pseudoreteaudii* | T16_B34_H23_C23 | TBHC genotype 9 | TB genotype 9 | Hainan | 2/1 | 13_Eucalyptus urophylla hybrid Leaf | (1) | CSFF 28154 | PV756897 | PV758463 | PV757526 | PV757668 |
| *Ca. pseudoreteaudii* | T16_B34_H23_C23 | TBHC genotype 9 | TB genotype 9 | Hainan | 2/1 | 16_Saccharum officinarum Leaf | (4) | CSFF 28106 | PV756849 | PV758415 | PV757522 | PV757664 |
| *Ca. pseudoreteaudii* | T16_B34_H23_C23 | TBHC genotype 9 | TB genotype 9 | Hainan | 2/1 | 19_Dianthus caryophyllus Leaf | (4) | CSFF 28070 | PV756813 | PV758379 | PV757519 | PV757661 |
| *Ca. pseudoreteaudii* | T17_B34_H23_C23 | TBHC genotype 10 | TB genotype 10 | Hainan | 2/1 | 13_Eucalyptus urophylla hybrid Leaf | (3) | CSFF 28158 | PV756901 | PV758467 | PV757527 | PV757669 |

^a^ T, B, H, C indicated tef1, tub2, his3, and cmdA these four gene regions, respectively. Different numbers indicated different genotypes by each gene region sequences.

^b^ T, B, H, C indicated tef1, tub2, his3, and cmdA these four gene regions, respectively. Different numbers indicated different genotypes by the combination of four gene region sequences.

^c^ T, B indicated tef1, and tub2 these two gene regions. Different numbers indicated different genotypes by the combination of two gene region sequences.

^d^ CSFF: Culture Collection of the Forest Pathogen Center (FPC), College of Forestry, Fujan Agricultural and Forestry University (FAFU) in Fuzhou, Fujian Province, China.

^e^ tef1: translation elongation factor 1-alpha; tub2: β-tubulin; his3: histone H3; cmdA: calmodulin.

**Table S3.** *Calonectria* spp. and isolates from published studies used for phylogenetic analyses in this study.

| **Species** | **Isolates representing the species^ab^** | **Other collection number^b^** | **Hosts** | **Area of occurrence** | **Collector** | **GenBank accession numbers^c^** | | | | References or source of data |
| --- | --- | --- | --- | --- | --- | --- | --- | --- | --- | --- |
|  |  |  |  |  |  | ***tef1*** | ***tub2*** | ***his3*** | ***cmdA*** |  |
| ***Calonectria colhounii* species complex** | | | | | | | | | | |
| *Ca. aciculata* | CERC 5342^T^ | CBS 142883; CMW 47645 | *Eucalyptus urophylla* × *E. grandis* | YunNan, China | S.F. Chen & J.Q. Li | MT412694 | MT412934 | MT335403 | MT335164 | Li et al. 2017; Liu et al. 2020 |
| *Ca. colhounii* | CBS 293.79^T^ | CMW 30999 | *Camellia sinensis* | Mauritius | A. Peerally | GQ267301 | DQ190564 | DQ190639 | GQ267373 | Peerally 1973; Crous 2002; Crous et al. 2006; Lombard et al. 2010a |
| *Ca. dianii* | CSF 6520^T^ | CGMCC 3.20446 | *Eucalyptus grandis* (leaf) | JingGu, PuEr, YunNan, China | G.Q. Li & C.F. Liu | OP322102 | OP322519 | OP321847 | OP321697 | Liu et al. 2024 |
|  | CSF 6439 | CGMCC 3.20445 | *Eucalyptus grandis* (leaf) | JingGu, PuEr, YunNan, China | G.Q. Li & C.F. Liu | OP322103 | OP322520 | OP321848 | OP321698 | Liu et al. 2024 |
| *Ca. eucalypti* | CMW 18444^T^ | CBS 125275 | *E. grandis* | Aek Nauli, Sumatra Utara, Indonesia | M.J. Wingfield | MT412774 | MT412992 | MT335483 | MT335243 | Lombard et al. 2010a; Liu et al. 2020 |
|  | CMW 18445 | CBS 125276 | *E. grandis* | Aek Nauli, Sumatra Utara, Indonesia | M.J. Wingfield | MT412775 | MT412993 | MT335484 | MT335244 | Lombard et al. 2010a; Liu et al. 2020 |
| *Ca. fujianensis* | CMW 27257^T^ | CBS 127201 | *E. grandis* | FuJian, China | M.J. Wingfield | MT412780 | MT412998 | MT335489 | MT335249 | Chen et al. 2011; Liu et al. 2020 |
|  | CMW 27254 | CBS 127200 | *E. grandis* | FuJian, China | M.J. Wingfield | MT412781 | MT412999 | MT335490 | MT335250 | Chen et al. 2011; Liu et al. 2020 |
| *Ca. honghensis* | CERC 5572^T^ | CBS 142885; CMW 47669 | Soil (*Eucalyptus* plantation) | HongHe, YunNan, China | S.F. Chen & J.Q. Li | MT412787 | MT413005 | MT335496 | MT335256 | Li et al. 2017; Liu et al. 2020 |
|  | CERC 5571 | CBS 142884; CMW 47668 | Soil (*Eucalyptus* plantation) | HongHe, YunNan, China | S.F. Chen & J.Q. Li | MT412788 | MT413006 | MT335497 | MT335257 | Li et al. 2017; Liu et al. 2020 |
| *Ca. indusiata* | CBS 144.36^T^ | CMW 23699 | *Camellia sinensis* | Sri lanka | N/A | GQ267332 | GQ267239 | GQ267262 | GQ267453 | Crous 2002; Lombard et al. 2010a, 2016; Marin-Felix et al. 2017 |
|  | CBS 114684 | CMW 51213; CPC 2446; UFV 16 | *Rhododendron* sp. | Florida, USA | N.E. El-Gholl | GQ267333 | AF232862 | DQ190653 | GQ267454 | Crous et al. 1999, 2006; Crous 2002 |
| *Ca. lichi* | CERC 8866^T^ | N/A | Soil | HeNan, China | S.F. Chen | MT412809 | MT413023 | MT335518 | MT335278 | Liu & Chen 2017; Liu et al. 2020 |
|  | CERC 8850 | N/A | Soil | HeNan, China | S.F. Chen | MT412810 | MT413024 | MT335519 | MT335279 | Liu & Chen 2017; Liu et al. 2020 |
| *Ca. macroconidialis* | CBS 114880^T^ | CMW 51219; CPC 307; PPRI 4000 | *E. grandis* | Sabie, Mpumalanga, South Africa | P.W. Crous | MT412813 | MT413027 | MT335522 | MT335282 | Crous 2002; Crous et al. 2006; Lombard et al. 2010b; Liu et al. 2020 |
|  | CBS 110798 | CMW 51817; CPC 410 | *E. grandis* (roots) | Sabie, Mpumalanga, South Africa | P.W. Crous | MT412814 | MT413028 | MT335523 | MT335283 | Lombard et al. 2016; Liu et al. 2020 |
| *Ca. madagascariensis* | CMW 23686^T^ | CBS 114572; CPC 2252 | Soil | Rona, Madagascar | J.E. Taylor | MT412815 | MT413029 | MT335524 | MT335284 | Crous 2002; Crous et al. 2006; Lombard et al. 2010a; Liu et al. 2020 |
|  | CMW 30993 | CBS 114571; CPC 2253 | Soil | Rona, Madagascar | J.E. Taylor | MT412816 | MT413030 | MT335525 | MT335285 | Crous 2002; Crous et al. 2006; Lombard et al. 2010a; Liu et al. 2020 |
| *Ca. minensis* | CSF 9941^T^ | CGMCC 3.18877 | Soil (*Eucalyptus* plantation) | XinLuo, LongYan, FuJian, China | S.F. Chen, Q.L. Liu & F.F. Liu | OK253814 | OK253967 | OK253403 | OK253259 | Liu et al. 2022 |
|  | CSF 9975 | CGMCC 3.18881 | Soil (natural forest area) | LianCheng, LongYan, FuJian, China | S.F. Chen, Q.L. Liu & F.F. Liu | OK253816 | OK253969 | OK253405 | OK253261 | Liu et al. 2022 |
| *Ca. monticola* | CBS 140645^T^ | CPC 28835 | Soil | Chiang Mai, Thailand | P.W. Crous | KT964773 | KT964769 | N/A | KT964771 | Crous et al. 2015 |
|  | CPC 28836 | N/A | Soil | Chiang Mai, Thailand | P.W. Crous | KT964774 | KT964770 | N/A | KT964772 | Crous et al. 2015 |
| *Ca. paracolhounii* | CBS 114679^T^ | CMW 51212; CPC 2445 | N/A | USA | A.Y. Rossman | KX784714 | KX784644 | N/A | KX784582 | Lombard et al. 2016; Marin-Felix et al. 2017 |
|  | CBS 114705 | CMW 51215; CPC 2423 | Fruit of *Annona reticulata* | Australia | D. Hutton | KX784715 | KX784645 | N/A | N/A | Lombard et al. 2016; Marin-Felix et al. 2017 |
| *Ca. populi* | NTCC 1299^T^ | N/A | *Populus deltoides* | FRI, Dehradun, India | A. Saini & S. Pandey | OR199919 | OR199921 | OR199917 | OR199915 | Saini and Pandey 2024 |
|  | NTCC 1300 | N/A | *Populus deltoides* | FRI, Dehradun, India | A. Saini & S. Pandey | OR199920 | OR199922 | OR199918 | OR199916 | Saini and Pandey 2024 |
| *Ca. xianrensis* | CSF 12909^T^ | CGMCC 3.19584 | Soil (near *Eucalyptus* plantation) | Dacheng Town, Gaozhou County, Maoming Region, GuangDong, China | S.F. Chen, Q.C. Wang & W. Wang | MK962869 | MK962833 | MK962857 | MK962845 | Wang et al. 2019 |
|  | CSF 12908 | CGMCC 3.19518 | Soil (near *Eucalyptus* plantation) | Dacheng Town, Gaozhou County, Maoming Region, GuangDong, China | S.F. Chen,Q.C. Wang& W. Wang | MK962868 | MK962832 | MK962856 | MK962844 | Wang et al. 2019 |
| ***Ca. kyotensis* species complex** | | | | | | | | | | |
| *Ca. aconidialis* | CMW 35174^T^ | CBS 136086; CERC 1850 | Soil (*Eucalyptus* plantation) | HaiNan, China | X. Mou & S.F. Chen | MT412695 | OK357463 | MT335404 | MT335165 | Lombard et al. 2015a; Liu et al. 2020; Liu et al. 2021 |
|  | CMW 35384 | CBS 136091; CERC 1886 | Soil (*Eucalyptus* plantation) | HaiNan, China | X. Mou & S.F. Chen | MT412696 | OK357464 | MT335405 | MT335166 | Lombard et al. 2015a; Liu et al. 2020; Liu et al. 2021 |
| *Ca. aeknauliensis* | CMW 48253^T^ | CBS 143559 | Soil (*Eucalyptus* plantation) | Aek Nauli, North Sumatra, Indonesia | M.J. Wingfield | MT412710 | OK357465 | MT335419 | MT335180 | Pham et al. 2019; Liu et al. 2020; Liu et al. 2021 |
|  | CMW 48254 | CBS 143560 | Soil (*Eucalyptus* plantation) | Aek Nauli, North Sumatra, Indonesia | M.J. Wingfield | MT412711 | OK357466 | MT335420 | MT335181 | Pham et al. 2019; Liu et al. 2020; Liu et al. 2021 |
| *Ca. asiatica* | CBS 114073^T^ | CMW 23782; CPC 3900 | Debris (leaf litter) | Prathet Thai, Thailand | N.L. Hywel-Jones | AY725705 | AY725616 | AY725658 | AY725741 | Crous et al. 2004; Lombard et al. 2010a |
| *Ca. borneana* | CMW 50782^T^ | CBS 144553 | Soil (*Eucalyptus* plantation) | Sabah, Tawau, Brumas, Malaysia | N.Q. Pham, Marincowitz & M.J. Wingfield | OL635019 | N/A | OL635043 | OL635067 | Pham et al. 2022 |
|  | CMW 50832 | CBS 144551 | Soil (*Eucalyptus* plantation) | Sabah, Tawau, Brumas, Malaysia | N.Q. Pham, Marincowitz & M.J. Wingfield | OL635017 | N/A | OL635041 | OL635065 | Pham et al. 2022 |
| *Ca. brassicicola* | CBS 112841^T^ | CMW 51206; CPC 4552 | Soil at *Brassica* sp. | Indonesia | M.J. Wingfield | KX784689 | KX784619 | N/A | KX784561 | Lombard et al. 2016 |
| *Ca. bumicola* | CMW 48257^T^ | CBS 143575 | Soil (*Eucalyptus* plantation) | Aek Nauli, North Sumatra, Indonesia | M.J. Wingfield | MT412736 | OK357467 | MT335445 | MT335205 | Pham et al. 2019; Liu et al. 2020; Liu et al. 2021 |
| *Ca. canadiana* | CMW 23673^T^ | CBS 110817; STE-U 499 | *Picea* sp. | Canada | S. Greifenhagen | MT412737 | MT412958 | MT335446 | MT335206 | Kang et al. 2001b; Crous 2002; Lechat et al. 2010; Liu et al. 2020 |
|  | CERC 8952 | N/A | Soil | HeNan, China | S.F. Chen | MT412821 | MT413035 | MT335530 | MT335290 | Liu & Chen 2017; Liu et al. 2020 |
| *Ca. cassiae* | ZHKUCC 21-0011^T^ | N/A | *Cassia surattensi* | GuangZhou, GuangDong, China | Y.X. Zhang | MZ516860 | MZ516863 | N/A | ON260790 | Zhang et al. 2022 |
|  | ZHKUCC 21-0012 | N/A | N/A | N/A | N/A | MZ516861 | MZ516864 | N/A | ON260791 | Zhang et al. 2022 |
| *Ca. chinensis* | CMW 23674^T^ | CBS 114827; CPC 4101 | Soil | Hong Kong, China | E.C.Y. Liew | MT412751 | MT412972 | MT335460 | MT335220 | Crous et al. 2004; Lombard et al. 2010a; Liu et al. 2020 |
|  | CMW 30986 | CBS 112744; CPC 4104 | Soil | Hong Kong, China | E.C.Y. Liew | MT412752 | MT412973 | MT335461 | MT335221 | Crous et al. 2004; Lombard et al. 2010a; Liu et al. 2020 |
| *Ca. cochinchinensis* | CMW 49915^T^ | CBS 143567 | Soil (*Hevea brasiliensis* plantation) | Duong Minh Chau, Tay Ninh, Vietnam | N.Q. Pham, Q.N. Dang & T.Q. Pham | MT412756 | MT412977 | MT335465 | MT335225 | Pham et al. 2019; Liu et al. 2020 |
|  | CMW 47186 | CBS 143568 | Soil (*A. auriculiformis* plantation) | Song May, Dong Nai, Vietnam | N.Q. Pham & T.Q. Pham | MT412757 | MT412978 | MT335466 | MT335226 | Pham et al. 2019; Liu et al. 2020 |
| *Ca. colombiensis* | CMW 23676^T^ | CBS 112220; CPC 723 | Soil (*E. grandis* trees) | La Selva, Colombia | M.J. Wingfield | MT412759 | MT412980 | MT335468 | MT335228 | Crous et al. 2004; Liu et al. 2020 |
|  | CMW 30985 | CBS 112221; CPC 724 | Soil (*E. grandis* trees) | La Selva, Colombia | M.J. Wingfield | MT412760 | MT412981 | MT335469 | MT335229 | Crous et al. 2004; Liu et al. 2020 |
| *Ca. curvispora* | CMW 23693^T^ | CBS 116159; CPC 765 | Soil | Tamatave, Madagascar | P.W. Crous | MT412763 | OK357468 | MT335472 | MT335232 | Victor et al. 1997; Crous 2002; Lombard et al. 2010a, 2015a; Liu et al. 2020; Liu et al. 2021 |
|  | CMW 48245 | CBS 143565 | Soil (*Eucalyptus* plantation) | Aek Nauli, North Sumatra, Indonesia | M.J. Wingfield | MT412764 | N/A | MT335473 | MT335233 | Pham et al. 2019; Liu et al. 2020 |
| *Ca. heveicola* | CMW 49913^T^ | CBS 143570 | Soil (*Hevea brasiliensis* plantation) | Bau Bang, Binh Duong, Vietnam | N.Q. Pham, Q.N. Dang & T.Q. Pham | MT412786 | MT413004 | MT335495 | MT335255 | Pham et al. 2019; Liu et al. 2020 |
|  | CMW 49928 | CBS 143571 | Soil | Bu Gia Map National Park, Binh Phuoc, Vietnam | N.Q. Pham, Q.N. Dang & T.Q. Pham | MT412811 | MT413025 | MT335520 | MT335280 | Pham et al. 2019; Liu et al. 2020 |
| *Ca. hongkongensis* | CBS 114828^T^ | CMW 51217; CPC 4670 | Soil | Hong Kong, China | M.J. Wingfield | MT412789 | MT413007 | MT335498 | MT335258 | Crous et al. 2004; Liu et al. 2020 |
|  | CERC 3570 | CMW 47271 | Soil (*Eucalyptus* plantation) | BeiHai, Guangxi, China | S.F. Chen,J.Q. Li &G.Q. Li | MT412791 | MT413009 | MT335500 | MT335260 | Li et al. 2017; Liu et al. 2020 |
| *Ca. ilicicola* | CMW 30998^T^ | CBS 190.50; IMI 299389; STE-U 2482 | *Solanum tuberosum* | Bogor, Java, Indonesia | K.B. Boedijn & J. Reitsma | MT412797 | OK357469 | MT335506 | MT335266 | Boedijn & Reitsma 1950; Crous 2002; Lombard et al. 2010a; Liu et al. 2020; Liu et al. 2021 |
| *Ca. indonesiae* | CMW 23683^T^ | CBS 112823; CPC 4508 | *Syzygium aromaticum* | Warambunga, Indonesia | M.J. Wingfield | MT412798 | MT413015 | MT335507 | MT335267 | Crous et al. 2004; Liu et al. 2020 |
|  | CBS 112840 | CMW 51205; CPC 4554 | *S. aromaticum* | Warambunga, Indonesia | M.J. Wingfield | MT412799 | MT413016 | MT335508 | MT335268 | Crous et al. 2004; Liu et al. 2020 |
| *Ca. kyotensis* | CBS 114525^T^ | ATCC 18834; CMW 51824; CPC 2367 | *Robinia pseudoacacia* | Japan | T. Terashita | MT412802 | MT413019 | MT335511 | MT335271 | Terashita 1968; Crous 2002; Lombard et al. 2016; Liu et al. 2020 |
|  | CBS 114550 | CMW 51825; CPC 2351 | Soil | China | M.J. Wingfield | MT412777 | MT412995 | MT335486 | MT335246 | Lombard et al. 2016; Liu et al. 2020 |
| *Ca. ladang* | CMW 50776^T^ | CBS 144550 | Soil (*Eucalyptus* plantation) | Sabah, Tawau, Brumas, Malaysia | N.Q. Pham, Marincowitz & M.J. Wingfield | OL635027 | N/A | OL635051 | OL635075 | Pham et al. 2022 |
|  | CMW 50775 | CBS 144549 | Soil (*Eucalyptus* plantation) | Sabah, Tawau, Brumas, Malaysia | N.Q. Pham, Marincowitz & M.J. Wingfield | OL635026 | N/A | OL635050 | OL635074 | Pham et al. 2022 |
| *Ca. lantauensis* | CERC 3302^T^ | CBS 142888; CMW 47252 | Soil | LiDao, Hong Kong, China | M.J. Wingfield & S.F. Chen | MT412803 | OK357470 | MT335512 | MT335272 | Li et al. 2017; Liu et al. 2020; Liu et al. 2021 |
|  | CERC 3301 | CBS 142887; CMW 47251 | Soil | LiDao, Hong Kong, China | M.J. Wingfield & S.F. Chen | MT412804 | OK357471 | MT335513 | MT335273 | Li et al. 2017; Liu et al. 2020; Liu et al. 2021 |
| *Ca. lateralis* | CMW 31412^T^ | CBS 136629 | Soil (*Eucalyptus* plantation) | GuangXi, China | X. Zhou, G. Zhao & F. Han | MT412805 | MT413020 | MT335514 | MT335274 | Lombard et al. 2015a; Liu et al. 2020 |
| *Ca. malesiana* | CMW 23687^T^ | CBS 112752; CPC 4223 | Soil | Northern Sumatra, Indonesia | M.J. Wingfield | MT412817 | MT413031 | MT335526 | MT335286 | Crous et al. 2004; Liu et al. 2020 |
|  | CBS 112710 | CMW 51199; CPC 3899 | Leaf litter | Prathet, Thailand | N.L. Hywel-Jones | MT412818 | MT413032 | MT335527 | MT335287 | Crous et al. 2004; Liu et al. 2020 |
| *Ca. pacifica* | CMW 16726^T^ | A 1568; CBS 109063; IMI 354528; STE-U 2534 | *Araucaria heterophylla* | Hawaii, USA | M. Aragaki | MT412842 | OK357472 | MT335551 | MT335311 | Kang et al. 2001b; Crous 2002; Crous et al. 2004; Liu et al. 2020; Liu et al. 2021 |
|  | CMW 30988 | CBS 114038 | *Ipomoea aquatica* | Auckland, New Zealand | C.F. Hill | MT412843 | OK357473 | MT335552 | MT335312 | Crous 2002; Crous et al. 2004; Lombard et al. 2010a; Liu et al. 2020; Liu et al. 2021 |
| *Ca. penicilloides* | CMW 23696^T^ | CBS 174.55; STE-U 2388 | *Prunus* sp. | Hatizyo Island, Japan | M. Ookubu | MT412869 | MT413081 | MT335578 | MT335338 | Tubaki 1958; Crous 2002; Liu et al. 2020 |
| *Ca. pseudomalesiana* | CMW 50821^T^ | CBS 144563 | Soil (*Eucalyptus* plantation) | Sabah, Tawau, Brumas, Malaysia | N.Q. Pham, Marincowitz & M.J. Wingfield | OL635028 | OL635137 | OL635052 | OL635076 | Pham et al. 2022 |
|  | CMW 50779 | CBS 144668 | Soil (*Eucalyptus* plantation) | Sabah, Tawau, Brumas, Malaysia | N.Q. Pham, Marincowitz & M.J. Wingfield | OL635029 | OL635138 | OL635053 | OL635077 | Pham et al. 2022 |
| *Ca. singaporensis* | CBS 146715^T^ | MUCL 048320 | leaf litter (submerged in a small stream) | South East Asian rainforest, Mac Ritchie Reservoir, Singapore | C. Decock | MW890086 | MW890124 | MW890055 | MW890042 | Crous et al. 2021 |
|  | CBS 146713 | MUCL 048171 | leaf litter (submerged in a small stream) | South East Asian rainforest, Mac Ritchie Reservoir, Singapore | C. Decock | MW890084 | MW890123 | MW890053 | MW890040 | Crous et al. 2021 |
| *Ca. sumatrensis* | CMW 23698^T^ | CBS 112829; CPC 4518 | Soil | Northern Sumatra, Indonesia | M.J. Wingfield | MT412913 | OK357474 | MT335622 | MT335382 | Crous et al. 2004; Liu et al. 2020; Liu et al. 2021 |
|  | CMW 30987 | CBS 112934; CPC 4516 | Soil | Northern Sumatra, Indonesia | M.J. Wingfield | MT412914 | OK357475 | MT335623 | MT335383 | Crous et al. 2004; Liu et al. 2020; Liu et al. 2021 |
| *Ca. syzygiicola* | CBS 112831^T^ | CMW 51204; CPC 4511 | *Syzygium aromaticum* | Sumatra, Indonesia | M.J. Wingfield | KX784736 | KX784663 | N/A | N/A | Lombard et al. 2016 |
| *Ca. tanah* | CMW 50777^T^ | CBS 144562 | Soil (*Eucalyptus* plantation) | Sabah, Tawau, Brumas, Malaysia | N.Q. Pham, Marincowitz & M.J. Wingfield | OL635040 | OL635146 | OL635064 | OL635088 | Pham et al. 2022 |
|  | CMW 50771 | CBS 144560 | Soil (*Eucalyptus* plantation) | Sabah, Tawau, Brumas, Malaysia | N.Q. Pham, Marincowitz & M.J. Wingfield | OL635038 | OL635144 | OL635062 | OL635086 | Pham et al. 2022 |
| *Ca. uniseptata* | CBS 413.67^T^ | CMW 23678; CPC 2391; IMI 299577 | *Paphiopedilum callosum* | Celle, Germany | W. Gerlach | GQ267307 | GQ267208 | GQ267248 | GQ267379 | Lombard et al. 2016 |
| *Ca. yunnanensis* | CERC 5339^T^ | CBS 142897; CMW 47644 | Soil (*Eucalyptus* plantation) | YunNan, China | S.F. Chen &J.Q. Li | MT412927 | MT413134 | MT335636 | MT335396 | Li et al. 2017; Liu et al. 2020 |
|  | CERC 5337 | CBS 142895; CMW 47642 | Soil (*Eucalyptus* plantation) | YunNan, China | S.F. Chen &J.Q. Li | MT412928 | MT413135 | MT335637 | MT335397 | Li et al. 2017; Liu et al. 2020 |
| ***Ca. reteaudii* species complex** | | | | | | | | | | |
| *Ca. acaciicola* | CMW 47173^T^ | CBS 143557 | Soil (*Acacia auriculiformis* plantation) | Do Luong, Nghe An, Vietnam | N.Q. Pham & T.Q. Pham | MT412690 | MT412930 | MT335399 | MT335160 | Pham et al. 2019; Liu et al. 2020 |
|  | CMW 47174 | CBS 143558 | Soil (*A. auriculiformis* plantation) | Do Luong, Nghe An, Vietnam | N.Q. Pham & T.Q. Pham | MT412691 | MT412931 | MT335400 | MT335161 | Pham et al. 2019; Liu et al. 2020 |
| *Ca. acicola* | CMW 30996^T^ | N/A | *Phoenix canariensis* | Northland, New Zealand | H. Pearson | MT412692 | MT412932 | MT335401 | MT335162 | Gadgil & Dick 2004; Lombard et al. 2010a; Liu et al. 2020 |
|  | CBS 114812 | CMW 51216 | *P. canariensis* | Northland, New Zealand | H. Pearson | MT412693 | MT412933 | MT335402 | MT335163 | Gadgil & Dick 2004; Lombard et al. 2010a; Liu et al. 2020 |
| *Ca. australiensis* | CMW 23669^T^ | CBS 112954; CPC 4714 | *Ficus pleurocarpa* | Queensland, Australia | C. Pearce & B. Paulus | MT412723 | MT412946 | MT335432 | MT335192 | Crous et al. 2006; Lombard et al. 2010a; Liu et al. 2020 |
| *Ca. crousiana* | CMW 27249^T^ | CBS 127198 | *E. grandis* | FuJian, China | M.J. Wingfield | MT412761 | MT412982 | MT335470 | MT335230 | Chen et al. 2011; Liu et al. 2020 |
|  | CMW 27253 | CBS 127199 | *E. grandis* | FuJian, China | M.J. Wingfield | MT412762 | MT412983 | MT335471 | MT335231 | Chen et al. 2011; Liu et al. 2020 |
| *Ca. guangdongensis* | ZHKUCC 21-0062^T^ | N/A | *Heliconia metallica* | GunagDong, China | Y.X. Zhang | MZ491149 | MZ491171 | N/A | MZ491127 | Zhang et al. 2022 |
|  | ZHKUCC 21-0063 | N/A | N/A | N/A | N/A | MZ491150 | MZ491172 | N/A | MZ491128 | Zhang et al. 2022 |
| *Ca. lombardiana* | CMW 30602^T^ | CBS 112634; CPC 4233; Lynfield 417 | *Xanthorrhoea australis* | Victoria, Australia | T. Baigent | MT412926 | MT413133 | MT335635 | MT335395 | Crous 2002; Crous et al. 2006; Lombard et al. 2010b; Liu et al. 2020 |
| *Ca. melaleucae* | ZHKUCC 21-0066^T^ | N/A | *Melaleuca bracteata* | GuangZhou, GunagDong, China | Y.X. Zhang | MZ491132 | MZ491154 | N/A | MZ491110 | Zhang et al. 2022 |
|  | ZHKUCC 21-0067 | N/A | N/A | N/A | N/A | MZ491133 | MZ491155 | N/A | MZ491111 | Zhang et al. 2022 |
| *Ca. multiseptata* | CMW 23692^T^ | CBS 112682; CPC 1589 | *E. grandis* | North Sumatra, Indonesia | M.J. Wingfield | MT412830 | MT413044 | MT335539 | MT335299 | Crous et al. 2004; Lombard et al. 2010a; Liu et al. 2020 |
| *Ca. pseudoreteaudii* | CMW 25310^T^ | CBS 123694 | *E. urophylla* × *E. grandis* | GuangDong, China | M.J. Wingfield & X.D. Zhou | MT412885 | MT413096 | MT335594 | MT335354 | Lombard et al. 2010b; Liu et al. 2020 |
|  | CMW 25292 | CBS 123696 | *E. urophylla* × *E. grandis* | GuangDong, China | M.J. Wingfield & X.D. Zhou | MT412886 | MT413097 | MT335595 | MT335355 | Lombard et al. 2010b; Liu et al. 2020 |
| *Ca. queenslandica* | CMW 30604^T^ | CBS 112146; CPC 3213 | *E. urophylla* | Lannercost, Queensland, Australia | B. Brown | MT412898 | MT413108 | MT335607 | MT335367 | Kang et al. 2001a; Lombard et al. 2010b; Liu et al. 2020 |
|  | CMW 30603 | CBS 112155; CPC 3210 | *E. pellita* | Lannercost, Queensland, Australia | P.Q Thu &K.M. Old | MT412899 | MT413109 | MT335608 | MT335368 | Kang et al. 2001a; Lombard et al. 2010b; Liu et al. 2020 |
| *Ca. reteaudii* | CMW 30984^T^ | CBS 112144; CPC 3201 | *E. camaldulensis* | Chon Thanh, Binh Phuoc, Vietnam | M.J. Dudzinski & P.Q. Thu | MT412901 | MT413111 | MT335610 | MT335370 | Kang et al. 2001a; Crous 2002; Crous et al. 2006; Liu et al. 2020 |
|  | CMW 16738 | CBS 112143; CPC 3200 | *Eucalyptus* leaves | Binh Phuoc, Vietnam | M.J. Dudzinski & P.Q. Thu | MT412902 | MT413112 | MT335611 | MT335371 | Kang et al. 2001a; Crous 2002; Crous et al. 2006; Liu et al. 2020 |
| *Ca. strelitziae* | ZHKUCC 21-0019^T^ | N/A | *Strelitzia reginae* | GuangZhou, GunagDong, China | Y.X. Zhang & C.T. Chen | MZ491129 | MZ491151 | N/A | MZ491105 | Zhang et al. 2022 |
|  | ZHKUCC 21-0047 | N/A | N/A | N/A | N/A | MZ491130 | MZ491152 | N/A | MZ491106 | Zhang et al. 2022 |
| *Curvicladiella cignea* | CBS 109167^T^ | CPC 1595; MUCL 40269 | Decaying leaf | French Guiana | C. Decock | KM231867 | KM232002 | KM231461 | KM231287 | Decock & Crous 1998; Crous et al. 2006; Lombard et al. 2015b |
|  | CBS 109168 | CPC 1594; MUCL 40268 | Decaying seed | French Guiana | C. Decock | KM231868 | KM232003 | KM231460 | KM231286 | Decock & Crous 1998; Crous et al. 2006; Lombard et al. 2015b |

^a^ T: ex-type isolates of the *Calonectria* species.

^b^ ATCC: American Type Culture Collection, Virginia, USA; CBS: Westerdijk Fungal Biodiversity Institute, Utrecht, The Netherlands; CERC: China Eucalypt Research Centre, ZhanJiang, GuangDong Province, China; CGMCC: China General Microbiological Culture Collection Center, Beijing, China; CMW: Culture collection of the Forestry and Agricultural Biotechnology Institute (FABI), University of Pretoria, Pretoria, South Africa; CPC: Pedro Crous working collection housed at Westerdijk Fungal Biodiversity Institute; CSF: Culture Collection from Southern Forests (CSF), ZhanJiang, GuangDong Province, China; IMI: International Mycological Institute, CABI Bioscience, Egham, Bakeham Lane, UK; MUCL: Mycotheque, Laboratoire de Mycologie Systematique st Appliqee, I’Universite, Louvian-la-Neuve, Belgium; NTCC: National Type Culture Collection, FRI, Dehradun, India; PPRI: Plant Protection Research Institute, Pretoria, South Africa; STE-U: Department of Plant Pathology, University of Stellenbosch, South Africa; UFV: Universidade Federal de Viçosa, Viçosa, Brazil; ZHKUCC: The culture collection of Zhongkai University of Agriculture and Engineering. N/A: information is not available.

^c^ *tef1*: translation elongation factor 1-alpha; *tub2*: β-tubulin; *his3*: histone H3; *cmdA*: calmodulin.

**References**

Boedijn KB, Reitsma J (1950) Notes on the genus *Cylindrocladium* (Fungi: *Mucedineae*). Reinwardtia 1: 51–60.

Chen SF, Lombard L, Roux J, Xie YJ, Wingfield MJ, Zhou XD (2011) Novel species of *Calonectria* associated with *Eucalyptus* leaf blight in Southeast China. Persoonia 26: 1–12.

Crous PW, Kang JC, Schoch CL, Mchau GRA (1999) Phylogenetic relationships of *Cylindrocladium pseudogracile* and *Cylindrocladium rumohrae* with morphologically similar taxa, based on morphology and DNA sequences of internal transcribed spacers and β-tubulin. Canadian Journal of Botany 77: 1813–1820.

Crous PW (2002) Taxonomy and pathology of *Cylindrocladium* (*Calonectria*) and allied genera. APS Press, St. Paul, Minnesota, USA.

Crous PW, Groenewald JZ, Risède JM, Simoneau P, Hyde KD (2004) *Calonectria* species and their *Cylindrocladium* anamorphs: species with sphaeropedunculate vesicles. Studies in Mycology 50: 415–430.

Crous PW, Groenewald JZ, Risède JM, Simoneau P, Hyde KD (2006) *Calonectria* species and their *Cylindrocladium* anamorphs: species with clavate vesicles. Studies in Mycology 55: 213–226.

Crous PW, Wingfield MJ, Le Roux JJ, Richardson DM, Strasberg D, Shivas RG, Alvarado P, Edwards J, Moreno G, Sharma R, Sonawane MS, Tan YP, Altés A, Barasubiye T, Barnes CW, Blanchette RA, Boertmann D, Bogo A, Carlavilla JR, Cheewangkoon R, Daniel R, de Beer ZW, Yáñez-Morales MDJ, Duong TA, Fernández-Vicente J, Geering ADW, Guest DI, Held BW, Heykoop M, Hubka V, Ismail AM, Kajale SC, Khemmuk W, Kolarík M, Kurli R, Lebeuf R, Lévesque CA, Lombard L, Magista D, Manjón JL, Marincowitz S, Mohedano JM, Nováková A, Oberlies NH, Otto EC, Paguigan ND, Pascoe IG, Pérez-Butrón JL, Perrone G, Rahi P, Raja HA, Rintoul T, Sanhueza RMV, Scarlett K, Shouche YS, Shuttleworth LA, Taylor PWJ, Thorn RG, Vawdrey LL, Solano-Vidal R, Voitk A, Wong PTW, Wood AR, Zamora JC, Groenewald JZ (2015) Fungal Planet description sheets: 371–399. Persoonia 35: 264–327.

Crous PW, Hernández-Restrepo M, Schumacher RK, Cowan DA, Maggs-Kölling G, Marais E, Wingfield MJ, Yilmaz N, Adan OCG, Akulov A, Álvarez Duarte E, Berraf-Tebbal A, Bulgakov TS, Carnegie AJ, de Beer ZW, Decock C, Dijksterhuis J, Duong TA, Eichmeier A, Hien LT, Houbraken JAMP, Khanh TN, Liem NV, Lombard L, Lutzoni FM, Miadlikowska JM, Nel WJ, Pascoe IG, Roets F, Roux J, Samson RA, Shen M, Spetik M, Thangavel R, Thanh HM, Thao LD, van Nieuwenhuijzen EJ, Zhang JQ, Zhang Y, Zhao LL, Groenewald JZ (2021) New and Interesting Fungi. 4. Fungal Systematics and Evolution 7: 255–343.

Decock C, Crous PW (1998) *Curvicladium* gen. nov., a new hyphomycete genus from French Guiana. Mycologia 90: 276–281.

Gadgil P, Dick M (2004) Fungi silvicolae novazelandiae: 10. New Zealand Journal of Forestry Science 44: 1–7.

Kang JC, Crous PW, Old KM, Dudzinski MJ (2001a) Non-conspecificity of *Cylindrocladium quinqueseptatum* and *Calonectria quinqueseptata* based on a β-tubulin gene phylogeny and morphology. Canadian Journal of Botany 79: 1241–1247.

Kang JC, Crous PW, Schoch CL (2001b) Species concepts in the *Cylindrocladium floridanum* and *Cy. spathiphylli* complexes (*Hypocreaceae*) based on multi-allelic sequence data, sexual compatibility and morphology. Systematic and Applied Microbiology 24: 206–217.

Lechat C, Crous PW, Groenewald JZ (2010) The enigma of *Calonectria* species occurring on leaves of *Ilex aquifolium* in Europe. IMA Fungus 1: 101–108.

Li JQ, Wingfield MJ, Liu QL, Barnes I, Roux J, Lombard L, Crous PW, Chen SF (2017) *Calonectria* species isolated from *Eucalyptus* plantations and nurseries in South China. IMA Fungus 8: 259–286.

Liu LL, Wu WX, Chen SF (2021) Species diversity and distribution characteristics of *Calonectria* in five soil layers in a *Eucalyptus* plantation. Journal of Fungi 7(10): 857.

Liu QL, Chen SF (2017) Two novel species of *Calonectria* isolated from soil in a natural forest in China. MycoKeys 26: 25–60.

Liu QL, Li JQ, Wingfield MJ, Duong TA, Wingfield BD, Crous PW, Chen SF (2020) Reconsideration of species boundaries and proposed DNA barcodes for *Calonectria*. Studies in Mycology 97: 100106.

Liu QL, Li JQ, Wingfield MJ, Duong TA, Wingfield BD, Crous PW, Chen SF (2022) Diversity and distribution of *Calonectria* species from plantation and forest soils in Fujian province, China. Journal of Fungi 8(8): 811.

Liu QL, Wingfield MJ, Duong TA, Wingfield BD, Chen SF (2024) Diversity of *Calonectria* species from leaves and soils in diseased Southern China *Eucalyptus* plantation. Fungal Biology 128: 2007–2021.

Lombard L, Crous PW, Wingfield BD, Wingfield MJ. (2010a) Phylogeny and systematics of the genus *Calonectria*. Studies in Mycology 66: 31–69.

Lombard L, Zhou XD, Crous PW., Wingfield BD, Wingfield MJ (2010b) *Calonectria* species associated with cutting rot of *Eucalyptus*. Persoonia 24: 1–11.

Lombard L, Chen SF, Mou X, Zhou XD, Crous PW, Wingfield MJ (2015a) New species, hyper-diversity and potential importance of *Calonectria* spp. from *Eucalyptus* in South China. Studies in Mycology 80: 151–188.

Lombard L, van der Merwe NA, Groenewald JZ, Crous PW (2015b) Generic concepts in *Nectriaceae*. Studies in Mycology 80: 189–245.

Lombard L, Wingfield MJ, Alfenas AC, Crous PW (2016) The forgotten *Calonectria* collection: pouring old wine into new bags. Studies in Mycology 85: 159–198.

Marin-Felix Y, Groenewald JZ, Cai L, Chen Q, Marincowitz S, Barnes I, Bensch K, Braun U, Camporesi E, Damm U, de Beer ZW, Dissanayake A, Edwards J, Giraldo A, Hernández-Restrepo M, Hyde KD, Jayawardena RS, Lombard L, Luangsa-Ard J, McTaggart AR, Rossman AY, Sandoval-Denis M, Shen M, Shivas RG, Tan YP, van der Linde EJ, Wingfield MJ, Wood AR, Zhang JQ, Zhang Y, Crous PW (2017) Genera of phytopathogenic fungi: GOPHY 1. Studies in Mycology 86: 99–216.

Peerally MA (1973) *Calonectria colhounii* sp. nov., a common parasite of tea in Mauritius. Transactions of the British Mycological Society 61: 89–93.

Pham NQ, Barnes I, Chen SF, Liu FF, Dang QN, Pham TQ, Lombard L, Crous PW, Wingfield MJ (2019) Ten new species of *Calonectria* from Indonesia and Vietnam. Mycologia 111: 78–102.

Pham NQ, Marincowitz S, Chen SF, Yaparudin Y, Wingfield MJ (2022) *Calonectria* species, including four novel taxa, associated with *Eucalyptus* in Malaysia. Mycological Progress 21: 181–197.

Saini A, Pandey S (2024) *Calonectria* *populi* sp. nov., causing leaf blight of *Populus deltoides* in India. World Journal of Microbiology and Biotechnology 40: 15.

Terashita T (1968) A new species of *Calonectria* and its conidial state. Transactions of the Mycological Society of Japan 8: 124–129.

Tubaki K (1958) Studies on the Japanese Hyphomycetes. V. Leaf & stem group with a discussion of the classification of Hyphomycetes and their perfect stages. The Journal of the Hattori Botanical Laboratory 20: 142–244.

Victor D, Crous PW, Janse BJH, Wingfield MJ (1997) Genetic variation in *Cylindrocladium* *floridanum* and other morphologically similar *Cylindrocladium* species. Systematic and Applied Microbiology 20: 268–285.

Wang QC, Liu QL, Chen SF (2019) Novel species of *Calonectria* isolated from soil near *Eucalyptus* plantations in southern China. Mycologia 111(6): 1028–1040.

Zhang YX, Chen CT, Chen C, Chen JW, Xiang MM, Wanasinghe DN, Hsiang T, Hyde KD, Manawasinghe IS (2022) Identification and characterization of *Calonectria* species associated with plant diseases in Southern China. Journal of Fungi 8(7): 719.

**Figure S1.**

**Figure S1. (continued)**

**Figure S1. (continued)**

 **Figure S2.**

**Figure S2. (continued)**

**Figure S2. (continued)**

**Figure S3.**

**Figure S3. (continued)**

**Figure S3. (continued)**

**Figure S4.**

**Figure S4. (continued)**

**Figure S4. (continued)**

**Figure S5.**

**Figure S5. (continued)**

**Figure S5. (continued)**

**Figure S6.**

**Figure S7.**

**Figure S8.**

**Figure S9.**

**Figure S10.**
